# Peopling History of the Tibetan Plateau and Multiple Waves of Admixture of Tibetans Inferred From Both Ancient and Modern Genome-Wide Data

**DOI:** 10.3389/fgene.2021.725243

**Published:** 2021-09-03

**Authors:** Guanglin He, Mengge Wang, Xing Zou, Pengyu Chen, Zheng Wang, Yan Liu, Hongbin Yao, Lan-Hai Wei, Renkuan Tang, Chuan-Chao Wang, Hui-Yuan Yeh

**Affiliations:** ^1^School of Humanities, Nanyang Technological University, Singapore, Singapore; ^2^State Key Laboratory of Cellular Stress Biology, National Institute for Data Science in Health and Medicine, School of Life Sciences, Xiamen University, Xiamen, China; ^3^Department of Anthropology and Ethnology, Institute of Anthropology, School of Sociology and Anthropology, Xiamen University, Xiamen, China; ^4^State Key Laboratory of Marine Environmental Science, Xiamen University, Xiamen, China; ^5^Institute of Forensic Medicine, West China School of Basic Science and Forensic Medicine, Sichuan University, Chengdu, China; ^6^Guangzhou Forensic Science Institute, Guangzhou, China; ^7^Faculty of Forensic Medicine, Zhongshan School of Medicine, Sun Yat-sen University, Guangzhou, China; ^8^School of Medicine, Chongqing University, Chongqing, China; ^9^Center of Forensic Expertise, Affiliated Hospital of Zunyi Medical University, Zunyi, China; ^10^School of Basic Medical Sciences, North Sichuan Medical College, Nanchong, China; ^11^Key Laboratory of Evidence Science of Gansu Province, Gansu Institute of Political Science and Law, Lanzhou, China; ^12^Department of Forensic Medicine, College of Basic Medicine, Chongqing Medical University, Chongqing, China

**Keywords:** East Asian, genetic history, Sino-Tibetan, Tibetan Plateau, ancient genomes

## Abstract

Archeologically attested human occupation on the Tibetan Plateau (TP) can be traced back to 160 thousand years ago (kya) via the archaic Xiahe people and 30∼40 kya via the Nwya Devu anatomically modern human. However, the history of the Tibetan populations and their migration inferred from the ancient and modern DNA remains unclear. Here, we performed the first ancient and modern genomic meta-analysis among 3,017 Paleolithic to present-day Eastern Eurasian genomes (2,444 modern individuals from 183 populations and 573 ancient individuals). We identified a close genetic connection between the ancient-modern highland Tibetans and lowland island/coastal Neolithic Northern East Asians (NEA). This observed genetic affinity reflected the primary ancestry of high-altitude Tibeto-Burman speakers originated from the Neolithic farming populations in the Yellow River Basin. The identified pattern was consistent with the proposed common north-China origin hypothesis of the Sino-Tibetan languages and dispersal patterns of the northern millet farmers. We also observed the genetic differentiation between the highlanders and lowland NEAs. The former harbored more deeply diverged Hoabinhian/Onge-related ancestry and the latter possessed more Neolithic southern East Asian (SEA) or Siberian-related ancestry. Our reconstructed *qpAdm* and *qpGrap*h models suggested the co-existence of Paleolithic and Neolithic ancestries in the Neolithic to modern East Asian highlanders. Additionally, we found that Tibetans from Ü-Tsang/Ando/Kham regions showed a strong population stratification consistent with their cultural background and geographic terrain. Ü-Tsang Tibetans possessed a stronger Chokhopani-affinity, Ando Tibetans had more Western Eurasian related ancestry and Kham Tibetans harbored greater Neolithic southern EA ancestry. Generally, ancient and modern genomes documented multiple waves of human migrations in the TP’s past. The first layer of local hunter-gatherers mixed with incoming millet farmers and arose the Chokhopani-associated Proto-Tibetan-Burman highlanders, which further respectively mixed with additional genetic contributors from the western Eurasian Steppe, Yellow River and Yangtze River and finally gave rise to the modern Ando, Ü-Tsang and Kham Tibetans.

## Introduction

The Tibetan Plateau (TP), widely known as the third pole of the world, forms the high-altitude core region of Asia with an average elevation more than 4,000 meters above sea level (masl). The TP represents one of the most challenging environments for human settlements due to the perennial low temperature, extreme aridity, and severe hypoxia. However, archeological and genetic studies have demonstrated that archaic hominins who occupied the TP had well adapted to the high-altitude hypoxic environment long before the arrival of modern *Homo sapiens.* The present-day Tibetans are suggested to have uniquely adapted to the extreme high-altitude conditions since the initial colonization of the TP (Qi et al., 2013; Jeong et al., 2016; Gnecchi-Ruscone et al., 2018; Chen F. et al., 2019). However, recent linguistic evidence suggested that Tibeto-Burman populations diverged from Han Chinese approximately 5.9 thousand years ago (kya) (Zhang et al., 2019). At present, over seven million indigenous Tibetans (2016 census) are living in the TP and have successfully adapted to the high-altitude hypoxic environment. Genomic analysis found multiple variants that may jointly deliver the high-altitude fitness of the modern Tibetans which is missing in the Hans (Yi et al., 2010). For example, the positively selected haplotypes of *HIF-1*α *prolyl hydroxylase1 (EGLN1)* and *Endothelial PAS domain protein 1 (EPAS1)* were introduced into modern Tibetans and surrounding highlanders via the Denisovan introgression, which further promoted Tibetan’s high-altitude hypoxia adaptation (Huerta-Sánchez et al., 2014). Compared to the well-established population prehistory in other parts of East Asia (He et al., 2020; Ning et al., 2020; Yang et al., 2020; Wang C. C. et al., 2021), the population history of the TP’s was far from clear due to the lack of excavated archeological sites and human remains. For example, there are a limited amount of zooarchaeological and archaeobotanical data for reconstructing the subsistence strategy and ancient DNA (aDNA) data for dissecting the genomic correlation between ancient individuals and modern Tibetan-like highlanders.

To date, when, where, and how the early human colonizers conquered the TP, and who were the ancestors of the modern Tibetans remain unanswered. Archeological, paleo-anthropological, and genetic studies focusing on the peopling processes of the TP and demographic history of Tibetan Highlanders are still in developmental stages (Aldenderfer, 2011). As revealed by the archeological evidence, handprints and footprints of *Homo sapiens* found at the Quesang site in southern TP (4,200 masl) suggested that the intermittent human presence on the TP could trace back to at least 20 kya (Zhang and Li, 2002), and the permanent human occupation was dated to the early Holocene (Meyer et al., 2017). The Nwya Devu site, located nearly 4,600 masl in Central Tibet, could be dated to at least 30 kya, which deepened considerably the history of the peopling of the TP and the antiquity of human high-altitude adaptations (Zhang et al., 2018). The palaeo-proteomic analysis of a Xiahe Denisovan mandible indicated that the prehistoric colonization of archaic hominins on the TP could be traced back to the Middle Pleistocene epoch (around 160 kya) (Chen F. et al., 2019). This Pleistocene colonization of archaic humans was recently evidenced via the Denisovan type of mtDNA found in Xiahe site (Zhang et al., 2020). Additionally, modern human genomic data also provided supporting evidence that humans did exist on the TP before the Last Glacial Maximum (LGM), and the genetic relics of the Upper Paleolithic inhabitants in modern Tibetans indicated some extent of genetic continuity between the initial Paleolithic settlers and modern Tibetan highlanders (Zhao et al., 2009; Qin et al., 2010; Qi et al., 2013; Li et al., 2015; Lu et al., 2016). The archaeogenetic investigation of prehistoric Himalayan populations provided supporting evidence for the high-elevation East Asian origin of the first inhabitants of the Himalayas, indirectly indicating the pre-Neolithic human activities on the TP (Jeong et al., 2016).

In contrast to the Late Pleistocene Hunter-Gatherer colonization, the timing and dynamics of the Holocene permanent human occupation of the TP have also provoked many debates (Ding et al., 2020; Liu W. et al., 2020). Recent archeological and genomic findings suggested that the permanent settlement on the TP was a relatively recent occurrence along with the establishment of farming and pastoralism on the Plateau (Chen et al., 2015; Li et al., 2019). Chen et al. reported archaeobotanical and zooarchaeological data from 53 archeological sites in the northeastern TP (NETP) and illustrated that the novel agropastoral subsistence strategy facilitated year-round living on the TP after 3.6 kya (Chen et al., 2015). The first comprehensive and in-depth genomic investigation of the Tibet sheep also revealed a stepwise pattern of recent permanent human occupation on the TP through the Tang-Bo Ancient Road (from northern China to the NETP ∼3,100 years ago and from the NETP to southwestern areas of the TP ∼1,300 years ago) (Hu et al., 2019). However, it remains unknown who brought the cold-tolerant barley agriculture and livestock to the TP, and how indigenous foragers interacted with the incoming farmers. The archeological observations demonstrated that incoming farmer groups did not replace the local foragers, but co-existed with them for extended periods (Gao et al., 2020; Ren et al., 2020). The mitochondrial evidence and radiocarbon dates of the cereal remains also revealed that millet farmers adopted and brought barley agriculture to the TP around 3.6–3.3 kya. Contemporary Tibetans could trace their main ancestry back to the Neolithic millet farmers (Li et al., 2019). Moreover, the genetic variations of modern Tibetan groups have also been explored based on the forensically available markers (Wang Z. et al., 2018; Zou et al., 2018; He et al., 2019). However, the low resolution of these markers hindered the comprehensive understanding of prehistoric human activities on the TP and impeded the dissection of the ancestral component of Tibetans. Lu and Zhang et al. conducted a series of typical population genomic studies focusing on the demographic history of modern Tibetans and other high-altitude highlanders (Lu et al., 2016; Zhang et al., 2017). They found that Tibetans arose from a mixture of multiple ancestral genetic sources with the co-existence of Paleolithic and Neolithic ancestries.

Collectively, previous studies paved the way toward a better understanding of the Middle Pleistocene arrival, Paleolithic colonization and Neolithic permanent settlement on the TP. However, most of the previous archeological investigations have primarily focused on the NETP (< 4000 masl). Besides, the lack of discussion of ancient samples from the TP and incomprehensive analysis of ancient/modern individuals from East Asia hindered our ability to spatiotemporally connect dispersed ancient East Asians and modern Tibetans. Thus, we comprehensively meta-analyzed the genetic variations of ancient/modern highlanders from the TP and surrounding lowland eastern Eurasians with the aims to (I) portray the genetic landscape of the East Asian highlanders, (II) study the genetic similarities and differences between highlanders and lowlanders, (III) explore the genetic substructure among geographically/culturally different Tibetans, (IV) reconstruct their deep evolutionary history and the corresponding migration and admixture processes. By analyzing genome-wide data of modern Tibetans and Neolithic-to-historic individuals from East Asia, we shed light on the genetic transition, turnover or continuity, ancestral composition, and demographic history of Tibetan highlanders.

## Materials and Methods

### Publicly Available Dataset

**FIGURE 1:**
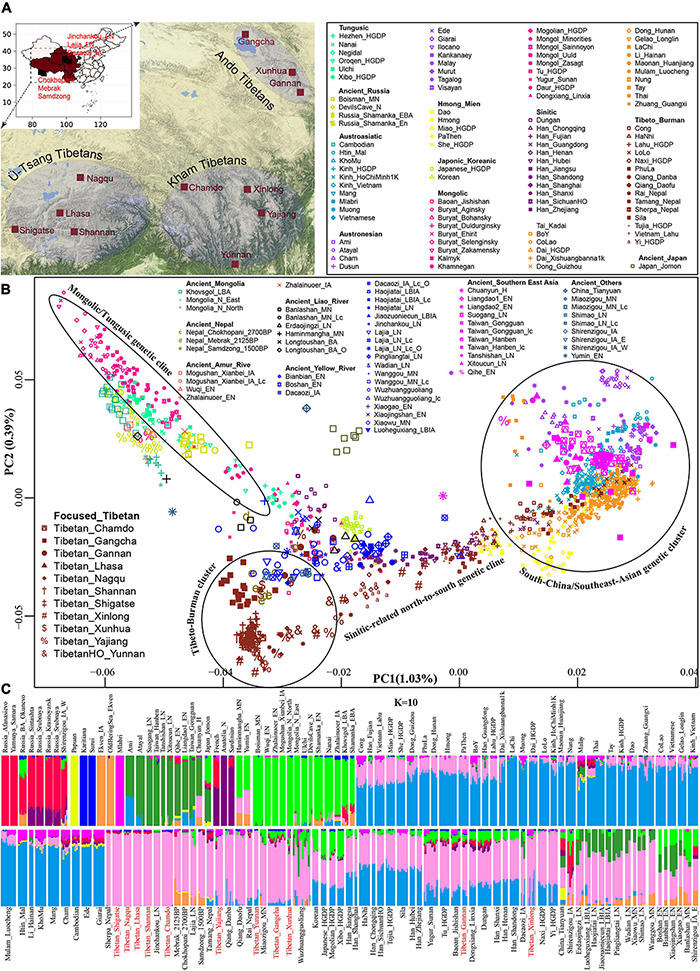
The geographical position of the focused Tibetans and genetic patterns of East Asians. **(A)** Sampling place of eleven geographically different modern Tibetan populations mainly discussed in the present study from the five provinces (Tibet Tibetan Autonomous Region, Qinghai, Gansu, Sichuan, and Yunnan) from western China. China map is presented in the top-left of **A** and five studied western provinces were zoom-in as the presented Google map. **(B)** Principal component analysis (PCA) showed the genetic similarities and differences between the ancient/modern East Asians from geographically/linguistically/culturally different populations. Spatial-temporally diverse ancient populations were projected onto the two-dimensional genetic background of modern East Asians. **(C)** Admixture ancestry estimation based on the model-based ADMIXTURE. Here, the optimal predefined ten ancestral populations were used. EN, early Neolithic; MN, middle Neolithic; LN, late Neolithic; IA, iron age; BA, bronze Age; LBIA, late bronze age and iron age; Lc, loc coverage; O, outlier.

We collected 2,444 individuals from 183 geographically/culturally different populations (Patterson et al., 2012; Lipson et al., 2018a; Jeong et al., 2019; Liu D. et al., 2020) belonging to fifteen language families or groups: Altai (also referred to as Trans-Eurasian including Mongolic, Japonic, Koranic, Tungusic, and Turkic), Sino-Tibetan (Sinitic and Tibeto-Burman), Hmong-Mien, Austronesian, Austroasiatic, Uralic, Caucasian, Chukotko-Kamchatkan, Eskimo-Aleut, Indo-European and Tai-Kadai. The 383 modern East Asian individuals genotyped via the Affymetrix Human Origins array were also used here (Wang C. C. et al., 2021). To explore the genomic history of modern Tibetans and elucidate the peopling process of the TP, we focused on the genome-wide data of 98 modern Tibetans collected from eleven geographically different regions with different cultural backgrounds, which includes five Ü-Tsang Tibetan groups from Tibet Autonomous Region, three Ando Tibetan groups from Qinghai and Gansu, four Kham Tibetan groups from Sichuan, Yunnan, and Tibet (Figure 1A). Raw data were quality-controlled using the PLINK v.1.9 (Chang et al., 2015) following the standard threshold (Wang C. C. et al., 2021; Yao et al., 2021). Besides, Paleolithic-to-historic published ancient genomes from East Eurasia (Russia, China, Mongolia, Nepal and Southeast Asia) were collected from recent ancient DNA studies or from Allen Ancient DNA Resource (AADR) released by Reich Lab (Jeong et al., 2016; Yang et al., 2017, 2020; Ning et al., 2020; Wang C. C. et al., 2021). A total of 161 Paleolithic to historic East Asians and eight Nepal ancients were collected and first comprehensively meta-analyzed and discussed (Jeong et al., 2016; Yang et al., 2017, 2020; Ning et al., 2020; Wang C. C. et al., 2021). Detailed information of key ancient populations is presented in Table 1.

**TABLE 1:** The detailed information of included ancient Chinese populations.

**Ancient populations**	**Time period**	**Sample size**	**Archeological site**	**Testing platform**	**Y haplogroup types**	**MtDNA haplogroup types**	**Reference**
China_Xinjian_IA	Iron Age	11	Shirenzigou	Shotgun	O2a, Q1a1, Q1a2a2b1∼, R1a1a1, R1b and R1b2b2	A17, D4j1b, G3b, H15b1, I1b, T1a1b, U4, U4′9, U5a2 and U5b2c	Ning et al., 2019
Wuzhuangguoliang_LN	Late Neolithic	12	Wuzhuangguoliang	Exome.capture	F(3), C	A7, A + 152 + 16362, B4a2b1, B4a4 (2), D4q, D5a3, F1g1, G2a1, M11a and R11a	Wang C. C. et al., 2021
China_AR_EN		2	Wuqi	1240K	.	C4a1 and C5	Ning et al., 2020
China_AR_IA	Iron Age	1	Zhalainuoer	1240K	.	N9a9	Ning et al., 2020
China_AR_Xianbei_IA	Iron Age	3	Mogushan Xianbei	1240K	.	C5a1, C4a1a4 and Z3a1	Ning et al., 2020
China_HMMH_MN	Middle Neolithic	1	Haminmangha	1240K	.	D4j	Ning et al., 2020
China_Miaozigou_MN	Middle Neolithic	3	Miaozigou	1240K	.	A14, C4a2a1 and D4b2	Ning et al., 2020
China_NEastAsia_Inland_EN	Early Neolithic	1	Yumin	1240K	.	.	Yang et al., 2020
China_SEastAsia_Coastal_EN	Early Neolithic	1	Qihedong	1240K	.	.	Yang et al., 2020
China_SEastAsia_Coastal_His	Historic	1	Chuanyundong	1240K	O1a1a1a1a1a1	.	Yang et al., 2020
China_SEastAsia_Coastal_LN	Late Neolithic	11	Xitoucun and Tanshishan	1240K	F, K2, NO, O, O1a2, O1b1a1a1a and O2a	.	Yang et al., 2020
China_SEastAsia_Island_EN	Early Neolithic	4	Liangdao	1240K	O1a, O and O2a2b	.	Yang et al., 2020
China_Shimao_LN	Late Neolithic	3	Shengedaliang	1240K	.	G2a1, D5a2a1b, M80′D	Ning et al., 2020
China_Upper_YR_IA	Iron Age	4	Dacaozi	1240K	.	D4b2b, G2b1b, F1g and Z3	Ning et al., 2020
China_Upper_YR_LN	Late Neolithic	7	Jinchankou and Lajia	1240K	.	B4c1b2c2, G3a2, A18, F1g, G1c1, F1a1a and F1g	Ning et al., 2020
China_WLR_BA		3	Longtoushan	1240K	.	D4m1, D4j14 and B4c1a2	Ning et al., 2020
China_WLR_LN	Late Neolithic	3	Erdaojingzi	1240K	.	B5b1a, A22 and N9a1	Ning et al., 2020
China_WLR_MN	Middle Neolithic	3	Banlashan	1240K	.	D5a3a1 and D5a3a1	Ning et al., 2020
China_YR_LBIA	Late Bronze Age/Iron Age	6	Haojiatai, Jiaozuoniecun, Luoheguxiang	1240K	.	M8a2b, B4d1′2′3, F4a2, C4a1a2 and A5b1b	Ning et al., 2020
China_YR_LN	Late Neolithic	8	Pingliangtai, Haojiatai and Wadian	1240K	.	D4b1a (3), D5a2a, F2h, N9a2, D4 and D4e1a	Ning et al., 2020
China_YR_MN	Middle Neolithic	8	Xiaowu and Wanggou	1240K	.	F1, M8a2, D4g2a1, B4d1 and C4a1a1	Ning et al., 2020
Nepal_Chokhopani_2700BP	Iron Age	1	Chokhopani	Shotgun	O2a2b1a1a1a4a1	D4j1b	Jeong et al., 2016
Nepal_Mebrak_2125BP	Iron Age	3	Mebrak	Shotgun	O2a2b1	Z3a1a, M9a1a1c1b1a and M9a1a2	Jeong et al., 2016
Nepal_Samdzong_1500BP	Historic	4	Samdzong	Shotgun	O2a2b1a1a1a4a1 (2), D1a1a1	M9a1a1c1b1a, M9a1a, M9a1a, F1c1a1a and F1d	Jeong et al., 2016
Russia_DevilsCave_N	Early Neolithic	6	Devil’s Gate Cave	Shotgun	C2a	D4m (3) and D4(2)	Sikora et al., 2019
Taiwan_Gongguan	Late Neolithic	2	Gongguan	1240K	.	Y2a1	Wang et al., 2020
Taiwan_Hanben_IA	Iron Age	45	Hanben	1240K	O2a2b2a2b(4), O1a1a1a1(3), O1a1a1a(2), O1a2(2), F(2), O2a2b2b(1), O2a2a1a2(1), O(1), O1a1a1a1a1a(1), O2a2b(1), O1a(1)	F4b1(5), R(5), E1a1a1(4), F3b1a + 16093(4), B4a1a(3), E1a1a(3), D6a2(2), E1a(2), M7b1a2a1(2), E2a(2), F1a3a(1), B4b1a2(1), F3b1(1), E2b(1), R30(1), F3b1a2(1), R9c1b2(1), M7c1c3(1), F4b(1), F3b1a(1), B4b1a2f(1), E1a1(1) and B5a2a1 + 16129(1)	Wang et al., 2020
AR33K	Paleolithic	1	AR33K	1240K	.	.	Mao et al., 2021
Longlin	Paleolithic	1	Longlin	1240K	.	M71 + 151	Yang et al., 2020
China_Tianyuan	Paleolithic	1	Tianyuan	1240K	K2b	.	Yang et al., 2017
Russia_Altai.DG	Paleolithic	1	Altai	1240K	.	.	Reich et al., 2010

### Principal Component Analysis

We performed principal component analysis (PCA) with the *smartpca* program of the EIGENSOFT package (Patterson et al., 2006) using the default settings with additional parameters: lsqproject: YES and numoutlieriter: 0. Population data of modern East Asia were used to reconstruct the genetic background of PCA, in which modern samples were mainly sampled from Altaic, Sino-Tibetan, Hmong-Mien, Austronesian, Austroasiatic, and Tai-Kadai language families. Ancient genomes were projected onto the first two components. The projected ancient populations included eight individuals from Nepal (Jeong et al., 2016) (Chokhopani, Samdzong, and Mebrak cultures), eighty-four samples from the Yellow River (Ning et al., 2020; Yang et al., 2020; Wang C. C. et al., 2021), Amur River and West Liao River in the coastal and inland northern East Asia (including Houli, Yangshao, Longshan, Qijia, Hongshan, Yumin and other cultures), fifty-eight individuals (Ning et al., 2020; Yang et al., 2020; Wang C. C. et al., 2021) belonging to Tanshishan and other cultures in the coastal southeast East Asia (Fujian and Taiwan).

### *F*_*ST*_ Calculation and TreeMix Analysis

We used the Plink 1.9 and an in-house script to estimate the pairwise *F*_*ST*_ genetic distance (Purcell et al., 2007) among 82 modern populations with a sample size large than five. We also calculated *F*_*ST*_ values among 31 ancient populations. We ran TreeMix v.1.13 (Pickrell and Pritchard, 2012) with migration events ranging from 0 to 8 to construct the topology among eastern Eurasians with the maximum likelihood tree.

### ADMIXTURE Analysis

We carried out the model-based clustering analysis using the ADMIXTURE (v.1.3.0) (Alexander et al., 2009) after pruning SNPs with a strong linkage disequilibrium via the PLINK v.1.9 (Chang et al., 2015) with the parameters of – indep-pairwise 200 25 0.4. We ran ADMIXTURE with the 10-fold cross-validation (−cv = 10). The predefined number of ancestral populations ranging from *K* = 2 to *K* = 20 with 100 bootstraps and different random seeds were used. We chose the best-fitted model with the minimum cross-validation errors. The smallest cross-validation error was obtained (0.4176) when we used 10 predefined ancestral sources.

### *F*-Statistics and Admixture Modeling Graph

We conducted two different forms of the three-population tests using the *qp3Pop* program implemented in the ADMIXTOOLS (Reich et al., 2009; Patterson et al., 2012). Outgroup-*f*_3_-statistics were performed in the form of *f_3_(Reference Eurasians, targeted Tibetans; Mbuti)* to assess the shared genetic drift between our focused Tibetans and their reference populations. A central African population Mbuti was used as the outgroup. Admixture-*f_3_(Surrogate population1, Surrogate population2; Targeted populations)* were performed to test whether our targeted population was an admixture of two sources related to our used surrogate populations. Negative *f*_3_-values with a Z-score smaller than −3 indicated that two source populations were admixed to form the targeted populations. Four-population comparisons were conducted using *qpDstat* programs implemented in the ADMIXTOOLS (Reich et al., 2009; Patterson et al., 2012) with the additional parameter (*f*_4_: YES) in three different forms. The first one was conducted in the form of *f_4_(Tibetan1, Tibetan2; Eurasian reference, Mbuti)* to test whether two Tibetans form one clade relative to the used Eurasian reference. Non-statistically significant *f*_4_ values showed two left populations formed one clade. Other two *f*_4_-statistics in the forms *f_4_(Eurasian, Source; Eurasian2, Mbuti)* and *f_4_(Eurasian1, Eurasian2; Source, Mbuti)* were conducted to examine whether the used ancestral source shared more alleles with one of the Eurasians compared with others. We assessed standard errors using the weighted block jackknife approach. We next used the *qpGraph* program implemented in the ADMIXTOOLS (Reich et al., 2009; Patterson et al., 2012) to reconstruct the deep population history of modern Tibetans and other modern and ancient East Asians based on the combined results of the *f*_2_, *f*_3_ and *f_4_-statistics*. The absolute Z-scores smaller than 3 indicated better-fitted models.

### Streams of Ancestry and Inference of Mixture Proportions

We used the *qpWave/qpAdm* programs implemented in the ADMIXTOOLS (Haak et al., 2015) to estimate mixture coefficient and corresponding standard errors according to a basic set of outgroup populations: Mbuti, Ust_Ishim, Russia_Kostenki14, Papuan, Australian, Mixe, MA1, and Mongolia_N_East.

## Results

### Close Genetic Affinity Between Ancient/Modern Tibetans With NEAs

Descriptive analyses of PCA and ADMIXTURE were first used to provide an overview of the genetic structure. All modern Tibetans and Neolithic-to-historic East Asians were grouped in the East-Asian genetic cline along with the second component in the Eurasian-PCA. To focus on the genetic variations of East Asians, we constructed East-Asian-PCA among 106 modern populations (Figure 1B) and found that modern East Asians grouped into four genetic clines or clusters: Mongolic/Tungusic genetic cline consisting of populations from northeast Asia; south-China/Southeast-Asian genetic cluster comprising of Austronesian, Austroasiatic, Tai-Kadai, and Hmong-Mien speakers; Sinitic-related north-to-south genetic cline, and Tibeto-Burman cluster, which were consistent with the linguistic/geographical divisions. Tibetan populations were grouped and showed a relatively close relationship with some of the Mongolic/Tungusic speakers in northern China, and they were also grouped closely with northern Han and other lowland Tibeto-Burman speakers. Focused on the population substructures within Tibetans, we further observed three different sub-clusters: the high-altitude Tibet-Ü-Tsang cluster (Lhasa, Nagqu, Shannan and Shigatse), Gan-Qing-Ando cluster in northeastern TP (Xunhua, Gangcha and Gannan) and Tibetan-Yi-corridor cluster (Chamdo, Xinlong, Yajiang and Yunnan), which were also consistent with the geographical positions of sampling places and cultural backgrounds.

We subsequently explored the patterns of genomic affinity between ancient populations and modern East Asians by projecting all included ancient individuals (243 eastern Eurasian ancients) onto the genetic background of modern populations. Here, we found four ancient population genetic clusters. Neolithic-to-historic SEAs (including Hanben and Gongguan from Taiwan, Late-Neolithic mainland Tanshishan and Xitoucun people) grouped together and clustered with modern Tai-Kadai, Austronesian, and Austroasiatic speakers. Neolithic-to-Iron Age NEAs (both coastal Shandong Houli and inland Yangshao, Longshan, and Qijia people) grouped together and were projected closely to the juncture position of three main East Asian genetic lines and the northmost end of Han Chinese genetic cline. We observed a close genetic relationship between early Neolithic Houli individuals associated with the main subsistence strategy of hunter-gathering and the Henan Middle/Late-Neolithic Yangshao/Longshan farmers, which indicated the genetic continuity in the Neolithic transition from foragers to millet farmers in the early Neolithic northern China. We also identified the subtle genetic differences within these Neolithic-to-Iron Age individuals from northern China. These Shandong Houli individuals were localized closely with modern Mongolic-speaking Baoan, Tu, Yugur, and Dongxiang, while the early Neolithic Xiaogao individuals were posited closely with modern Tungusic-speaking Hezhen and Xibo. All Shandong Neolithic ancient populations were localized distantly from the modern Shandong Han Chinese and shifted to modern northern Chinese minorities, which indicated that modern northern Han received additional gene flow from SEA related ancestral lineage or ancient Houli individuals harbored more Siberian-associated ancestry. Late-Neolithic Longshan individuals (Pingliangtai, Haojiatai, and Wadian) and Bronze/Iron Age individuals (Haojiatai, Jiaozuoniecun, and Luoheguxiang) in Henan province were grouped together and shifted to the Han Chinese genetic cline and partially overlapped with Han Chinese from Shanxi and Shandong provinces. This observed genetic similarities among the Late Neolithic to present-day NEAs from the Central Plain (Henan, Shanxi, and Shandong) indicated a genetic stability in the core region of Chinese civilization since the Late-Neolithic period. Middle-Neolithic Yangshao individuals (Xiaowu and Wanggou) in Henan province grouped with some of the Wuzhuangguoliang_LN individuals collected from Shaanxi province and were shifted to more northern modern minorities. The inland Middle/Late-Neolithic NEAs from Shaanxi (Shimao), Inner Mongolia (Miaozigou) and upper Yellow River (Lajia and Jinchankou) clustered together and were shifted toward modern Tibetans and ancient Nepal samples (Mebrak, Samdzong and Chokhopani).

For ancient populations from the West Liao River, three genetic-affinity clusters could be identified in the projected PCA results: northern cluster (Haminmangha_MN and Longtoushan_BA_O) showed a genetic affinity with Shamanka and Mongolia Neolithic people; middle Hongshan cluster was localized between Mongolia minorities and modern Gangcha Tibetan; southern cluster (Upper Xiajiadian Longtoushan_BA and Erdaojingzi_LN) possessed close relationship with the Yellow River farmers, which suggested that both Neolithic ancients associated with steppe pastoralists from Mongolia Plateau and millet farmers from Yellow River Basin had participated in the formation of the Late Neolithic and subsequent populations in the West Liao River Basin. These population movements, interactions, and admixture processes have recently been fully elucidated by Ning et al. (2020). Here, we observed that the Late Neolithic populations in the southern cluster were localized between the coastal early Neolithic NEAs and inland Neolithic Yangshao and Longshan individuals, which indicated that millet farmers from the middle/lower Yellow Rivers (Henan and Shandong) had played an important role in the formation of Hongshan people or their descendants via both inland and coastal northward migration routes. For ancient populations from Mongolia Plateau, Russia Far East, Trans-Baikal-Region, and Amur River Basin, all included forty-six individuals (Neolithic-to-Bronze Shamanka, Mongolian, DevilsCave, Boisman, and others) clustered closely to modern Tungusic language speakers (Nanai and Ulchi) and also to some Mongolic speakers. Jomon individuals were grouped together in the intermediate position between the northern Russian coastal Neolithic people and southern Iron Age Taiwan Hanben and coastal Neolithic SEAs, but localized far away from modern Japanese populations.

Patterns of genetic relationship revealed from the top two components (extracting 1.42% variation: PC1: 1.03% and PC2: 0.39%) showed a genomic affinity between modern Tibetans, ancient Nepal populations, and ancient/modern East Asians and Siberians. To further explore the genetic structure and corresponding population relationships, we estimated the ancestry composition and cluster patterns according to the model-based maximizing likelihood clustering algorithm (Figure 1C and Supplementary Figure 1). We observed two northern and two southern East Asian dominant ancestries. The coastal NEA ancestry (light green) maximized in Neolithic northeast Asians (Boisman_MN, Wuqi_EN, Zhalainuoer_EN, Mongolia_N_North, Mongolia_N_East, DevilsCave_N and Shamanka_EN) and modern Tungusic speakers (Ulchi and Nanai). This light green ancestry also existed in the Bronze Age to present-day populations from northeastern China and Russia, and reached at a high proportion in the coastal Early Neolithic NEAs from Shandong. The other type of northern ancestry was enriched in modern highland Tibetans and Qijia culture-related Late Neolithic Lajia and Jinchankou populations, which also maximized in Nepal Bronze Age to historic individuals and ancient NEAs, as well as the lowland modern Sino-Tibetan speakers, inland Hmong-Mien and Tai-Kadai language speakers. We named this Tibetan-associated ancestry as inland NEA ancestry, which was the direct indicator of the close genetic affinity between Tibetan and ancient/modern NEAs. Dark green ancestry was enriched in the coastal Early Neolithic SEAs, Iron-Age Hanben, and modern Austronesian Ami and Atayal. Therefore, we referred to this dark green component as the coastal SEA ancestry. The blue component maximized in LaChi samples as the counterpart of the coastal ancestry that was widely distributed in Hmong-Mien and Tai-Kadai-speaking populations. This blue inland SEA ancestry also existed in the lowland Tibetans with a relatively high proportion in all Kham and Ando Tibetans except for Chamdo Tibetans. Besides, we found that Tibetans collected from the northeast TP harbored more coastal NEA ancestry. Some Austroasiatic-associated dark pink ancestry maximized in Mlabri also appeared in Yajiang, Xinlong Kham, and Xunhua and Gannan Ando Tibetans. The Steppe pastoralist-like red component was enriched in Bronze Age Afanasievo and Yamnaya, which was also identified in Qinghai and Gansu Ando Tibetans.

### Population Differentiation Between Highland and Lowland East Asians and Substructure Among Tibetans

To further explore the genetic differentiation between eleven modern Tibetan populations and ancient/modern reference populations, we first calculated the pairwise *F*_*ST*_ genetic distances among 82 modern populations (Supplementary Table 1, modern dataset) and 32 ancient/modern populations (Supplementary Table 2, ancient dataset). We found a strong genetic affinity among geographically close populations. As shown in Supplementary Figures 2, 3, the high-altitude Tibetans from the south (Shigatse and Shannan), central (Lhasa), north or northeast (Nagqu and Chamdo) of Tibet Autonomous Region had the smallest *F*_*ST*_ genetic distances with their geographical neighbors, followed by lowland Ando Tibetans from the northeastern TP (Qinghai and Gansu) and the Kham Tibetans from the southeastern region of the TP (Sichuan and Yunnan) and other Tibeto-Burman-speaking populations (Qiang, Tu and Yi). For Ando Tibetans from the Ganqing region, Gangcha Tibetan harbored a close genetic affinity with northern or northeastern Tibet Tibetans (Chamdo and Nagqu) with the smallest *F*_*ST*_ genetic distances, followed by Qiang, Yugur, and Tu or other geographically close Tibetans (Supplementary Figure 4). Different patterns were observed in Gangcha and Xunhua Tibetans, which showed the closest relationship with each other, and then followed by Tu and Yugur. We also found relatively small genetic distances between Tibetans (Gannan and Xunhua) and the Turkic-speaking Kazakh population, suggesting a western Eurasian affinity of Tibetans from the northeastern region of the TP relative to the Tibetans from the central region. Supplementary Figure 5 presented the patterns of genetic differentiation between lowland Kham Tibetans and their reference populations. We found that Yajiang and Xinlong Tibetans from Sichuan province harbored a close genetic affinity with the geographically close populations (Tibetan, Qiang, Yugur and Tu). Yunnan Tibetans had the smallest genetic distance with Gangcha and Chamdo Tibetans, followed by Qiang, Yi, and Tu. Among Tibetans and Neolithic to Iron Age East Asians (Supplementary Figure 6), we also found Iron Age Hanben population from Taiwan and some southern Siberian ancients showed a closer relationship with modern Tibetans relative to other ancient East Asians. We should note there might be statistical bias in the *F*_*ST*_-based analyses because of the different sample sizes in different populations.

**FIGURE 2:**
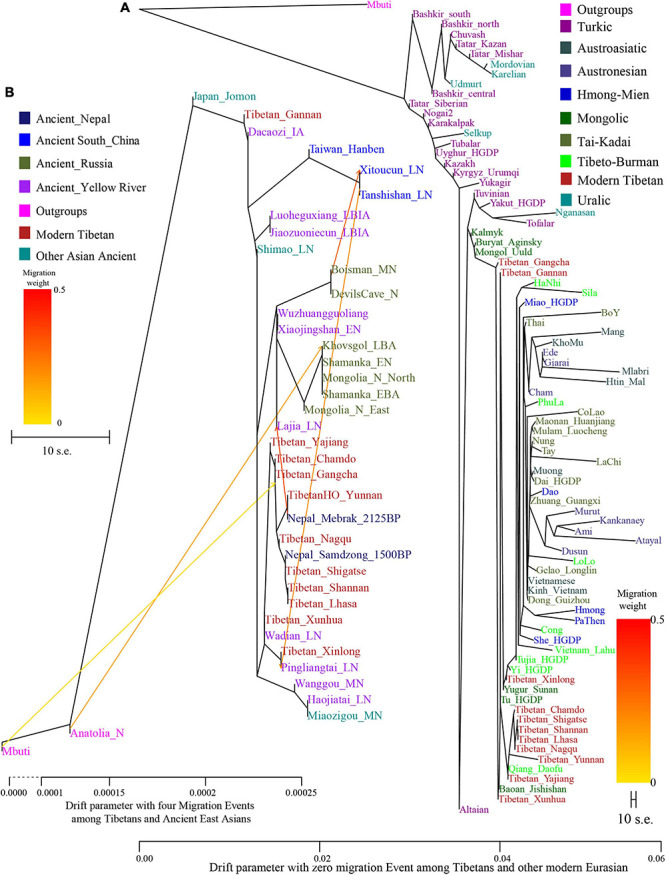
Maximum likelihood phylogeny reconstruction based on the genetic variation from both modern Tibetan and Eurasian modern reference populations. **(A)**, modern Tibetan and Neolithic-to-historic East Asian **(B)**. Mbuti was used as the root. Focused on the phylogenetic relationship among all modern populations, we used the patterns of genetic relationship with zero migration events. And evaluating the evolutionary history among modern Tibetan and ancient Chinese, we included three migration events. To better present our result, the drift branch length of Mlabri was shortened as the third of the truth drift branch length due to the strong genetic drift that occurred in Mlabri.

Phylogenetic relationships were further reconstructed based on the genetic variations of modern Eurasian populations and ancient eastern Eurasians using TreeMix software based on genetic distances. As shown in Figure 2, a phylogenetic tree with no migration events showed that modern populations from similar language families tended to cluster into one clade. Altaic-speaking (Turkic and Mongolic) populations clustered with Uralic speakers. Southern Austronesians first clustered with Tai-Kadai speakers and then clustered with Hmong-Mien and Austroasiatic speakers. Tibetans first clustered with each other, especially for high-altitude Ü-Tsang Tibetans, and then clustered with the lowland East Asians. The observed geographical affinity showed that the genetic differentiation between modern highland Tibetans and lowland East Asians could be identified although they both derived majority of their ancestry from Neolithic Yellow River farmers. We further analyzed the population splits and gene flow events between modern Tibetans and 26 ancients from eastern Eurasia (except for Anatolia_N from Near East) with three predefined admixture events. Modern Tibetans (except for Gannan and Xinlong Tibetans) first clustered with the highland Nepal ancients and then clustered with the lowland Neolithic-NEAs and Neolithic to Bronze Age southern Siberians. The cluster patterns also showed a distant relationship between northern and southern East Asians, as well as the genetic distinction between the highland ancient/modern Tibetans and the lowland SEAs, which further provided evidence for some special connections or close genetic relationships between Tibetans and NEAs.

**FIGURE 3:**
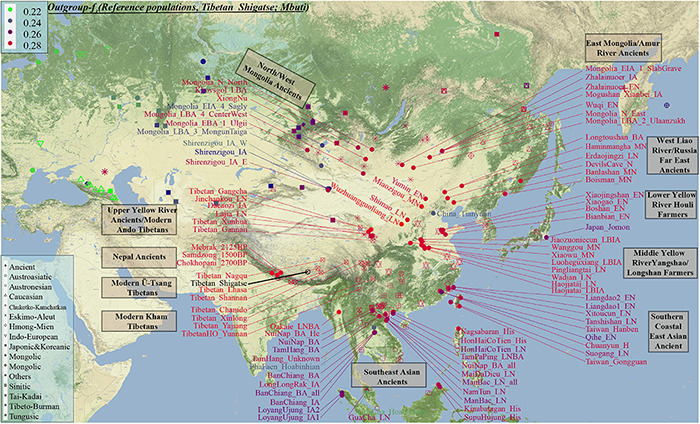
The genomic affinity between our Shigatse Tibetan populations and other modern and ancient spatial-temporally different eastern Eurasian populations. The red color denoted a stronger genetic affinity with Shigatse Tibetans, and the blue color showed a lower genetic affinity.

Genetic affinity was further evaluated via the outgroup-*f*_3_-statistics in the form *f_3_(modern Tibetans, ancient/modern Eurasians, Mbuti)*. We found a close genetic affinity within Tibetan populations and identified the genetic connection between Tibetan and Han Chinese. Among 184 modern populations (Figure 3 and Supplementary Table 3), the top allele sharing population for each Tibet Tibetan was another geographically close Tibetan group. Shannan Tibetan shared the most alleles with Lhasa/Shigatse/Nagqu Tibetans, and similar patterns of population affinity were identified in southern Shigatse Tibetan and central Lhasa Tibetan. However, Nagqu Tibetan shared the most alleles with the northeastern Chamdo Kham Tibetan (followed by Tibetan-Burman-speaking Qiang from Sichuan province and other Tibetans or Sherpa), and these patterns of genetic affinity were consistent with that of Chamdo Tibetan and others. Following the genomic affinity within Tibetans, we also found that these five Tibet Tibetans shared the strongest genetic affinity with the lowland Han Chinese, which was consistent with the common origin of Sino-Tibetan speakers from the Upper and Middle Yellow River Basin (YRB). For Sichuan/Yunnan lowland Kham Tibetans, Xinlong Tibetan shared the most genetic drift with Han Chinese and other lowland Tibeto-Burman-speaking Qiang and Tujia. Being different from Xinlong Tibetan, geographically close Yajiang and Yunnan Tibetans shared the most genetic drifts with Qiang and geographically close Tibetans (Chamdo and Xinlong), followed by Han Chinese and other Tibetans. These lowland Han/SEA affinities of Kham Tibetans suggested that lowland Tibetans from southwestern China harbored ancestry that derived from SEAs via the massive migrations and admixtures in the prehistoric/historic times. Gangcha Ando Tibetan not only showed the genetic affinity with Sinitic and Tibeto-Burman speakers but also showed the signals of genetic affinity with Turkic-speaking populations. Allele sharing results from Gannan and Xunhua Tibetans showed that the Han Chinese groups shared the most ancestry components with them.

Levels of allele sharing between modern Tibetans and 106 Paleolithic to historic Eurasian ancients (including 33 populations from Russia, 41 from China, 29 from Mongolia, and 3 from Nepal) inferred from the outgroup-*f*_3_-statistics showed that modern Tibetans had a clear connection with ancient Neolithic to Iron Age NEAs, which was consistent with the patterns observed in the PCA, *F*_*ST*_, ADMIXTURE and modern population-based affinity estimations (Supplementary Table 3). Middle-altitude Chamdo Tibetan shared the most genetic drift with Neolithic Wuzhuangguoliang_LN (low coverage samples), upper Yellow River Late Neolithic farmers (Jinchankou and Lajia, which are the represented typical source populations for Qijia culture), followed by Iron Age Dacaozi people, Shimao people from Shaanxi, Middle-Neolithic Banlashan associated with Hongshan culture in northern China and other NEAs from lower and middle YRB (Supplementary Figure 7). Neolithic people from Russia and Mongolia and Bronze to historic Nepal ancients showed a relatively distant genetic relationship with modern Chamdo Tibetan (Supplementary Table 3). Different from the pattern of Chamdo Tibetan, southern and central Ü-Tsang Tibetans showed increased ancestry associated with Nepal ancient people, and northern Nagqu Tibetan showed the intermediate trend of population affinity with 2700-year-old Chokhopani. As showed in Supplementary Figures 8, 9, lowland Tibetans from southwestern China and northeastern China showed a similar population affinity with NEA ancients. The genomic affinity between modern Tibetans and some southern East Asians (such as Oakaie_LNBA) could be also identified in Figure 3.

### Admixture Signatures of Modern Tibetans and Ancient Populations From Tibetan Plateau

We carried out admixture-*f*_3_-statistics in the form *f_3_(source population1, source population2; Targeted Tibetan)* to detect the signals of recent genetic admixture in Tibetans. We also re-evaluated the admixture signatures in the eight ancient individuals from Nepal and eleven ancient individuals from Qinghai province using this three-population comparison testing and our comprehensive ancient/modern reference dataset. We found different patterns of admixture signals and source populations in the highland/lowland ancient/modern Tibetans (Supplementary Tables 4–18). Besides, we also identified small but significant differences within geographically/culturally different Tibetans. By setting the statistically significant threshold at Z-score < −3, no admixture signals were observed in southern Tibetans (Shannan and Shigatse) over forty thousand tested pairs, and only four pairs in central Lhasa Tibetan with one source from 1500-year-old Samdzong and other from Kham Tibetan/Qiang, or the combination of southern Tibet Tibetan with Neolithic-NEAs or Baikal ancients (Supplementary Tables 4–6). It was interesting to find that 188 tested population pairs showed statistically significant *f*_3_-statistic values with one source from Tibeto-Burman speakers and the other from Western Eurasian Steppe pastoralists (Alan, Andronovo, Sintashta, Poltavka, and Yamnaya) in *f_3_(Source1, Source2; Nagqu Tibetan)*. Tibetans from southern and central Tibet combined with the lowland modern East Asians, but not with ancient East Asians, could also produce significant admixture signals for Nagqu Tibetan (Supplementary Table 7). Chamdo Tibetan at the junction regions between Ü-Tsang Tibetan and Kham Tibetan had the potential possibility of cultural contact and population admixture, but only one pair of source populations could give a significant admixture signal in Chamdo Tibetans: *f_3_(Lhasa Tibetan, Yajiang Tibetan; Chamdo Tibetan)* = −*3.49^∗^SE* (Supplementary Table 8). Three Tibetans from the Gansu-Qinghai region possessed admixture signatures from over several thousand population pairs with one from modern or ancient East Asians and the other from Western Eurasians (Supplementary Tables 9–11). Results from *f_3_(Yumin_EN, Austronesian/Tai-Kadai; Gansu-Qinghai Tibetan)* showed that the combination of inland Neolithic NEA of Yumin_EN as northern ancestral source with Austronesian/Tai-Kadai speakers as the southern ancestral source could produce significant negative *f*_3_-values, and these admixture signals could also be identified in *f_3_(Neolithic NEAs, Neolithic-Russian/modern Turkic/Mongolic/Indo-European speakers; Gansu-Qinghai Tibetan)*. Tibetans from Sichuan province only showed significant signals as an admixture between northern and southern East Asians or the highland Tibeto-Burman speakers and lowland East Asians, i.e., *f_3_(highland Tibeto-Burman speakers, lowland Tibeto-Burman speakers; Sichuan Tibetan)* < −*3^∗^SE* (Supplementary Tables 12, 13). Similar to the southern Tibet Tibetans, no obvious admixture signals were observed in Yunnan Tibetans, which may be caused by the genetic isolation or obvious genetic drift that occurred recently (Supplementary Table 14). The statistics focused on the ancient populations from the TP showed seven pairs can give admixture signals for modeling Qinghai Iron Age Dacaozi samples (Supplementary Tables 15–18), which are the pairs of ancient NEAs and modern SEAs, or Chamdo Tibetan-related source and Taiwan Iron Age Hanben-like populations.

### Intra Population Differentiation Amongst High-Altitude and Low-Altitude Residing Tibetans Inferred From *f*_4_-Statistics

To gain insights into the population substructures among modern Tibetans, we first conducted symmetry-*f*_4_-statistics in the form *f_4_(modern Tibetan1, modern Tibetan2; modern Tibetan3, Mbuti)*, in which we expected to observe the non-significant *f*_4_-values if no significant differences existed between different Tibetan groups. As shown in Supplementary Table 19 and Supplementary Figure 10, we observed that Chamdo Tibetan formed a clade with Nagqu/Yunnan Tibetans compared with others in *f_4_(Tibetan1, Chamdo Tibetan; Tibetan2, Mbuti)* and all included Tibetans shared more alleles with Chamdo Tibetan compared with Ando Tibetans. Compared to the low-altitude Sichuan Tibetans, Chamdo Tibetan had more high-altitude Tibetan-related ancestry, while Gannan Tibetan shared more alleles with Xinlong Tibetan compared with Chamdo Tibetan. Compared with high-altitude Tibetans, Chamdo Tibetan shared more alleles with other low-altitude Tibetans. Results from the symmetry-*f_4_(Shigatse/Shannan/Lhasa Tibetans, Shigatse/Shannan/Lhasa Tibetans; Tibetan2, Mbuti)* with non-significant Z-scores showed clear genetic homogeneity among Tibet central/southern-Ü-Tsang Tibetans (Supplementary Figures 11, 12). Negative-*f*_4_-values in *f_4_(Gansu-Qinghai Ando Tibetans, Shigatse/Shannan/Lhasa Tibetan; Tibetans, Mbuti)* showed that all included Tibetans shared more alleles with southern Tibet Tibetans relative to Gansu-Qinghai Ando Tibetans. However, northern Tibet Tibetans formed a clade with Chamdo and Yunnan Tibetans and received more high-altitude Tibetan-related derived alleles compared with Gansu-Qinghai and Sichuan Tibetans. For lowland Tibetans, northwestern Tibetans in Gangcha and Xunhua formed one clade, i.e., all absolute Z-scores of *f_4_(Gangcha, Xunhua Tibetan; Tibetan2, Mbuti)* were less than three (Supplementary Figure 13). Compared with Gannan Tibetans, Qinghai Tibetans had more ancestry sharing with Tibet Tibetans. We did not find Tibetan populations shared more alleles with Gannan Tibetans relative to other Tibetans, as all values in *f_4_(Tibetan1, Gannan Tibetan; Tibetan2, Mbuti)* were larger than zero. Southwestern Yunnan Tibetan formed one clade with Chamdo/Xinlong/Yajiang Tibetans, all of them belonged to Kham Tibetans (Supplementary Figures 14, 15). Lowland Sichuan/Yunnan Tibetans harbored increased Tibetan-related derived alleles compared with Gansu-Qinghai Tibetans and more ancestry related to highland Tibetans compared with other highland Tibetans.

We additionally explored genetic affinity and population substructure among highland and lowland Tibetans using ancient Eurasian populations via *f_4_(Modern Tibetan1, Modern Tibetan2; Ancient Eurasians, Mbuti)*. The non-significant Z-scores in *f_4_(Ü-Tsang Tibetans1, Ü-Tsang Tibetans2; Ancient Eurasians, Mbuti)* confirmed the genomic homogeneity within the four high-altitude Ü-Tsang Tibetans. We could also identify the more allele sharing between the Nepal ancients and Ü-Tsang Tibetans compared to Ando and Kham Tibetans (Supplementary Figures 16–19). Compared with Shannan Tibetan, Nagqu Tibetan harbored increased ancestry associated with the lowland ancient populations. Compared to Qinghai Ando Tibetans, Nagqu Tibetan possessed both increased Nepal ancients-related ancestry and increased Late Neolithic Lajia-related ancestry relative to Xunhua Tibetan. Nagqu Tibetan also harbored additionally increased ancestry related to the coastal Late Neolithic SEAs, middle Yellow River Middle-Neolithic to Iron Age ancient populations, Upper Xiajiadian culture-related Bronze Age populations, inland Neolithic NEAs and other upper Yellow River Late Neolithic and Iron Age populations. Significant negative-*f*_4_-values were observed in Ando Tibetans via *f_4_(modern Tibetan1, Gansu-Qinghai Ando Tibetans; Bronze Age stepped pastoralists, Mbuti)*, which suggested that Ando Tibetans harbored increased ancestry related to steppe pastoralists, such as Sintashta, Yamnaya, Afanasievo, Srubnaya, Andronovo and Xinjiang Iron Age Shirenzigou populations. Although strong genetic affinity within Ando Tibetans was confirmed with the similar patterns of *f*_4_-based sharing alleles and non-significant statistical results in symmetry-*f*_4_ statistics. Statistically significant negative *f*_4_-values in *f_4_(Gangcha Tibetan, Gannan Tibetan; Ami/Atayal/Hanben/Gongguan/Tanshishan_LN/Qihe_EN, Mbuti)* showed that Gannan Tibetan harbored increased SEA ancestry related to modern Austronesian or Proto-Austronesian-related Neolithic to present-day southeastern coastal/island populations (Supplementary Figures 20–22). A similar SEA affinity of Gannan Tibetan was also identified compared with Tibet Ü-Tsang Tibetans. Results of the four-population comparison analysis focused on Kham Tibetans are presented in Supplementary Figures 23–25, which suggested that Kham Tibetans had increased both northern and SEA ancestry.

### Spatiotemporal Comparison Analysis Among Modern Tibetans and All Paleolithic-to-Historic East Asians Showed the Genetic Admixture and Continuity of Modern Tibetans

We nest used *f*_4_-statistics to elucidate the patterns of genomic structure and population dynamic of East Asians and provide new insights into the origin of culturally/geographically diverse Tibetans. Focused on four early coastal Neolithic NEAs from Shandong province, *f_4_(coastal Neolithic NEA1, coastal Neolithic NEA2; Modern Tibetans/Ancient East Asians, Mbuti)* revealed the similar genetic relationship between modern Tibetans and these different Neolithic NEAs (Supplementary Figure 26). Results from *f_4_(Bronze/Iron Age Henan populations, Neolithic-to-Iron-Age Henan populations; Eastern Modern Tibetan/Ancient East Asians, Mbuti)* only revealed Luoheguxiang people had increased ancestry associated with modern Austronesian-speaking Ami (Supplementary Figures 27–29) relative to Wanggou_MN. The Late Neolithic Haojiatai population had more SEA-like ancestry related to Xitoucun_LN and Iron Age Hanben people compared with Wanggou_MN (Supplementary Figure 30). The genetic affinity with southern coastal populations (Ami/Atayal/Hanben-related) was also observed in Pingliangtai_LN, but not in Wadian_LN and Middle Neolithic Wanggou_MN and Xiaowu_EN (Supplementary Figures 31–34). Focused on ancients from Shaanxi and Inner Mongolia, we found that modern Tibetans and northern and southern EAs from the Yellow River and south China shared more alleles with Late Neolithic Shimao populations (Supplementary Figure 35). Temporal analysis among upper Yellow River ancients showed all modern Tibetans showed a similar relationship with them, although Iron Age Dacaozi people harbored more SEA ancestry. These results suggested that population movements from southern China have a significant influence on the gene pool formation of northeastern populations on the TP at least from the Iron Age (Supplementary Figure 36). Symmetrical relationships among East Asians with temporally different Nepal ancient populations were shown in Supplementary Figure 37.

**FIGURE 4:**
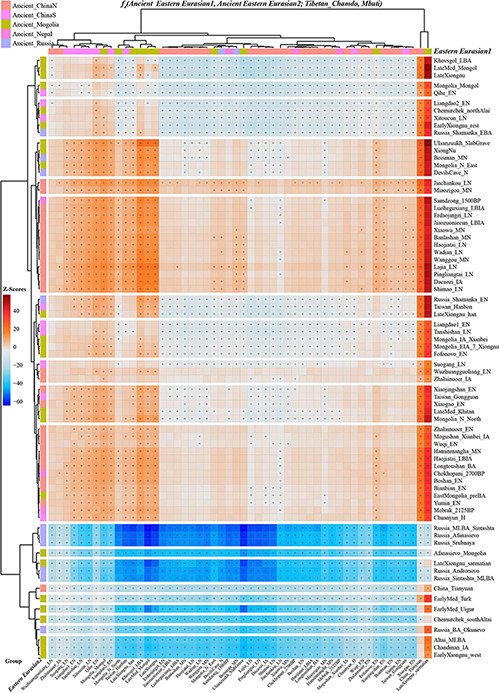
The genomic affinity between Chamdo Tibetans and other eastern Eurasian ancient populations inferred from four population affinity-f_4_ statistics of the form *f*_4_(Ancient Eastern Eurasian1, Ancient Eastern Eurasian; Tibetan_Chamdo, Mbuti). Red color with statistically significant f_4_-values (marked with “+”) demoted Chamdo Tibetans shared more derived alleles with Ancient Eastern Eurasian1 (right population lists) compared with Ancient Eastern Eurasian2 (bottom population lists). Blue color with significant f_4_-values denoted Chamdo Tibetans shared more Ancient Eastern Eurasian1-related derived alleles relative to their counterpart.

Next, we also explored the similarities and differences of the shared genetic profiles related to northern Neolithic East Asians via the spatial comparison analysis with modern Tibetans and all available ancient East Asians as reference. We conducted a series of symmetry *f*_4_-statistics to compare all eleven modern Tibetan populations and other ancient East Asians against the geographically different ancient NEAs and ancient Tibetans. Figure 4 and Supplementary Figures 38–41 showed the shared alleles between the targeted populations and the lowland early Neolithic NEAs and others. The *f_4_(NEAs, Chokhopani; Modern Tibetan/Neolithic-to-historic East Asians, Mbuti)* was used to determine the lowland and highland East Asian affinity. Compared with four coastal Neolithic Shandong populations, we found that Ü-Tsang Tibetans had a strong highland East Asian affinity. Besides, comparison against the coastal and inland ancients revealed that modern Tibetans had a strong inland-NEA-affinity, especially with Late Neolithic Lajia people from the upper Yellow River. This Lajia-affinity or inland-NEA-affinity persisted when we substituted inland Yumin_MN with the coastal Neolithic NEAs (Supplementary Figure 42), but disappeared when we substituted the latter Neolithic groups with the early Neolithic NEAs (Supplementary Figures 43–48). We summarized the overall highland/lowland East Asian affinities of Tibetans in Supplementary Figure 49, which showed the Ando and Kham Tibetans had lowland NEA affinity, and Ü-Tsang Tibetans possessed additional Nepal ancient affinity.

Our genomic studies have identified population substructures within modern Tibetans. Modern Tibetans can be classified into three subgroups by their different affinities with NEAs, SEAs and Siberians, which were confirmed by the negative values in *f_4_(Reference populations, modern Tibetans; northern/southern EAs and Siberians, Mbuti)*. We further tested if one single source could explain the observed genetic variations in Tibetans. We first assumed that modern Tibetans were the direct descendants of SEAs which is associated with the Yangtze Rice farmers. As shown in Supplementary Figures 50–58, we observed significant negative *f*_4_-values in *f_4_(SEAs, modern Tibetans; Reference populations, Mbuti)* when we used NEAs/Siberians as the reference populations, which indicated obvious gene flow events from these reference populations into modern Tibetans. We then assumed that Tibetans’ direct ancestor was coastal Neolithic-NEAs, we conducted *f_4_(Shandong ancients, modern Tibetans; Neolithic-to-historic East Asians, Mbuti)* and found only Nepal ancients showed the negative-*f*_4_-values, which was consistent with the common origin of the Sino-Tibetan speakers from YRB (Supplementary Figures 59–62). The patterns were confirmed when we assumed Yangshao and Longshan farmers or their related populations (Supplementary Figures 63–71), Shaanxi ancients (Supplementary Figures 72–74), and other ancient NEAs and southern Siberians (Supplementary Figures 75–88) as the direct ancestor of modern Tibetans. As shown in Supplementary Figures 75–88, when assuming Yumin or Ulchi as the direct ancestor of Tibetans, we identified additional gene flows from the SEAs (Hanben and Tanshishan et al.) and Yellow River farmers into Tibetans. Assuming the Nepal ancients as direct ancestors, we detected obvious additional gene flow from the lowland ancient East Asians to Kham Tibetans (Supplementary Figures 89–91). Additional predefined ancestral populations from Russia and Chinese Xinjiang further confirmed the strong northern East Asian affinity (Supplementary Figures 92–104). Thus, *f*_4_-statistics showed that the formation of modern Tibetans had involved multiple admixture events.

### Ancestry Compositions of Ancient/Modern Tibetans via *qpWave/qpAdm* and *qpGraph*

**FIGURE 5:**
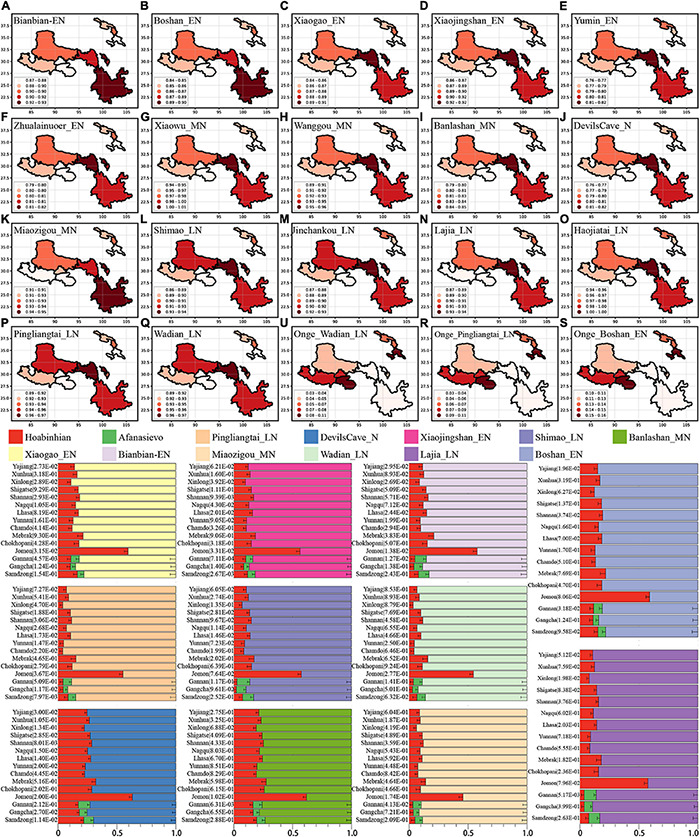
Results of qpAdm showed the main ancestry composition of ancient/modern Tibetans and Jomon Hunter-Gatherer were the results of the mixing of ancient NEA and one deep lineage associated with South Asian Hunter-Gatherer Onge or Southeast Hunter-Gatherer Hoabinhian (the early Asian). Heatmap showed the NEA-related ancestry in the two-way admixture model of Onge and the early Neolithic East Asian **(A–F)**, Middle-Neolithic NEA **(G–K)**, and Late-Neolithic NEA **(L–Q)**. Onge-related ancestry was presented with three cases **(R,S,U)**. Bar plots showed the ancestry composition of the two-way model of Hoabinhian and East Asian for modern Tibetan, Jomon and Ancient Nepal Mebrak and Samdzong people, and three-way model for Qinghai and Gansu Tibetans.

From the autosomal perspective, we found the close connections of modern Tibetans and Neolithic NEAs. From a paternal Y chromosomal perspective, Tibetan shared a genetic affinity with Andamanese Onge and Jomon hunter-gatherers from the Japanese archipelago (Shi et al., 2008). Onge and Jomon were suggested to be an early Asian lineage with a close relationship with 7700-year-old Hoabinhian from southeast Asia (McColl et al., 2018). We further explored the number of ancestral populations of modern Tibetans, Nepal ancients and Jomon using the *qpWave* and estimated their corresponding ancestry proportions under one-way, two-day and three-way admixture models. The *qpWave* results (p_rank < 0.05) showed that at least two ancestral populations were needed to explain the observed genetic variations in targeted populations. We first employed the two-way model of Onge and six inland/coastal early Neolithic-NEAs and found inland Yumin failed to fit our targeted populations’ genetic variations (all p_values < 0.05). The two-way model “Xiaogao_EN-Onge” could fitted all modern Tibetans well except for Gannan Tibetan with the Xiaogao-related ancestry proportion ranging from 0.846 in Shannan Tibetan to 0.906 in Xinlong Tibetan. The 2700-year-old Chokhopani, like geographically close Shigatse Ü-Tsang Tibetans, could be fitted as an admixture of 0.861 NEA Xiaogao-related ancestry and 0.139 Onge-related ancestry (Supplementary Table 20 and Figure 5). Younger Nepal ancient could be modeled as major ancestry from Onge-related ancestry and minor ancestry associated with NEA lineage. Jomon could be modeled as deriving 0.484 of its ancestry from populations related to Xiaogao_EN and 0.516 from groups related to Onge with marginal statistical significance. We substituted Boshan_EN and Bianbian_EN with Xiaogao_EN, we could obtain similar results, however, when we substituted Xiaojingshan_EN with Boshan_EN, 1500-year-old Samdzong failed to fit our two-way model (p_rank1 = 0.00007). The “Zhalainuoer_EN-Onge” model could be successfully fitted highland Tibet Tibetans and Yunnan Tibetan with high Onge-related ancestry but failed to fit other Ando and Kham Tibetans. Using Middle-Neolithic East Asian as the source, the “Xiaowu_MN-Onge” model failed to all targets, and the “DevilsCave_N-Onge” model could only fit the Sichuan Tibetans, Jomon, and Chokhopani with a high proportion of Onge-related ancestry. Except for populations with a western Eurasian affinity (Ando Tibetans and Samdzong), all remaining ancient/modern populations could be fitted as the admixture between Onge and Middle Neolithic Wanggou_MN, Banlashan_MN, or Miaozigou_MN. We additionally substituted Onge with Hoabinhian as the southern source representative for deep lineage and used early Neolithic to Late-Neolithic NEAs as the other source to perform the two-way admixture model for estimating the ancestry proportion of modern Tibetan without Gangcha and Gannan Tibetans and Nepal ancients except for ancient Samdzong and Jomon. As shown in Figure 5, a good fit could be acquired with slightly variable ancestry composition compared with Onge-based two-way models. We finally employed the Afanasievo (significant negative-*f*_3_ value in admixture-*f*_3_-statistics) as the western Eurasian source in a three-way admixture model to fit the genetic variations in Ando Gangcha and Gannan Tibetans and Samdzong. All three populations could be successfully fitted when we introduced the Bronze Age steppe pastoralists’ related ancestry.

**FIGURE 6:**
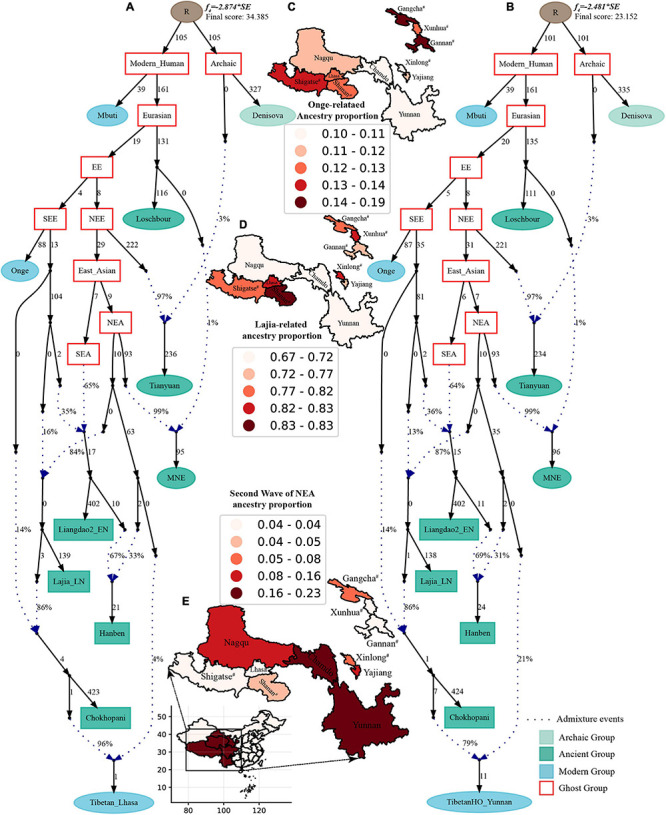
Admixture graph model of East Asians and modern Tibetans based on the Human Origin dataset. Admixture history of highland Tibetan from Lhasa **(A)** and lowland Tibetan from Yunnan **(B)**. Heatmap showed the ancestry composition of modern Tibetans from three source populations: deep hunter-gatherer One-related ancestry **(C)**, the first batch of Neolithic farmer-associated ancestry **(D)** and the second batch of Neolithic farmer related ancestry **(E)**. Denisovan and Central African of Mbuti were used as the Archaic and modern roots respectively. Western Eurasian was represented by Loschbour. Deep southern Eurasian (SEE) and northern Eurasian (NEE) were represented by South Asian Hunter-Gatherers of Onge and 40,000-year-old Tianyuan people. East Asian subsequently diverged as NEA (NEA) and SEA (SEA). All f_4_-statistics of included populations are predicted to within 3 standard errors of their observed values. Branch lengths are given in units of 1000 times the f_2_ drift distance (rounded to the nearest integer). Pound signs denoted the modern populations added to the basic model of A,B with larger Z-scores or Zero internal branch length. Blue dotted lines denoted admixture events with admixture proportions as shown.

**FIGURE 7:**
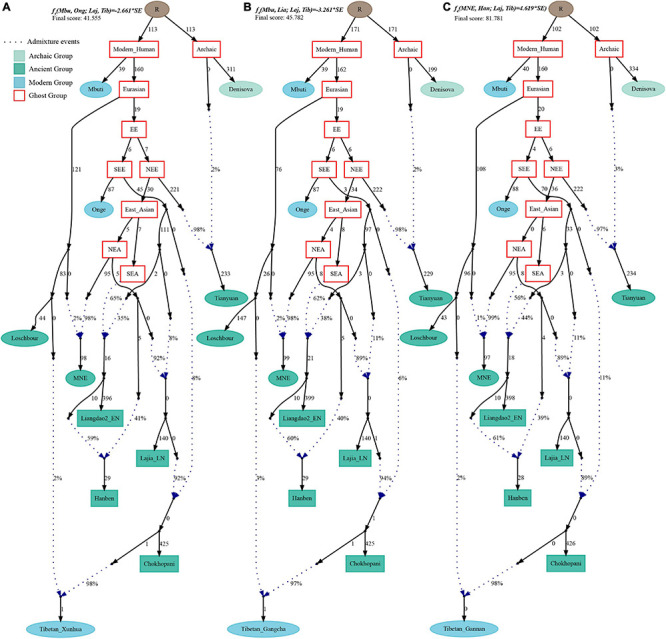
Admixture graph model of East Asians and modern Tibetans from the northeast Tibetan Plateau based on the Human Origin dataset. Admixture history of Tibetan from Xunhua **(A)**, Tibetan from Gangcha **(B)**, and Tibetan from Gannan **(C)**.

**FIGURE 8:**
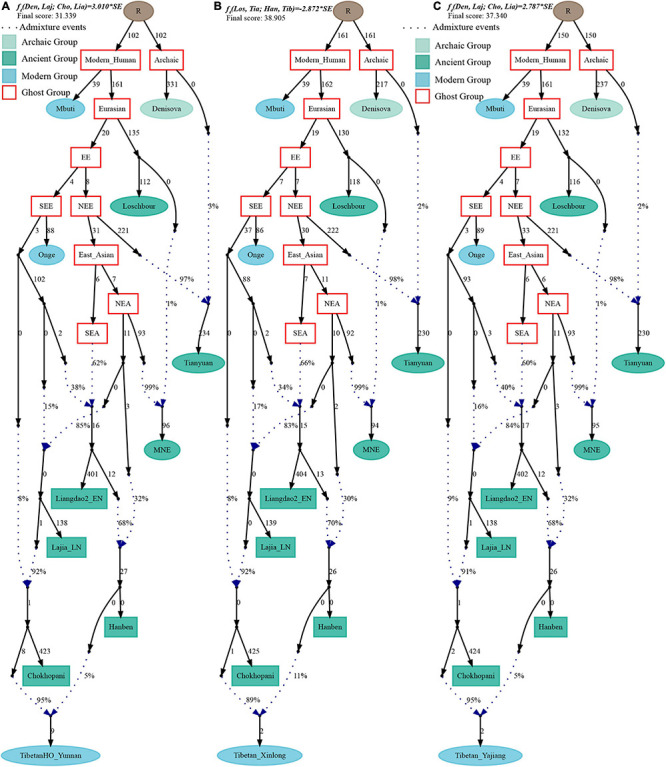
Admixture graph model of East Asians and modern lowland Tibetans based on the Human Origin dataset. Admixture history of lowland Tibetan from Yunnan **(A)**, Tibetan from Xinlong **(B)**, and Tibetan from Yajiang **(C)**.

**FIGURE 9:**
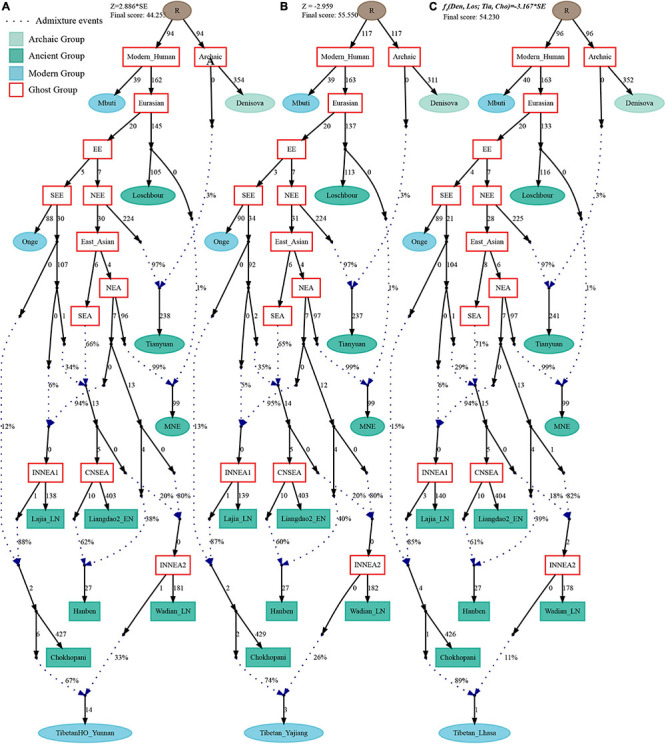
Admixture graph model of modern highland and lowland Tibetans based on the Human Origin dataset using Late-Neolithic Wadian people as the source of the second migration into Tibetan Plateau. Admixture history of lowland Tibetan from Yunnan **(A)**, Tibetan from Yajiang **(B)**, and highland Tibetan from Lhasa **(C)**.

Finally, to comprehensively summarize the phylogenetic relationships and reconstruct the population history between Neolithic East Asians and modern Tibetans in one phylogenetic framework, we built a series of admixture graph models via *qpGraph*. The core model of our admixture graph included archaic Denisovan and central African Mbuti as the roots, Loschbour as the representative of western Eurasian, modern Onge hunter-gatherer from Andaman island and 40,000-year-old Tianyuan (3% ancestry from Denisovan) as representatives of deep lineages of southern East Eurasian and northern East Eurasian. As shown in Figure 6A, East Asians diverged into northern lineage (represented by East Mongolia Neolithic population with 1% gene flow from western Eurasian) and southern lineage (represented by Liangdao2_EN with 35% ancestry deriving from lineages close to Onge). Here, Late Neolithic Qijia-related Lajia people could be fitted as an admixture of 84% from a lineage related to NEAs and 16% from a lineage associated with Andamanese Onge. Ancient Chokhopani in Nepal could be modeled as driving 86% of the ancestry from Lajia_LN and 14% from the Onge side. Our model provided ancient genomic evidence of the co-existence of both Paleolithic hunter-gatherer ancestry associated with the indigenous TP people and Neolithic NEA ancestry in Chokhopani culture-related ancient Tibetans and Late Neolithic Lajia people. We subsequently added all eleven modern Tibetan populations to this scaffold model and found all Ü-Tsang and Kham Tibetans except for Xinlong Tibetan could be fitted as direct descendants from Chokhopani with additional gene flow from one NEA related population, which also contributed additional 33% ancestry to Iron Age Hanben people. This gene flow could be regarded as the epitome of the second wave of Neolithic expansion into TP. Thus, results from Figure 6 suggested that seven Tibetans could be well fitted with three sources of ancestry: Onge-related, Lajia_LN-related and second wave of NEA lineage-related, in respective proportion of 0.1235, 0.8265, and 0.0500 (Shannan); 0.1440, 0.8160, and 0.0400 (Shigatse); 0.1344, 0.8256, and 0.0400 (Lhasa), 0.1176, 0.7224, and 0.1600 (Nagqu); 0.1001, 0.6699, and 0.2300 (Chamdo); 0.1106, 0.6794, and 0.2100 (Yunnan); 0.1232, 0.7568, and 0.1200 (Yajiang). We could obtain a good fit when considering one gene flow event for Gansu-Qinghai Ando Tibetans with the Loschbour-related ancestry proportion varying from 2 to 3% (Figure 7). To further explore the best ancestral source proximity of the second migration wave, extended admixture graphs introducing inland/coastal northern and SEA Neolithic populations were reconstructed. As shown in Figure 8, the second wave into lowland Kham Tibetans with Neolithic SEA affinity could be well fitted as directly deriving from Hanben-related ancestral population with the proportion ranging from 5 to 11%. We then added northern coastal early Neolithic Houli Boshan people, Middle Neolithic Xiaowu Yangshao people, Late Neolithic Wadian people, and Bronze to Iron Age Haojiatai Shangzhou people to our core model in Figure 6 and then fitted all Tibetans on it. We found that Yunnan Kham Tibetan harbored 33% additional ancestry associated with Longshan people, and Sichuan Yajiang Kham Tibetan with 26% additional Longshan-related ancestry (Figure 9). It was interesting to find that the gene pool of the Lhasa Ü-Tsang Tibetan was also influenced by the second population migration associated with the Longshan people. This second gene flow event persisted when we substituted Longshan people with other Neolithic or Bronze to Iron Age populations with acceptable ancestry proportions (Supplementary Figures 105–107). These phenomena may be caused by the genetic stability of the main ancestry in the Central Plain (Henan and Shandong provinces).

**FIGURE 10:**
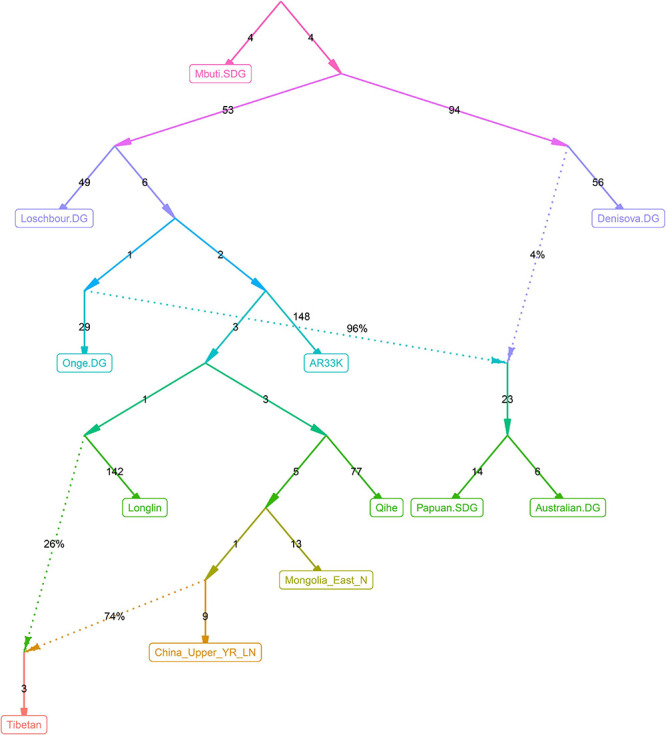
qpGraph-based admixture models showed the differentiated Denisovan admixture landscape between East Asians and Oceanians.

Recent genetic studies have evidenced that Denisovan-like haplotypes have contributed to the high-altitude adaptation of modern Tibetans (Huerta-Sánchez et al., 2014). Morphologic evidence from Xiahe people and mitochondrial DNA from Baishiya Karst Cave’s ancient remains further suggested that archaic people related to Denisovan had arrived at TP during the Pleistocene (Chen F. et al., 2019; Zhang et al., 2020). However, our best-fitted *qpGraph*-based phylogenetic frameworks (Figures 6–9) did not show the expected genetic contribution from archaic hominid into modern Tibetans, probably due to the limited number of the available SNPs and the relatively low proportion of Denisovan ancestry in East Asians. Ancient genomes from ∼34,000-year-old Mongolia Salkhit and 40,000-year-old Tianyuan people have been evidenced for carrying genomic segments of Denisovan ancestry (Massilani et al., 2020). We also identified the archaic admixture into Tianyuan-related people in our reconstructed models. Next, we conducted the *f*_4_-statistics in the form of *f*_4_(modern and ancient East Asians, Tibetans; Denisovan, Chimpanzee) and did not identify significant *f*_4_-values, which suggested that highland and lowland East Asians formed a clade relative to the Denisovan and both harbored equal levels of Denisovan related ancestry. Similar patterns of archaic admixture between Neolithic East Asians and modern East Asians were also evidenced in a recent study on ancient DNA (Yang et al., 2020). We further tried to explore the highly differentiated 5-SNP EPAS1 haplotype motif (AGGAA) in our studied Tibetan populations, and we found that these five SNP loci were not included in the array we used. To further explore the possible bias caused by the lower SNP density in the HO dataset, we reconstructed a new *qpGraph* model based on the 1240K dataset focusing on both East Asians and Oceanians since Australians and Papuans have been suggested to possess a higher proportion of Denisovan related ancestry (Browning et al., 2018). We successfully identified an additional Denisovan-related gene flow into modern Oceanian populations (4%), but the obtained best-fitted model also did not include the genetic contribution from archaic people into modern Tibetans (Figure 10). Our *qpGraph*-based phylogeny showed that Tibetan was modeled as an admixture of 74% ancestry from the upper Yellow River farmers and 26% from Guangxi pre-Neolithic Longlin people. We should note that the obvious archaic gene flow into modern Tibetans had been documented in the proposed two-wave model of ‘Admixture of Admixture’ based on the phased haplotype via Lu’s whole-genome sequenced study (Lu et al., 2016). Besides, Browning et al. (2018) also documented two different pluses of archaic Denisovan admixture into East Asians. Thus, our reconstructed phylogenetic modeling graph without Denisovan archaic gene flow into modern Tibetans may be caused by the low admixture introgression levels at whole-genome scale, or by the enrichment of archaic genes in just certain specific regions, such as the EPAS1. The actual genetic interaction and introgression between lowland/highland anatomically and behaviorally modern humans and archaic people may be more complex. More powerful statistical computational methods and long-read sequencing data may provide new insights into the archaic admixture landscape of ancestral Tibetan populations. Thus, deep-whole-genome sequencing of modern and ancient highland East Asians needs to be conducted to further explore, simulate and validate the complete landscape of Denisovan gene diversity in modern Tibetans.

## Discussion

Prehistoric human activities and the origin of the high-altitude Tibetans are the research topic in a variety of disciplines, mainly including genetics, archeology, anthropology, history and literature. Recent genome-wide sequencing and paleo-genomic researches have revolutionized our knowledge of the peopling history of Europe (Olalde et al., 2018), Central/South Asia (Narasimhan et al., 2019), America (Nakatsuka et al., 2020), Africa (Skoglund et al., 2017), and Oceania (Lipson et al., 2018b). More and more ancient genomes from the surrounding regions of East Asia have been reported to explore the population dynamics in Southeast Asia (Lipson et al., 2018a; McColl et al., 2018) and South Siberia or Eurasia’s Eastern Steppe (Lazaridis et al., 2014; Raghavan et al., 2014; Mathieson et al., 2015; Damgaard et al., 2018; Sikora et al., 2019), but the ancient genomes in China are still lacking. Fortunately, eight ancient DNA studies from China have been conducted to characterize the deep population history of East Asians. Yang et al. (2017) sequenced 40,000-year-old Tianyuan individual from Beijing and found that the early Asian population substructures have existed before the divergence between East Asians and Native Americans and the peopling of America by anatomically modern human populations. Late Pleistocene and Holocene genomes from the Amur River reported by Mao et al. (2021) demonstrated the genetic transformation among the paleolithic population and their genetic stability in the Neolithic period. Yang et al. (2020) recently conducted another ancient DNA work focused on 24 ancient genomes from Neolithic northern East Asia (eight samples), Neolithic southern East Asia (fifteen samples), and one historic Chuanyun sample, and they found the north-south genetic differentiation among East Asians persisted since the early Neolithic period due to the observed significant genetic differences between Neolithic Shandong and Fujian samples (Yang et al., 2020). Besides, they also identified southward migrations from Shandong Houli populations and northward migrations from Fujian Tanshishan populations, as well as a Neolithic coastal connection from southeastern Vietnam to Russia Far East, and a Proto-Austronesian connection between SEAs and southeast Pacific Vanuatu islanders. The 11,000-year population dynamic documented in Guangxi province showed the extensive admixture between Guangxi, Fujian and Vietnam ancients, which contributed to the formation of pre-agriculture populations (Baojianshan and Dushan) and the affinity between historic Guangxi people and modern Tai-Kadai and Hmong-Mien people (Wang T. et al., 2021). Ning et al. (2020) reported the population history of northern China using fifteen ancient genomes from the Yellow River, West Liao River, and Amur River and discovered that the subsistence strategy changes were associated with the population movement and admixture. Ning and Wang et al. also reported the genomes of ten Iron Age Shirenzigou samples and found the Yamnaya-related steppe pastoralists mediated the population communications between East Asia and western Eurasia, and probably dispersed Indo-European language into Northwest China (Wang et al., 2020). Although these signs of progress have been achieved, the population history, genetic relationship, and genetic differentiation between the highland and lowland modern/ancient East Asians kept in their infancy and remained to be clarified. Thus, we collected nineteen TP-related Neolithic to historic ancients, seventy-eight modern Tibetans from Ü-Tsang, Ando and Kham Tibetan regions, as well as all available eastern Eurasian ancients with different prehistoric human cultural backgrounds as well as modern Eurasians from Indo-European, Altaic, Uralic, Sino-Tibetan, Austronesian, Austroasiatic, Hmong-Mien and Tai-Kadai language families and conducted one comprehensive Paleolithic to present-day ancient/modern genomic meta-analysis. We provided new insights into the peopling of TP and clarify the relationships between high-altitude and lowland ancient/modern East Asians.

There are three hypotheses proposed to elucidate the origin of the Sino-Tibetan language family based on linguistic diversity and others (Zhang et al., 2019). The three hypotheses are North China origin associated with Yangshao/Majiayao hypothesis, Southwest Sichuan origin hypothesis, and Northeast India origin hypothesis. Ancient/modern genomes from the TP showed a clear connection with the northern modern Han Chinese and Neolithic-NEAs, especially with the coastal Houli people from Shandong, inland Yangshao and Longshan people from Henan, and Qijia people from Ganqing region, which supported the northern China origin of modern Tibeto-Burman-speaking populations. Shared ancestry revealed by our PCA, pairwise *F*_*ST*_ and outgroup-*f*_3_-values, ADMIXTURE, and *f*_4_-statistics among ancient/modern highlanders and NEA lowlanders showed their close relationship, which was consistent with genetic similarities revealed by the forensic low-density genetic markers and uniparental haplotype/haplogroup data (Zou et al., 2018; Chen P. et al., 2019; He et al., 2019). Direct evidence supported and confirmed this proposed common origin of the Sino-Tibetan (North China origin hypothesis) that was provided by the phylogenetic relationship reconstruction. Both *TreeMix*- and *qpGraph*-based phylogenetic framework supported that the main ancestry in modern Tibetans and ancient TP samples (Nepal and Qijia ancients) was derived from the common NEA lineage related to East Mongolia Neolithic people and Yangshao/Longshan/Houli people from the Central Plain in northern China. Thus, our results in this meta-genomic analysis supported the main lineage that contributed to TP people originated from the Upper and Middle Yellow River with the Neolithic expansion of millet farmers. Our analysis confirmed the origin, diversification, and expansion of the modern Sino-Tibetan populations revealed by the mitochondrial and Y-chromosome variations (Wang L. X. et al., 2018; Li et al., 2019).

Although strong evidence for the common origin of Sino-Tibetan speakers was provided, we still identified the differences in their ancestry composition. Compared with the highlanders on the TP, lowland Late Neolithic to present-day East Asians harbored more ancestry related to Neolithic SEAs and Siberians. Iron Age Dacaozi people from the Gansu-Qinghai region also showed a close genetic affinity with southern people from Tanshishan culture, which indicated the northward dispersal of rice farmers. Compared with the lowland Yangshao/Longshan or coastal Houli populations, the highland populations harbored a certain (8∼14%) proportion of Paleolithic hunter-gatherer ancestry related to the early diverged deep eastern Eurasian lineages (Onge or Hoabinhian related lineages). Lu et al. (2016) illuminated the co-existence of Paleolithic and Neolithic ancestry in modern Tibetans based on the shared haplotypes. Here, we further evidenced the Neolithic and Pre-Neolithic ancestries co-existed in highland East Asians using the allele frequency spectrum in the *f*-statistics (especially for the admixture models of *qpAdm* and *qpGraph*). Thus, our meta-analysis provided new robust evidence for the co-existence of both Paleolithic and Neolithic ancestries in the gene pool of East Asian highlanders as well as the Paleolithic colonization and Neolithic expansion of TP people, which was previously clarified via the modern whole genomes, mitochondrial and Y-chromosomal data (Qi et al., 2013; Wang L. X. et al., 2018; Li et al., 2019).

Additionally, we also found obvious population substructures among modern Tibetans: Ü-Tsang Tibetans in Tibet core region had predominant original Paleolithic and Neolithic ancestries; Ando Tibetans from Gansu-Qinghai region in northwest China had 2∼3% western Eurasian related ancestry via *qpGraph*-based model; Kham Tibetans from Sichuan and Yunnan provinces possessed a strong southern Neolithic East Asian affinity. Thus, population substructures observed in modern Tibetans were consistent with the geographic and cultural divisions, which suggested that the complex cultural background and terrain to some extent served as the barriers for population movement and admixture. Our *qpGraph*-based phylogeny revealed the gene flow from southern Iron-Age East Asians into Kham Tibetans, from Neolithic NEAs into Kham and Ü-Tsang Tibetans, from western Eurasians into Ando Tibetans, which demonstrated multiple waves of migrant influx from the Siberia, northern and southern East Asia had shaped the gene pool of Tibetan highlanders.

## Conclusion

We performed a comprehensive genomic meta-analysis focused on Neolithic to present-day people to clarify the relationship between the TP highlanders and lowland East Asians and to explore the peopling of TP. We found a strong genetic affinity between Tibetans and Neolithic to present-day NEAs, which suggested Tibeto-Burman speakers originated from the Upper and Middle YRB in northern China. The observation of the shared ancestry between Han Chinese and Tibetans was consistent with the co-dispersal of millet farmers and Sino-Tibetan languages. Although the shared ancestry persisted between ancient Tibetans and lowland Neolithic people (Yangshao/Longshan/Houli culture), we also found genetic differentiation between them: highland Tibetans harbored more deeply diverged eastern Eurasian Onge-related hunter-gatherer ancestry, but the lowland Neolithic to present-day NEAs possessed more ancestry related to the Neolithic SEAs and Siberians, which not only suggested the co-existence of Paleolithic and Neolithic ancestries in ancient/modern Tibetans but also illuminated the population history of Paleolithic colonization and Neolithic expansion. Besides, consistent with the geographic/linguistic divisions, we identified population substructures in modern Tibetans: more Onge/Hoabinhian related ancestry in Ü-Tsang Tibetans, much more western Eurasian related ancestry in Ando Tibetans, and more Neolithic SEA related ancestry in Kham Tibetan. In short, modern East Asian highlanders derived their ancestry from at least five waves of population admixture: Hoabinhian as the oldest Paleolithic layer; additional gene flow from two Neolithic expansions (inland and coastal) from NEAs, one Neolithic SEA northwestward expansion and one western Eurasian eastward expansion.

## Significance Statement

The Tibetan Plateau has a harsh and extreme high-altitude hypoxic environment, which is inhospitable for human permanent settlement. The population genomic history of modern Tibetans and the population dynamic demographic history of their predecessors need to be comprehensively characterized. We used one large-scale modern and ancient Eurasian meta-dataset to perform genomic analyses focusing on the fine-scale population structure of modern and ancient East Asian highlanders. Firstly, we identified the genomic affinity between highlanders and Neolithic-to-modern Northern East Asians, which was in accordance with the archeologically documented phenomena of Neolithic millet farmer expansion from the Yellow River Basin with the dissemination of Tibeto-Burman languages. Secondly, we identified the obvious population substructure in modern Tibetans along with their cultural division. Thirdly, we documented multiple waves of peopling the Tibetan Plateau and the complex admixture history of East Asian highlanders via the *qpGraph*-based phylogenetic frameworks.

## Data Availability Statement

The datasets presented in this study can be found in online repositories. The names of the repository/repositories and accession number(s) can be found in the article/ Supplementary Material.

## Ethics Statement

The studies involving human participants were reviewed and approved by this project was inspected and approved by the Medical Ethics Committee of the Xiamen University. The patients/participants provided their written informed consent to participate in this study. Written informed consent was obtained from the individual(s) for the publication of any potentially identifiable images or data included in this article.

## Author Contributions

GH, RT, C-CW, H-YY, and MW conceived the idea for the study. GH, MW, XZ, PC, ZW, YL, HY, and L-HW performed or supervised wet laboratory work and analyzed the data. GH, MW, and XZ wrote and edited the manuscript. All authors contributed to the article and approved the submitted version.

## Conflict of Interest

The authors declare that the research was conducted in the absence of any commercial or financial relationships that could be construed as a potential conflict of interest. The reviewer JH declared a past co-authorship with the authors GH, XZ, C-CW, L-HW, H-YY to the handling editor.

## Publisher’s Note

All claims expressed in this article are solely those of the authors and do not necessarily represent those of their affiliated organizations, or those of the publisher, the editors and the reviewers. Any product that may be evaluated in this article, or claim that may be made by its manufacturer, is not guaranteed or endorsed by the publisher.
