# Supplementary material for: Peopling History of the Tibetan Plateau and Multiple Waves of Admixture of Tibetans Inferred From Both Ancient and Modern Genome-Wide Data
Source: Front Genet. 2021 Sep 3;12:725243. doi: 10.3389/fgene.2021.725243 (PMC8506211; doi:10.3389/fgene.2021.725243)
Supplement: Supplementary file 2 [file Data_Sheet_2.PDF]

Supplementary Figures S21-S49

Guanglin He[1,2,*,#], Mengge Wang[3,*], Xing Zou[3,*], Pengyu Chen[4], Zheng Wang[2], Yan Liu[5], Hongbin Yao[6], Lan-Hai Wei[2], Renkuan Tang[7,#], Chuan-Chao Wang[2,#], Hui-Yuan Yeh[1,#]

[1]School of Humanities, Nanyang Technological University, Nanyang, 639798, Singapore
[2]Department of Anthropology and Ethnology, Institute of Anthropology, National Institute for Data Science in Health and Medicine, and School of Life Sciences, Xiamen University, Xiamen, 361005, China
[3]Institute of Forensic Medicine, West China School of Basic Science and Forensic Medicine, Sichuan University, Chengdu, 610041, China
[4]Center of Forensic Expertise, Affiliated hospital of Zunyi Medical University, Zunyi, Guizhou, 563099, China
[5]School of Basic Medical Sciences, North Sichuan Medical College, Nanchong, Sichuan, 637100, China
[6]Key Laboratory of Evidence Science of Gansu Province, Gansu Institute of Political Science and Law, Lanzhou, 730070, China
[7]Department of Forensic Medicine, College of Basic Medicine, Chongqing Medical University, Chongqing, 400016, China
*These authors contributed equally to this work and should be considered co-first authors.
[#]Corresponding author
Guanglin He
Affiliation: School of Humanities, Nanyang Technological University, Nanyang, 639798, Singapore; Department of Anthropology and Ethnology, Institute of Anthropology, National Institute for Data Science in Health and Medicine, Xiamen University, Xiamen, China.
E-mail: Guanglinhescu@163.com
Renkuan Tang
Department of Forensic Medicine, College of Basic Medicine, Chongqing Medical University, Chongqing, China
E-mail: renktang2012@163.com
Chuan-Chao Wang
Affiliation: Department of Anthropology and Ethnology, Institute of Anthropology, National Institute for Data Science in Health and Medicine, Xiamen University, Xiamen, China.
E-mail: wang@xmu.edu.cn
Hui-Yuan Yeh
Affiliation: School of Humanities, Nanyang Technological University, Nanyang, 639798, Singapore
E-mail: hyyeh@ntu.edu.sg

Contents

Figure S21. Genomic affinity between modern Tibetans and eastern Eurasian ancient populations inferred from four population symmetry-f_4 statistics of the form *f_4(Tibetan1, Xunhua Tibetan; eastern Eurasian ancients, Mbuti)*. 1

Figure S22. Genomic affinity between modern Tibetans and eastern Eurasian ancient populations inferred from four population symmetry-f_4 statistics of the form *f_4(Tibetan1, Gannan Tibetan; eastern Eurasian ancients, Mbuti)*. 3

Figure S23. Genomic affinity between modern Tibetans and eastern Eurasian ancient populations inferred from four population symmetry-f_4 statistics of the form *f_4(Tibetan1, Xinlong Tibetan; eastern Eurasian ancients, Mbuti)*. 4

Figure S24. Genomic affinity between modern Tibetans and eastern Eurasian ancient populations inferred from four population symmetry-f_4 statistics of the form *f_4(Tibetan1, Yajiang Tibetan; eastern Eurasian ancients, Mbuti)*. 5

Figure S25. Genomic affinity between modern Tibetans and eastern Eurasian ancient populations inferred from four population symmetry-f_4 statistics of the form *f_4(Tibetan1, Yunnan Tibetan; eastern Eurasian ancients, Mbuti)*. 7

Spatiotemporal comparison analysis among modern Tibetan and all Paleolithic-to-historic East Asians showed the genetic admixture and continuity of modern Tibetans 9

Figure S26. Shared ancestry associated with coastal Neolithic northern East Asian in modern Tibetans and ancient East Asians compared with geographically different coastal Neolithic northern East Asian inferred from four population symmetry-f_4-statistics of the form *f_4(Coastal Neolithic northern East Asian1, Coastal Neolithic northern East Asian2; Eastern Modern Tibetan/Ancient East Asians, Mbuti)*. 9

Figure S27. Shared ancestry associated with inland Neolithic to Iron Age northern East Asian from Henan province in modern Tibetans and ancient East Asians compared with geographically different ancient Henan populations inferred from four population symmetry-f_4-statistics of the form *f_4(Haojiatai_LBIA, Neolithic to Iron Age Henan populations; Eastern Modern Tibetan/Ancient East Asians, Mbuti)*. 11

Figure S28. Shared ancestry associated with inland Neolithic to Iron Age northern East Asian from Henan province in modern Tibetans and ancient East Asians compared with geographically different ancient Henan populations inferred from four population symmetry-f_4-statistics of the form *f_4(Jiaozuoniecun_LBIA, Neolithic to Iron Age Henan populations; Eastern Modern Tibetan/Ancient East Asians, Mbuti)*. 12

Figure S29. Shared ancestry associated with inland Neolithic to Iron Age northern East Asian from Henan province in modern Tibetans and ancient East Asians compared with geographically different ancient Henan populations inferred from four population symmetry-f_4-statistics of the form *f_4(Luoheguxiang_LBIA, Neolithic to Iron Age Henan populations; Eastern Modern Tibetan/Ancient East Asians, Mbuti)*. 13

Figure S30. Shared ancestry associated with inland Neolithic to Iron Age northern East Asian from Henan province in modern Tibetans and ancient East Asians compared with geographically different ancient Henan populations inferred from four population symmetry-f_4-statistics of the form *f_4(Haojiatai_LN, Neolithic to Iron Age Henan populations; Eastern Modern Tibetan/Ancient East Asians, Mbuti)*. 14

Figure S31. Shared ancestry associated with inland Neolithic to Iron Age northern East Asian from Henan province in modern Tibetans and ancient East Asians compared with geographically different

ancient Henan populations inferred from four population symmetry-f_4-statistics of the form *f_4(Pingliangtai_LN, Neolithic to Iron Age Henan populations; Eastern Modern Tibetan/Ancient East Asians, Mbuti)*. 16
Figure S32. Shared ancestry associated with inland Neolithic to Iron Age northern East Asian from Henan province in modern Tibetans and ancient East Asians compared with geographically different ancient Henan populations inferred from four population symmetry-f_4-statistics of the form *f_4(Wadian_LN, Neolithic to Iron Age Henan populations; Eastern Modern Tibetan/Ancient East Asians, Mbuti)*. 17
Figure S33. Shared ancestry associated with inland Neolithic to Iron Age northern East Asian from Henan province in modern Tibetans and ancient East Asians compared with geographically different ancient Henan populations inferred from four population symmetry-f_4-statistics of the form *f_4(Wanggou_MN, Neolithic to Iron Age Henan populations; Eastern Modern Tibetan/Ancient East Asians, Mbuti)*. 18
Figure S34. Shared ancestry associated with inland Neolithic to Iron Age northern East Asian from Henan province in modern Tibetans and ancient East Asians compared with geographically different ancient Henan populations inferred from four population symmetry-f_4-statistics of the form *f_4(Xiaowu_MN, Neolithic to Iron Age Henan populations; Eastern Modern Tibetan/Ancient East Asians, Mbuti)*. 19
Figure S35. Shared ancestry associated with inland Neolithic northern East Asian from Shaanxi and Inner Mongolia provinces in modern Tibetans and ancient East Asians compared with geographically different ancient populations inferred from four population symmetry-f_4-statistics of the form *f_4(Inland Neolithic northern East Asian1, Inland Neolithic northern East Asian2; Eastern Modern Tibetan/Ancient East Asians, Mbuti)*. 20
Figure S36. Shared ancestry associated with inland Neolithic northern East Asian from Qinghai province in modern Tibetans and ancient East Asians compared with geographically different ancient populations inferred from four population symmetry-f_4-statistics of the form *f_4(Inland Neolithic northern East Asian1, Inland Neolithic northern East Asian2; Eastern Modern Tibetan/Ancient East Asians, Mbuti)*. 21
Figure S37. Shared ancestry associated with Bronze Age to historic period from Nepal in modern Tibetans and ancient East Asians compared with geographically different ancient populations inferred from four population symmetry-f_4-statistics of the form *f_4(Nepal Ancient1, Nepal Ancient2; Eastern Modern Tibetan/Ancient East Asians, Mbuti)*. 22

Similarities and differences of the shared genetic profiles related to northern Neolithic East Asians via the spatial comparison analysis in modern Tibetans and all available ancient East Asians. 23

Figure S38. Spatial comparison analysis showed the shared ancestry related to early northern Neolithic East Asians in modern Tibetans and all available ancient East Asians inferred from four population symmetry-f_4-statistics of the form *f_4(Bianbian_EN, Ancient Northern East Asians; Modern Tibetan/Neolithic to Historic East Asians, Mbuti)*. 23
Figure S39. Spatial comparison analysis showed the shared ancestry related to early northern Neolithic East Asians in modern Tibetans and all available ancient East Asians inferred from four population symmetry-f_4-statistics of the form *f_4(Boshan_EN, Ancient Northern East Asians; Modern

Tibetan/Neolithic to Historic East Asians, Mbuti). **24**
Figure S40. Spatial comparison analysis showed the shared ancestry related to early northern Neolithic East Asians in modern Tibetans and all available ancient East Asians inferred from four population symmetry-f_4-statistics of the form *f_4(Xiaogao_EN, Ancient Northern East Asians; Modern Tibetan/Neolithic to Historic East Asians, Mbuti)*. **26**
Figure S41. Spatial comparison analysis showed the shared ancestry related to early northern Neolithic East Asians in modern Tibetans and all available ancient East Asians inferred from four population symmetry-f_4-statistics of the form *f_4(Xiaojingshan_EN, Ancient Northern East Asians; Modern Tibetan/Neolithic to Historic East Asians, Mbuti)*. **27**
Figure S42. Spatial comparison analysis showed the shared ancestry related to early northern Neolithic East Asians in modern Tibetans and all available ancient East Asians inferred from four population symmetry-f_4-statistics of the form *f_4(Yumin_EN, Ancient Northern East Asians; Modern Tibetan/Neolithic to Historic East Asians, Mbuti)*. **28**
Figure S43. Spatial comparison analysis showed the shared ancestry related to middle northern Neolithic East Asians in modern Tibetans and all available ancient East Asians inferred from four population symmetry-f_4-statistics of the form *f_4(Wanggou_MN, Ancient Northern East Asians; Modern Tibetan/Neolithic to Historic East Asians, Mbuti)*. **29**
Figure S44. Spatial comparison analysis showed the shared ancestry related to middle northern Neolithic East Asians in modern Tibetans and all available ancient East Asians inferred from four population symmetry-f_4-statistics of the form *f_4(Xiaowu_MN, Ancient Northern East Asians; Modern Tibetan/Neolithic to Historic East Asians, Mbuti)*. **31**
Figure S45. Spatial comparison analysis showed the shared ancestry related to late northern Neolithic East Asians in modern Tibetans and all available ancient East Asians inferred from four population symmetry-f_4-statistics of the form *f_4(Haojiatai_LN, Ancient Northern East Asians; Modern Tibetan/Neolithic to Historic East Asians, Mbuti)*. **32**
Figure S46. Spatial comparison analysis showed the shared ancestry related to late northern Neolithic East Asians in modern Tibetans and all available ancient East Asians inferred from four population symmetry-f_4-statistics of the form *f_4(Wadian_LN, Ancient Northern East Asians; Modern Tibetan/Neolithic to Historic East Asians, Mbuti)*. **33**
Figure S47. Spatial comparison analysis showed the shared ancestry related to late northern Neolithic East Asians in modern Tibetans and all available ancient East Asians inferred from four population symmetry-f_4-statistics of the form *f_4(Lajia_LN, Ancient Northern East Asians; Modern Tibetan/Neolithic to Historic East Asians, Mbuti)*. **34**
Figure S48. Spatial comparison analysis showed the shared ancestry related to late northern Neolithic East Asians in modern Tibetans and all available ancient East Asians inferred from four population symmetry-f_4-statistics of the form *f_4(Shimao_LN, Ancient Northern East Asians; Modern Tibetan/Neolithic to Historic East Asians, Mbuti)*. **35**
Figure S49. Spatial comparison analysis showed the shared ancestry related to the Ancient Nepal population in modern Tibetans and all available ancient East Asians inferred from four population symmetry-f_4-statistics of the form *f_4(Chokhopani, Ancient Northern East Asians; Modern Tibetan/Neolithic to Historic East Asians, Mbuti)*. **37**

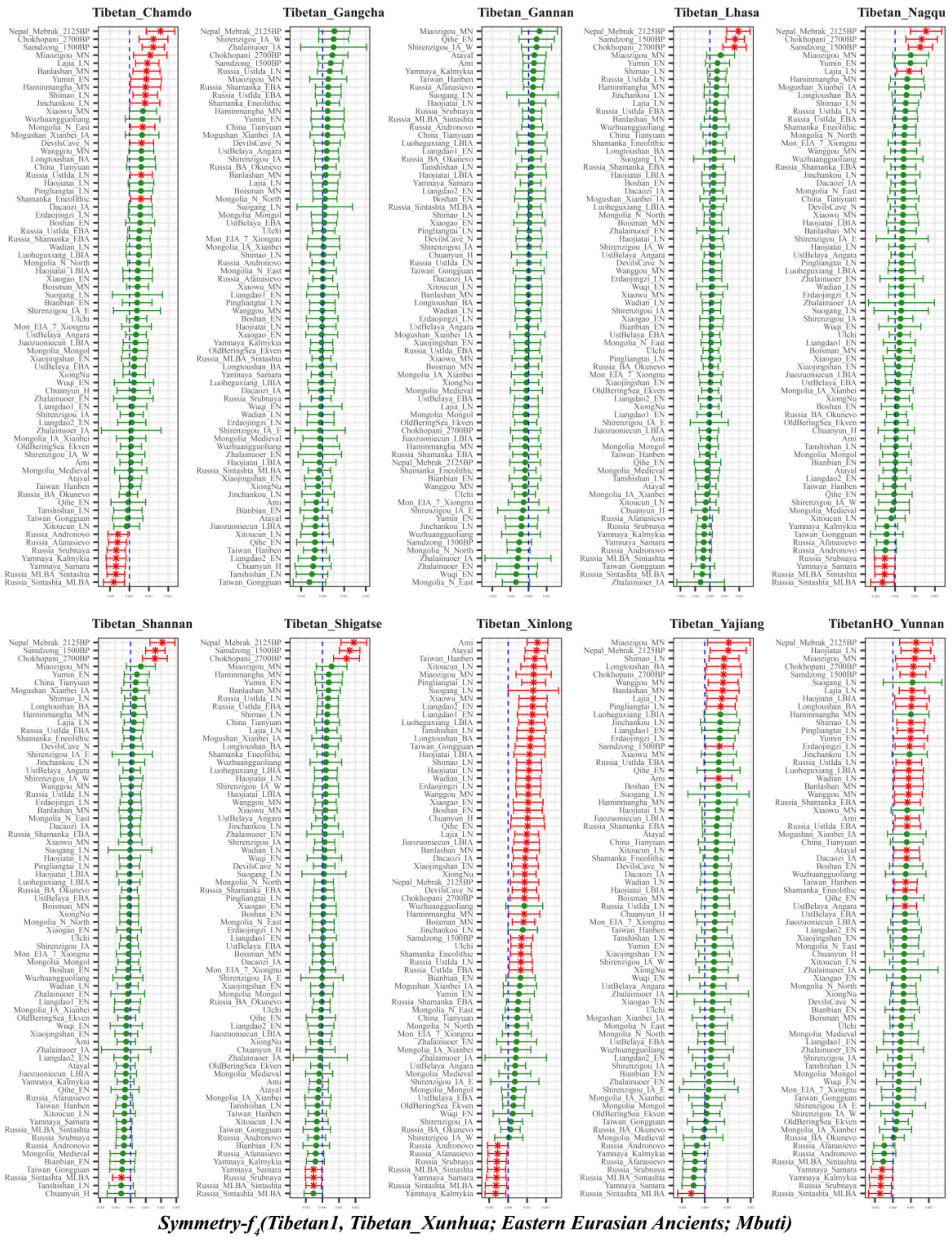

Figure S21. Genomic affinity between modern Tibetans and eastern Eurasian ancient populations inferred from four population symmetry-f_4 statistics of the form f_4*(Tibetan1, Xunhua Tibetan; eastern Eurasian ancients, Mbuti)*.

Here, overlapping SNP loci included in the Affymetrix Human Origins platform among four analyzed populations were used. We used the genetic variation of Mbuti as the outgroup. Red asterisk point meant the significant value (Absolute value of Z-scores larger than three or equal to three) observed in the symmetry-f_4 statistics and green circle point denoted the non-significant f_4-statistic values (Absolute value of Z-scores less than three). All reference modern and ancient East Asian populations were listed along the Y-axis and f_4 values were labeled along the X-axis. All tested population pairs were faceted or grouped via the Tibetan1. Significant negative f_4 values indicated that the included eastern Eurasian ancient population shared more alleles with Xunhua Tibetan compared with Tibetan1 or Xunhua Tibetan harbored increased eastern Eurasian ancient population-related ancestry compared with Tibetan1, and significant positive f_4 value indicated that the included eastern Eurasian ancient population shared more

derived alleles with Tibetan1 compared with Xunhua Tibetan or elucidated as Xunhua Tibetan had increased eastern Eurasian ancient population-related ancestry relative to Tibetan1. The value of f_4-statistics equal to zero was marked as the blue dash line. The bar indicated three standard errors.

Results of non-significant f_4-statistic of the form f_4*(Gangcha/Gannan Tibetans, Xunhua Tibetan; eastern Eurasian ancients, Mbuti)* showed that both Gangcha Tibetan and Gannan Tibetan formed one clade with Xunhua Tibetan. Compared with five Tibetans from Tibet Province harboring more Nepal ancient-related ancestry, significant signals of western Eurasian Steppe population affinity with Xunhua Tibetan were observed except for Lhasa Tibetan via f_4*(Chamdo/Lhasa/Nagqu/Shannan/Shigatse Tibetans, Xunhua Tibetan; eastern Eurasian ancients, Mbuti)*. Compared with lowland Tibetans, the Steppe-related population affinity with Xunhua Tibetan was also observed.

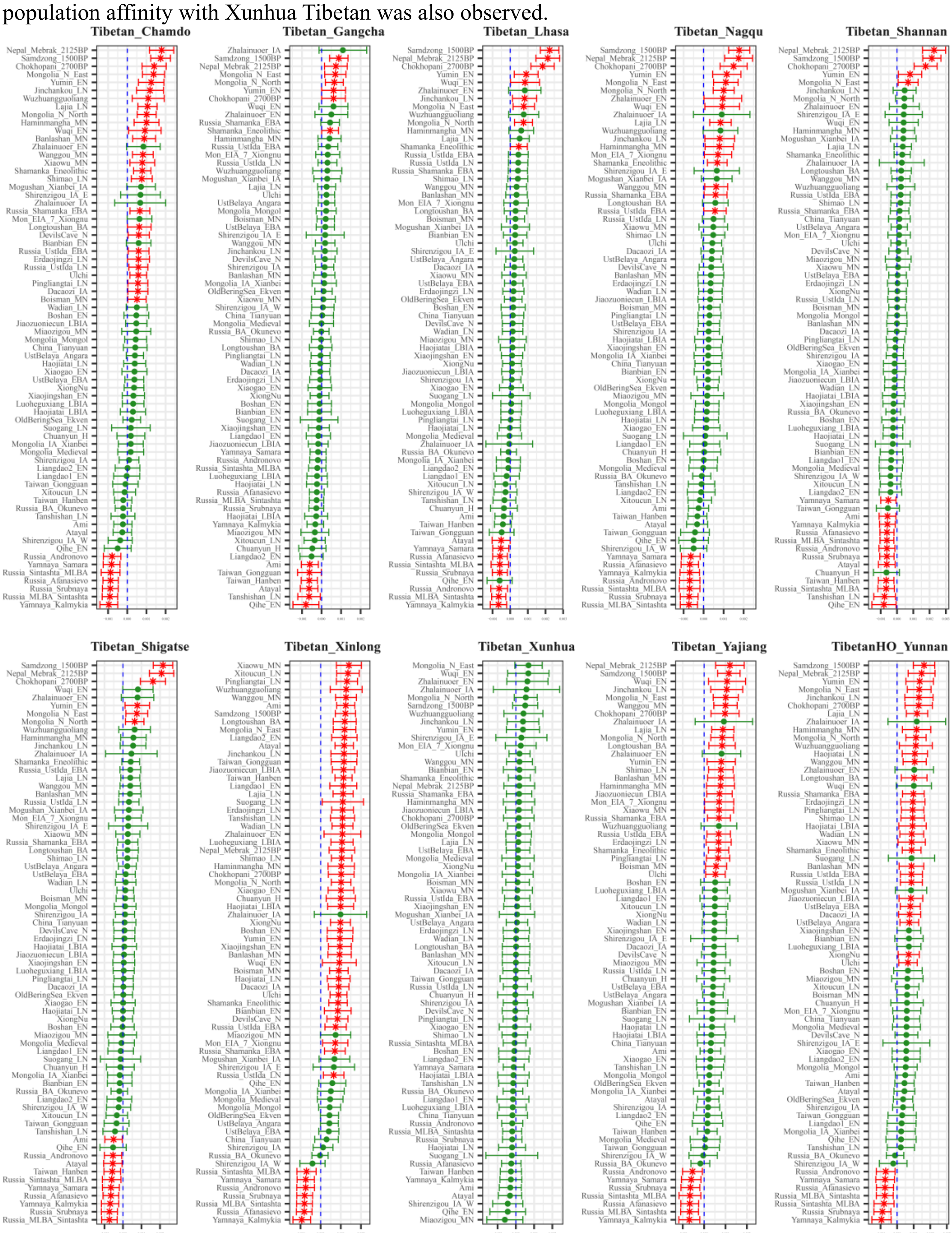

Symmetry-f_4(Tibetan1, Tibetan_Gannan; Eastern Eurasian Ancients; Mbuti)

Figure S22. Genomic affinity between modern Tibetans and eastern Eurasian ancient populations inferred from four population symmetry-f_4 statistics of the form f_4*(Tibetan1, Gannan Tibetan; eastern Eurasian ancients, Mbuti)*.

Here, overlapping SNP loci included in the Affymetrix Human Origins platform among four analyzed populations were used. We used the genetic variation of Mbuti as the outgroup. Red asterisk point meant the significant value (Absolute value of Z-scores larger than three or equal to three) observed in the symmetry-f_4 statistics and green circle point denoted the non-significant f_4-statistic values (Absolute value of Z-scores less than three). All reference modern and ancient East Asian populations were listed along the Y-axis and f_4 values were labeled along the X-axis. All tested population pairs were faceted or grouped via the Tibetan1. Significant negative f_4 values indicated that the included eastern Eurasian ancient population shared more alleles with Gannan Tibetan compared with Tibetan1 or Gannan Tibetan harbored increased eastern Eurasian ancient population-related ancestry compared with Tibetan1, and significant positive f_4 value indicated that the included eastern Eurasian ancient population shared more derived alleles with Tibetan1 compared with Gannan Tibetan or elucidated as Gannan Tibetan had increased eastern Eurasian ancient population-related ancestry relative to Tibetan1. The value of f_4-statistics equal to zero was marked as the blue dash line. The bar indicated three standard errors.

As non-significant signals were observed in f_4*(Xunhua Tibetan, Gannan Tibetan; eastern Eurasian ancients, Mbuti)*, Gannan Tibetan formed one clade with Xunhua Tibetan.

For five Tibetans from Tibet Province, Gannan Tibetan had increased ancestry related to early and middle Bronze Age Steppe Yamnaya ancestry (Yamnaya_Kalmykia, Yamnaya_Samara), and also owned more Sintashta-like ancestry (Russia_MLBA_Sintashta, Russia_Sintashta_MLBA) and Srubnaya-/Afanasievo-/Andronovo-like ancestry (Russia_Srubnaya, Russia_Afanasievo, Russia_Andronovo) relative to Shigatse Tibetan. We also found that Gannan Tibetan had increased ancestry related to coastal Neolithic to Modern southern East Asians (Taiwan_Hanben, Atayal and Qihe_EN) compared with Shigatse Tibetan.

Relative to Shannan Tibetan, Gannan Tibetan harbored increased ancestry related to Bonze Age Steppe Pastoralists (Sintashta: Russia_Sintashta_MLBA and Russia_MLBA_Sintashta, Yamnaya: Yamnaya_Kalmykia, Yamnaya_Samara and others: Russia_Srubnaya, Russia_Andronovo, Russia_Afanasievo) and it also harbored more coastal Neolithic to modern southern East Asian ancestry (Qihe_EN, Tanshishan_LN, Taiwan_Hanben, Atayal and Ami).

Relative to Lhasa Tibetan, Gannan Tibetan shared more coastal early Neolithic southern East Asian Qihe ancestry and modern southern East Asian Atayal ancestry, and it also owned increased ancestry related to the Steppe pastoralists (Yamnaya_Kalmykia, Yamnaya_Samara, Russia_MLBA_Sintashta, Russia_Sintashta_MLBA, Russia_Andronovo, Russia_Srubnaya, Russia_Afanasievo).

Relative to Nagqu Tibetan, Gannan Tibetan harbored more western Eurasian Stepped Pastoralists-related ancestry (Russia_MLBA_Sintashta, Russia_Srubnaya, Russia_Sintashta_MLBA, Russia_Andronovo, Yamnaya_Kalmykia, Russia_Afanasievo and Yamnaya_Samara). Similar patterns of Steppe Pastoralists affinity were observed in f_4*(Chamdo Tibetan, Gannan Tibetan; early and middle Bronze Age Steppe Pastoralists, Mbuti)*.

Compared with Gangcha Tibetan from Qinghai Province, Gannan Tibetan harbored increased ancestry related to coastal Neolithic to Bronze Age to modern southern East Asian (Qihe_EN, Tanshishan_LN, Atayal, Taiwan_Hanben, Taiwan_Gongguan and Ami).

The results of f_4*(Xinlong/Yajiang Tibetan, Gannan Tibetan; early and middle Bronze Age Steppe Pastoralists, Mbuti)* showed significant negative f_4-statistics values, which suggested more Steppe Pastoralists-related ancestry in Gannan Tibetan than in Xinjiang Tibetan and Yajiang Tibetan from Sichuan Province. This western Eurasian Steppe Pastoralist affinity was also identified via f_4*(Yunnan Tibetan, Gannan Tibetan; early and middle Bronze Age Steppe Pastoralists, Mbuti)*.

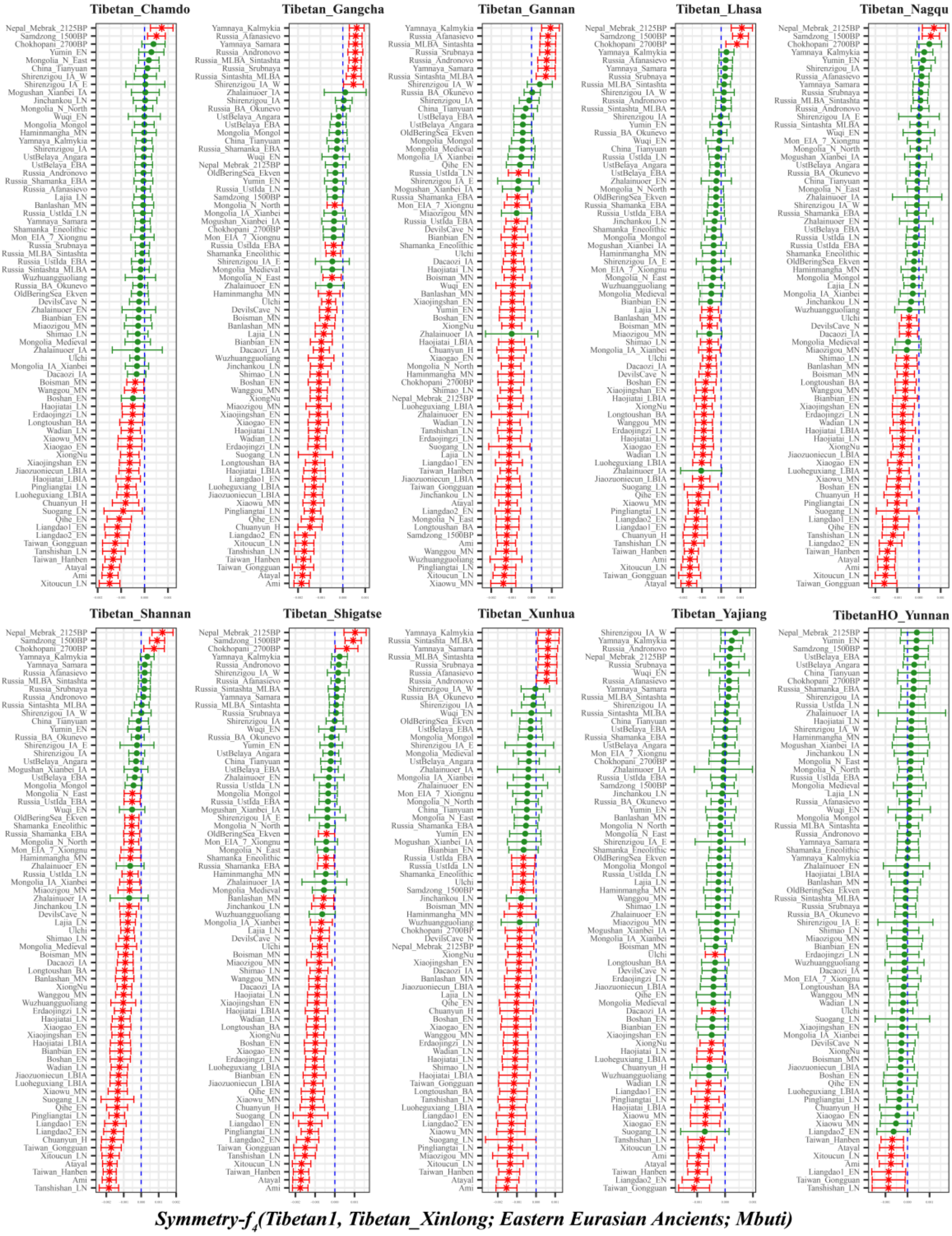

Figure S23. Genomic affinity between modern Tibetans and eastern Eurasian ancient populations inferred from four population symmetry-f_4 statistics of the form f_4*(Tibetan1, Xinlong Tibetan; eastern Eurasian ancients, Mbuti)*.

Here, overlapping SNP loci included in the Affymetrix Human Origins platform among four analyzed populations were used. We used the genetic variation of Mbuti as the outgroup. Red asterisk point meant the significant value (Absolute value of Z-scores larger than three or equal to three) observed in the symmetry-f_4 statistics and green circle point denoted the non-significant f_4-statistic values (Absolute value of Z-scores less than three). All reference modern and ancient East Asian populations were listed along the Y-axis and f_4 values were labeled along the X-axis. All tested population pairs were faceted or grouped via the Tibetan1. Significant negative f_4 values indicated that the included eastern Eurasian ancient population shared more alleles with Xinlong Tibetan compared with Tibetan1 or Xinlong Tibetan harbored increased eastern Eurasian ancient population-related ancestry compared with Tibetan1, and significant positive f_4 value indicated that the included eastern Eurasian ancient population shared more

derived alleles with Tibetan1 compared with Xinlong Tibetan or elucidated as Xinlong Tibetan had increased eastern Eurasian ancient population-related ancestry relative to Tibetan1. The value of f_4-statistics equal to zero was marked as the blue dash line. The bar indicated three standard errors.

Compared to Yunnan Tibetan, Xinlong Tibetan harbored increased ancestry related to Coastal/island Neolithic to modern southern East Asian (Tanshishan_LN, Taiwan_Gongguan, Liangdao1_EN, Ami, Xitoucun_LN, Atayal and Taiwan_Hanben). Compared with other Tibetans, Xinlong Tibetan owned more lowland ancient or modern East Asian-related ancestry.

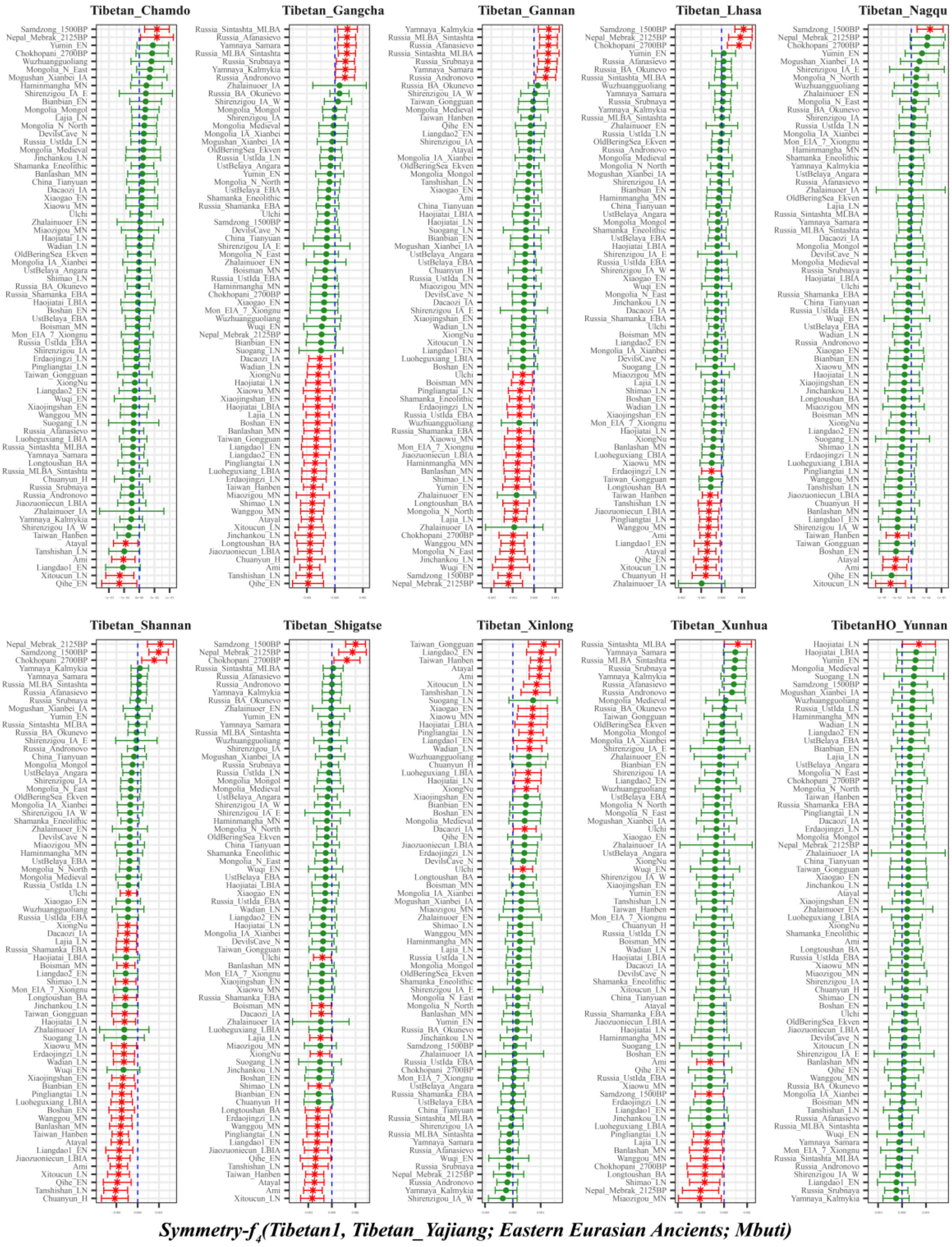

Figure S24. Genomic affinity between modern Tibetans and eastern Eurasian ancient populations inferred from four population symmetry-f_4 statistics of the form f_4*(Tibetan1, Yajiang Tibetan; eastern Eurasian ancients, Mbuti)*.

Here, overlapping SNP loci included in the Affymetrix Human Origins platform among four analyzed populations were used. We used the genetic variation of Mbuti as the outgroup. Red asterisk point meant

the significant value (Absolute value of Z-scores larger than three or equal to three) observed in the symmetry-f_4 statistics and green circle point denoted the non-significant f_4-statistic values (Absolute value of Z-scores less than three). All reference modern and ancient East Asian populations were listed along the Y-axis and f_4 values were labeled along the X-axis. All tested population pairs were faceted or grouped via the Tibetan1. Significant negative f_4 values indicated that the included eastern Eurasian ancient population shared more alleles with Yajiang Tibetan compared with Tibetan1 or Yajiang Tibetan harbored increased eastern Eurasian ancient population-related ancestry compared with Tibetan1, and significant positive f_4 value indicated that the included eastern Eurasian ancient population shared more derived alleles with Tibetan1 compared with Yajiang Tibetan or elucidated as Yajiang Tibetan had increased eastern Eurasian ancient population-related ancestry relative to Tibetan1. The value of f_4-statistics equal to zero was marked as the blue dash line. The bar indicated three standard errors.

Compared with Xinlong Tibetan and Yunnan Tibetan, no signals of additional shared ancestry from other source populations into Yajiang Tibetan were identified, as no significant negative f_4-statistics were observed in *f_4(Xinlong/Yunnan Tibetan, Yajiang Tibetan; eastern Eurasian ancients, Mbuti)*. Compared with other Tibetans, Yajiang harbored increased lowland East Asian ancestry and especially for more coastal southern East Asian ancestry. We also find more highland Nepal ancient-related ancestry in Yajiang Tibetan relative to Gannan Tibetan and Xunhua Tibetan.

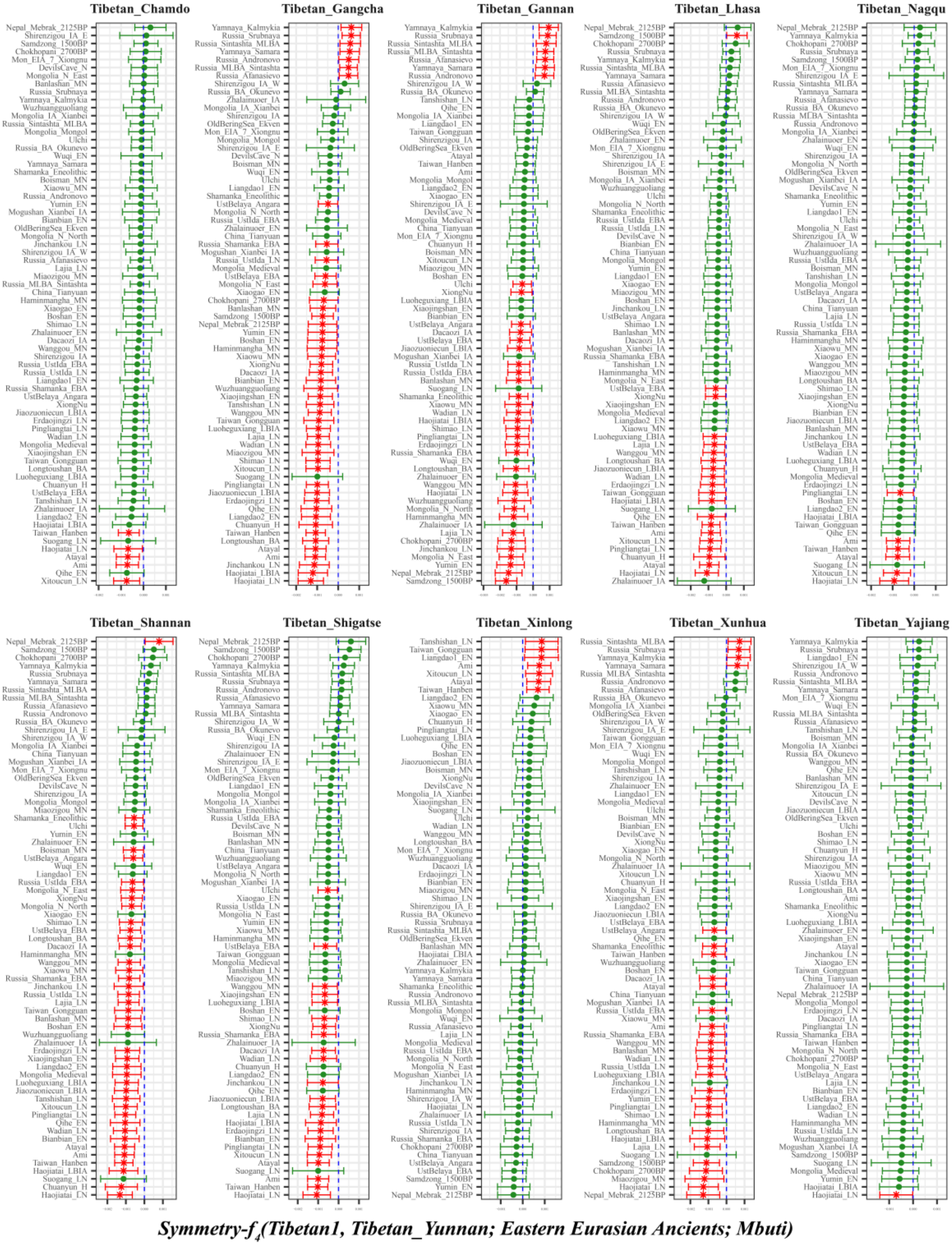

Figure S25. Genomic affinity between modern Tibetans and eastern Eurasian ancient populations inferred from four population symmetry-f_4 statistics of the form f_4*(Tibetan1, Yunnan Tibetan; eastern Eurasian ancients, Mbuti)*.

Here, overlapping SNP loci included in the Affymetrix Human Origins platform among four analyzed populations were used. We used the genetic variation of Mbuti as the outgroup. Red asterisk point meant the significant value (Absolute value of Z-scores larger than three or equal to three) observed in the symmetry-f_4 statistics and green circle point denoted the non-significant f_4-statistic values (Absolute value of Z-scores less than three). All reference modern and ancient East Asian populations were listed along the Y-axis and f_4 values were labeled along the X-axis. All tested population pairs were faceted or grouped via the Tibetan1. Significant negative f_4 values indicated that the included eastern Eurasian ancient population shared more alleles with Yunnan Tibetan compared with Tibetan1 or Yunnan Tibetan harbored increased eastern Eurasian ancient population-related ancestry compared with Tibetan1, and

significant positive f_4 value indicated that the included eastern Eurasian ancient population shared more derived alleles with Tibetan1 compared with Yunnan Tibetan or elucidated as Yunnan Tibetan had increased eastern Eurasian ancient population-related ancestry relative to Tibetan1. The value of f_4-statistics equal to zero was marked as the blue dash line. The bar indicated three standard errors.

Compared with Yajiang Tibetan and Xinlong Tibetan from Sichuan Province, no signals of additional gene flow into Yunnan Tibetan, except for Yunnan had more Neolithic Haojiatai-related ancestry related to Yajiang Tibetan. Relative to other Tibetans, Yunnan Tibetan harbored more lowland East Asian related ancestry. We also found that compared with Gannan Tibetan, Yunnan Tibetan had more Nepal ancients-related ancestry.

Spatiotemporal comparison analysis among modern Tibetan and all Paleolithic-to-historic East Asians showed the genetic admixture and continuity of modern Tibetans

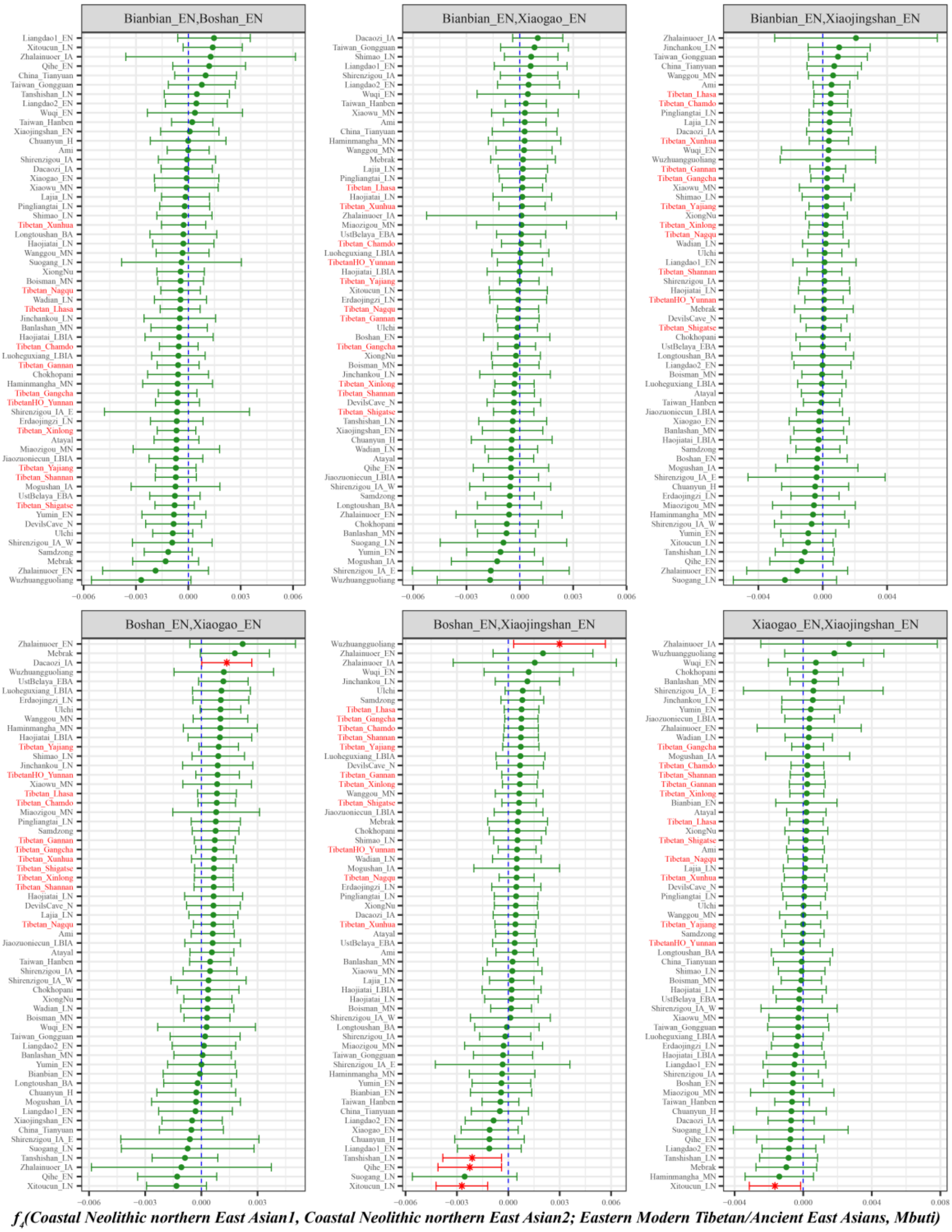

Figure S26. Shared ancestry associated with coastal Neolithic northern East Asian in modern Tibetans and ancient East Asians compared with geographically different coastal Neolithic northern East Asian inferred from four population symmetry-f_4-statistics of the form f_4*(Coastal Neolithic northern East Asian1, Coastal Neolithic northern East Asian2; Eastern Modern Tibetan/Ancient East Asians, Mbuti).*

Here, overlapping SNP loci included in the Affymetrix Human Origins platform among four analyzed populations were used. We used the genetic variation of Mbuti as the outgroup. Red asterisk point meant the significant value (Absolute value of Z-scores larger than three or equal to three) observed in the symmetry-f_4 statistics and green circle point denoted the non-significant f_4-statistic values (Absolute value of Z-scores less than three). All Eastern Modern Tibetan/Ancient East Asians were listed along the

Y-axis and f_4 values were labeled along the X-axis. All tested population pairs were faceted or grouped via the combination of Coastal Neolithic northern East Asian1 and Coastal Neolithic northern East Asian2. Significant negative f_4 values indicated that the third population shared more alleles with the second population and significant positive f_4 value indicated that the third population shared more derived alleles with the first population. The value of f_4-statistics equal to zero was marked as the blue dash line. The bar indicated three standard errors.

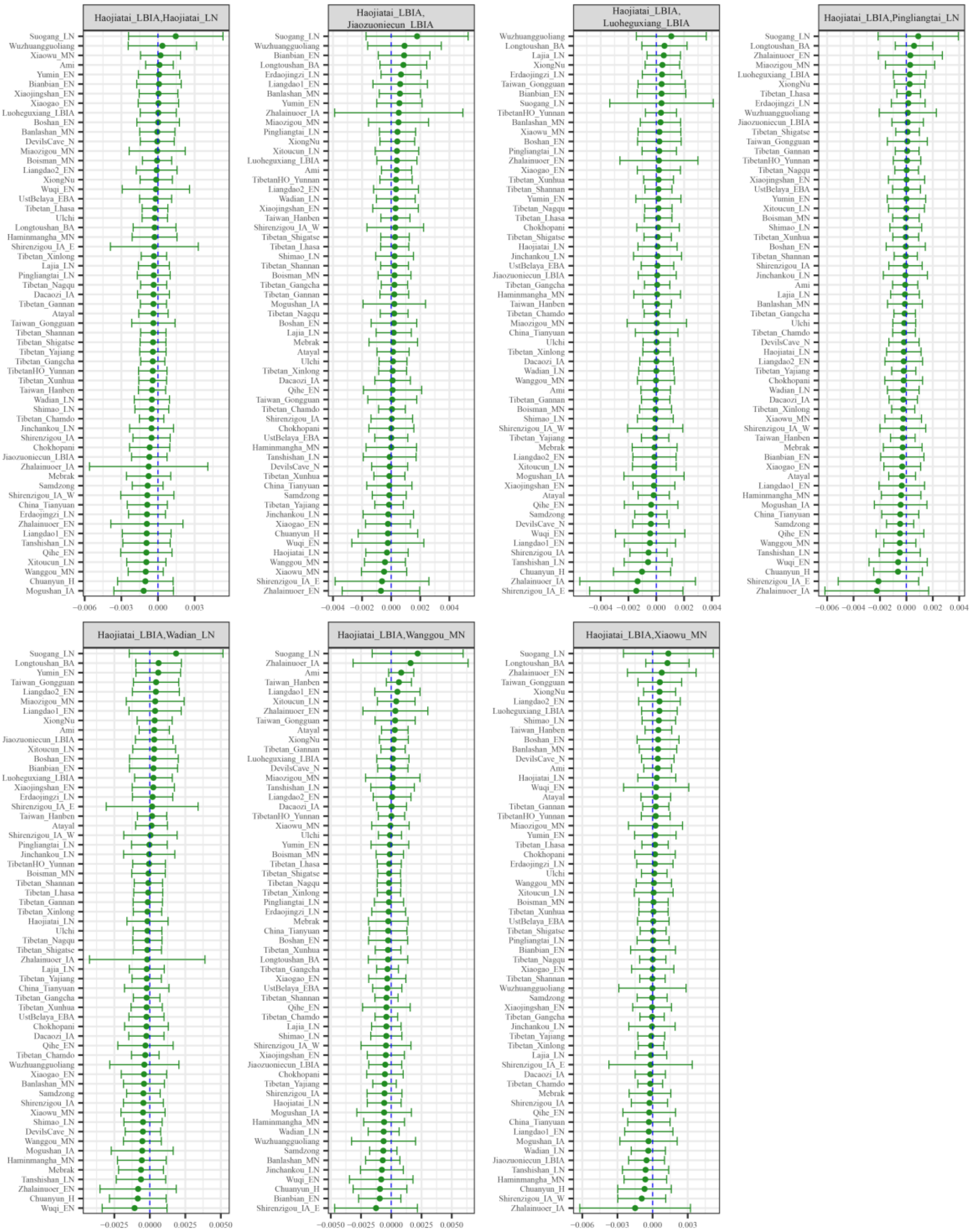

f_4(Haojiatai_LBIA, Neolithic to Iron Age Henan populations; Eastern Modern Tibetan/Ancient East Asians, Mbuti)

Figure S27. Shared ancestry associated with inland Neolithic to Iron Age northern East Asian from Henan province in modern Tibetans and ancient East Asians compared with geographically different ancient Henan populations inferred from four population symmetry-f_4-statistics of the form f_4*(Haojiatai_LBIA, Neolithic to Iron Age Henan populations; Eastern Modern Tibetan/Ancient East Asians, Mbuti)*.

Here, overlapping SNP loci included in the Affymetrix Human Origins platform among four analyzed populations were used. We used the genetic variation of Mbuti as the outgroup. Red asterisk point meant the significant value (Absolute value of Z-scores larger than three or equal to three) observed in the symmetry-f_4 statistics and green circle point denoted the non-significant f_4-statistic values (Absolute value of Z-scores less than three). All Eastern Modern Tibetan/Ancient East Asians were listed along the Y-axis and f_4 values were labeled along the X-axis. All tested population pairs were faceted or grouped via the combination of the first and second populations. Significant negative f_4 values indicated that the third population shared more alleles with the second population and significant positive f_4 value indicated that the third population shared more derived alleles with the first population. The value of f_4-statistics equal to zero was marked as the blue dash line. The bar indicated three standard errors.

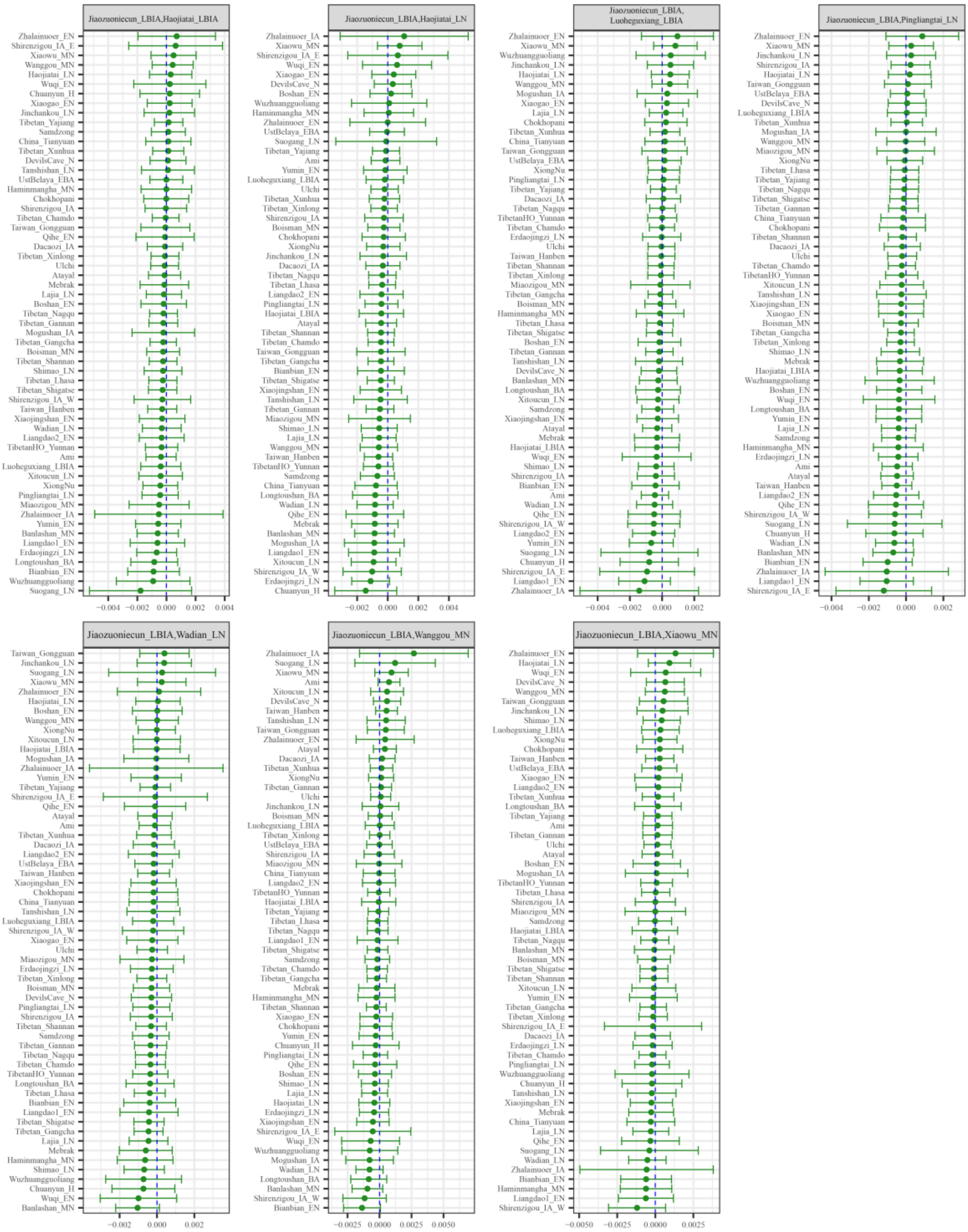

Figure S28. Shared ancestry associated with inland Neolithic to Iron Age northern East Asian from Henan province in modern Tibetans and ancient East Asians compared with geographically different ancient Henan populations inferred from four population symmetry-f_4-statistics of the form f_4*(Jiaozuoniecun_LBIA, Neolithic to Iron Age Henan populations; Eastern Modern Tibetan/Ancient East Asians, Mbuti)*.

Here, overlapping SNP loci included in the Affymetrix Human Origins platform among four analyzed populations were used. We used the genetic variation of Mbuti as the outgroup. Red asterisk point meant the significant value (Absolute value of Z-scores larger than three or equal to three) observed in the symmetry-f_4 statistics and green circle point denoted the non-significant f_4-statistic values (Absolute value of Z-scores less than three). All Eastern Modern Tibetan/Ancient East Asians were listed along the Y-axis and f_4 values were labeled along the X-axis. All tested population pairs were faceted or grouped via the combination of the first and second populations. Significant negative f_4 values indicated that the

third population shared more alleles with the second population and significant positive f_4 value indicated that the third population shared more derived alleles with the first population. The value of f_4-statistics equal to zero was marked as the blue dash line. The bar indicated three standard errors.

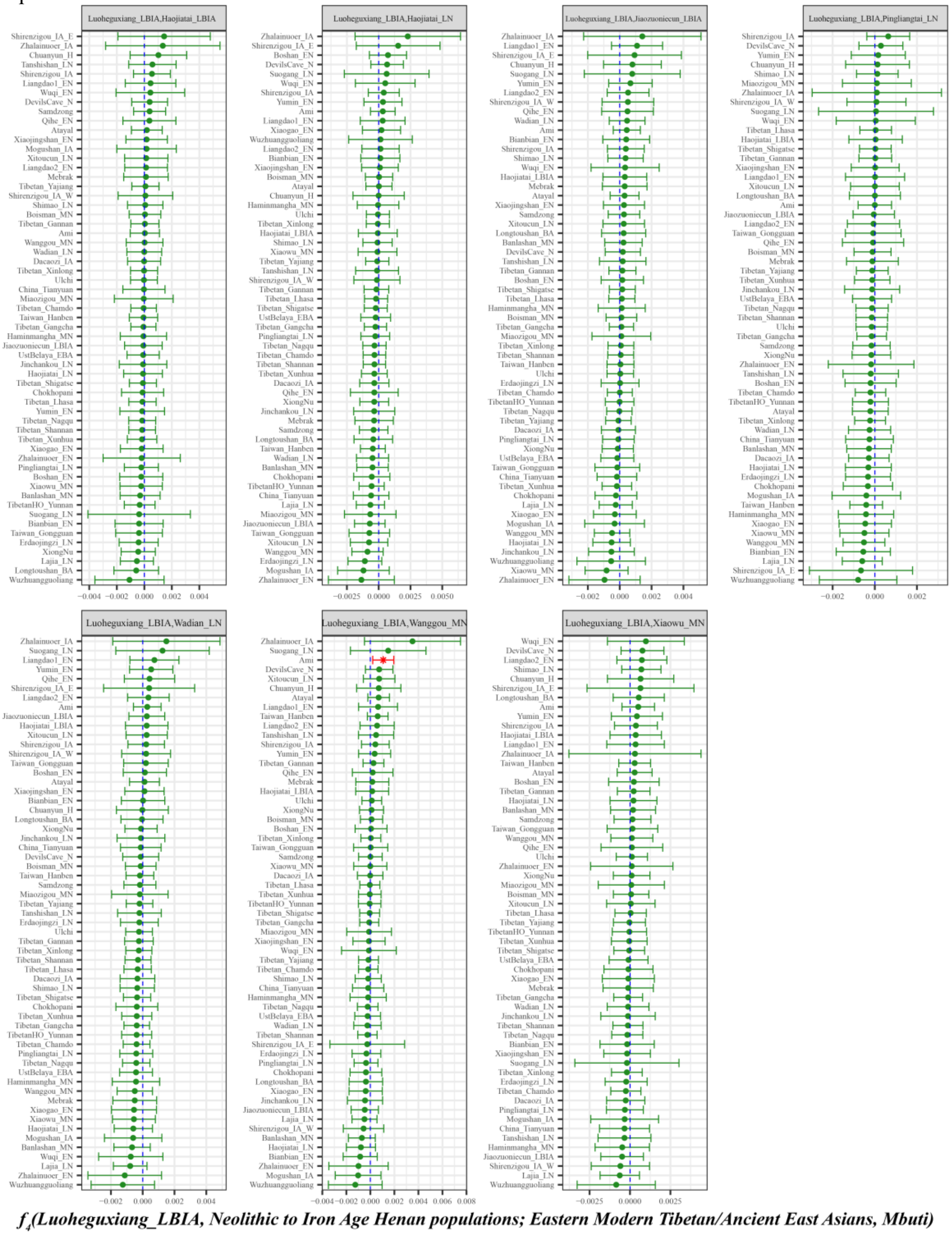

Figure S29. Shared ancestry associated with inland Neolithic to Iron Age northern East Asian from Henan province in modern Tibetans and ancient East Asians compared with geographically different ancient Henan populations inferred from four population symmetry-*f_4*-statistics of the form *f_4(Luoheguxiang_LBIA, Neolithic to Iron Age Henan populations; Eastern Modern Tibetan/Ancient East Asians, Mbuti)*.

Here, overlapping SNP loci included in the Affymetrix Human Origins platform among four analyzed populations were used. We used the genetic variation of Mbuti as the outgroup. Red asterisk point meant the significant value (Absolute value of Z-scores larger than three or equal to three) observed in the symmetry-f_4 statistics and green circle point denoted the non-significant f_4-statistic values (Absolute value of Z-scores less than three). All Eastern Modern Tibetan/Ancient East Asians were listed along the

Y-axis and f_4 values were labeled along the X-axis. All tested population pairs were faceted or grouped via the combination of the first and second populations. Significant negative f_4 values indicated that the third population shared more alleles with the second population and significant positive f_4 value indicated that the third population shared more derived alleles with the first population. The value of f_4-statistics equal to zero was marked as the blue dash line. The bar indicated three standard errors.

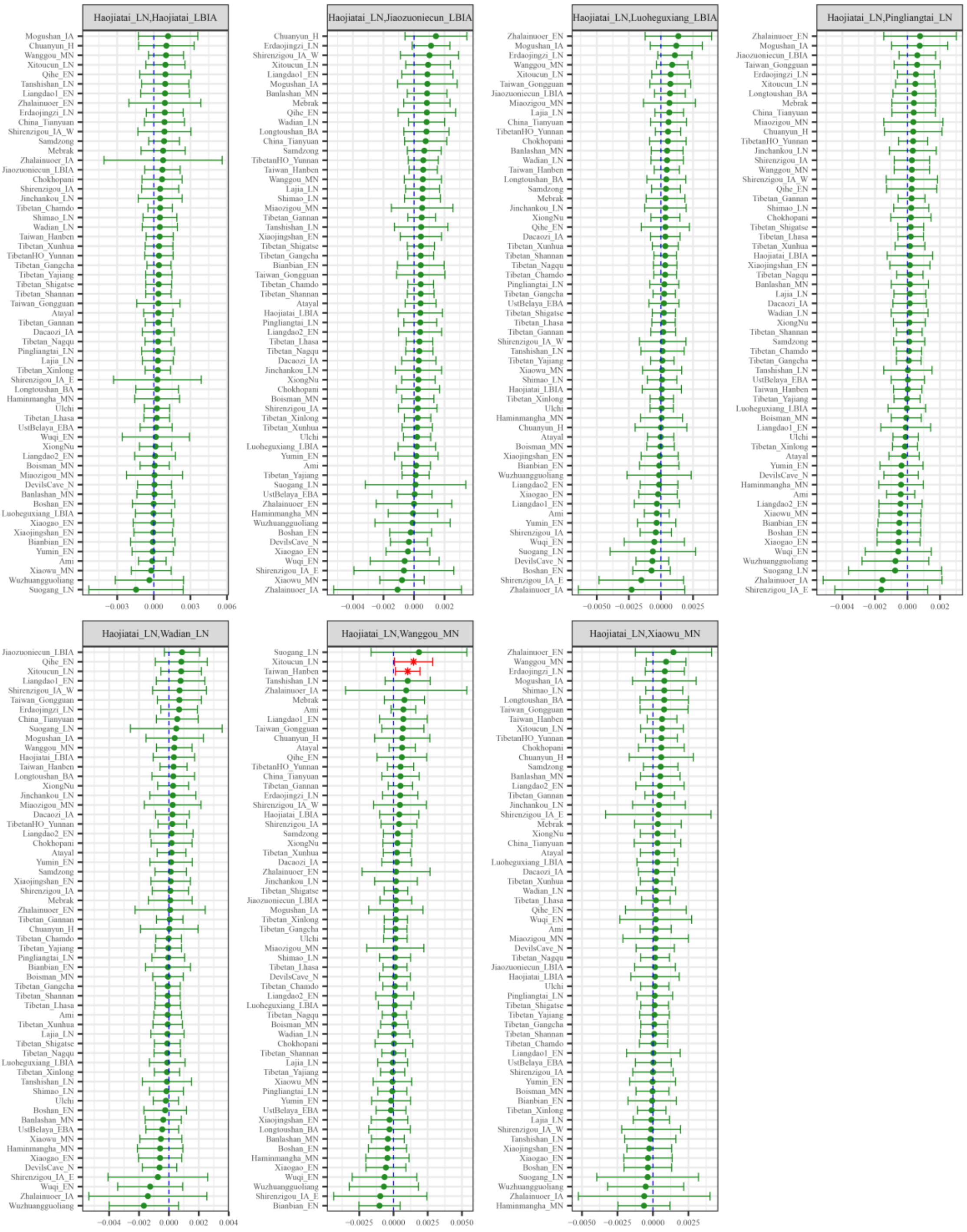

f4(Haojiatai_LN, Neolithic to Iron Age Henan populations; Eastern Modern Tibetan/Ancient East Asians, Mbuti)

Figure S30. Shared ancestry associated with inland Neolithic to Iron Age northern East Asian from Henan province in modern Tibetans and ancient East Asians compared with geographically different ancient Henan populations inferred from four population symmetry-f_4-statistics of the form f_4*(Haojiatai_LN, Neolithic to Iron Age Henan populations; Eastern Modern Tibetan/Ancient East Asians, Mbuti)*.

Here, overlapping SNP loci included in the Affymetrix Human Origins platform among four analyzed

populations were used. We used the genetic variation of Mbuti as the outgroup. Red asterisk point meant the significant value (Absolute value of Z-scores larger than three or equal to three) observed in the symmetry-f_4 statistics and green circle point denoted the non-significant f_4-statistic values (Absolute value of Z-scores less than three). All Eastern Modern Tibetan/Ancient East Asians were listed along the Y-axis and f_4 values were labeled along the X-axis. All tested population pairs were faceted or grouped via the combination of the first and second populations. Significant negative f_4 values indicated that the third population shared more alleles with the second population and significant positive f_4 value indicated that the third population shared more derived alleles with the first population. The value of f_4-statistics equal to zero was marked as the blue dash line. The bar indicated three standard errors.

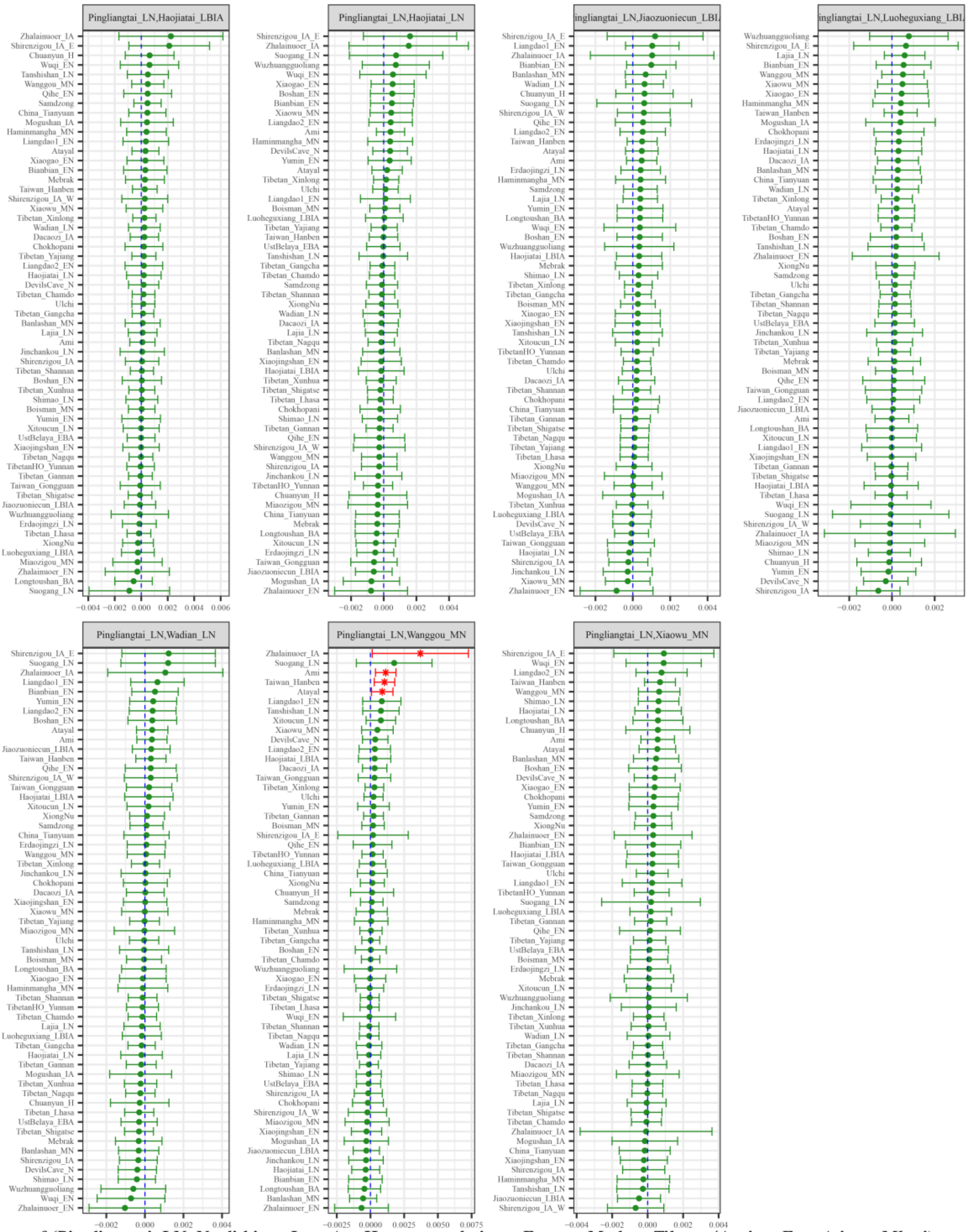

f_4(Pingliangtai_LN, Neolithic to Iron Age Henan populations; Eastern Modern Tibetan/Ancient East Asians, Mbuti)

Figure S31. Shared ancestry associated with inland Neolithic to Iron Age northern East Asian from Henan province in modern Tibetans and ancient East Asians compared with geographically different ancient Henan populations inferred from four population symmetry-*f_4*-statistics of the form *f_4(Pingliangtai_LN, Neolithic to Iron Age Henan populations; Eastern Modern Tibetan/Ancient East Asians, Mbuti)*.

Here, overlapping SNP loci included in the Affymetrix Human Origins platform among four analyzed populations were used. We used the genetic variation of Mbuti as the outgroup. Red asterisk point meant the significant value (Absolute value of Z-scores larger than three or equal to three) observed in the symmetry-f_4 statistics and green circle point denoted the non-significant f_4-statistic values (Absolute value of Z-scores less than three). All Eastern Modern Tibetan/Ancient East Asians were listed along the Y-axis and f_4 values were labeled along the X-axis. All tested population pairs were faceted or grouped via the combination of the first and second populations. Significant negative f_4 values indicated that the third population shared more alleles with the second population and significant positive f_4 value indicated that the third population shared more derived alleles with the first population. The value of f_4-statistics equal to zero was marked as the blue dash line. The bar indicated three standard errors.

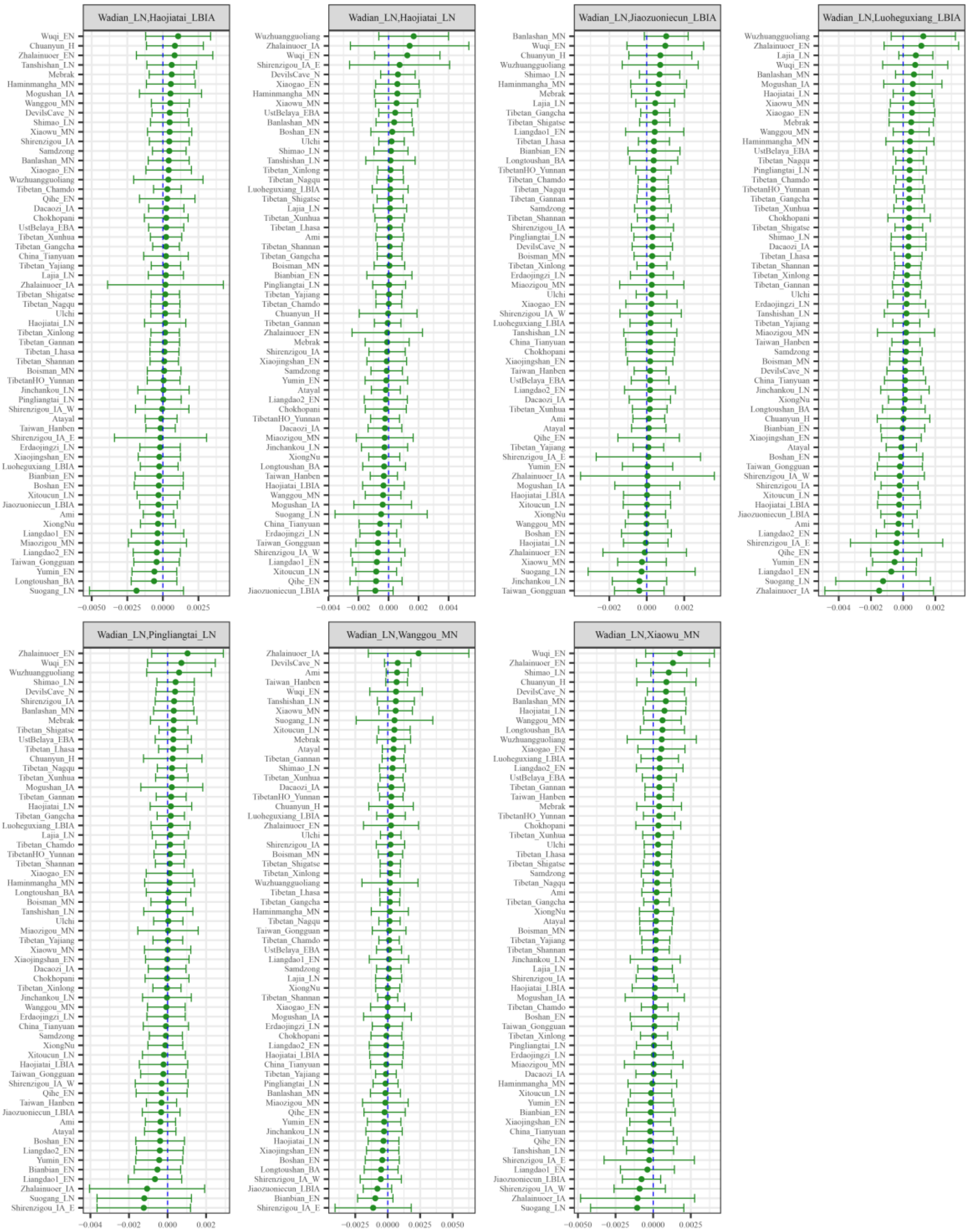

f_4(Wadian_LN, Neolithic to Iron Age Henan populations; Eastern Modern Tibetan/Ancient East Asians, Mbuti)

Figure S32. Shared ancestry associated with inland Neolithic to Iron Age northern East Asian from Henan province in modern Tibetans and ancient East Asians compared with geographically different ancient Henan populations inferred from four population symmetry-f_4-statistics of the form f_4*(Wadian_LN, Neolithic to Iron Age Henan populations; Eastern Modern Tibetan/Ancient East Asians, Mbuti)*.

Here, overlapping SNP loci included in the Affymetrix Human Origins platform among four analyzed populations were used. We used the genetic variation of Mbuti as the outgroup. Red asterisk point meant the significant value (Absolute value of Z-scores larger than three or equal to three) observed in the symmetry-f_4 statistics and green circle point denoted the non-significant f_4-statistic values (Absolute value of Z-scores less than three). All Eastern Modern Tibetan/Ancient East Asians were listed along the Y-axis and f_4 values were labeled along the X-axis. All tested population pairs were faceted or grouped via the combination of the first and second populations. Significant negative f_4 values indicated that the third population shared more alleles with the second population and significant positive f_4 value indicated

that the third population shared more derived alleles with the first population. The value of f_4-statistics equal to zero was marked as the blue dash line. The bar indicated three standard errors.

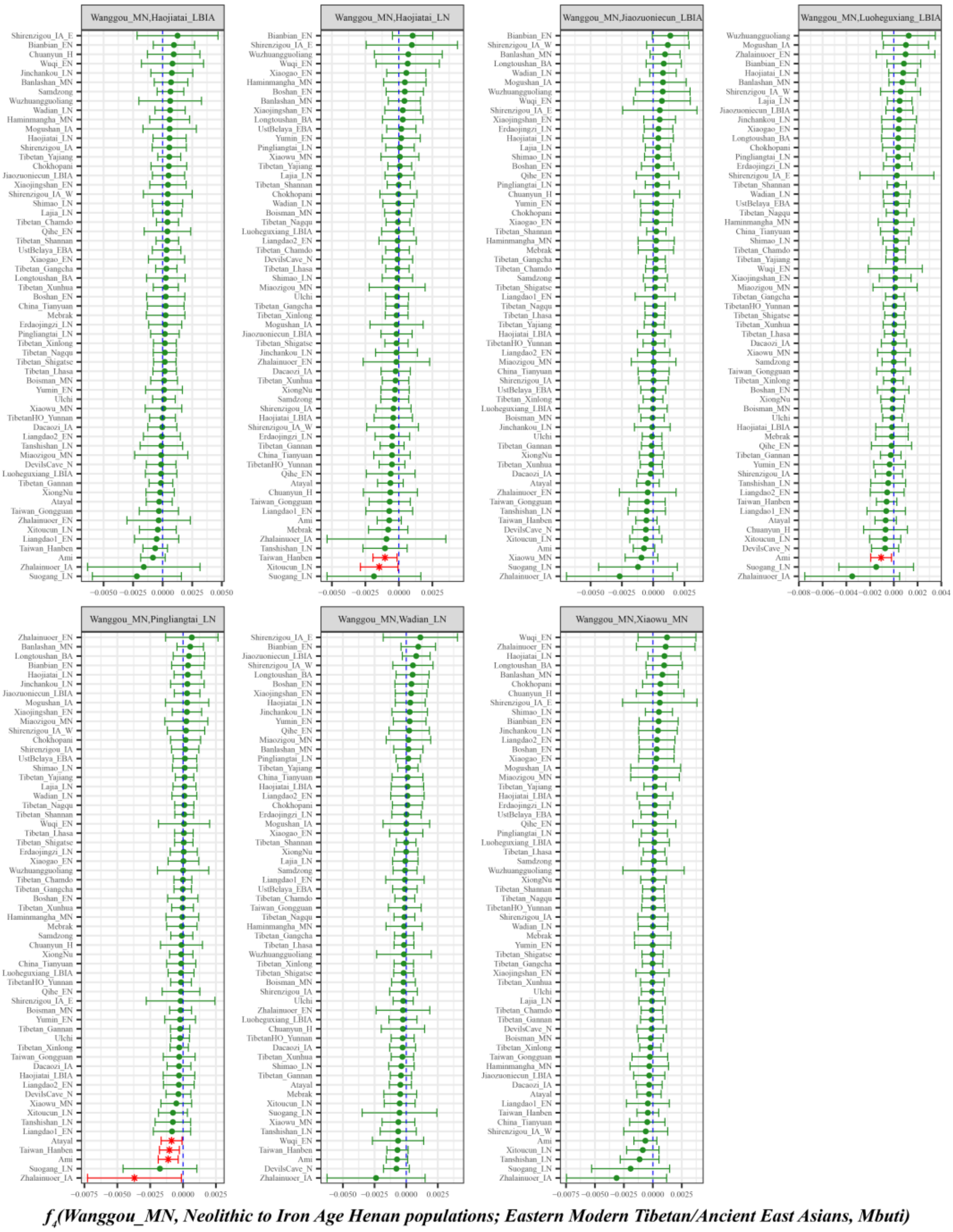

Figure S33. Shared ancestry associated with inland Neolithic to Iron Age northern East Asian from Henan province in modern Tibetans and ancient East Asians compared with geographically different ancient Henan populations inferred from four population symmetry-f_4-statistics of the form f_4*(Wanggou_MN, Neolithic to Iron Age Henan populations; Eastern Modern Tibetan/Ancient East Asians, Mbuti)*.

Here, overlapping SNP loci included in the Affymetrix Human Origins platform among four analyzed populations were used. We used the genetic variation of Mbuti as the outgroup. Red asterisk point meant the significant value (Absolute value of Z-scores larger than three or equal to three) observed in the symmetry-f_4 statistics and green circle point denoted the non-significant f_4-statistic values (Absolute value of Z-scores less than three). All Eastern Modern Tibetan/Ancient East Asians were listed along the Y-axis and f_4 values were labeled along the X-axis. All tested population pairs were faceted or grouped via the combination of the first and second populations. Significant negative f_4 values indicated that the third population shared more alleles with the second population and significant positive f_4 value indicated that the third population shared more derived alleles with the first population. The value of f_4-statistics

equal to zero was marked as the blue dash line. The bar indicated three standard errors.

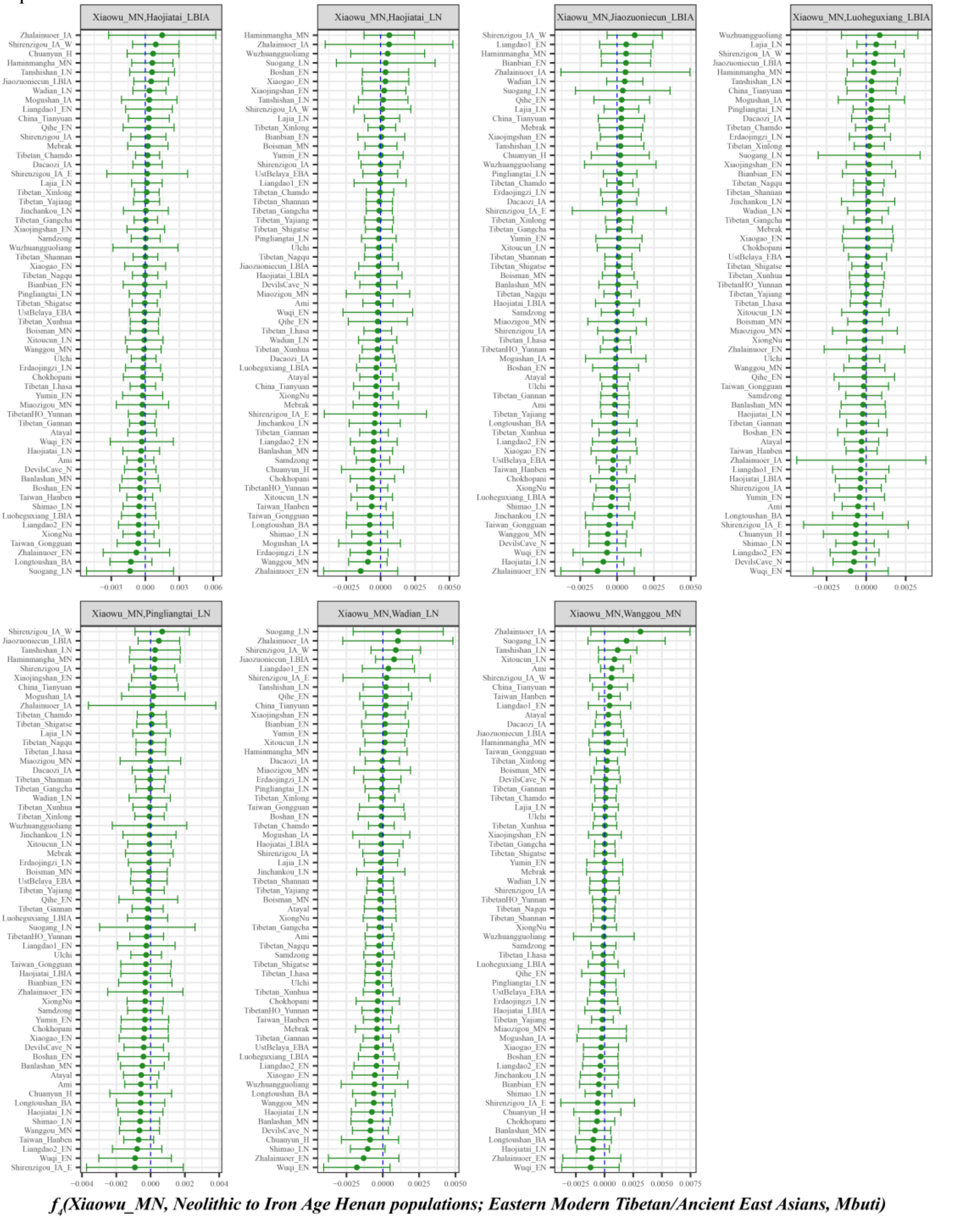

Figure S34. Shared ancestry associated with inland Neolithic to Iron Age northern East Asian from Henan province in modern Tibetans and ancient East Asians compared with geographically different ancient Henan populations inferred from four population symmetry-f_4-statistics of the form f_4*(Xiaowu_MN, Neolithic to Iron Age Henan populations; Eastern Modern Tibetan/Ancient East Asians, Mbuti)*.

Here, overlapping SNP loci included in the Affymetrix Human Origins platform among four analyzed populations were used. We used the genetic variation of Mbuti as the outgroup. Red asterisk point meant the significant value (Absolute value of Z-scores larger than three or equal to three) observed in the symmetry-f_4 statistics and green circle point denoted the non-significant f_4-statistic values (Absolute value of Z-scores less than three). All Eastern Modern Tibetan/Ancient East Asians were listed along the Y-axis and f_4 values were labeled along the X-axis. All tested population pairs were faceted or grouped via the combination of the first and second populations. Significant negative f_4 values indicated that the third population shared more alleles with the second population and significant positive f_4 value indicated

that the third population shared more derived alleles with the first population. The value of f_4-statistics equal to zero was marked as the blue dash line. The bar indicated three standard errors.

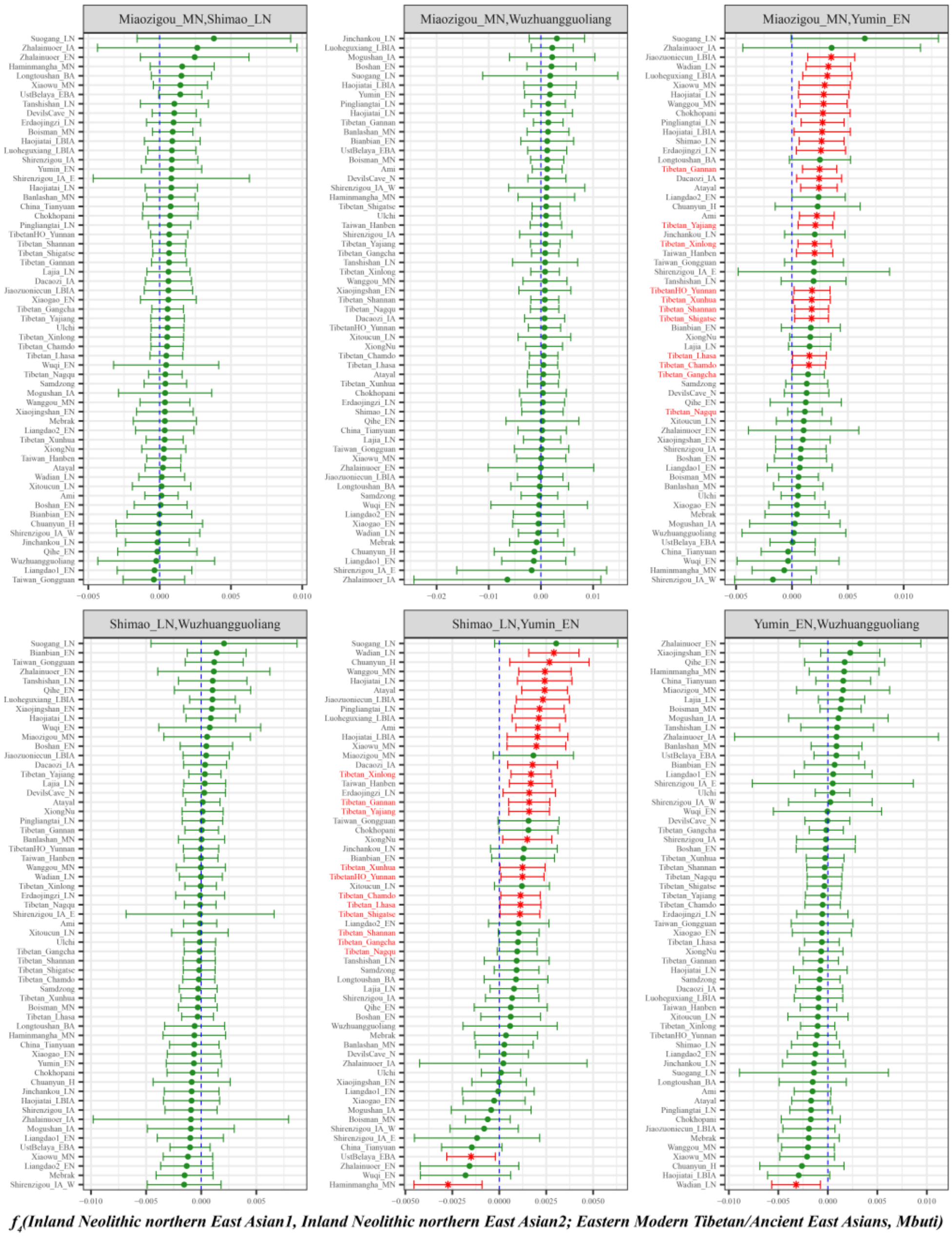

Figure S35. Shared ancestry associated with inland Neolithic northern East Asian from Shaanxi and Inner Mongolia provinces in modern Tibetans and ancient East Asians compared with geographically different ancient populations inferred from four population symmetry-f_4-statistics of the form f_4*(Inland Neolithic northern East Asian1, Inland Neolithic northern East Asian2; Eastern Modern Tibetan/Ancient East Asians, Mbuti)*.

Here, overlapping SNP loci included in the Affymetrix Human Origins platform among four analyzed populations were used. We used the genetic variation of Mbuti as the outgroup. Red asterisk point meant the significant value (Absolute value of Z-scores larger than three or equal to three) observed in the symmetry-f_4 statistics and green circle point denoted the non-significant f_4-statistic values (Absolute value of Z-scores less than three). All Eastern Modern Tibetan/Ancient East Asians were listed along the Y-axis and f_4 values were labeled along the X-axis. All tested population pairs were faceted or grouped via the combination of the first and second populations. Significant negative f_4 values indicated that the third population shared more alleles with the second population and significant positive f_4 value indicated that the third population shared more derived alleles with the first population. The value of f_4-statistics equal to zero was marked as the blue dash line. The bar indicated three standard errors.

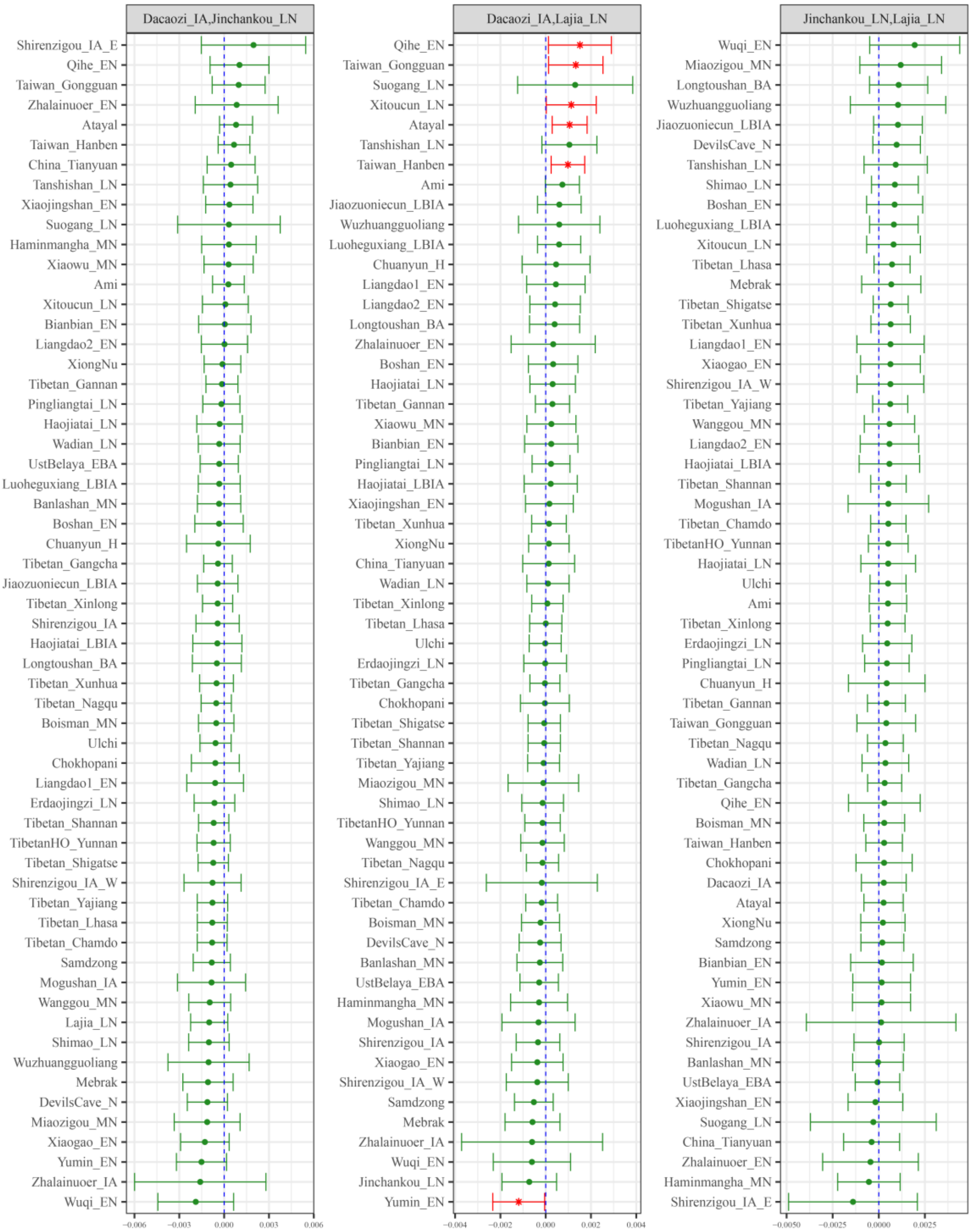

f_4(Inland Neolithic northern East Asian1, Inland Neolithic northern East Asian2; Eastern Modern Tibetan/Ancient East Asians, Mbuti)

Figure S36. Shared ancestry associated with inland Neolithic northern East Asian from Qinghai province in modern Tibetans and ancient East Asians compared with geographically different ancient populations inferred from four population symmetry-f_4-statistics of the form *f_4(Inland Neolithic northern East Asian1, Inland Neolithic northern East Asian2; Eastern Modern Tibetan/Ancient East Asians, Mbuti)*.

Here, overlapping SNP loci included in the Affymetrix Human Origins platform among four analyzed populations were used. We used the genetic variation of Mbuti as the outgroup. Red asterisk point meant the significant value (Absolute value of Z-scores larger than three or equal to three) observed in the symmetry-f_4 statistics and green circle point denoted the non-significant f_4-statistic values (Absolute value of Z-scores less than three). All Eastern Modern Tibetan/Ancient East Asians were listed along the Y-axis and f_4 values were labeled along the X-axis. All tested population pairs were faceted or grouped via the combination of the first and second populations. Significant negative f_4 values indicated that the

third population shared more alleles with the second population and significant positive f_4 value indicated that the third population shared more derived alleles with the first population. The value of f_4-statistics equal to zero was marked as the blue dash line. The bar indicated three standard errors.

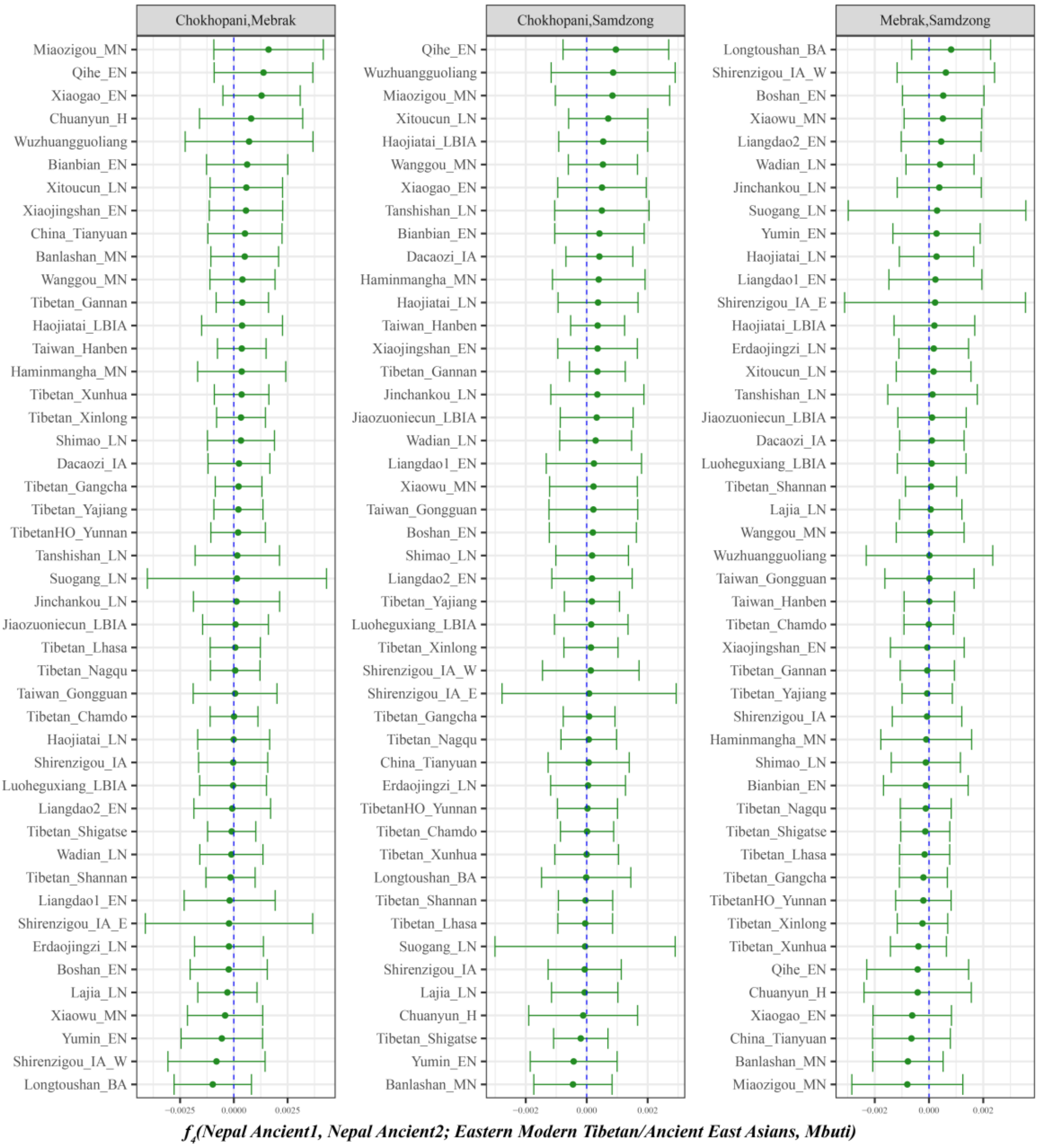

Figure S37. Shared ancestry associated with Bronze Age to historic period from Nepal in modern Tibetans and ancient East Asians compared with geographically different ancient populations inferred from four population symmetry-f_4-statistics of the form f_4*(Nepal Ancient1, Nepal Ancient2; Eastern Modern Tibetan/Ancient East Asians, Mbuti)*.

Here, overlapping SNP loci included in the Affymetrix Human Origins platform among four analyzed populations were used. We used the genetic variation of Mbuti as the outgroup. Red asterisk point meant the significant value (Absolute value of Z-scores larger than three or equal to three) observed in the symmetry-f_4 statistics and green circle point denoted the non-significant f_4-statistic values (Absolute value of Z-scores less than three). All Eastern Modern Tibetan/Ancient East Asians were listed along the Y-axis and f_4 values were labeled along the X-axis. All tested population pairs were faceted or grouped via the combination of the first and second populations. Significant negative f_4 values indicated that the third population shared more alleles with the second population and significant positive f_4 value indicated that the third population shared more derived alleles with the first population. The value of f_4-statistics equal to zero was marked as the blue dash line. The bar indicated three standard errors.

Similarities and differences of the shared genetic profiles related to northern Neolithic East Asians via the spatial comparison analysis in modern Tibetans and all available ancient East Asians.

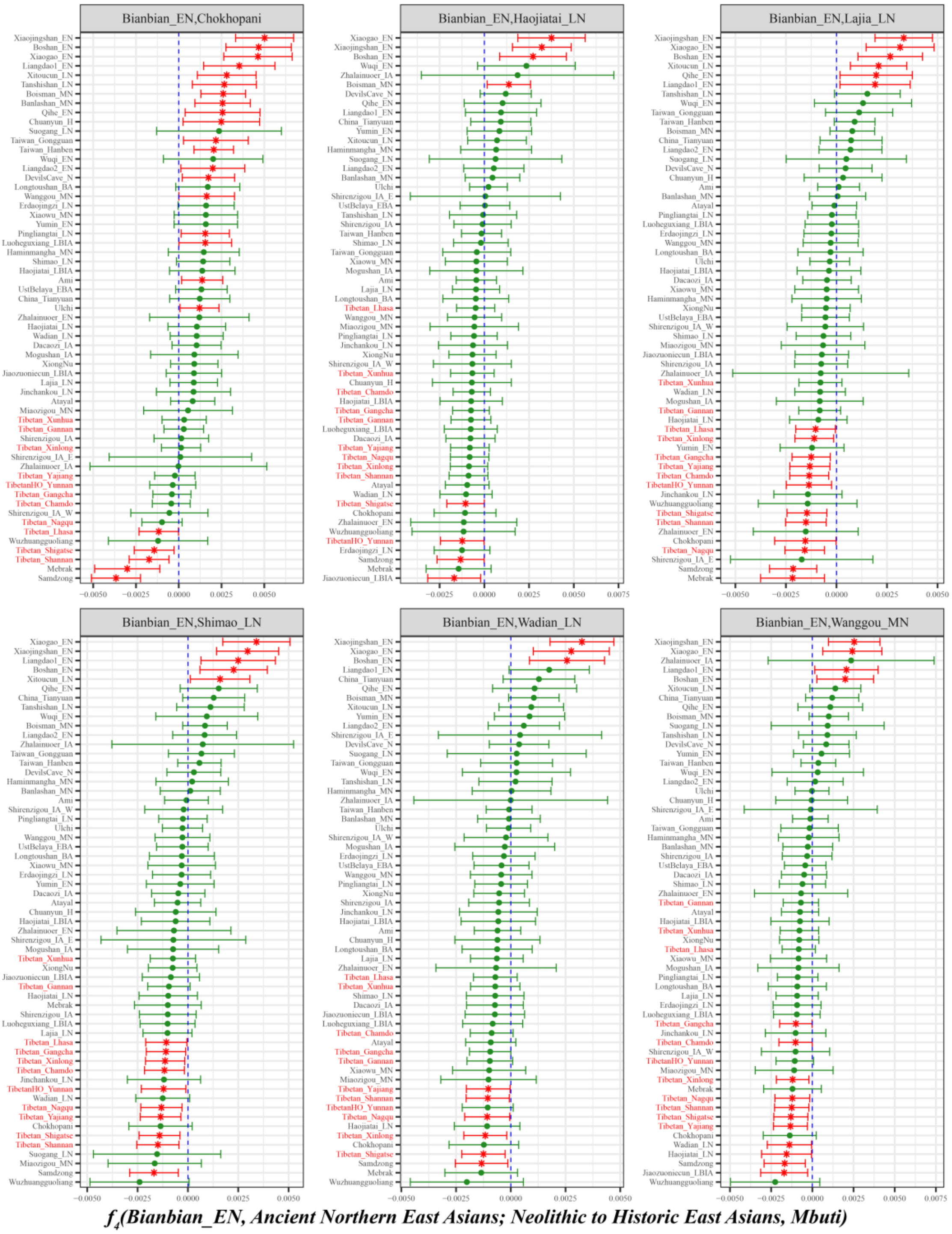

Figure S38. Spatial comparison analysis showed the shared ancestry related to early northern Neolithic East Asians in modern Tibetans and all available ancient East Asians inferred from four population symmetry-f_4-statistics of the form f_4*(Bianbian_EN, Ancient Northern East Asians; Modern Tibetan/Neolithic to Historic East Asians, Mbuti)*.

Here, overlapping SNP loci included in the Affymetrix Human Origins platform among four analyzed populations were used. We used the genetic variation of Mbuti as the outgroup. Red asterisk point meant the significant value (Absolute value of Z-scores larger than three or equal to three) observed in the symmetry-f_4 statistics and green circle point denoted the non-significant f_4-statistic values (Absolute value of Z-scores less than three). All Eastern Modern Tibetan/Ancient East Asians were listed along the

Y-axis and f_4 values were labeled along the X-axis. All tested population pairs were faceted or grouped via the combination of the first and second populations. Significant negative f_4 values indicated that the third population shared more alleles with the second population and significant positive f_4 value indicated that the third population shared more derived alleles with the first population. The value of f_4-statistics equal to zero was marked as the blue dash line. The bar indicated three standard errors.

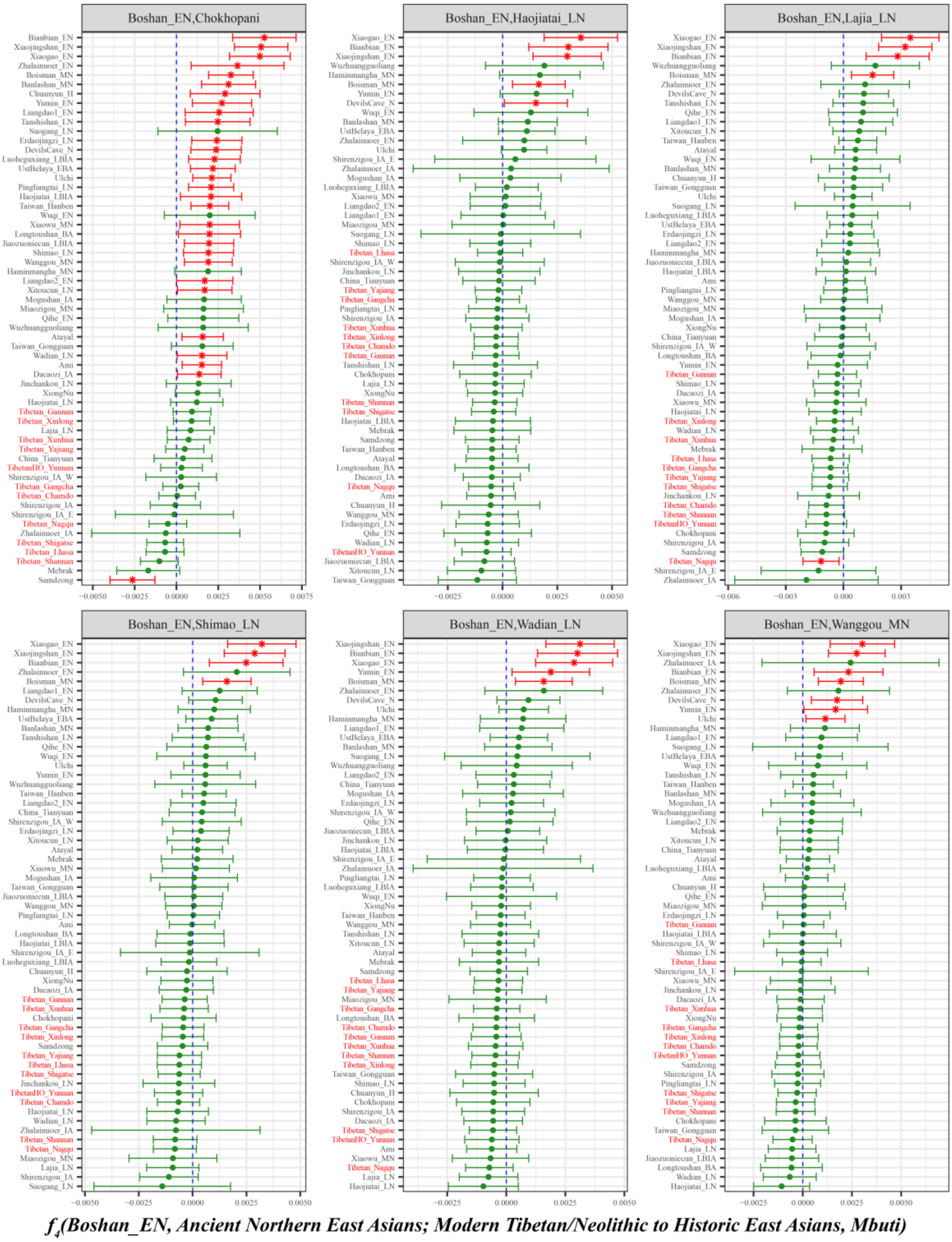

Figure S39. Spatial comparison analysis showed the shared ancestry related to early northern Neolithic East Asians in modern Tibetans and all available ancient East Asians inferred from four population symmetry-f_4-statistics of the form *f_4(Boshan_EN, Ancient Northern East Asians; Modern Tibetan/Neolithic to Historic East Asians, Mbuti)*.

Here, overlapping SNP loci included in the Affymetrix Human Origins platform among four analyzed populations were used. We used the genetic variation of Mbuti as the outgroup. Red asterisk point meant

the significant value (Absolute value of Z-scores larger than three or equal to three) observed in the symmetry-f_4 statistics and green circle point denoted the non-significant f_4-statistic values (Absolute value of Z-scores less than three). All Eastern Modern Tibetan/Ancient East Asians were listed along the Y-axis and f_4 values were labeled along the X-axis. All tested population pairs were faceted or grouped via the combination of the first and second populations. Significant negative f_4 values indicated that the third population shared more alleles with the second population and significant positive f_4 value indicated that the third population shared more derived alleles with the first population. The value of f_4-statistics equal to zero was marked as the blue dash line. The bar indicated three standard errors.

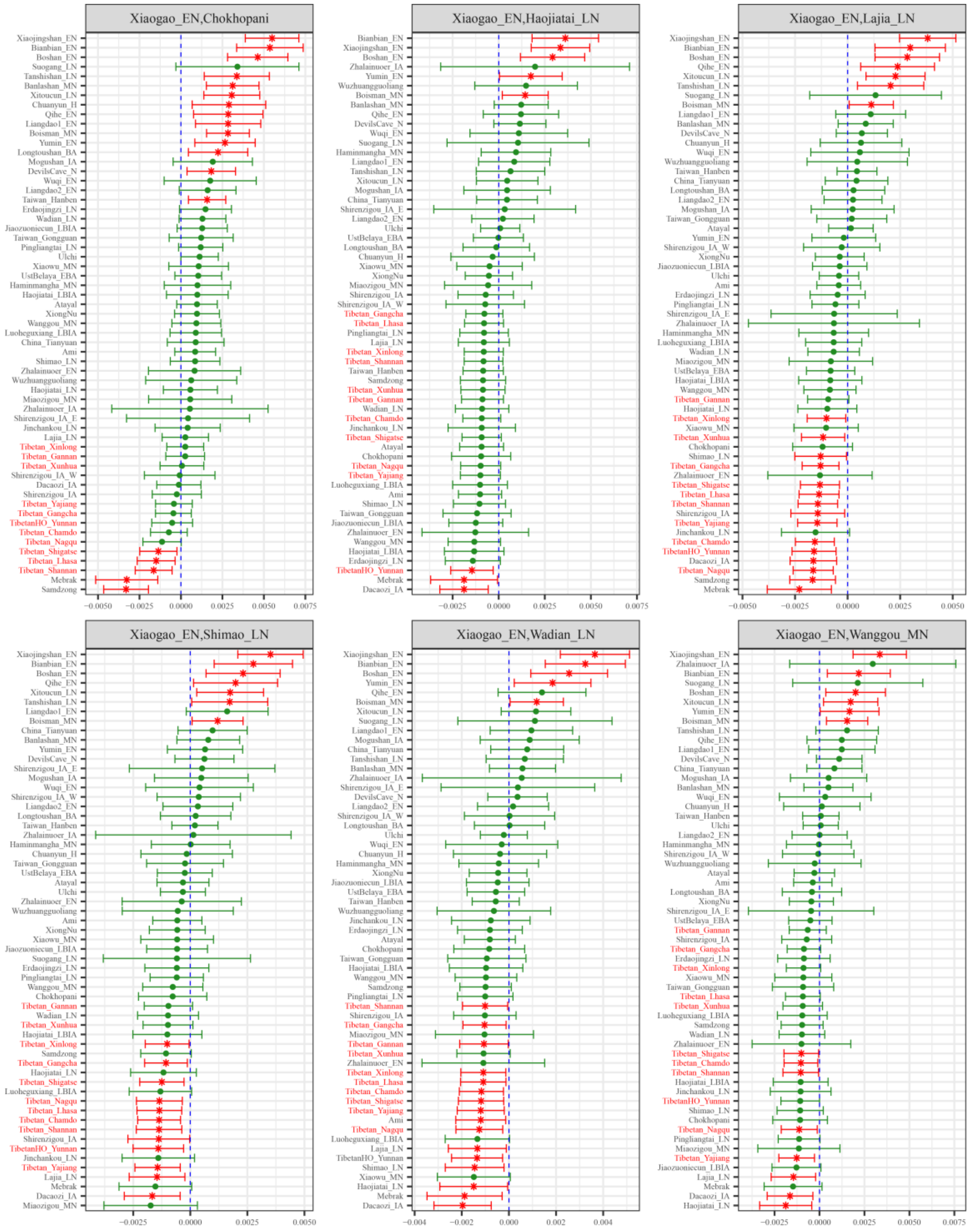

f_4(Xiaogao_EN, Ancient Northern East Asians; Modern Tibetan/Neolithic to Historic East Asians, Mbuti)

Figure S40. Spatial comparison analysis showed the shared ancestry related to early northern Neolithic East Asians in modern Tibetans and all available ancient East Asians inferred from four population symmetry-f_4-statistics of the form f_4*(Xiaogao_EN, Ancient Northern East Asians; Modern Tibetan/Neolithic to Historic East Asians, Mbuti)*.

Here, overlapping SNP loci included in the Affymetrix Human Origins platform among four analyzed populations were used. We used the genetic variation of Mbuti as the outgroup. Red asterisk point meant the significant value (Absolute value of Z-scores larger than three or equal to three) observed in the symmetry-f_4 statistics and green circle point denoted the non-significant f_4-statistic values (Absolute value of Z-scores less than three). All Eastern Modern Tibetan/Ancient East Asians were listed along the Y-axis and f_4 values were labeled along the X-axis. All tested population pairs were faceted or grouped via the combination of the first and second populations. Significant negative f_4 values indicated that the third population shared more alleles with the second population and significant positive f_4 value indicated that the third population shared more derived alleles with the first population. The value of f_4-statistics equal to zero was marked as the blue dash line. The bar indicated three standard errors.

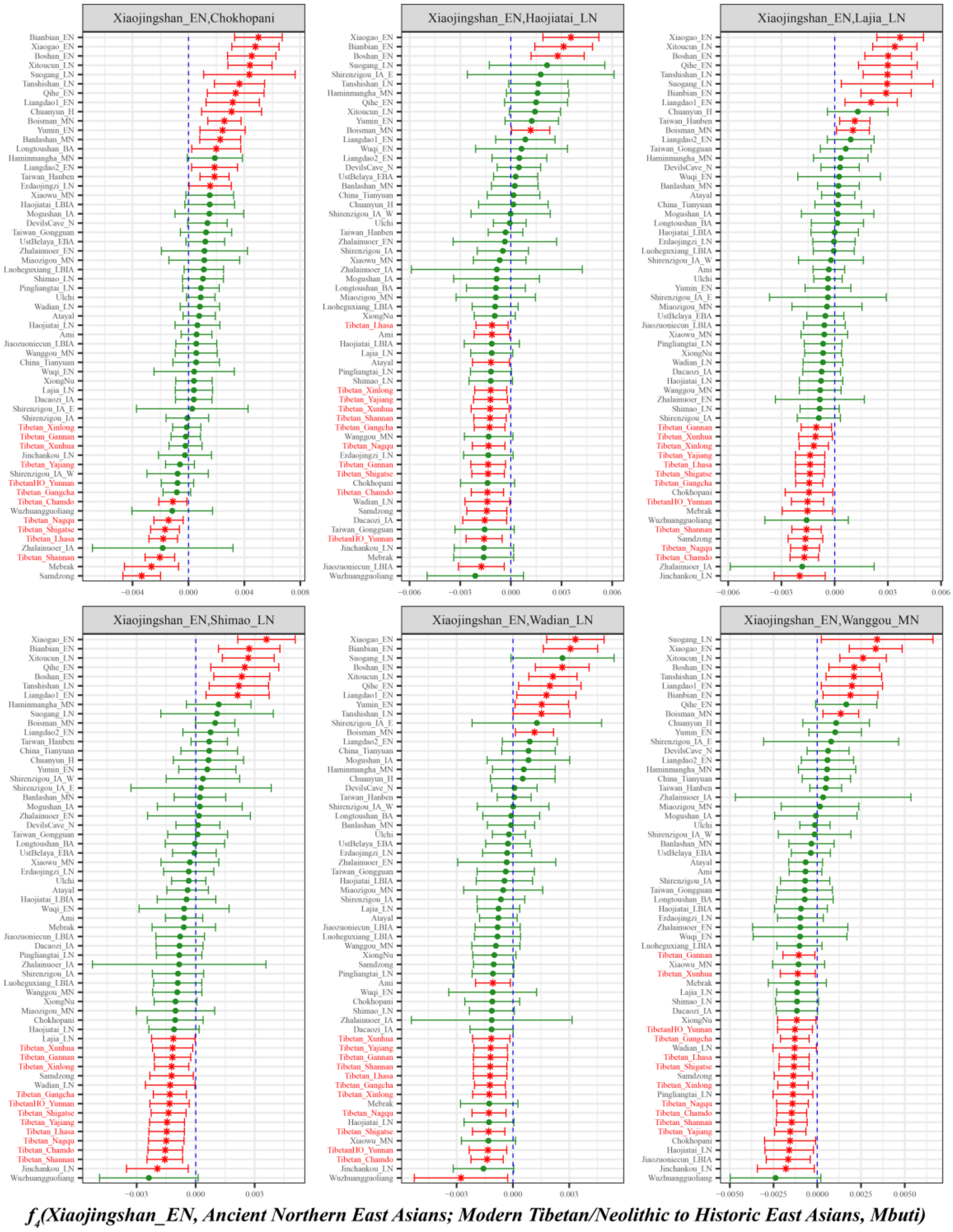

Figure S41. Spatial comparison analysis showed the shared ancestry related to early northern Neolithic East Asians in modern Tibetans and all available ancient East Asians inferred from four population symmetry-f_4-statistics of the form f_4*(Xiaojingshan_EN, Ancient Northern East Asians; Modern Tibetan/Neolithic to Historic East Asians, Mbuti)*.

Here, overlapping SNP loci included in the Affymetrix Human Origins platform among four analyzed populations were used. We used the genetic variation of Mbuti as the outgroup. Red asterisk point meant the significant value (Absolute value of Z-scores larger than three or equal to three) observed in the symmetry-f_4 statistics and green circle point denoted the non-significant f_4-statistic values (Absolute value of Z-scores less than three). All Eastern Modern Tibetan/Ancient East Asians were listed along the Y-axis and f_4 values were labeled along the X-axis. All tested population pairs were faceted or grouped via the combination of the first and second populations. Significant negative f_4 values indicated that the third population shared more alleles with the second population and significant positive f_4 value indicated

that the third population shared more derived alleles with the first population. The value of f_4-statistics equal to zero was marked as the blue dash line. The bar indicated three standard errors.

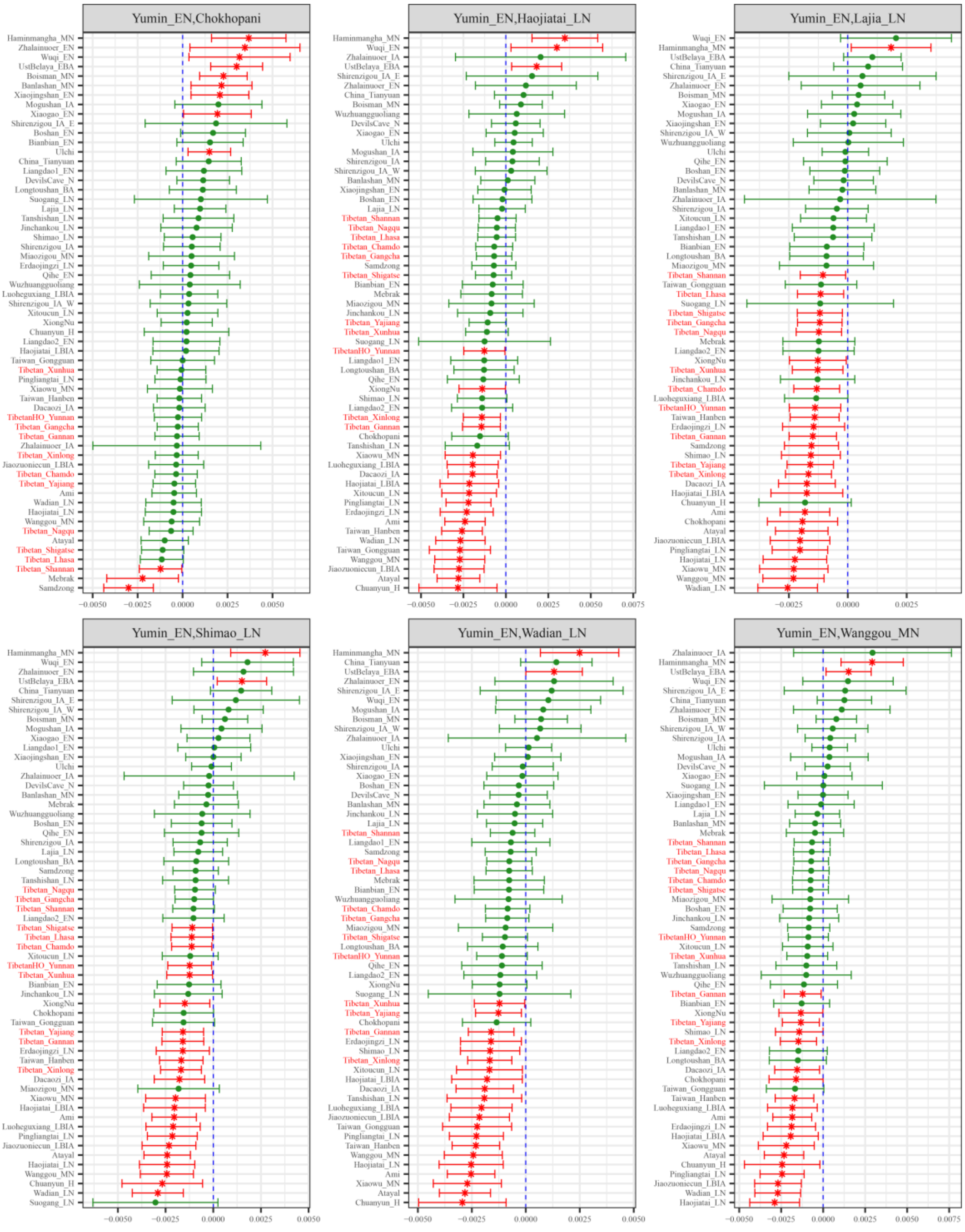

f_4(Yumin_EN, Ancient Northern East Asians; Modern Tibetan/Neolithic to Historic East Asians, Mbuti)

Figure S42. Spatial comparison analysis showed the shared ancestry related to early northern Neolithic East Asians in modern Tibetans and all available ancient East Asians inferred from four population symmetry-f_4-statistics of the form f_4*(Yumin_EN, Ancient Northern East Asians; Modern Tibetan/Neolithic to Historic East Asians, Mbuti)*.

Here, overlapping SNP loci included in the Affymetrix Human Origins platform among four analyzed populations were used. We used the genetic variation of Mbuti as the outgroup. Red asterisk point meant the significant value (Absolute value of Z-scores larger than three or equal to three) observed in the symmetry-f_4 statistics and green circle point denoted the non-significant f_4-statistic values (Absolute value of Z-scores less than three). All Eastern Modern Tibetan/Ancient East Asians were listed along the

Y-axis and f_4 values were labeled along the X-axis. All tested population pairs were faceted or grouped via the combination of the first and second populations. Significant negative f_4 values indicated that the third population shared more alleles with the second population and significant positive f_4 value indicated that the third population shared more derived alleles with the first population. The value of f_4-statistics equal to zero was marked as the blue dash line. The bar indicated three standard errors.

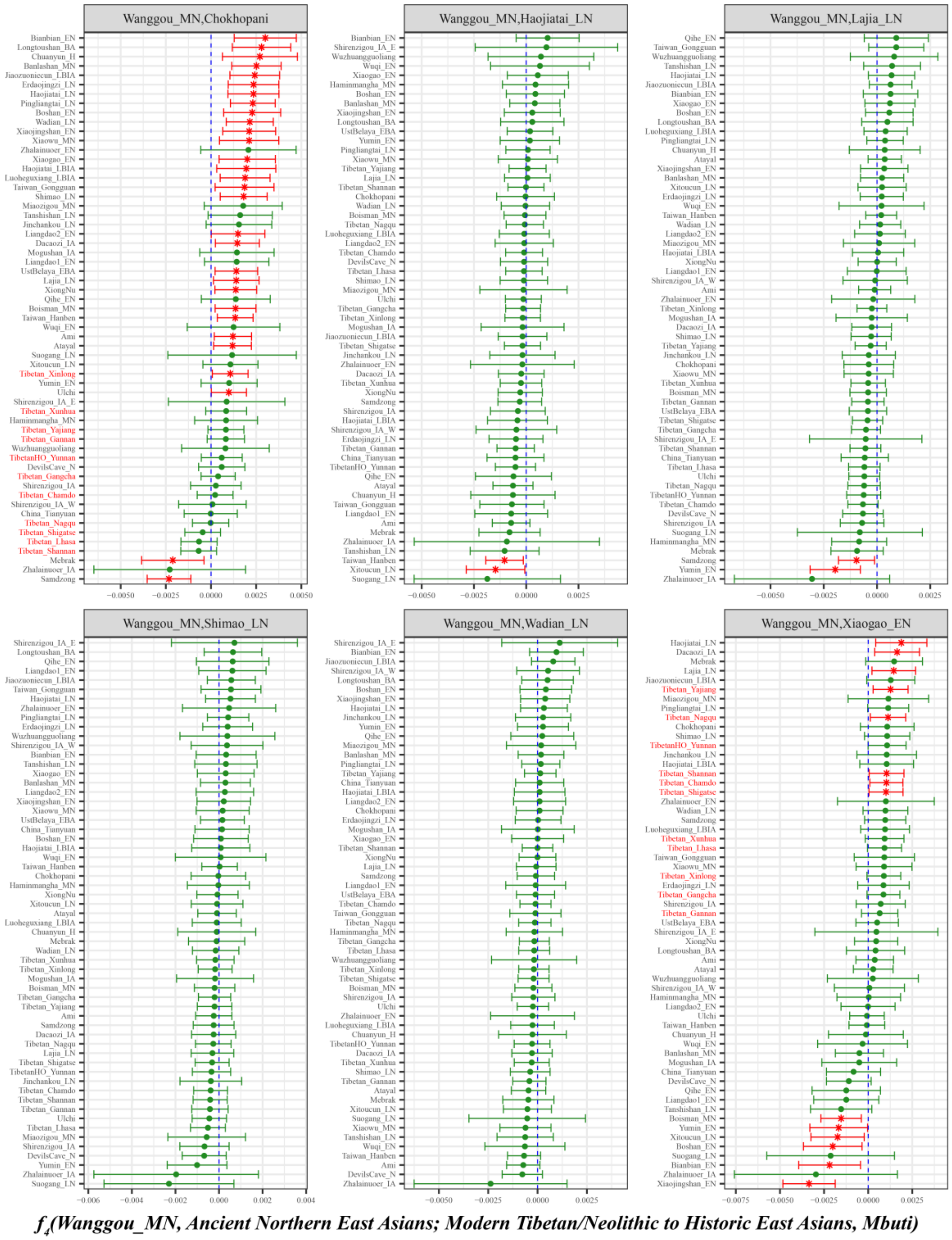

Figure S43. Spatial comparison analysis showed the shared ancestry related to middle northern Neolithic East Asians in modern Tibetans and all available ancient East Asians inferred from four population symmetry-f_4-statistics of the form *f_4(Wanggou_MN, Ancient Northern East Asians; Modern Tibetan/Neolithic to Historic East Asians, Mbuti)*.

Here, overlapping SNP loci included in the Affymetrix Human Origins platform among four analyzed populations were used. We used the genetic variation of Mbuti as the outgroup. Red asterisk point meant

the significant value (Absolute value of Z-scores larger than three or equal to three) observed in the symmetry-f_4 statistics and green circle point denoted the non-significant f_4-statistic values (Absolute value of Z-scores less than three). All Eastern Modern Tibetan/Ancient East Asians were listed along the Y-axis and f_4 values were labeled along the X-axis. All tested population pairs were faceted or grouped via the combination of the first and second populations. Significant negative f_4 values indicated that the third population shared more alleles with the second population and significant positive f_4 value indicated that the third population shared more derived alleles with the first population. The value of f_4-statistics equal to zero was marked as the blue dash line. The bar indicated three standard errors.

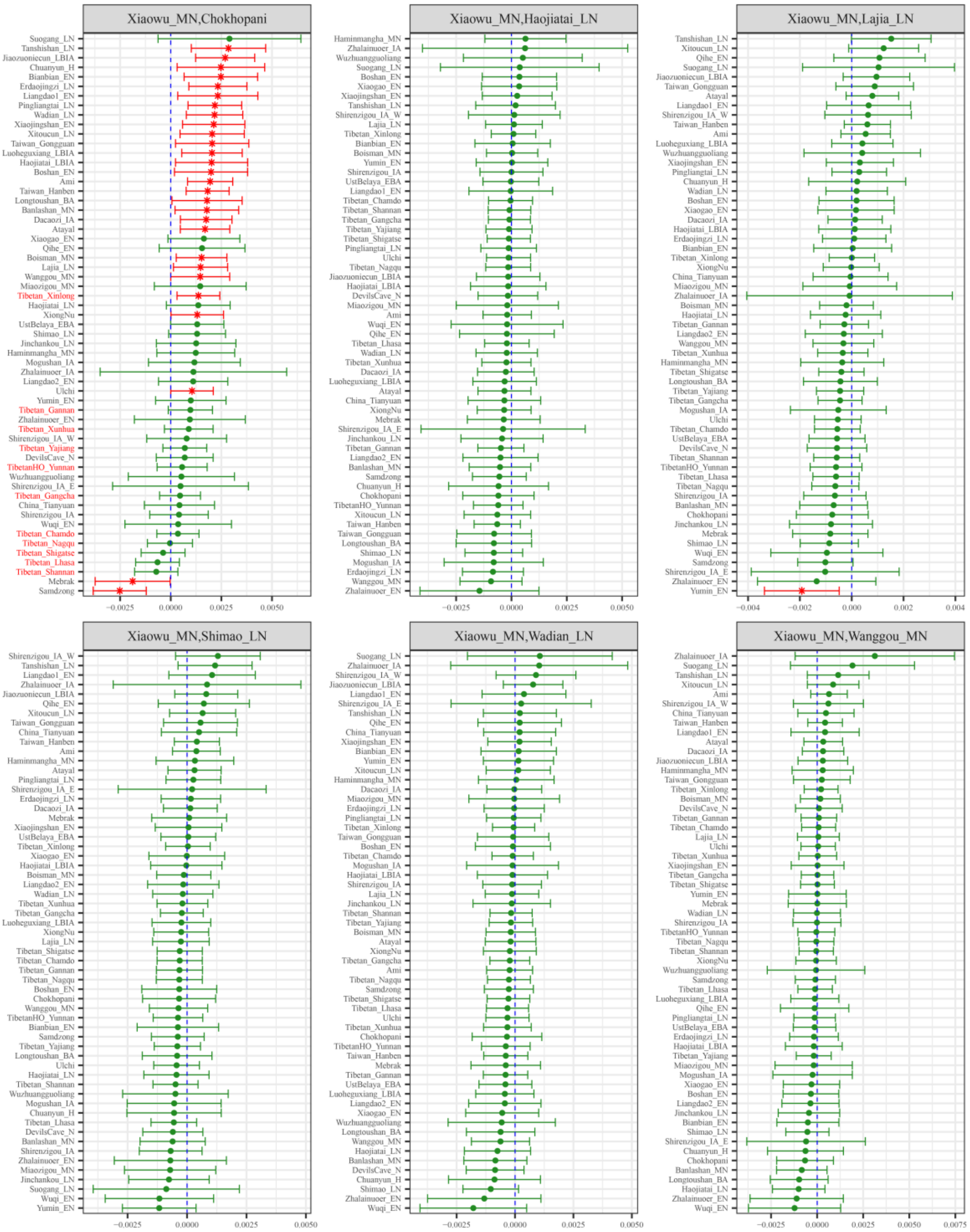

f_4(Xiaowu_MN, Ancient Northern East Asians; Modern Tibetan/Neolithic to Historic East Asians, Mbuti)

Figure S44. Spatial comparison analysis showed the shared ancestry related to middle northern Neolithic East Asians in modern Tibetans and all available ancient East Asians inferred from four population symmetry-f_4-statistics of the form *f_4(Xiaowu_MN, Ancient Northern East Asians; Modern Tibetan/Neolithic to Historic East Asians, Mbuti)*.

Here, overlapping SNP loci included in the Affymetrix Human Origins platform among four analyzed populations were used. We used the genetic variation of Mbuti as the outgroup. Red asterisk point meant the significant value (Absolute value of Z-scores larger than three or equal to three) observed in the symmetry-f_4 statistics and green circle point denoted the non-significant f_4-statistic values (Absolute value of Z-scores less than three). All Eastern Modern Tibetan/Ancient East Asians were listed along the Y-axis and f_4 values were labeled along the X-axis. All tested population pairs were faceted or grouped via the combination of the first and second populations. Significant negative f_4 values indicated that the third population shared more alleles with the second population and significant positive f_4 value indicated that the third population shared more derived alleles with the first population. The value of f_4-statistics equal to zero was marked as the blue dash line. The bar indicated three standard errors.

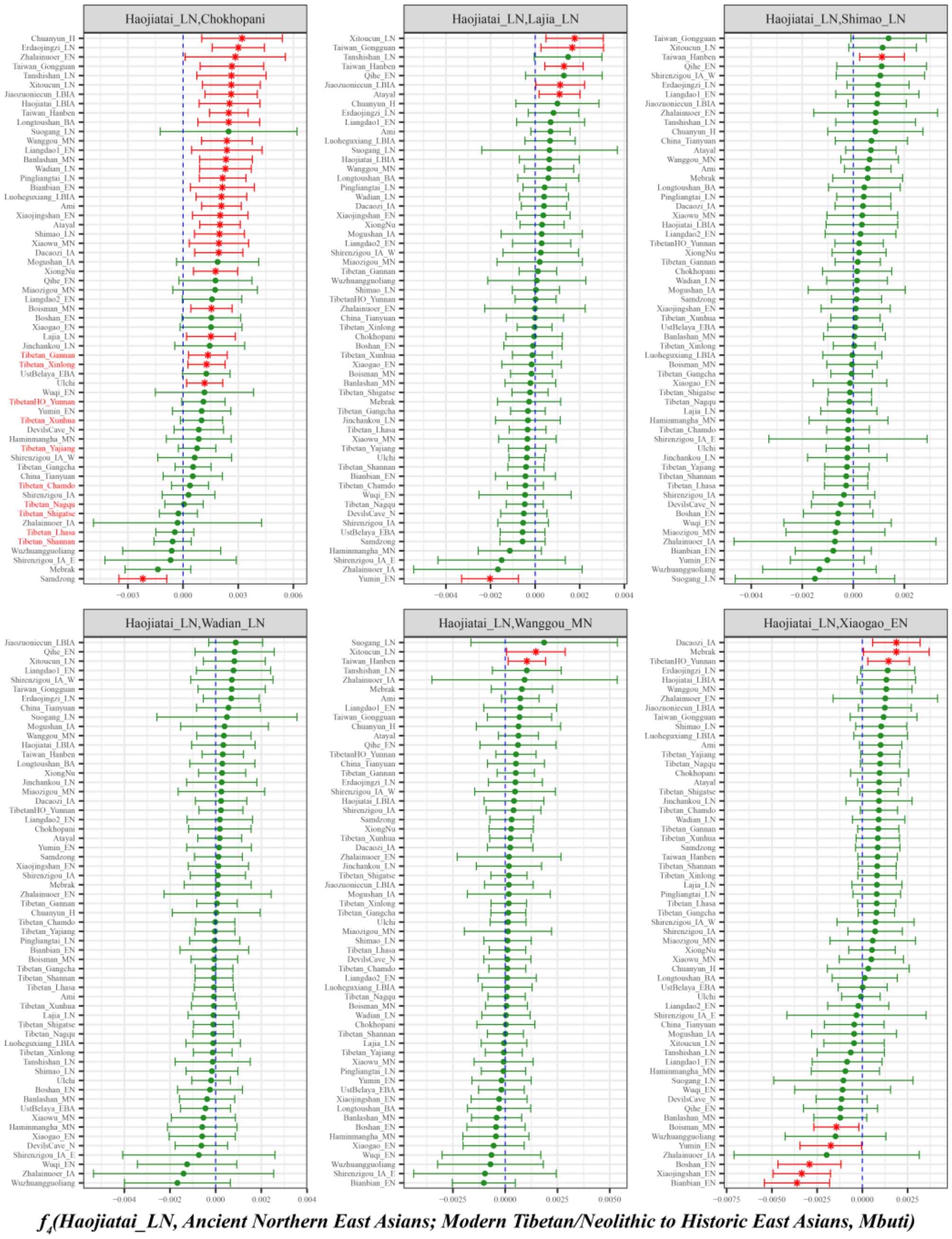

Figure S45. Spatial comparison analysis showed the shared ancestry related to late northern Neolithic East Asians in modern Tibetans and all available ancient East Asians inferred from four population symmetry-f_4-statistics of the form *f_4(Haojiatai_LN, Ancient Northern East Asians; Modern Tibetan/Neolithic to Historic East Asians, Mbuti)*.

Here, overlapping SNP loci included in the Affymetrix Human Origins platform among four analyzed populations were used. We used the genetic variation of Mbuti as the outgroup. Red asterisk point meant the significant value (Absolute value of Z-scores larger than three or equal to three) observed in the symmetry-f_4 statistics and green circle point denoted the non-significant f_4-statistic values (Absolute value of Z-scores less than three). All Eastern Modern Tibetan/Ancient East Asians were listed along the Y-axis and f_4 values were labeled along the X-axis. All tested population pairs were faceted or grouped via the combination of the first and second populations. Significant negative f_4 values indicated that the third population shared more alleles with the second population and significant positive f_4 value indicated that the third population shared more derived alleles with the first population. The value of f_4-statistics equal to zero was marked as the blue dash line. The bar indicated three standard errors.

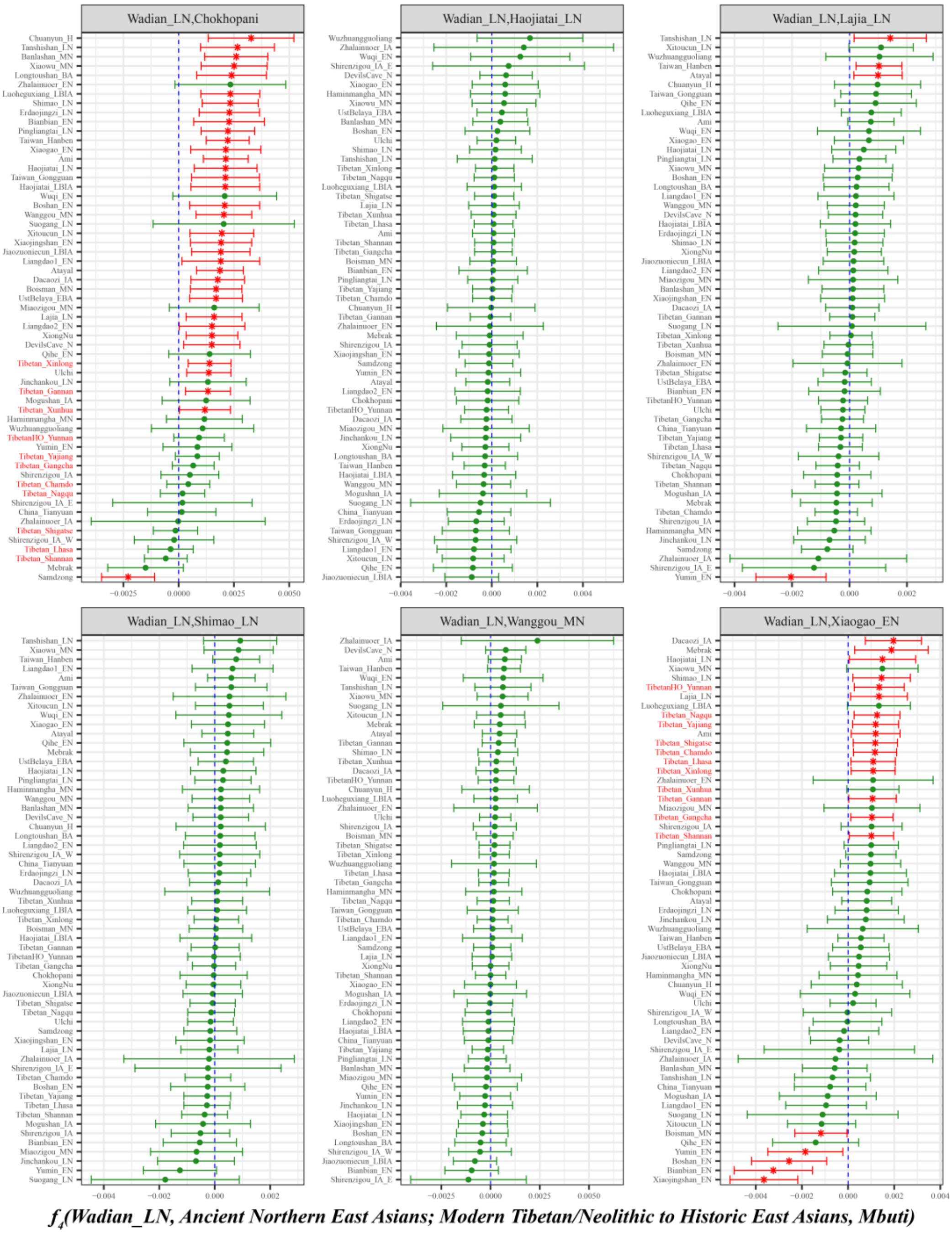

Figure S46. Spatial comparison analysis showed the shared ancestry related to late northern Neolithic East Asians in modern Tibetans and all available ancient East Asians inferred from four population symmetry-f_4-statistics of the form f_4*(Wadian_LN, Ancient Northern East Asians; Modern Tibetan/Neolithic to Historic East Asians, Mbuti)*.

Here, overlapping SNP loci included in the Affymetrix Human Origins platform among four analyzed populations were used. We used the genetic variation of Mbuti as the outgroup. Red asterisk point meant the significant value (Absolute value of Z-scores larger than three or equal to three) observed in the symmetry-f_4 statistics and green circle point denoted the non-significant f_4-statistic values (Absolute value of Z-scores less than three). All Eastern Modern Tibetan/Ancient East Asians were listed along the Y-axis and f_4 values were labeled along the X-axis. All tested population pairs were faceted or grouped via the combination of the first and second populations. Significant negative f_4 values indicated that the third population shared more alleles with the second population and significant positive f_4 value indicated that the third population shared more derived alleles with the first population. The value of f_4-statistics equal to zero was marked as the blue dash line. The bar indicated three standard errors.

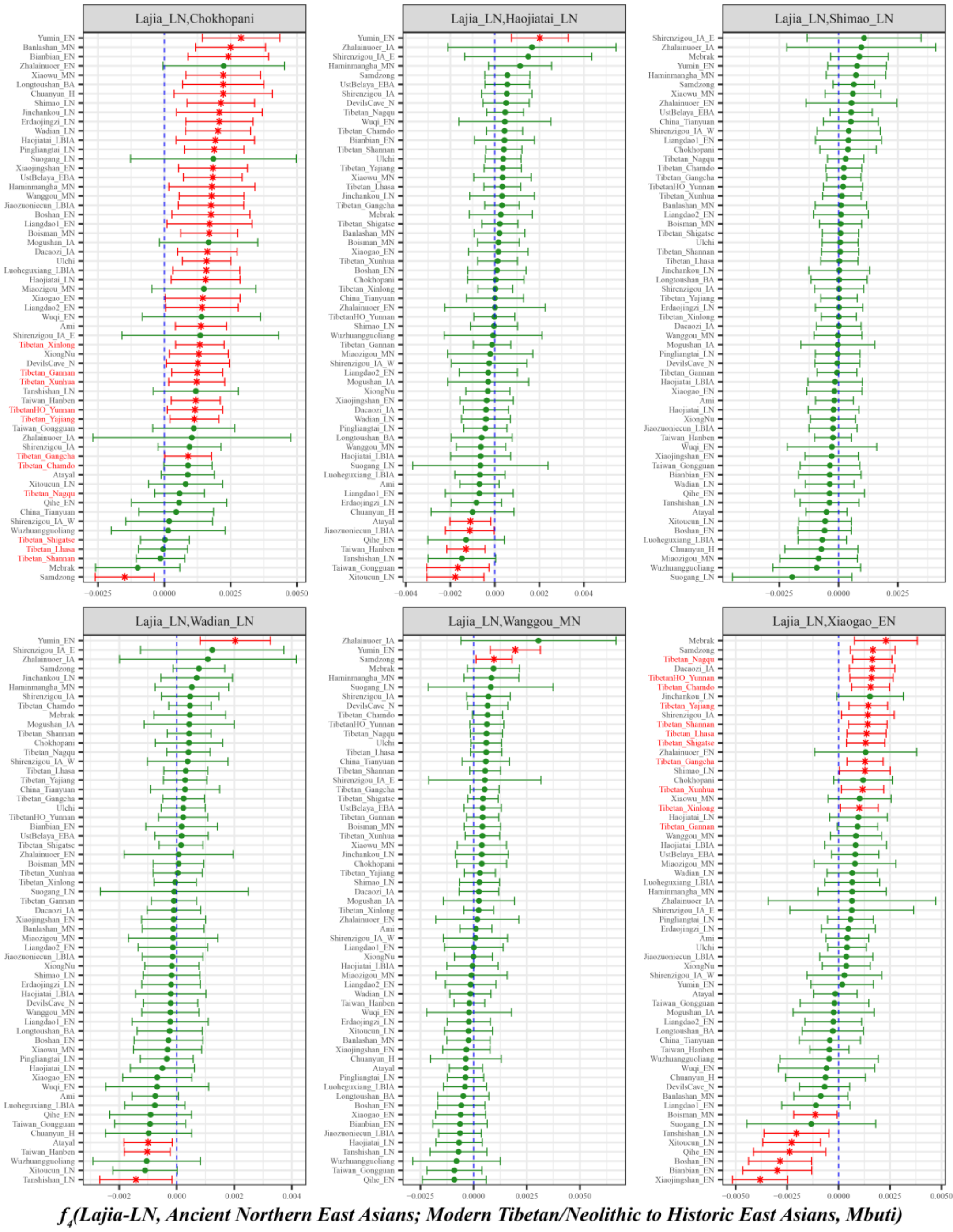

Figure S47. Spatial comparison analysis showed the shared ancestry related to late northern Neolithic East Asians in modern Tibetans and all available ancient East Asians inferred from four population symmetry-f_4-statistics of the form f_4*(Lajia_LN, Ancient Northern East Asians; Modern Tibetan/Neolithic to Historic East Asians, Mbuti)*.
Here, overlapping SNP loci included in the Affymetrix Human Origins platform among four analyzed populations were used. We used the genetic variation of Mbuti as the outgroup. Red asterisk point meant the significant value (Absolute value of Z-scores larger than three or equal to three) observed in the symmetry-f_4 statistics and green circle point denoted the non-significant f_4-statistic values (Absolute value of Z-scores less than three). All Eastern Modern Tibetan/Ancient East Asians were listed along the Y-axis and f_4 values were labeled along the X-axis. All tested population pairs were faceted or grouped via the combination of the first and second populations. Significant negative f_4 values indicated that the third population shared more alleles with the second population and significant positive f_4 value indicated that the third population shared more derived alleles with the first population. The value of f_4-statistics equal to zero was marked as the blue dash line. The bar indicated three standard errors.

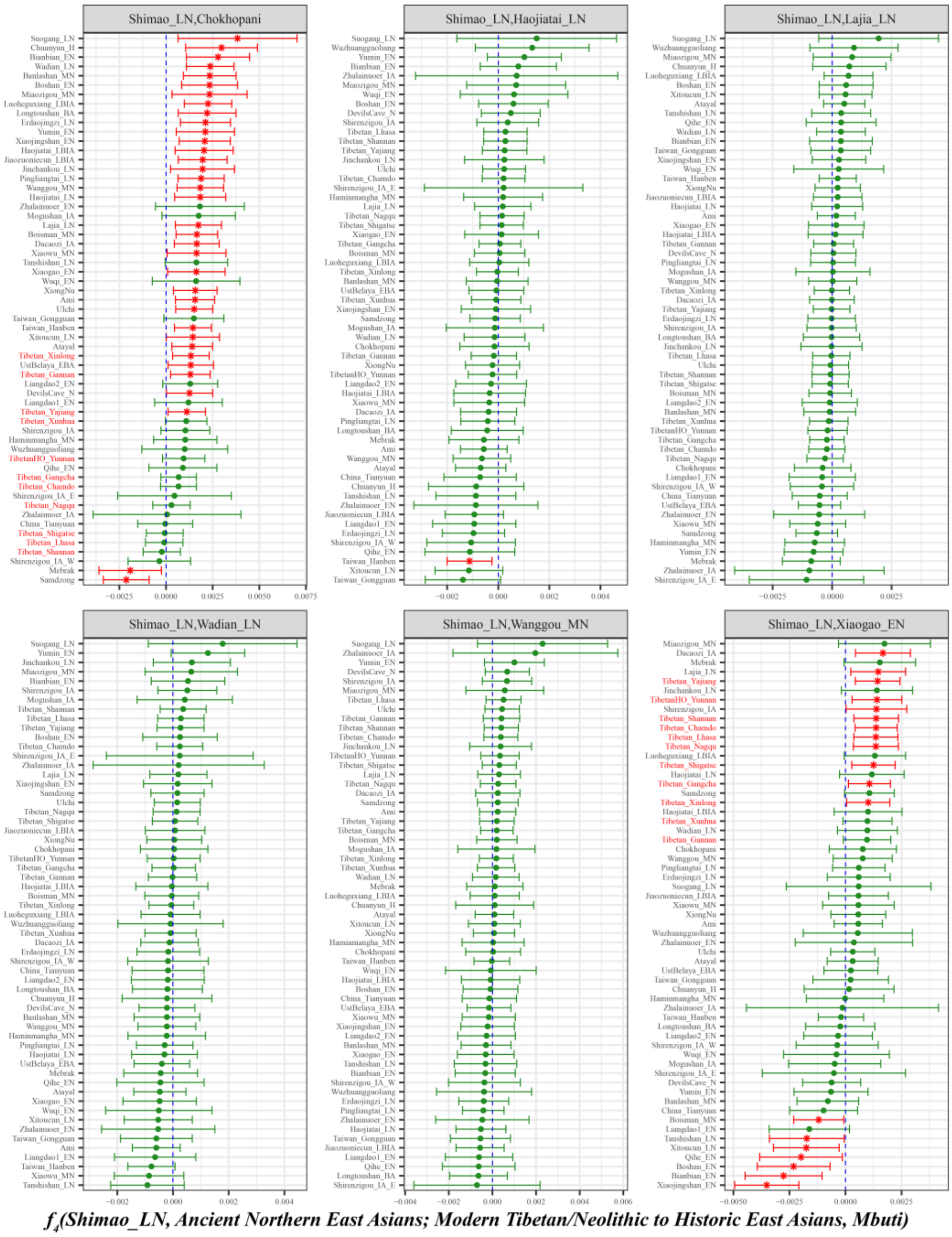

Figure S48. Spatial comparison analysis showed the shared ancestry related to late northern Neolithic East Asians in modern Tibetans and all available ancient East Asians inferred from four population symmetry-f_4-statistics of the form f_4*(Shimao_LN, Ancient Northern East Asians; Modern Tibetan/Neolithic to Historic East Asians, Mbuti)*.

Here, overlapping SNP loci included in the Affymetrix Human Origins platform among four analyzed populations were used. We used the genetic variation of Mbuti as the outgroup. Red asterisk point meant the significant value (Absolute value of Z-scores larger than three or equal to three) observed in the symmetry-f_4 statistics and green circle point denoted the non-significant f_4-statistic values (Absolute value of Z-scores less than three). All Eastern Modern Tibetan/Ancient East Asians were listed along the Y-axis and f_4 values were labeled along the X-axis. All tested population pairs were faceted or grouped via the combination of the first and second populations. Significant negative f_4 values indicated that the third population shared more alleles with the second population and significant positive f_4 value indicated that the third population shared more derived alleles with the first population. The value of f_4-statistics equal to zero was marked as the blue dash line. The bar indicated three standard errors.

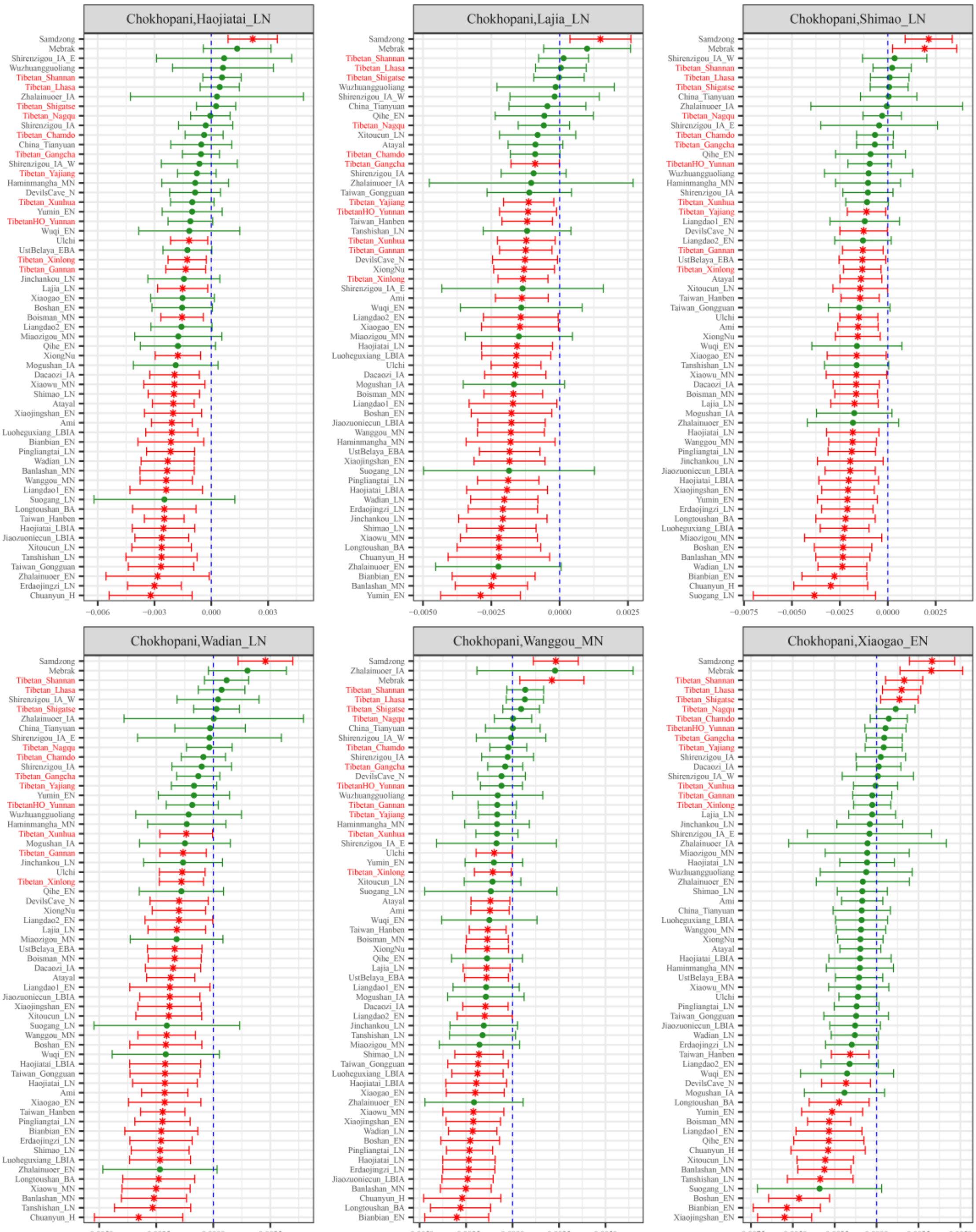

f_4(Chokhopani, Ancient Northern East Asians; Modern Tibetan/Neolithic to Historic East Asians, Mbuti)

Figure S49. Spatial comparison analysis showed the shared ancestry related to the Ancient Nepal population in modern Tibetans and all available ancient East Asians inferred from four population symmetry-*f_4*-statistics of the form *f_4(Chokhopani, Ancient Northern East Asians; Modern Tibetan/Neolithic to Historic East Asians, Mbuti)*.

Here, overlapping SNP loci included in the Affymetrix Human Origins platform among four analyzed populations were used. We used the genetic variation of Mbuti as the outgroup. Red asterisk point meant the significant value (Absolute value of Z-scores larger than three or equal to three) observed in the symmetry-f_4 statistics and green circle point denoted the non-significant f_4-statistic values (Absolute value of Z-scores less than three). All Eastern Modern Tibetan/Ancient East Asians were listed along the Y-axis and f_4 values were labeled along the X-axis. All tested population pairs were faceted or grouped via the combination of the first and second populations. Significant negative f_4 values indicated that the third population shared more alleles with the second population and significant positive f_4 value indicated that the third population shared more derived alleles with the first population. The value of f_4-statistics equal to zero was marked as the blue dash line. The bar indicated three standard errors.
