# Supplementary material for: Peopling History of the Tibetan Plateau and Multiple Waves of Admixture of Tibetans Inferred From Both Ancient and Modern Genome-Wide Data
Source: Front Genet. 2021 Sep 3;12:725243. doi: 10.3389/fgene.2021.725243 (PMC8506211; doi:10.3389/fgene.2021.725243)
Supplement: Supplementary file 3 [file Data_Sheet_3.PDF]

Supplementary Figures S50-S80

Peopling history of Tibet Plateau and multiple waves of admixture of Tibetans inferred from both ancient and modern genome-wide data

Guanglin He[1,2,*,#], Mengge Wang[3,*], Xing Zou[3,*], Pengyu Chen[4], Zheng Wang[2], Yan Liu[5], Hongbin Yao[6], Lan-Hai Wei[2], Renkuan Tang[7,#], Chuan-Chao Wang[2,#], Hui-Yuan Yeh[1,#]

[1]School of Humanities, Nanyang Technological University, Nanyang, 639798, Singapore
[2]Department of Anthropology and Ethnology, Institute of Anthropology, National Institute for Data Science in Health and Medicine, and School of Life Sciences, Xiamen University, Xiamen, 361005, China
[3]Institute of Forensic Medicine, West China School of Basic Science and Forensic Medicine, Sichuan University, Chengdu, 610041, China
[4]Center of Forensic Expertise, Affiliated hospital of Zunyi Medical University, Zunyi, Guizhou, 563099, China
[5]School of Basic Medical Sciences, North Sichuan Medical College, Nanchong, Sichuan, 637100, China
[6]Key Laboratory of Evidence Science of Gansu Province, Gansu Institute of Political Science and Law, Lanzhou, 730070, China
[7]Department of Forensic Medicine, College of Basic Medicine, Chongqing Medical University, Chongqing, 400016, China
*These authors contributed equally to this work and should be considered co-first authors.
[#]Corresponding author
Guanglin He
Affiliation: School of Humanities, Nanyang Technological University, Nanyang, 639798, Singapore; Department of Anthropology and Ethnology, Institute of Anthropology, National Institute for Data Science in Health and Medicine, Xiamen University, Xiamen, China.
E-mail: Guanglinhescu@163.com
Renkuan Tang
Department of Forensic Medicine, College of Basic Medicine, Chongqing Medical University, Chongqing, China
E-mail: renktang2012@163.com
Chuan-Chao Wang
Affiliation: Department of Anthropology and Ethnology, Institute of Anthropology, National Institute for Data Science in Health and Medicine, Xiamen University, Xiamen, China.
E-mail: wang@xmu.edu.cn
Hui-Yuan Yeh
Affiliation: School of Humanities, Nanyang Technological University, Nanyang, 639798, Singapore
E-mail: hyyeh@ntu.edu.sg

Contents of Supplementary Figures S50-S107

Additional gene flow events when we assumed that Tibetans' direct ancestor is Yangtze rice farmer related ancestral populations 1

Figure S50. Results of affinity-f_4 statistics for the form *f_4(Liaodao1_EN, Modern Tibetan; Neolithic to Historic East Asians, Mbuti)* showed the additional shared derived alleles from source populations except for Yangtze rice farmer related ancestral populations. 1
Figure S51. Results of affinity-f_4 statistics for the form *f_4(Liaodao2_EN, Modern Tibetan; Neolithic to Historic East Asians, Mbuti)* showed the additional shared derived alleles from source populations except for Yangtze rice farmer related ancestral populations. 2
Figure S52. Results of affinity-f_4 statistics for the form *f_4(Qihe_EN, Modern Tibetan; Neolithic to Historic East Asians, Mbuti)* showed the additional shared derived alleles from source populations except for Yangtze rice farmer related ancestral populations. 3
Figure S53. Results of affinity-f_4 statistics for the form *f_4(Xitoucun_LN, Modern Tibetan; Neolithic to Historic East Asians, Mbuti)* showed the additional shared derived alleles from source populations except for Yangtze rice farmer related ancestral populations. 4
Figure S54. Results of affinity-f_4 statistics for the form *f_4(Tanshishan_LN, Modern Tibetan; Neolithic to Historic East Asians, Mbuti)* showed the additional shared derived alleles from source populations except for Yangtze rice farmer related ancestral populations. 5
Figure S55. Results of affinity-f_4 statistics for the form *f_4(Suogang_LN, Modern Tibetan; Neolithic to Historic East Asians, Mbuti)* showed the additional shared derived alleles from source populations except for Yangtze rice farmer related ancestral populations. 6
Figure S56. Results of affinity-f_4 statistics for the form *f_4(Taiwan_Hanben, Modern Tibetan; Neolithic to Historic East Asians, Mbuti)* showed the additional shared derived alleles from source populations except for Yangtze rice farmer related ancestral populations. 7
Figure S57. Results of affinity-f_4 statistics for the form *f_4(Taiwan_Gongguan, Modern Tibetan; Neolithic to Historic East Asians, Mbuti)* showed the additional shared derived alleles from source populations except for Yangtze rice farmer related ancestral populations. 8
Figure S58. Results of affinity-f_4 statistics for the form *f_4(Ami, Modern Tibetan; Neolithic to Historic East Asians, Mbuti)* showed the additional shared derived alleles from source populations except for Yangtze rice farmer related ancestral populations. 9
Additional gene flow events when we assumed that Tibetans' direct ancestor is coastal Neolithic northern East Asian related ancestral populations 11
Figure S59. Results of affinity-f_4 statistics for the form *f_4(Bianbian_EN, Modern Tibetan; Neolithic to Historic East Asians, Mbuti)* showed the additional shared derived alleles from source populations except for coastal Neolithic northern East Asian related ancestral populations. 11
Figure S60. Results of affinity-f_4 statistics for the form *f_4(Boshan_EN, Modern Tibetan; Neolithic to Historic East Asians, Mbuti)* showed the additional shared derived alleles from source populations except for coastal Neolithic northern East Asian related ancestral populations. 12
Figure S61. Results of affinity-f_4 statistics for the form *f_4(Xiaogao_EN, Modern Tibetan; Neolithic to Historic East Asians, Mbuti)* showed the additional shared derived alleles from source populations except for coastal Neolithic northern East Asian related ancestral populations. 13
Figure S62. Results of affinity-f_4 statistics for the form *f_4(Xiaojingshan_EN, Modern Tibetan; Neolithic to Historic East Asians, Mbuti)* showed the additional shared derived alleles from source populations except for coastal Neolithic northern East Asian related ancestral populations. 14

Additional gene flow events when we assumed that Tibetans' direct ancestor is Henan Neolithic to Iron Age East Asian related ancestral populations 15

Figure S63. Results of affinity-f_4 statistics for the form f_4*(Xiaowu_LN, Modern Tibetan; Neolithic to Historic East Asians, Mbuti)* showed the additional shared derived alleles from source populations except for Henan ancient population or their related ancestral populations. 15

Figure S64. Results of affinity-f_4 statistics for the form f_4*(Wanggou_LN, Modern Tibetan; Neolithic to Historic East Asians, Mbuti)* showed the additional shared derived alleles from source populations except for Henan ancient population or their related ancestral populations. 16

Figure S65. Results of affinity-f_4 statistics for the form f_4*(Wadian_LN, Modern Tibetan; Neolithic to Historic East Asians, Mbuti)* showed the additional shared derived alleles from source populations except for Henan ancient population or their related ancestral populations. 17

Figure S66. Results of affinity-f_4 statistics for the form f_4*(Wadian_LN, Modern Tibetan; Neolithic to Historic East Asians, Mbuti)* showed the additional shared derived alleles from source populations except for Henan ancient population or their related ancestral populations. 18

Figure S67. Results of affinity-f_4 statistics for the form f_4*(Pingliangtai_LN, Modern Tibetan; Neolithic to Historic East Asians, Mbuti)* showed the additional shared derived alleles from source populations except for Henan ancient population or their related ancestral populations. 19

Figure S68. Results of affinity-f_4 statistics for the form f_4*(Haojiatai_LN, Modern Tibetan; Neolithic to Historic East Asians, Mbuti)* showed the additional shared derived alleles from source populations except for Henan ancient population or their related ancestral populations. 20

Figure S69. Results of affinity-f_4 statistics for the form f_4*(Haojiatai_LBIA, Modern Tibetan; Neolithic to Historic East Asians, Mbuti)* showed the additional shared derived alleles from source populations except for Henan ancient population or their related ancestral populations. 21

Figure S70. Results of affinity-f_4 statistics for the form f_4*(Jiaozuoniecun_LBIA, Modern Tibetan; Neolithic to Historic East Asians, Mbuti)* showed the additional shared derived alleles from source populations except for Henan ancient population or their related ancestral populations. 22

Figure S71. Results of affinity-f_4 statistics for the form f_4*(Luoheguxiang_LBIA, Modern Tibetan; Neolithic to Historic East Asians, Mbuti)* showed the additional shared derived alleles from source populations except for Henan ancient population or their related ancestral populations. 23

Additional gene flow events when we assumed that Tibetans' direct ancestor is inland Neolithic northern East Asian from Shaanxi or Inner Mongolia or their related ancestral populations 25

Figure S72. Results of affinity-f_4 statistics for the form f_4*(Wuzhuangguoliang, Modern Tibetan; Neolithic to Historic East Asians, Mbuti)* showed the additional shared derived alleles from source populations except for inland Neolithic northern East Asian from Shaanxi or Inner Mongolia or their related ancestral populations. 25

Figure S73. Results of affinity-f_4 statistics for the form f_4*(Shimao_LN, Modern Tibetan; Neolithic to Historic East Asians, Mbuti)* showed the additional shared derived alleles from source populations except for inland Neolithic northern East Asian from Shaanxi or Inner Mongolia or their related ancestral populations. 26

Figure S74. Results of affinity-f_4 statistics for the form f_4*(Miaozigou_MN, Modern Tibetan; Neolithic to Historic East Asians, Mbuti)* showed the additional shared derived alleles from source populations except for inland Neolithic northern East Asian from Shaanxi or Inner Mongolia or their related ancestral populations. 27

Figure S75. Results of affinity-f_4 statistics for the form *f_4(Yumin_EN, Modern Tibetan; Neolithic to Historic East Asians, Mbuti)* showed the additional shared derived alleles from source populations except for inland Neolithic northern East Asian from Shaanxi or Inner Mongolia or their related ancestral populations. 28

Additional gene flow events when we assumed that Tibetans' direct ancestor is inland Neolithic to Iron Age northern East Asian from upper Yellow River Basin or their related ancestral populations 30

Figure S76. Results of affinity-f_4 statistics for the form *f_4(Lajia_LN, Modern Tibetan; Neolithic to Historic East Asians, Mbuti)* showed the additional shared derived alleles from source populations except for inland Neolithic northern East Asian from Shaanxi or Inner Mongolia or their related ancestral populations. 30

Figure S77. Results of affinity-f_4 statistics for the form *f_4(Jinchankou_LN, Modern Tibetan; Neolithic to Historic East Asians, Mbuti)* showed the additional shared derived alleles from source populations except for inland Neolithic northern East Asian from Shaanxi or Inner Mongolia or their related ancestral populations. 31

Figure S78. Results of affinity-f_4 statistics for the form *f_4(Dacaozi_IA, Modern Tibetan; Neolithic to Historic East Asians, Mbuti)* showed the additional shared derived alleles from source populations except for inland Neolithic northern East Asian from Shaanxi or Inner Mongolia or their related ancestral populations. 32

Additional gene flow events when we assumed that Tibetans' direct ancestor is Liao River ancients or their related ancestral populations 33

Figure S79. Results of affinity-f_4 statistics for the form *f_4(Banhashan_MN, Modern Tibetan; Neolithic to Historic East Asians, Mbuti)* showed the additional shared derived alleles from source populations except for ancestral populations from Liao River Basin. 33

Figure S80. Results of affinity-f_4 statistics for the form *f_4(Haminmangha_MN, Modern Tibetan; Neolithic to Historic East Asians, Mbuti)* showed the additional shared derived alleles from source populations except for ancestral populations from Liao River Basin. 34

Additional gene flow events when we assumed that Tibetans' direct ancestor is Yangtze rice farmer related ancestral populations

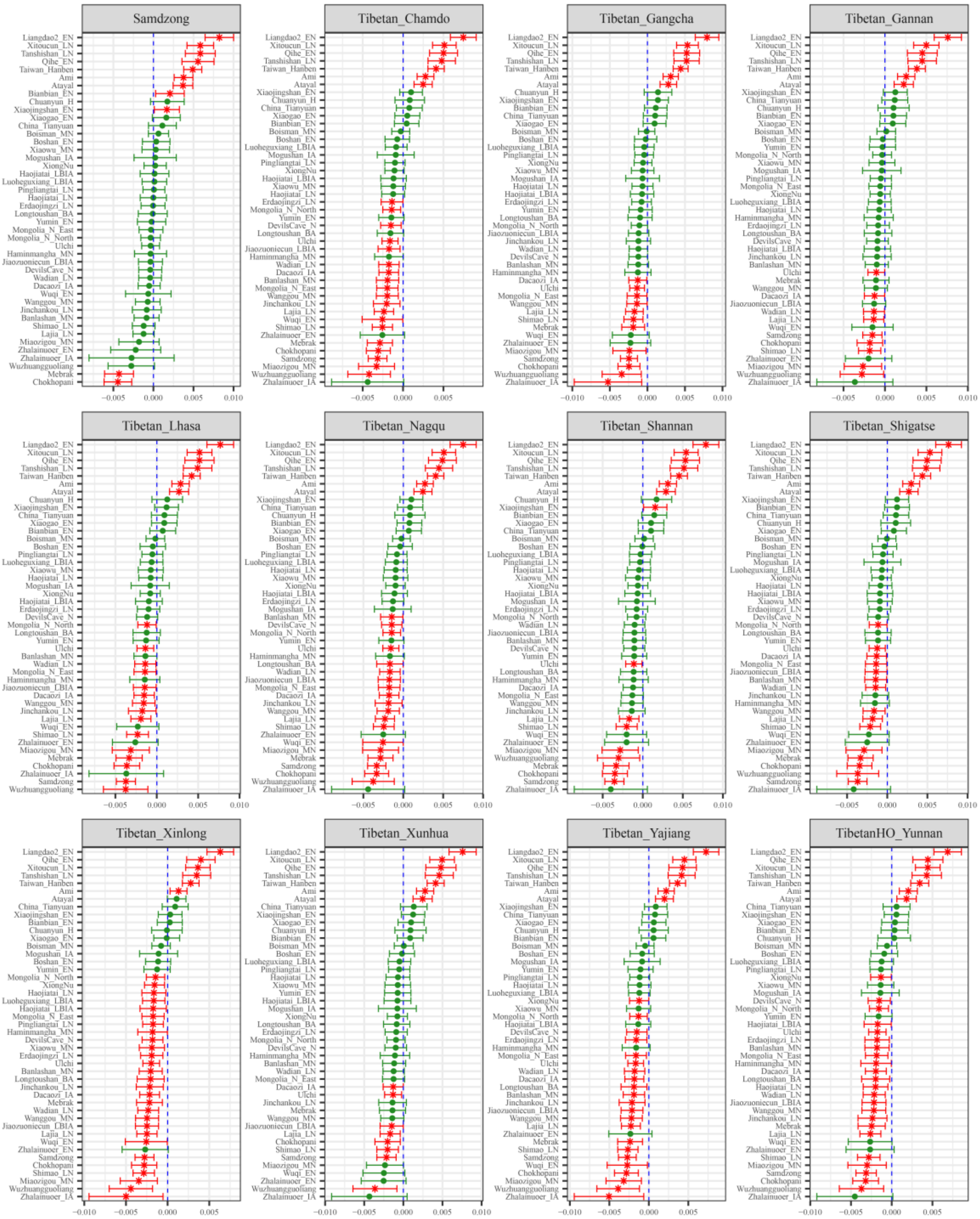

Figure S50. Results of affinity-f_4 statistics for the form f_4*(Liaodao1_EN, Modern Tibetan; Neolithic to Historic East Asians, Mbuti)* showed the additional shared derived alleles from source populations except for Yangtze rice farmer related ancestral populations.

Here, overlapping SNP loci included in the Affymetrix Human Origins platform among four analyzed populations were used. We used the genetic variation of Mbuti as the outgroup. Red asterisk point meant the significant value (Absolute value of Z-scores larger than three or equal to three) observed in the symmetry-f_4 statistics and green circle point denoted the non-significant f_4-statistic values (Absolute value of Z-scores less than three). All ancient East Asians were listed along the Y-axis and f_4 values were labeled along the X-axis. All results were faceted or grouped via Tibetan populations. Significant negative f_4 values indicated that the third population shared more alleles with the second population, and also means Tibetan obtained additional gene flow from the third source population (or related populations). And the significant positive f_4 value indicated that the third population shared more derived

alleles with the first population. The value of f_4-statistics equal to zero was marked as the blue dash line. The bar indicated three standard errors.

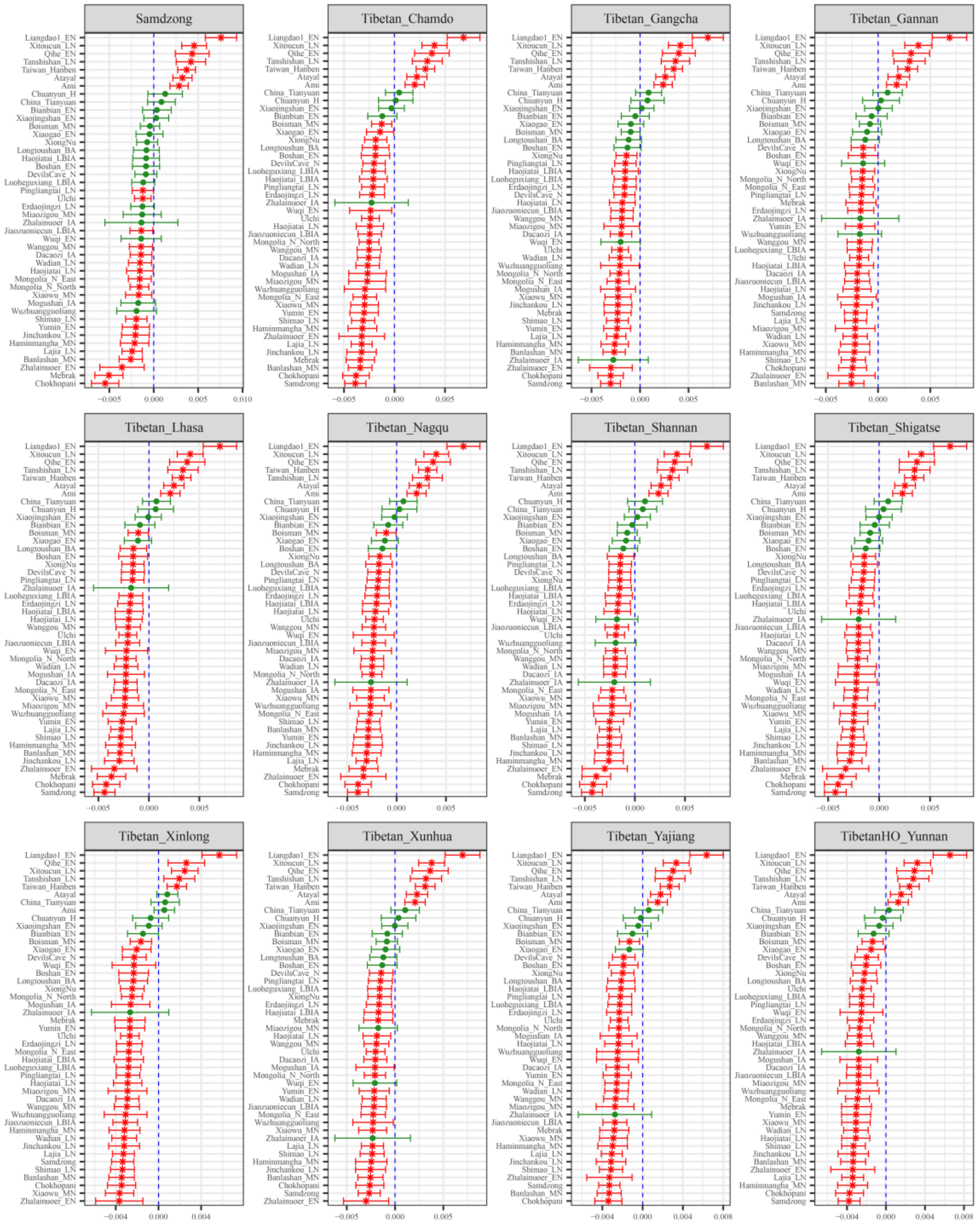

Figure S51. Results of affinity-f_4 statistics for the form f_4*(Liaodao2_EN, Modern Tibetan; Neolithic to Historic East Asians, Mbuti)* showed the additional shared derived alleles from source populations except for Yangtze rice farmer related ancestral populations.

Here, overlapping SNP loci included in the Affymetrix Human Origins platform among four analyzed populations were used. We used the genetic variation of Mbuti as the outgroup. Red asterisk point meant the significant value (Absolute value of Z-scores larger than three or equal to three) observed in the symmetry-f_4 statistics and green circle point denoted the non-significant f_4-statistic values (Absolute value of Z-scores less than three). All ancient East Asians were listed along the Y-axis and f_4 values were labeled along the X-axis. All results were faceted or grouped via Tibetan populations. Significant negative f_4 values indicated that the third population shared more alleles with the second population, and also means Tibetan obtained additional gene flow from the third source population (or related populations). And the significant positive f_4 value indicated that the third population shared more derived

alleles with the first population. The value of f_4-statistics equal to zero was marked as the blue dash line. The bar indicated three standard errors.

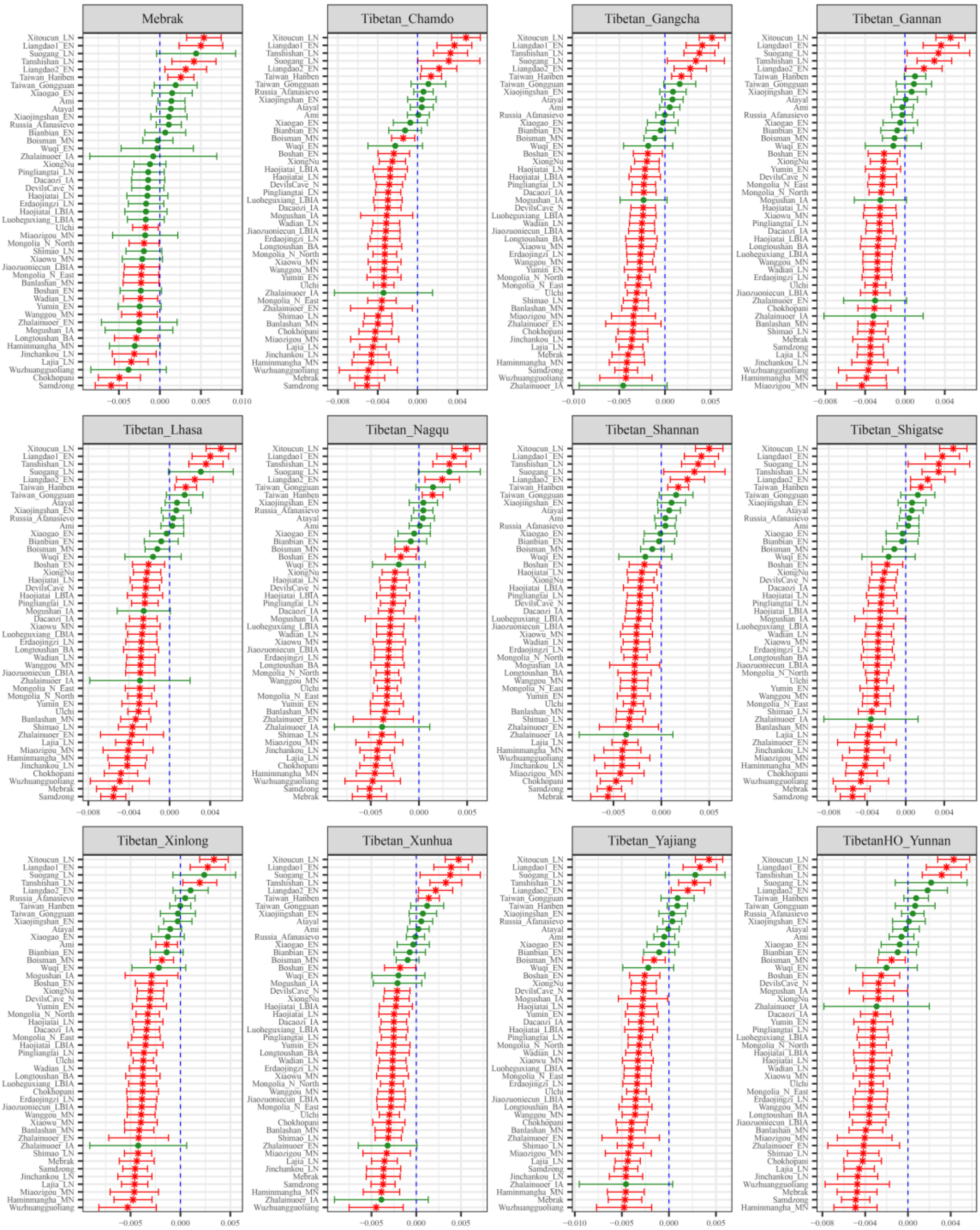

Figure S52. Results of affinity-f_4 statistics for the form f_4*(Qihe_EN, Modern Tibetan; Neolithic to Historic East Asians, Mbuti)* showed the additional shared derived alleles from source populations except for Yangtze rice farmer related ancestral populations.

Here, overlapping SNP loci included in the Affymetrix Human Origins platform among four analyzed populations were used. We used the genetic variation of Mbuti as the outgroup. Red asterisk point meant the significant value (Absolute value of Z-scores larger than three or equal to three) observed in the symmetry-f_4 statistics and green circle point denoted the non-significant f_4-statistic values (Absolute value of Z-scores less than three). All ancient East Asians were listed along the Y-axis and f_4 values were labeled along the X-axis. All results were faceted or grouped via Tibetan populations. Significant negative f_4 values indicated that the third population shared more alleles with the second population, and also means Tibetan obtained additional gene flow from the third source population (or related populations). And the significant positive f_4 value indicated that the third population shared more derived

alleles with the first population. The value of f_4-statistics equal to zero was marked as the blue dash line. The bar indicated three standard errors.

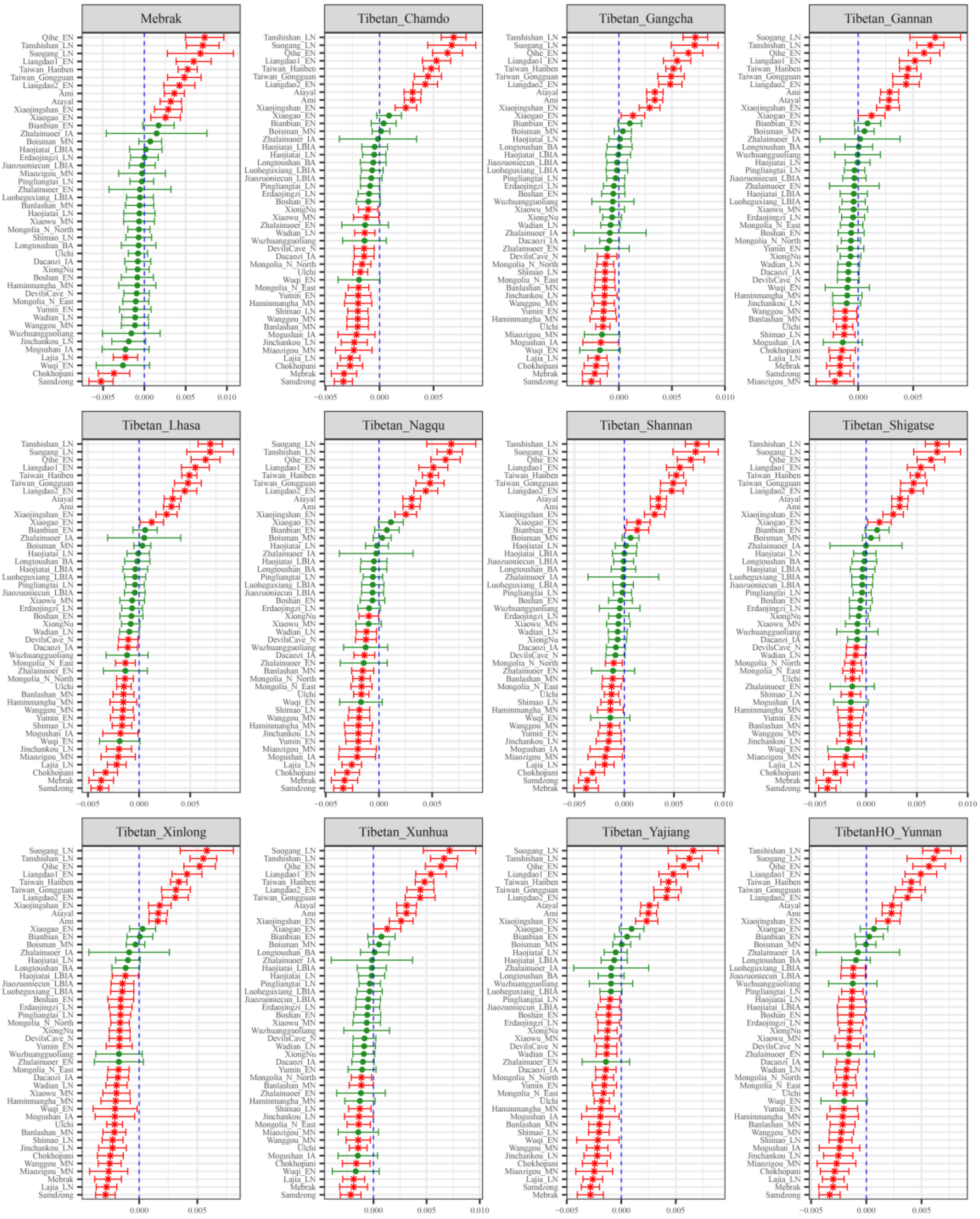

Figure S53. Results of affinity-f_4 statistics for the form f_4*(Xitoucun_LN, Modern Tibetan; Neolithic to Historic East Asians, Mbuti)* showed the additional shared derived alleles from source populations except for Yangtze rice farmer related ancestral populations.

Here, overlapping SNP loci included in the Affymetrix Human Origins platform among four analyzed populations were used. We used the genetic variation of Mbuti as the outgroup. Red asterisk point meant the significant value (Absolute value of Z-scores larger than three or equal to three) observed in the symmetry-f_4 statistics and green circle point denoted the non-significant f_4-statistic values (Absolute value of Z-scores less than three). All ancient East Asians were listed along the Y-axis and f_4 values were labeled along the X-axis. All results were faceted or grouped via Tibetan populations. Significant negative f_4 values indicated that the third population shared more alleles with the second population, and also means Tibetan obtained additional gene flow from the third source population (or related populations). And the significant positive f_4 value indicated that the third population shared more derived

alleles with the first population. The value of f_4-statistics equal to zero was marked as the blue dash line. The bar indicated three standard errors.

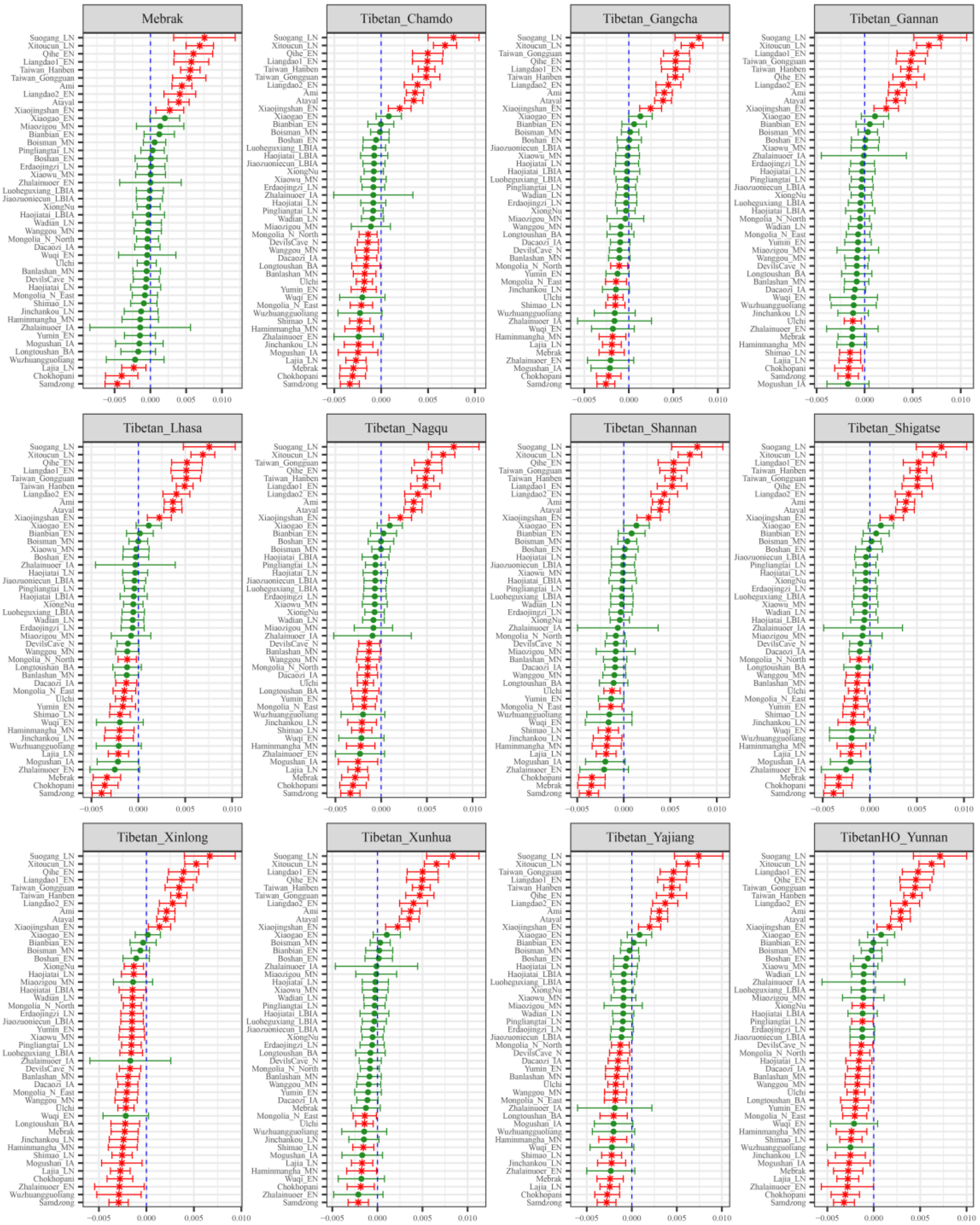

Figure S54. Results of affinity-f_4 statistics for the form f_4*(Tanshishan_LN, Modern Tibetan; Neolithic to Historic East Asians, Mbuti)* showed the additional shared derived alleles from source populations except for Yangtze rice farmer related ancestral populations.

Here, overlapping SNP loci included in the Affymetrix Human Origins platform among four analyzed populations were used. We used the genetic variation of Mbuti as the outgroup. Red asterisk point meant the significant value (Absolute value of Z-scores larger than three or equal to three) observed in the symmetry-f_4 statistics and green circle point denoted the non-significant f_4-statistic values (Absolute value of Z-scores less than three). All ancient East Asians were listed along the Y-axis and f_4 values were labeled along the X-axis. All results were faceted or grouped via Tibetan populations. Significant negative f_4 values indicated that the third population shared more alleles with the second population, and also means Tibetan obtained additional gene flow from the third source population (or related populations). And the significant positive f_4 value indicated that the third population shared more derived

alleles with the first population. The value of f_4-statistics equal to zero was marked as the blue dash line. The bar indicated three standard errors.

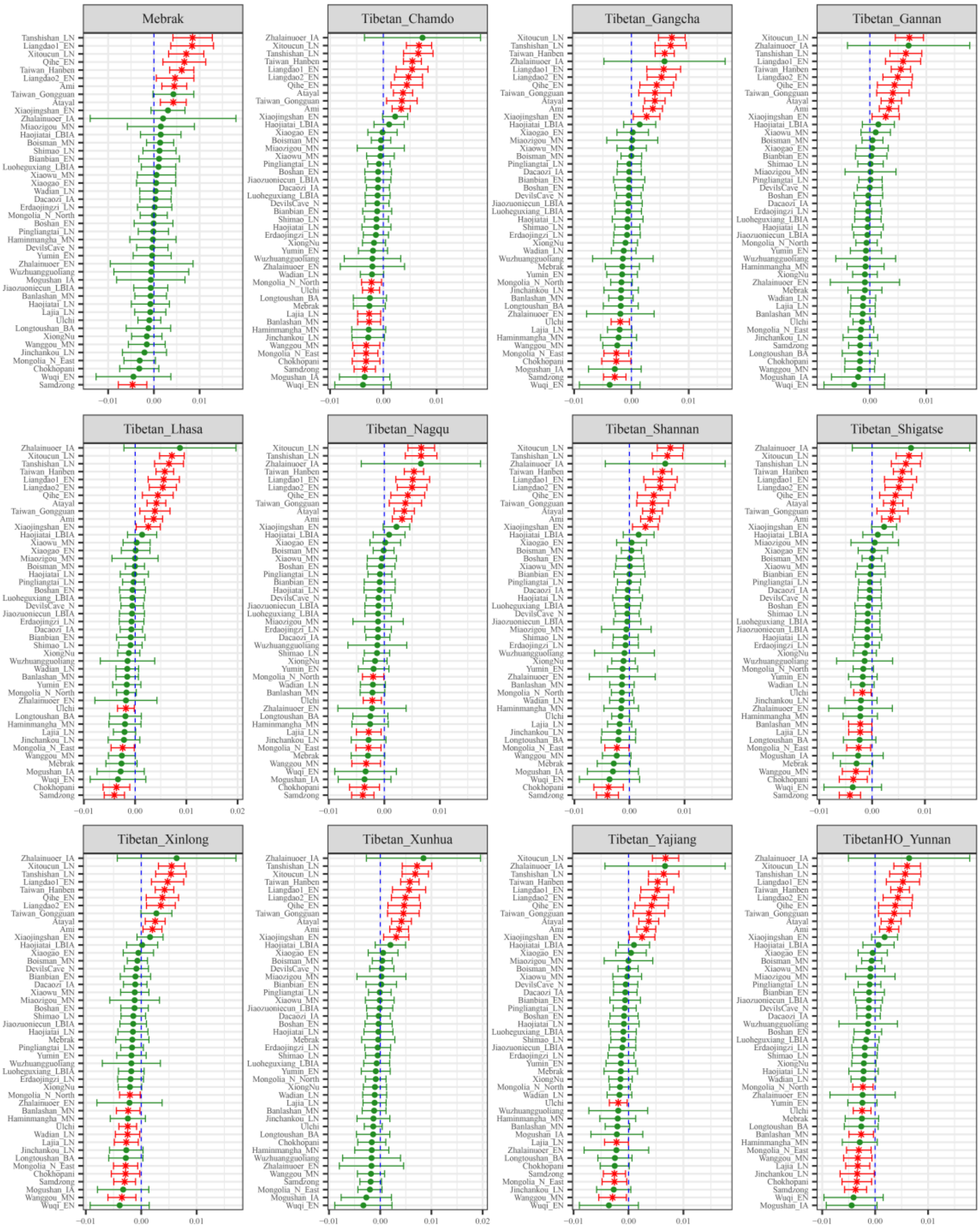

Figure S55. Results of affinity-f_4 statistics for the form f_4*(Suogang_LN, Modern Tibetan; Neolithic to Historic East Asians, Mbuti)* showed the additional shared derived alleles from source populations except for Yangtze rice farmer related ancestral populations.

Here, overlapping SNP loci included in the Affymetrix Human Origins platform among four analyzed populations were used. We used the genetic variation of Mbuti as the outgroup. Red asterisk point meant the significant value (Absolute value of Z-scores larger than three or equal to three) observed in the symmetry-f_4 statistics and green circle point denoted the non-significant f_4-statistic values (Absolute value of Z-scores less than three). All ancient East Asians were listed along the Y-axis and f_4 values were labeled along the X-axis. All results were faceted or grouped via Tibetan populations. Significant negative f_4 values indicated that the third population shared more alleles with the second population, and also means Tibetan obtained additional gene flow from the third source population (or related populations). And the significant positive f_4 value indicated that the third population shared more derived

alleles with the first population. The value of f_4-statistics equal to zero was marked as the blue dash line. The bar indicated three standard errors.

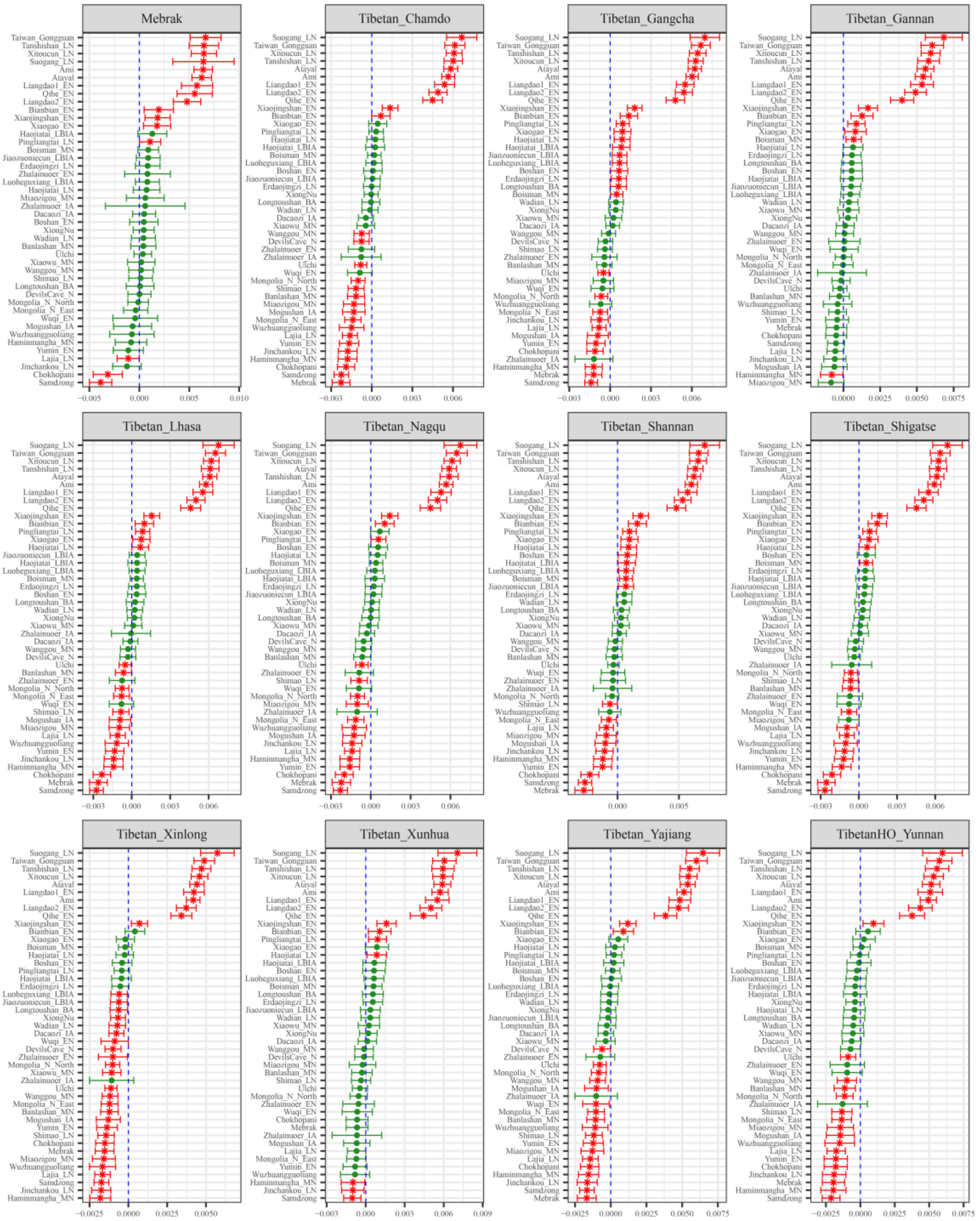

Figure S56. Results of affinity-f_4 statistics for the form f_4(*Taiwan_Hanben, Modern Tibetan; Neolithic to Historic East Asians, Mbuti*) showed the additional shared derived alleles from source populations except for Yangtze rice farmer related ancestral populations.

Here, overlapping SNP loci included in the Affymetrix Human Origins platform among four analyzed populations were used. We used the genetic variation of Mbuti as the outgroup. Red asterisk point meant the significant value (Absolute value of Z-scores larger than three or equal to three) observed in the symmetry-f_4 statistics and green circle point denoted the non-significant f_4-statistic values (Absolute value of Z-scores less than three). All ancient East Asians were listed along the Y-axis and f_4 values were labeled along the X-axis. All results were faceted or grouped via Tibetan populations. Significant negative f_4 values indicated that the third population shared more alleles with the second population, and also means Tibetan obtained additional gene flow from the third source population (or related

populations). And the significant positive f_4 value indicated that the third population shared more derived alleles with the first population. The value of f_4-statistics equal to zero was marked as the blue dash line. The bar indicated three standard errors.

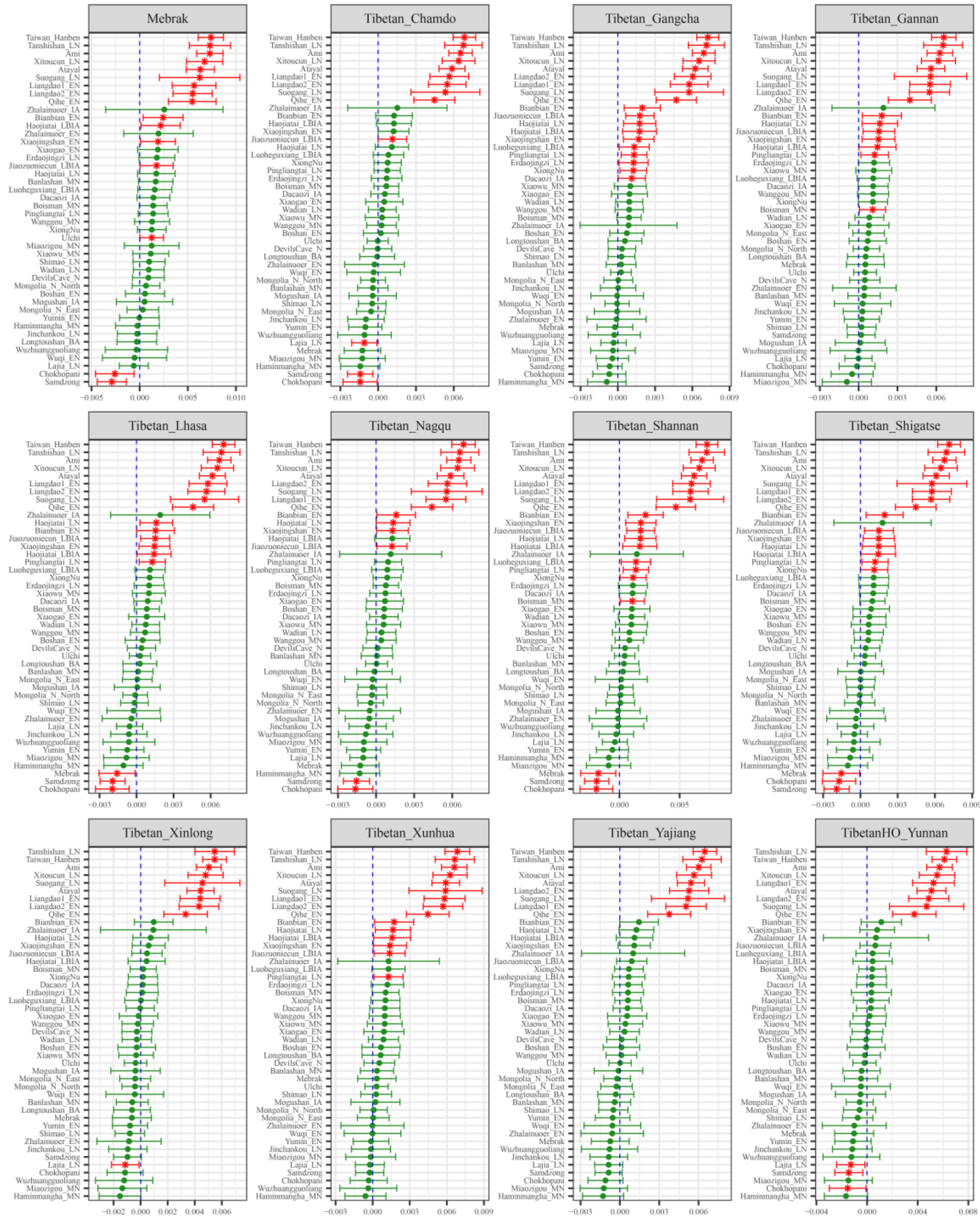

Figure S57. Results of affinity-f_4 statistics for the form f_4*(Taiwan_Gongguan, Modern Tibetan; Neolithic to Historic East Asians, Mbuti)* showed the additional shared derived alleles from source populations except for Yangtze rice farmer related ancestral populations.

Here, overlapping SNP loci included in the Affymetrix Human Origins platform among four analyzed populations were used. We used the genetic variation of Mbuti as the outgroup. Red asterisk point meant the significant value (Absolute value of Z-scores larger than three or equal to three) observed in the symmetry-f_4 statistics and green circle point denoted the non-significant f_4-statistic values (Absolute value of Z-scores less than three). All ancient East Asians were listed along the Y-axis and f_4 values were labeled along the X-axis. All results were faceted or grouped via Tibetan populations. Significant negative f_4 values indicated that the third population shared more alleles with the second population, and also means Tibetan obtained additional gene flow from the third source population (or related

populations). And the significant positive f_4 value indicated that the third population shared more derived alleles with the first population. The value of f_4-statistics equal to zero was marked as the blue dash line. The bar indicated three standard errors.

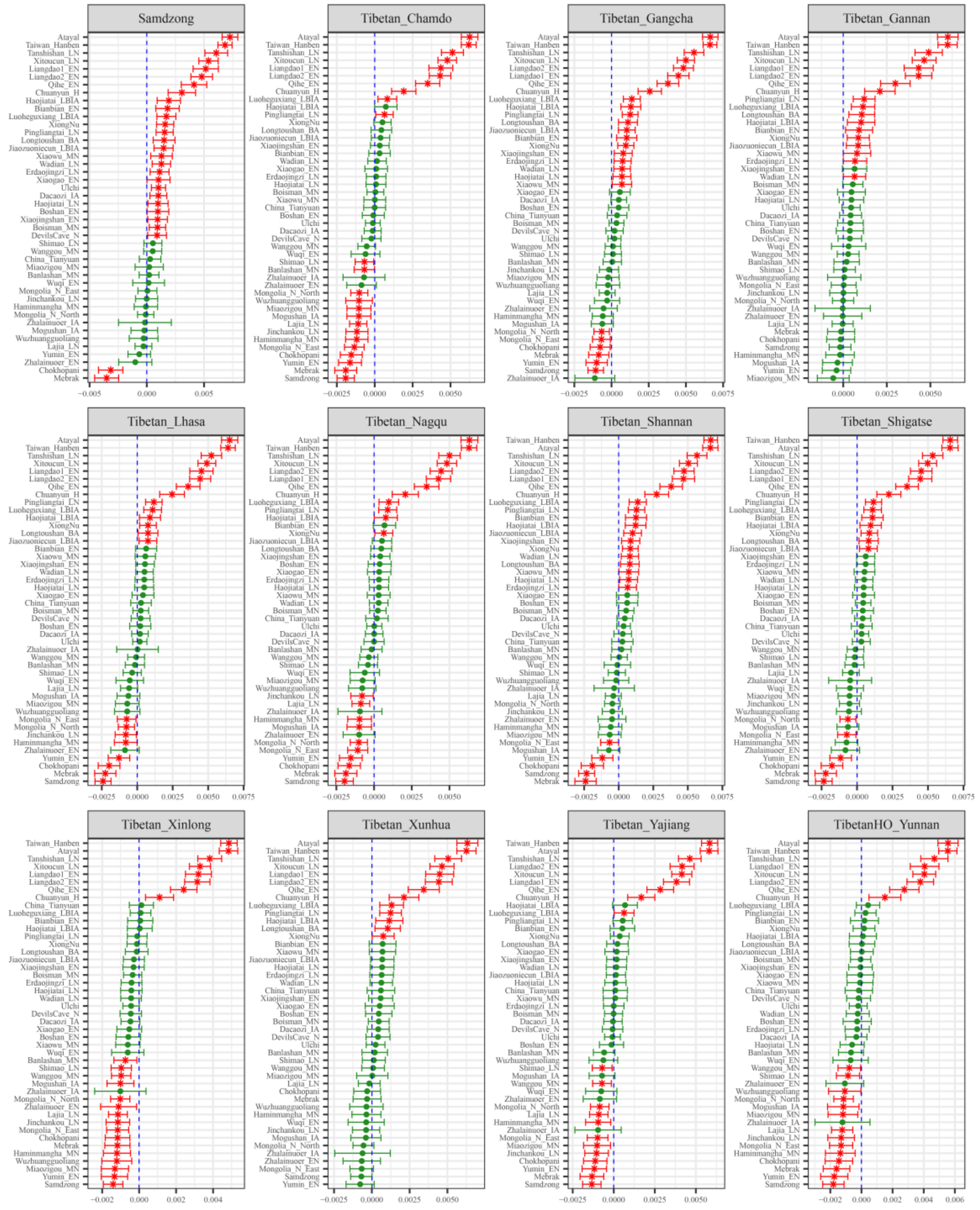

Figure S58. Results of affinity-f_4 statistics for the form f_4*(Ami, Modern Tibetan; Neolithic to Historic East Asians, Mbuti)* showed the additional shared derived alleles from source populations except for Yangtze rice farmer related ancestral populations.

Here, overlapping SNP loci included in the Affymetrix Human Origins platform among four analyzed populations were used. We used the genetic variation of Mbuti as the outgroup. Red asterisk point meant the significant value (Absolute value of Z-scores larger than three or equal to three) observed in the symmetry-f_4 statistics and green circle point denoted the non-significant f_4-statistic values (Absolute value of Z-scores less than three). All ancient East Asians were listed along the Y-axis and f_4 values were labeled along the X-axis. All results were faceted or grouped via Tibetan populations. Significant negative f_4 values indicated that the third population shared more alleles with the second population, and also means Tibetan obtained additional gene flow from the third source population (or related

populations). And the significant positive f_4 value indicated that the third population shared more derived alleles with the first population. The value of f_4-statistics equal to zero was marked as the blue dash line. The bar indicated three standard errors.

Additional gene flow events when we assumed that Tibetans' direct ancestor is coastal Neolithic northern East Asian related ancestral populations

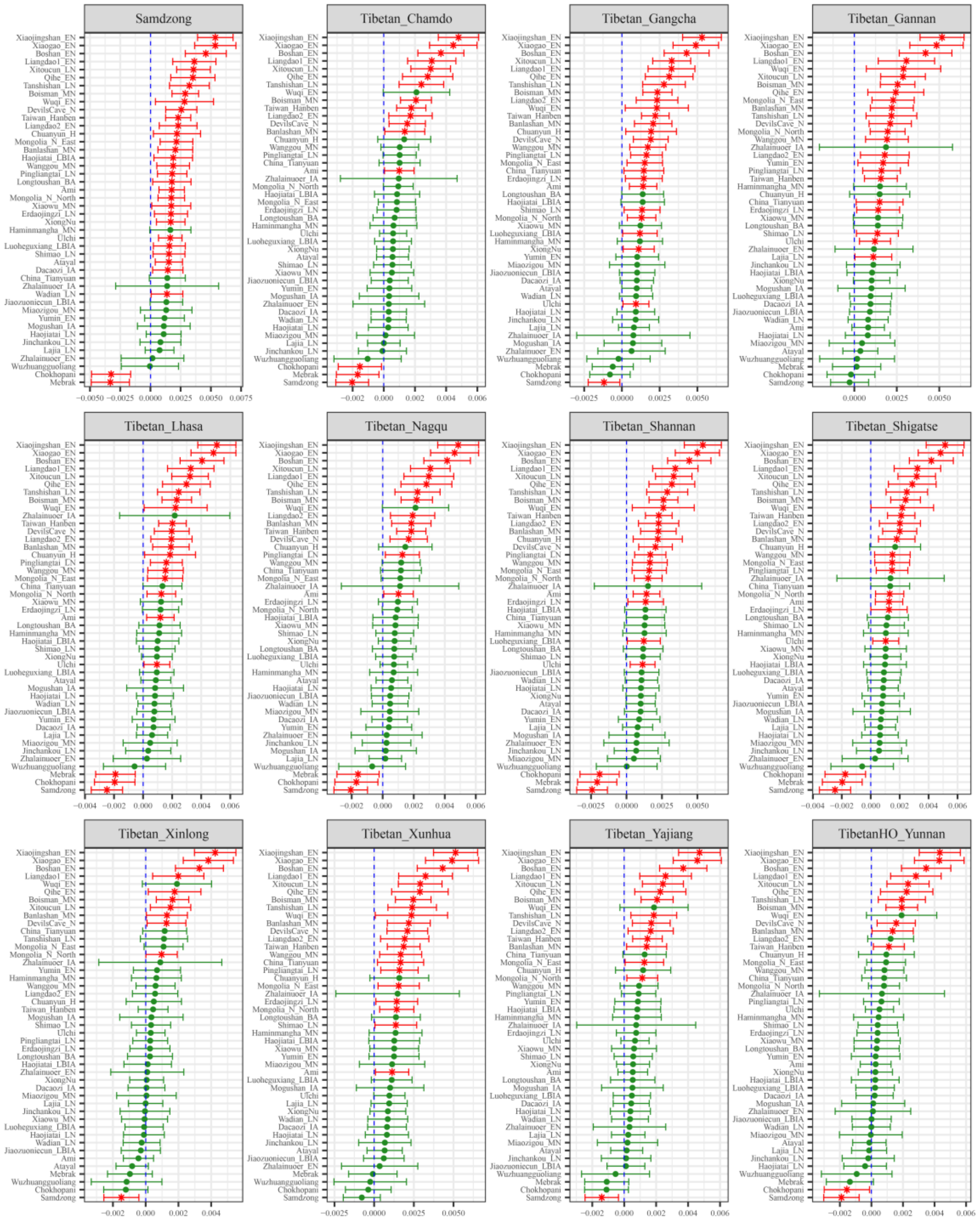

Figure S59. Results of affinity-f_4 statistics for the form f_4*(Bianbian_EN, Modern Tibetan; Neolithic to Historic East Asians, Mbuti)* showed the additional shared derived alleles from source populations except for coastal Neolithic northern East Asian related ancestral populations.
Here, overlapping SNP loci included in the Affymetrix Human Origins platform among four analyzed populations were used. We used the genetic variation of Mbuti as the outgroup. Red asterisk point meant the significant value (Absolute value of Z-scores larger than three or equal to three) observed in the symmetry-f_4 statistics and green circle point denoted the non-significant f_4-statistic values (Absolute value of Z-scores less than three). All ancient East Asians were listed along the Y-axis and f_4 values were labeled along the X-axis. All results were faceted or grouped via Tibetan populations. Significant negative f_4 values indicated that the third population shared more alleles with the second population, and also means Tibetan obtained additional gene flow from the third source population (or related populations). And the significant positive f_4 value indicated that the third population shared more derived alleles with the first population. The value of f_4-statistics equal to zero was marked as the blue dash line.

The bar indicated three standard errors.

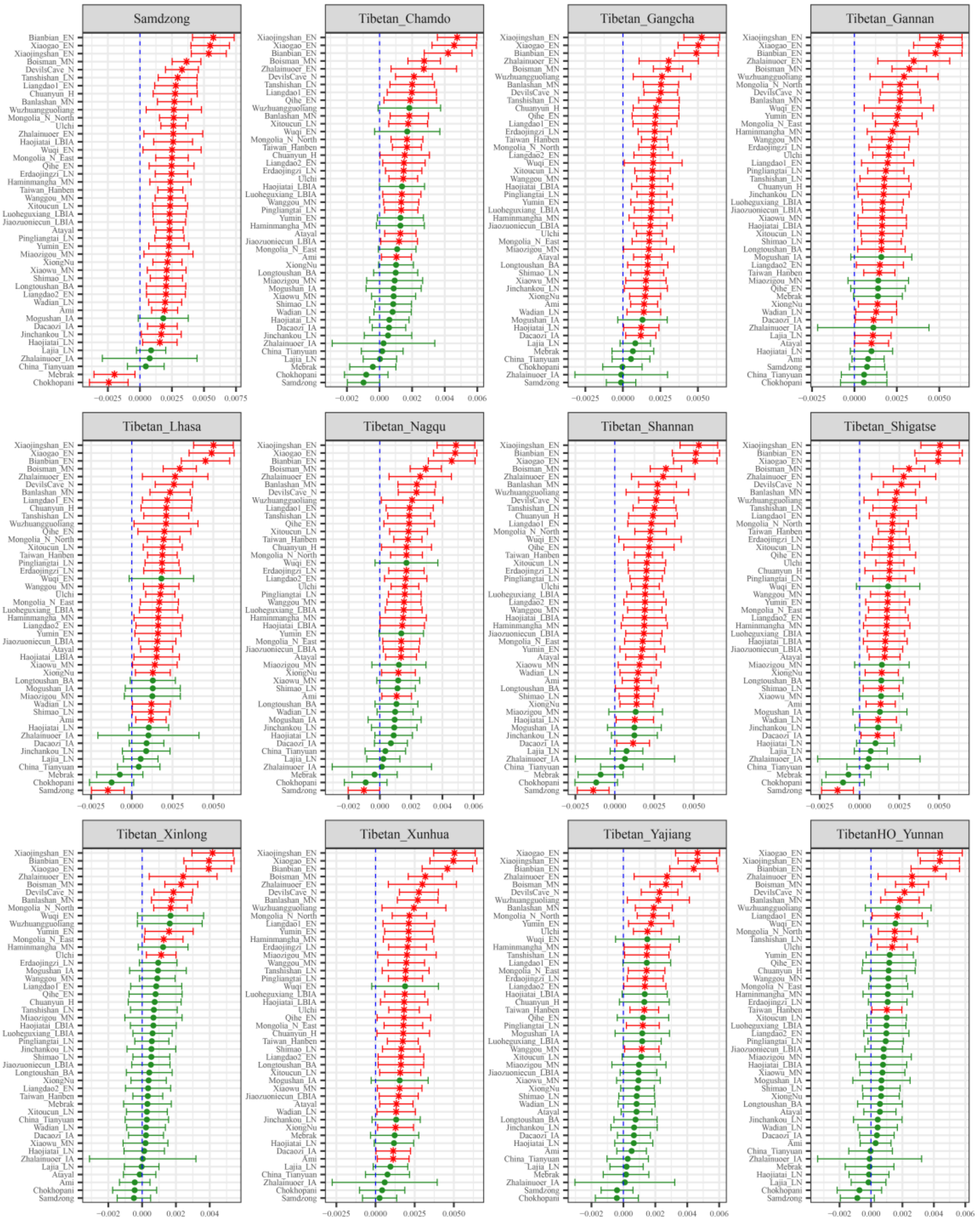

Figure S60. Results of affinity-f_4 statistics for the form f_4(*Boshan_EN, Modern Tibetan; Neolithic to Historic East Asians, Mbuti*) showed the additional shared derived alleles from source populations except for coastal Neolithic northern East Asian related ancestral populations.
Here, overlapping SNP loci included in the Affymetrix Human Origins platform among four analyzed populations were used. We used the genetic variation of Mbuti as the outgroup. Red asterisk point meant the significant value (Absolute value of Z-scores larger than three or equal to three) observed in the symmetry-f_4 statistics and green circle point denoted the non-significant f_4-statistic values (Absolute value of Z-scores less than three). All ancient East Asians were listed along the Y-axis and f_4 values were labeled along the X-axis. All results were faceted or grouped via Tibetan populations. Significant negative f_4 values indicated that the third population shared more alleles with the second population, and also means Tibetan obtained additional gene flow from the third source population (or related populations). And the significant positive f_4 value indicated that the third population shared more derived alleles with the first population. The value of f_4-statistics equal to zero was marked as the blue dash line. The bar indicated three standard errors.

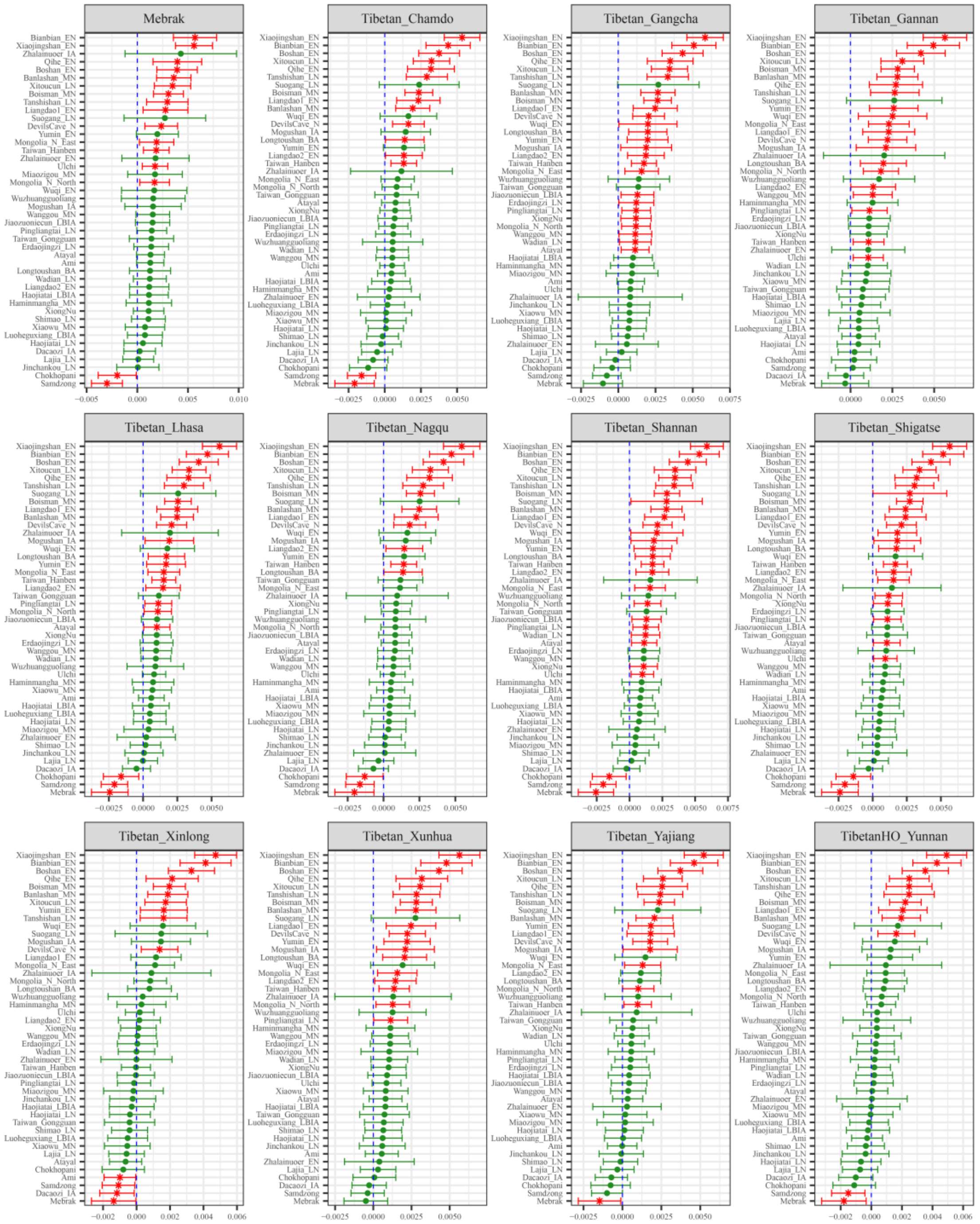

Figure S61. Results of affinity-f_4 statistics for the form f_4*(Xiaogao_EN, Modern Tibetan; Neolithic to Historic East Asians, Mbuti)* showed the additional shared derived alleles from source populations except for coastal Neolithic northern East Asian related ancestral populations.
Here, overlapping SNP loci included in the Affymetrix Human Origins platform among four analyzed populations were used. We used the genetic variation of Mbuti as the outgroup. Red asterisk point meant the significant value (Absolute value of Z-scores larger than three or equal to three) observed in the symmetry-f_4 statistics and green circle point denoted the non-significant f_4-statistic values (Absolute value of Z-scores less than three). All ancient East Asians were listed along the Y-axis and f_4 values were labeled along the X-axis. All results were faceted or grouped via Tibetan populations. Significant negative f_4 values indicated that the third population shared more alleles with the second population, and also means Tibetan obtained additional gene flow from the third source population (or related populations). And the significant positive f_4 value indicated that the third population shared more derived alleles with the first population. The value of f_4-statistics equal to zero was marked as the blue dash line.

The bar indicated three standard errors.

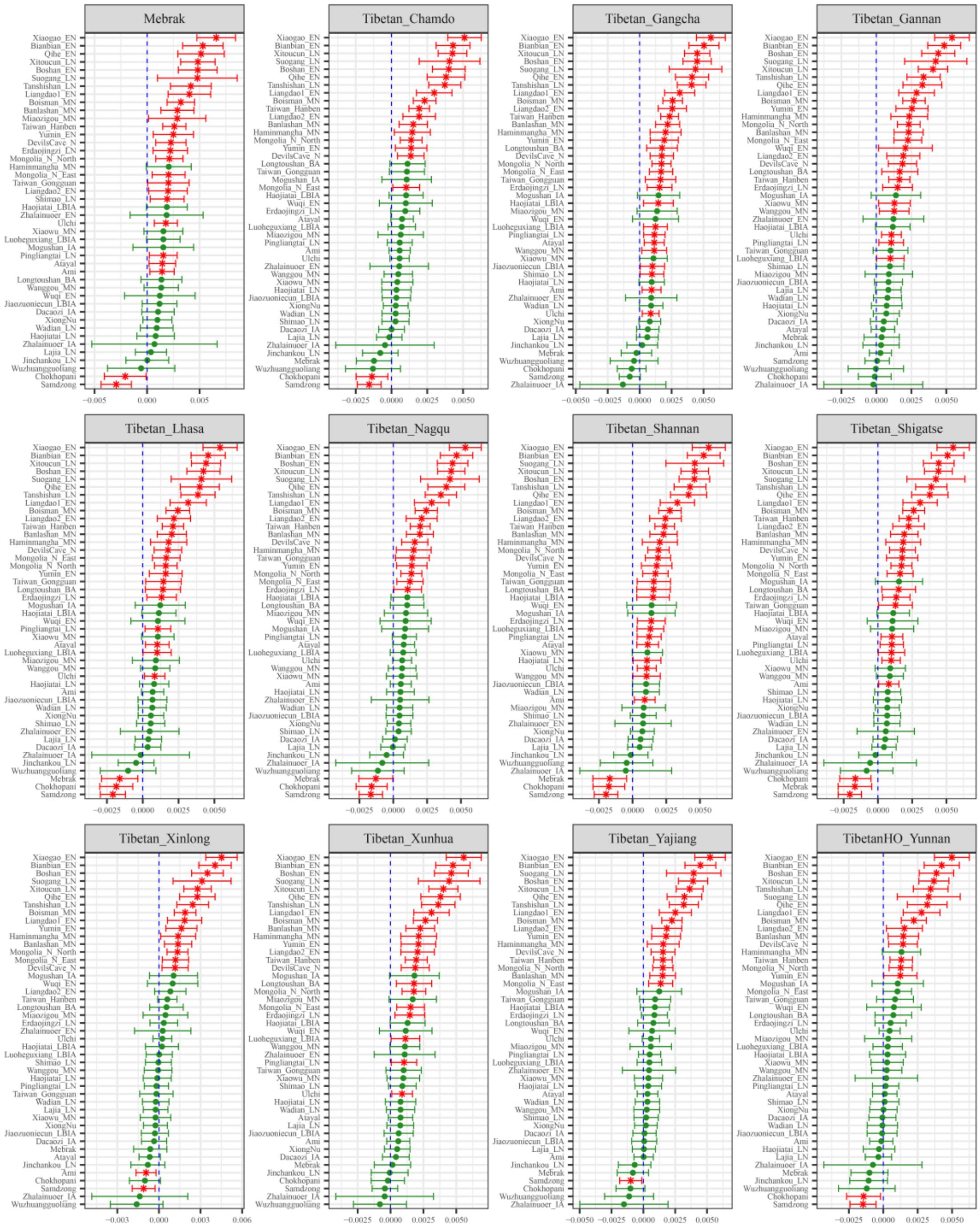

Figure S62. Results of affinity-f_4 statistics for the form f_4*(Xiaojingshan_EN, Modern Tibetan; Neolithic to Historic East Asians, Mbuti)* showed the additional shared derived alleles from source populations except for coastal Neolithic northern East Asian related ancestral populations.
Here, overlapping SNP loci included in the Affymetrix Human Origins platform among four analyzed populations were used. We used the genetic variation of Mbuti as the outgroup. Red asterisk point meant the significant value (Absolute value of Z-scores larger than three or equal to three) observed in the symmetry-f_4 statistics and green circle point denoted the non-significant f_4-statistic values (Absolute value of Z-scores less than three). All ancient East Asians were listed along the Y-axis and f_4 values were labeled along the X-axis. All results were faceted or grouped via Tibetan populations. Significant negative f_4 values indicated that the third population shared more alleles with the second population, and also means Tibetan obtained additional gene flow from the third source population (or related populations). And the significant positive f_4 value indicated that the third population shared more derived alleles with the first population. The value of f_4-statistics equal to zero was marked as the blue dash line.

The bar indicated three standard errors.

Additional gene flow events when we assumed that Tibetans' direct ancestor is Henan Neolithic to Iron Age East Asian related ancestral populations

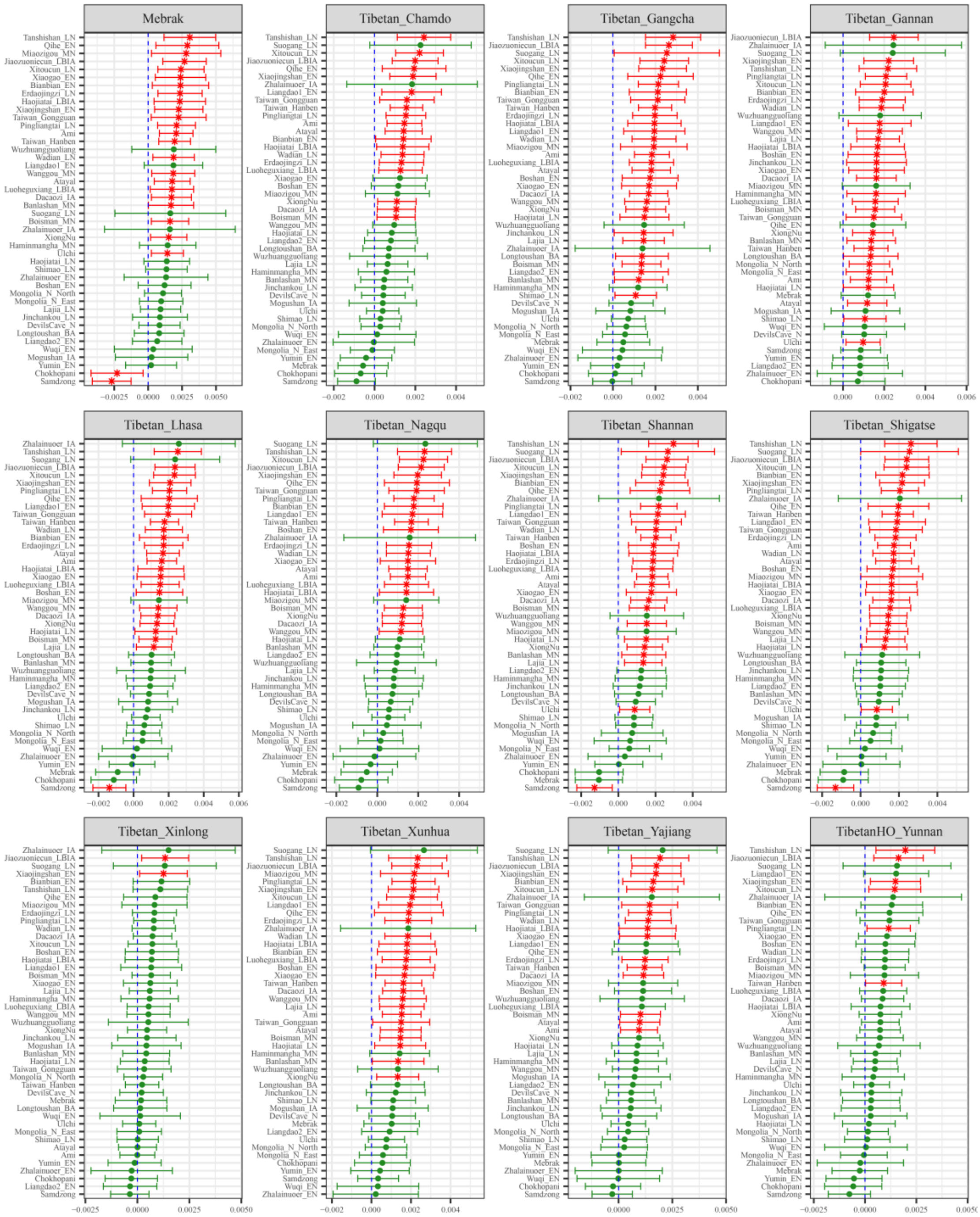

Figure S63. Results of affinity-f_4 statistics for the form f_4*(Xiaowu_LN, Modern Tibetan; Neolithic to Historic East Asians, Mbuti)* showed the additional shared derived alleles from source populations except for Henan ancient population or their related ancestral populations.
Here, overlapping SNP loci included in the Affymetrix Human Origins platform among four analyzed populations were used. We used the genetic variation of Mbuti as the outgroup. Red asterisk point meant the significant value (Absolute value of Z-scores larger than three or equal to three) observed in the symmetry-f_4 statistics and green circle point denoted the non-significant f_4-statistic values (Absolute value of Z-scores less than three). All ancient East Asians were listed along the Y-axis and f_4 values were labeled along the X-axis. All results were faceted or grouped via Tibetan populations. Significant negative f_4 values indicated that the third population shared more alleles with the second population, and

also means Tibetan obtained additional gene flow from the third source population (or related populations). And the significant positive f_4 value indicated that the third population shared more derived alleles with the first population. The value of f_4-statistics equal to zero was marked as the blue dash line. The bar indicated three standard errors.

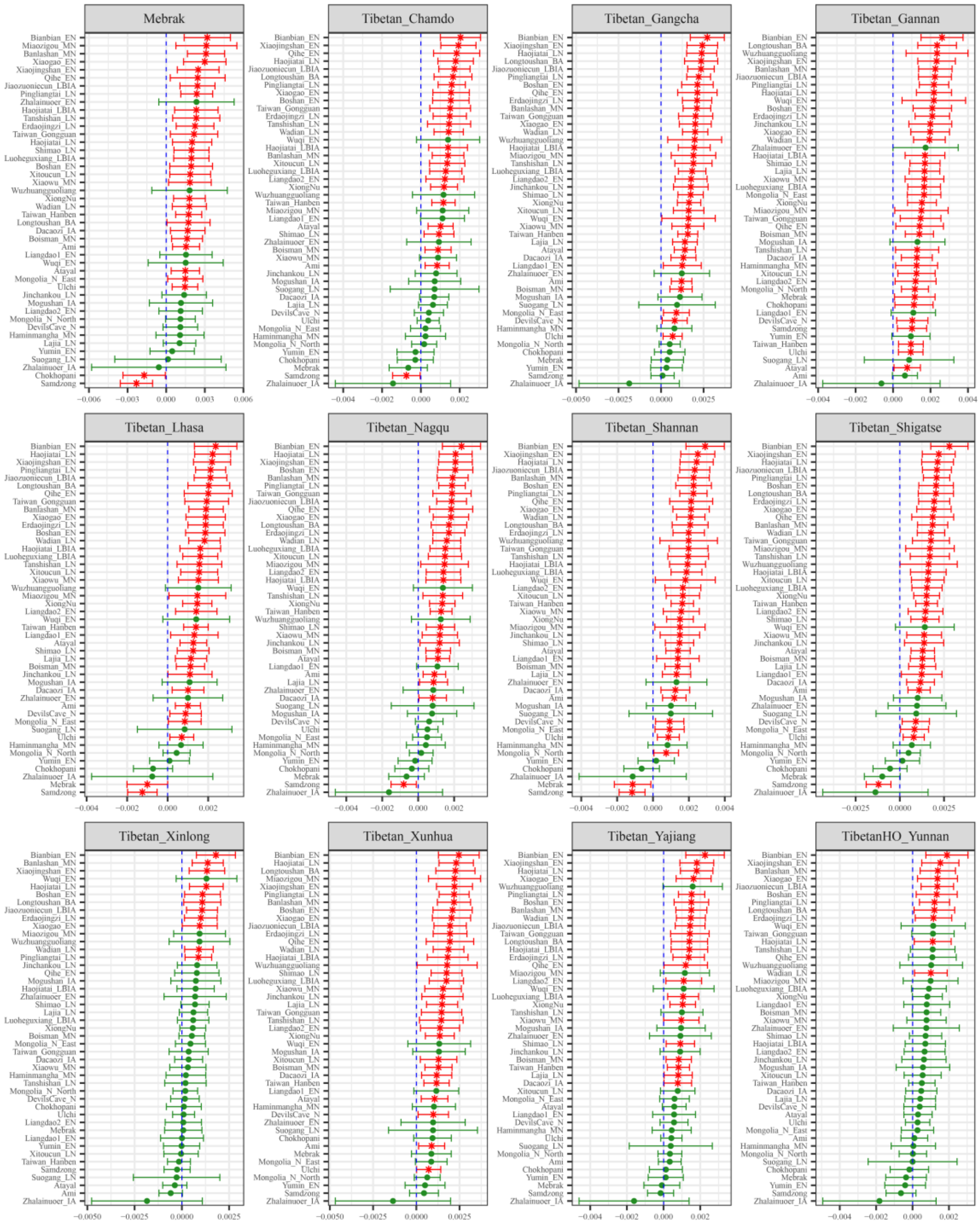

Figure S64. Results of affinity-f_4 statistics for the form f_4*(Wanggou_LN, Modern Tibetan; Neolithic to Historic East Asians, Mbuti)* showed the additional shared derived alleles from source populations except for Henan ancient population or their related ancestral populations.

Here, overlapping SNP loci included in the Affymetrix Human Origins platform among four analyzed populations were used. We used the genetic variation of Mbuti as the outgroup. Red asterisk point meant the significant value (Absolute value of Z-scores larger than three or equal to three) observed in the symmetry-f_4 statistics and green circle point denoted the non-significant f_4-statistic values (Absolute value of Z-scores less than three). All ancient East Asians were listed along the Y-axis and f_4 values were labeled along the X-axis. All results were faceted or grouped via Tibetan populations. Significant negative f_4 values indicated that the third population shared more alleles with the second population, and

also means Tibetan obtained additional gene flow from the third source population (or related populations). And the significant positive f_4 value indicated that the third population shared more derived alleles with the first population. The value of f_4-statistics equal to zero was marked as the blue dash line. The bar indicated three standard errors.

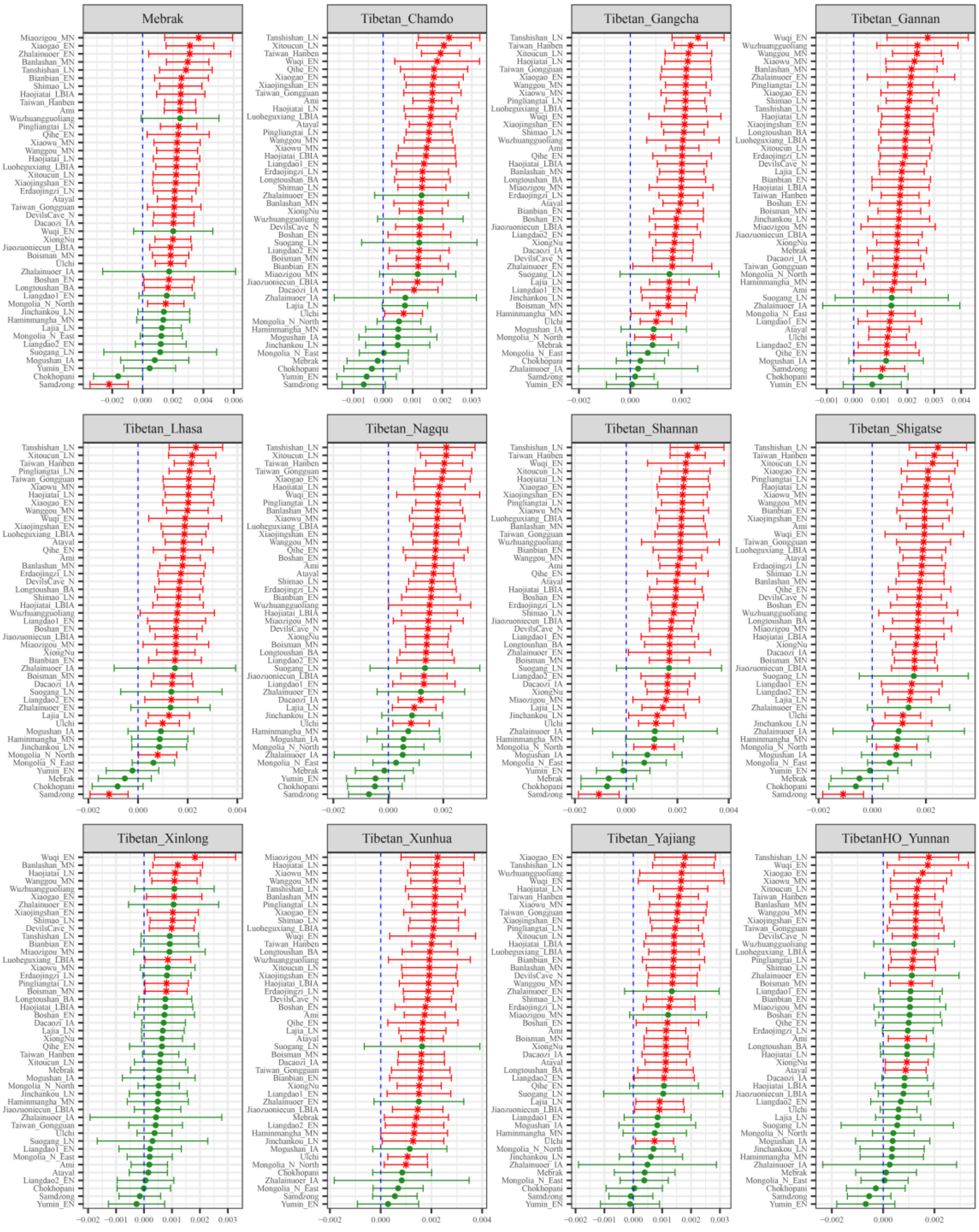

Figure S65. Results of affinity-f_4 statistics for the form f_4*(Wadian_LN, Modern Tibetan; Neolithic to Historic East Asians, Mbuti)* showed the additional shared derived alleles from source populations except for Henan ancient population or their related ancestral populations.

Here, overlapping SNP loci included in the Affymetrix Human Origins platform among four analyzed populations were used. We used the genetic variation of Mbuti as the outgroup. Red asterisk point meant the significant value (Absolute value of Z-scores larger than three or equal to three) observed in the symmetry-f_4 statistics and green circle point denoted the non-significant f_4-statistic values (Absolute value of Z-scores less than three). All ancient East Asians were listed along the Y-axis and f_4 values were labeled along the X-axis. All results were faceted or grouped via Tibetan populations. Significant

negative f_4 values indicated that the third population shared more alleles with the second population, and also means Tibetan obtained additional gene flow from the third source population (or related populations). And the significant positive f_4 value indicated that the third population shared more derived alleles with the first population. The value of f_4-statistics equal to zero was marked as the blue dash line. The bar indicated three standard errors.

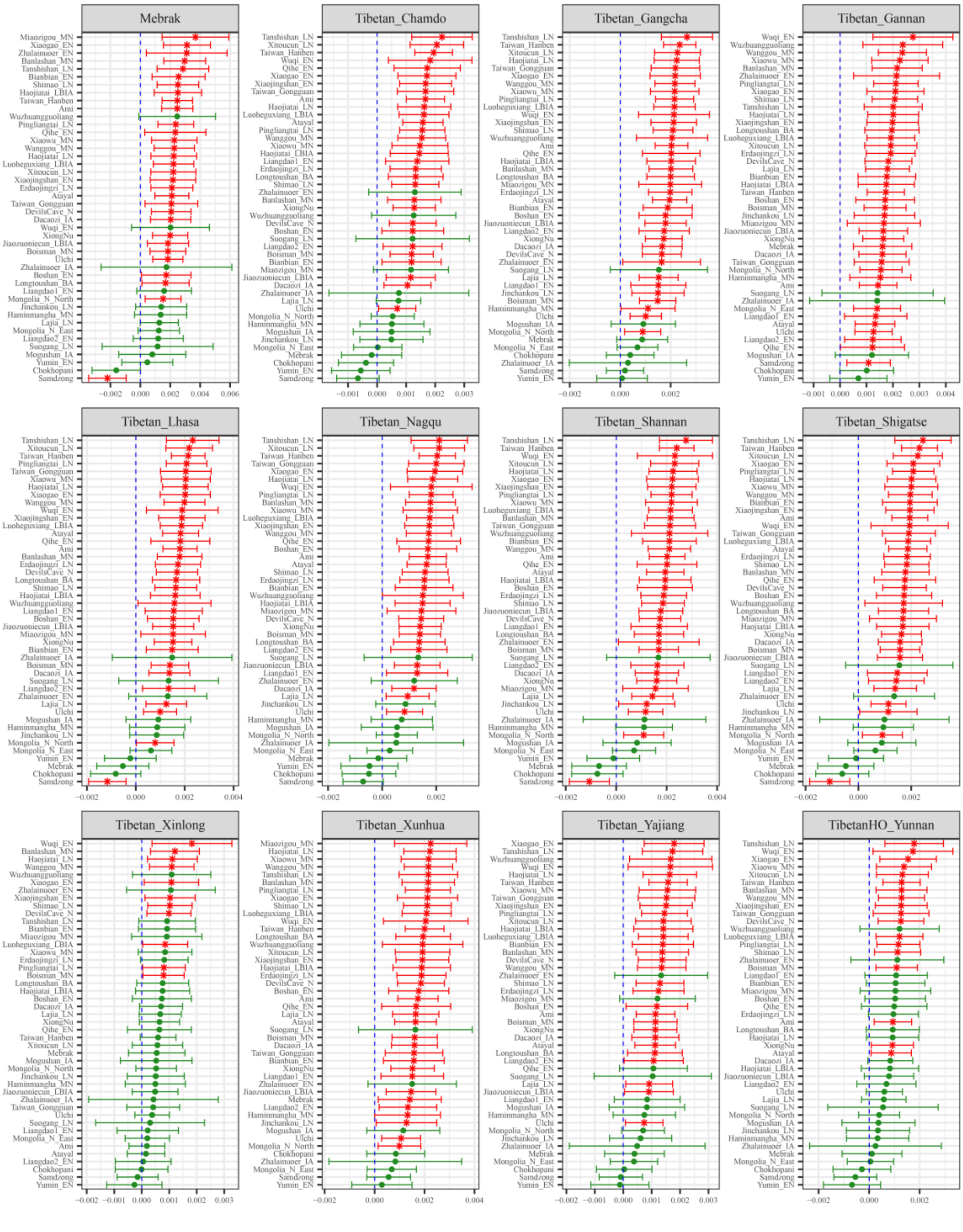

Figure S66. Results of affinity-f_4 statistics for the form f_4*(Wadian_LN, Modern Tibetan; Neolithic to Historic East Asians, Mbuti)* showed the additional shared derived alleles from source populations except for Henan ancient population or their related ancestral populations.

Here, overlapping SNP loci included in the Affymetrix Human Origins platform among four analyzed populations were used. We used the genetic variation of Mbuti as the outgroup. Red asterisk point meant the significant value (Absolute value of Z-scores larger than three or equal to three) observed in the symmetry-f_4 statistics and green circle point denoted the non-significant f_4-statistic values (Absolute value of Z-scores less than three). All ancient East Asians were listed along the Y-axis and f_4 values were labeled along the X-axis. All results were faceted or grouped via Tibetan populations. Significant

negative f_4 values indicated that the third population shared more alleles with the second population, and also means Tibetan obtained additional gene flow from the third source population (or related populations). And the significant positive f_4 value indicated that the third population shared more derived alleles with the first population. The value of f_4-statistics equal to zero was marked as the blue dash line. The bar indicated three standard errors.

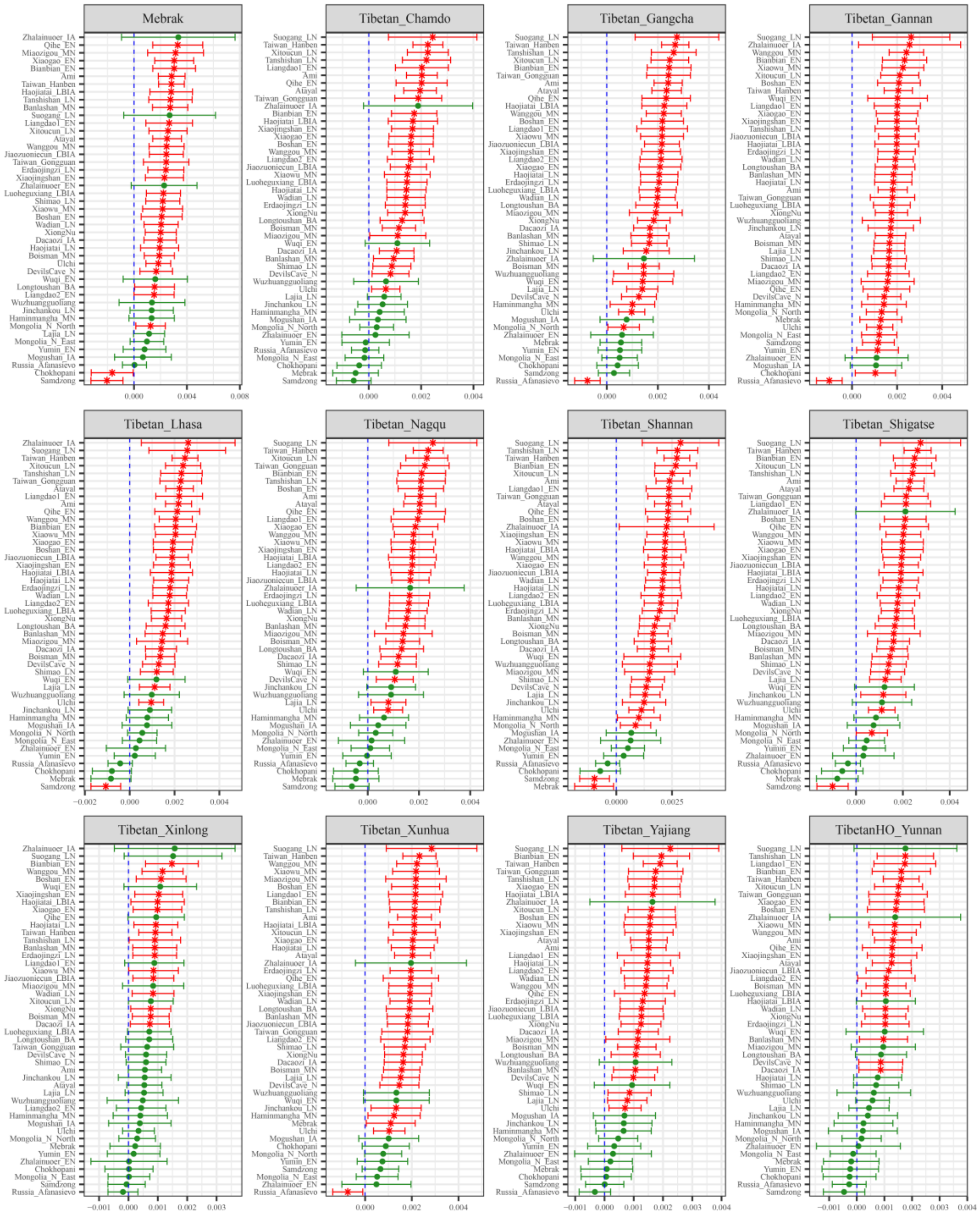

Figure S67. Results of affinity-f_4 statistics for the form f_4(*Pingliangtai_LN, Modern Tibetan; Neolithic to Historic East Asians, Mbuti*) showed the additional shared derived alleles from source populations except for Henan ancient population or their related ancestral populations.

Here, overlapping SNP loci included in the Affymetrix Human Origins platform among four analyzed populations were used. We used the genetic variation of Mbuti as the outgroup. Red asterisk point meant the significant value (Absolute value of Z-scores larger than three or equal to three) observed in the symmetry-f_4 statistics and green circle point denoted the non-significant f_4-statistic values (Absolute value of Z-scores less than three). All ancient East Asians were listed along the Y-axis and f_4 values were labeled along the X-axis. All results were faceted or grouped via Tibetan populations. Significant

negative f_4 values indicated that the third population shared more alleles with the second population, and also means Tibetan obtained additional gene flow from the third source population (or related populations). And the significant positive f_4 value indicated that the third population shared more derived alleles with the first population. The value of f_4-statistics equal to zero was marked as the blue dash line. The bar indicated three standard errors.

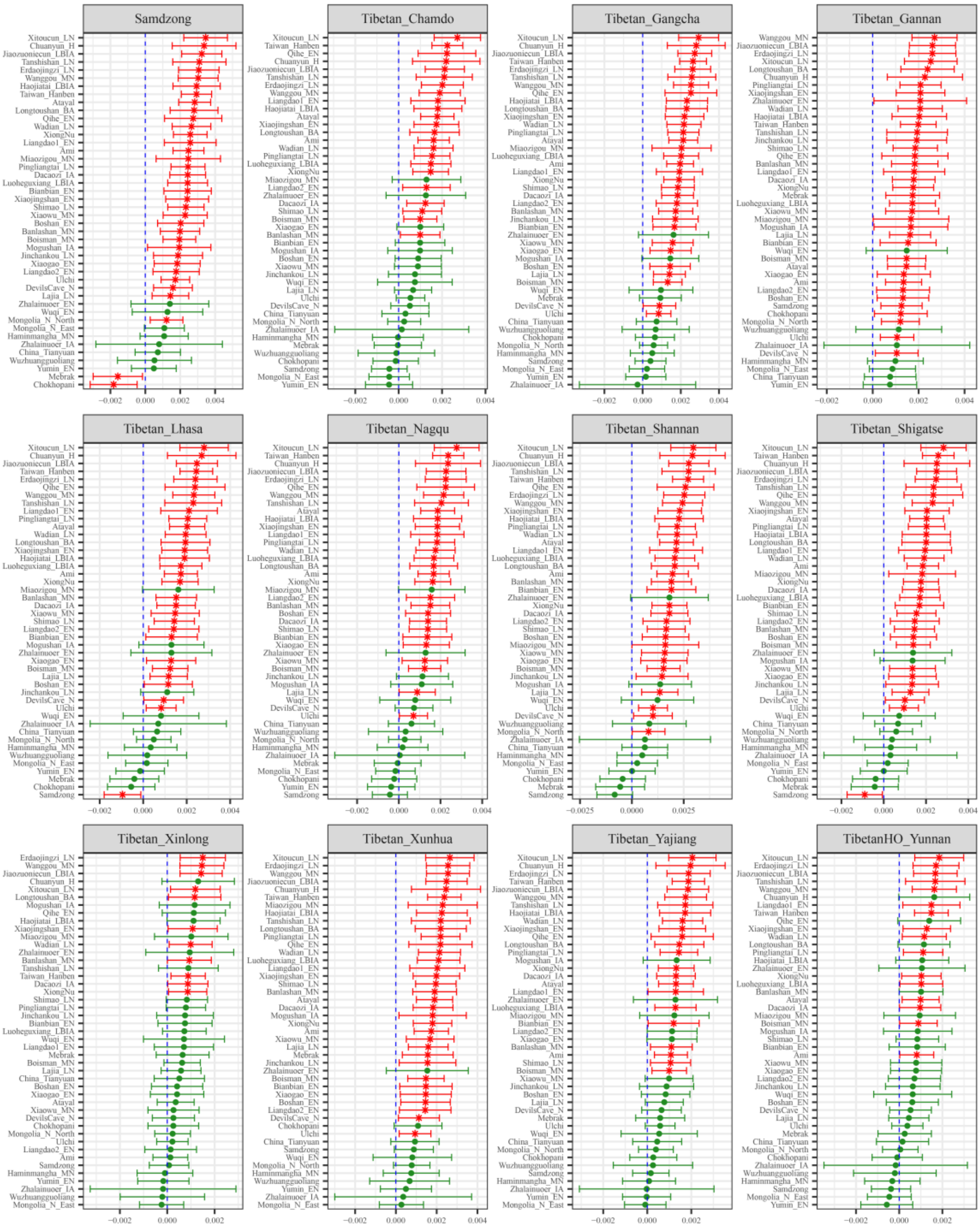

Figure S68. Results of affinity-f_4 statistics for the form f_4(*Haojiatai_LN, Modern Tibetan; Neolithic to Historic East Asians, Mbuti*) showed the additional shared derived alleles from source populations except for Henan ancient population or their related ancestral populations.

Here, overlapping SNP loci included in the Affymetrix Human Origins platform among four analyzed populations were used. We used the genetic variation of Mbuti as the outgroup. Red asterisk point meant the significant value (Absolute value of Z-scores larger than three or equal to three) observed in the symmetry-f_4 statistics and green circle point denoted the non-significant f_4-statistic values (Absolute value of Z-scores less than three). All ancient East Asians were listed along the Y-axis and f_4 values were

labeled along the X-axis. All results were faceted or grouped via Tibetan populations. Significant negative f_4 values indicated that the third population shared more alleles with the second population, and also means Tibetan obtained additional gene flow from the third source population (or related populations). And the significant positive f_4 value indicated that the third population shared more derived alleles with the first population. The value of f_4-statistics equal to zero was marked as the blue dash line. The bar indicated three standard errors.

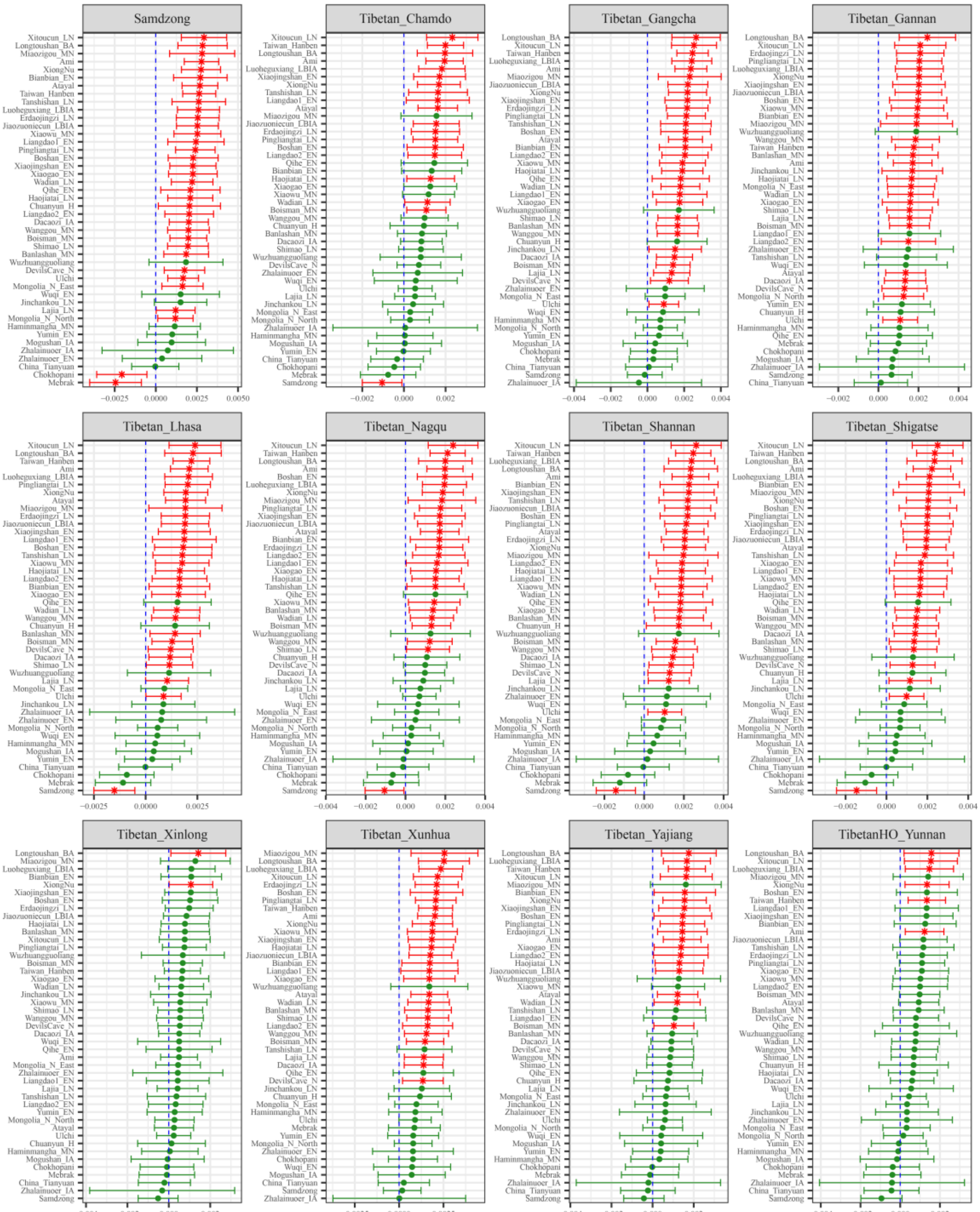

Figure S69. Results of affinity-f_4 statistics for the form f_4*(Haojiatai_LBIA, Modern Tibetan; Neolithic to Historic East Asians, Mbuti)* showed the additional shared derived alleles from source populations except for Henan ancient population or their related ancestral populations.

Here, overlapping SNP loci included in the Affymetrix Human Origins platform among four analyzed populations were used. We used the genetic variation of Mbuti as the outgroup. Red asterisk point meant the significant value (Absolute value of Z-scores larger than three or equal to three) observed in the symmetry-f_4 statistics and green circle point denoted the non-significant f_4-statistic values (Absolute value of Z-scores less than three). All ancient East Asians were listed along the Y-axis and f_4 values were

labeled along the X-axis. All results were faceted or grouped via Tibetan populations. Significant negative f_4 values indicated that the third population shared more alleles with the second population, and also means Tibetan obtained additional gene flow from the third source population (or related populations). And the significant positive f_4 value indicated that the third population shared more derived alleles with the first population. The value of f_4-statistics equal to zero was marked as the blue dash line. The bar indicated three standard errors.

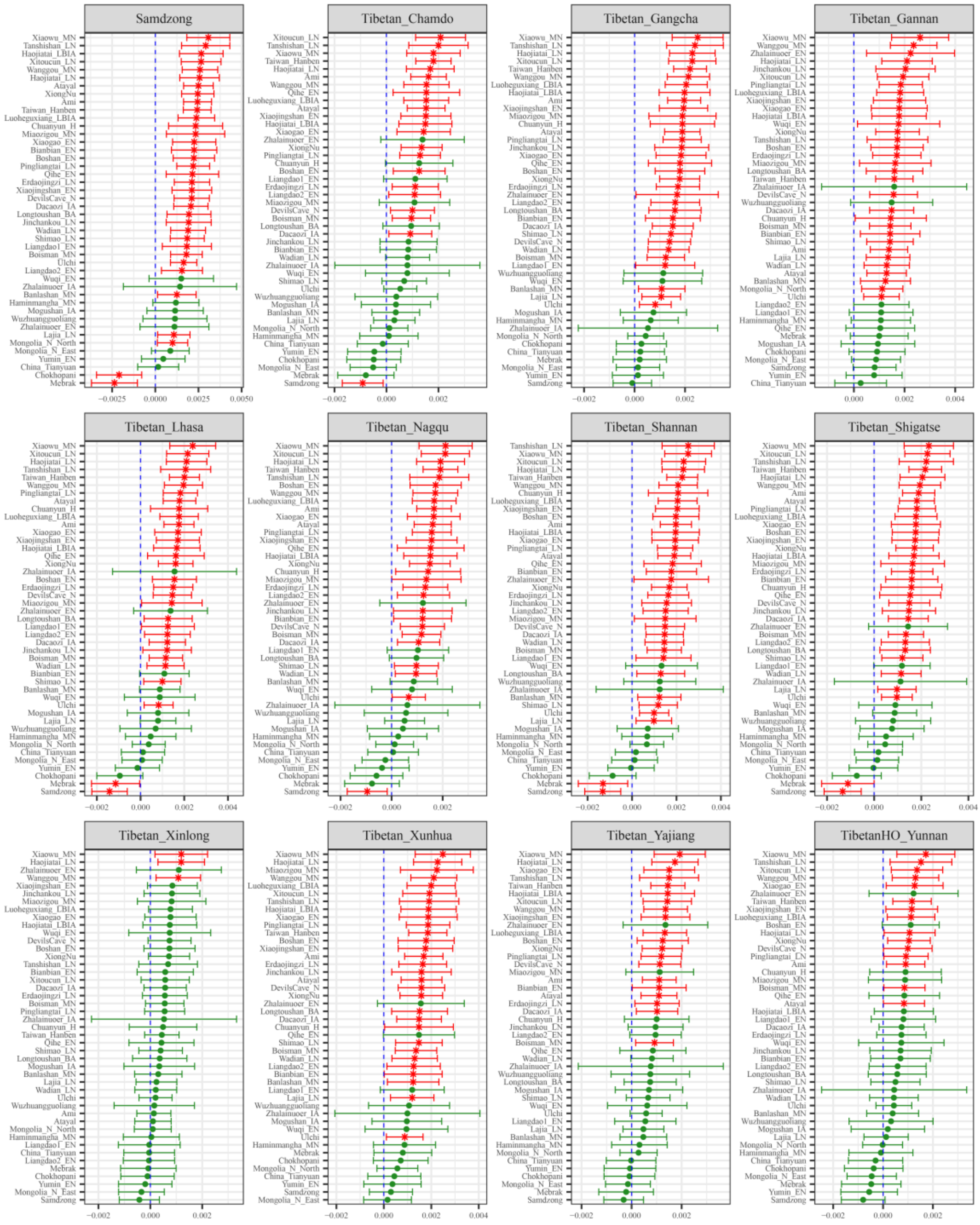

Figure S70. Results of affinity-f_4 statistics for the form f_4(*Jiaozuoniecun_LBIA, Modern Tibetan; Neolithic to Historic East Asians, Mbuti*) showed the additional shared derived alleles from source populations except for Henan ancient population or their related ancestral populations.

Here, overlapping SNP loci included in the Affymetrix Human Origins platform among four analyzed populations were used. We used the genetic variation of Mbuti as the outgroup. Red asterisk point meant the significant value (Absolute value of Z-scores larger than three or equal to three) observed in the symmetry-f_4 statistics and green circle point denoted the non-significant f_4-statistic values (Absolute value of Z-scores less than three). All ancient East Asians were listed along the Y-axis and f_4 values were

labeled along the X-axis. All results were faceted or grouped via Tibetan populations. Significant negative f_4 values indicated that the third population shared more alleles with the second population, and also means Tibetan obtained additional gene flow from the third source population (or related populations). And the significant positive f_4 value indicated that the third population shared more derived alleles with the first population. The value of f_4-statistics equal to zero was marked as the blue dash line. The bar indicated three standard errors.

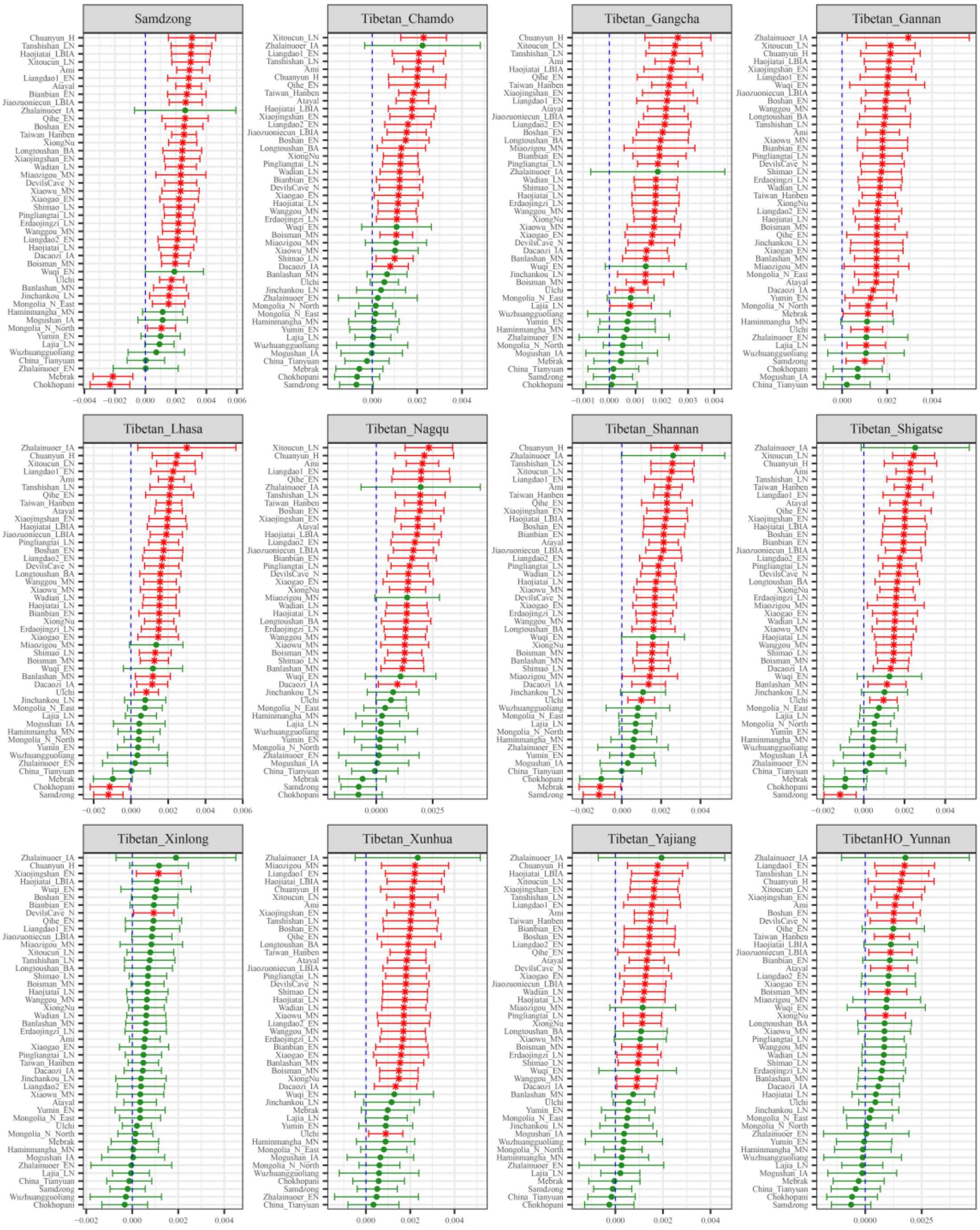

Figure S71. Results of affinity-f_4 statistics for the form f_4*(Luoheguxiang_LBIA, Modern Tibetan; Neolithic to Historic East Asians, Mbuti)* showed the additional shared derived alleles from source populations except for Henan ancient population or their related ancestral populations.

Here, overlapping SNP loci included in the Affymetrix Human Origins platform among four analyzed populations were used. We used the genetic variation of Mbuti as the outgroup. Red asterisk point meant the significant value (Absolute value of Z-scores larger than three or equal to three) observed in the symmetry-f_4 statistics and green circle point denoted the non-significant f_4-statistic values (Absolute value of Z-scores less than three). All ancient East Asians were listed along the Y-axis and f_4 values were

labeled along the X-axis. All results were faceted or grouped via Tibetan populations. Significant negative f_4 values indicated that the third population shared more alleles with the second population, and also means Tibetan obtained additional gene flow from the third source population (or related populations). And the significant positive f_4 value indicated that the third population shared more derived alleles with the first population. The value of f_4-statistics equal to zero was marked as the blue dash line. The bar indicated three standard errors.

Additional gene flow events when we assumed that Tibetans' direct ancestor is inland Neolithic northern East Asian from Shaanxi or Inner Mongolia or their related ancestral populations

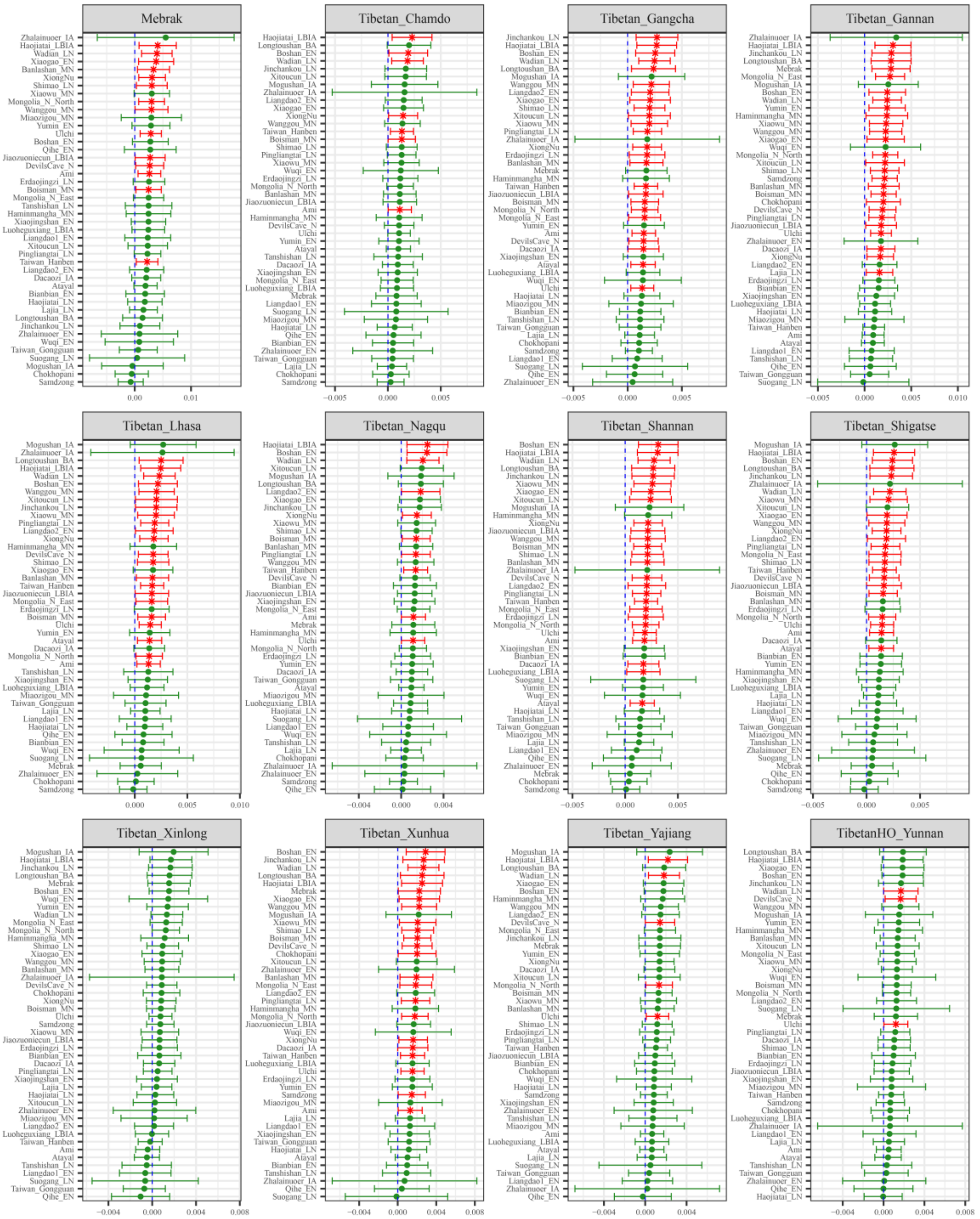

Figure S72. Results of affinity-*f₄* statistics for the form *f₄(Wuzhuangguoliang, Modern Tibetan; Neolithic to Historic East Asians, Mbuti)* showed the additional shared derived alleles from source populations except for inland Neolithic northern East Asian from Shaanxi or Inner Mongolia or their related ancestral populations.

Here, overlapping SNP loci included in the Affymetrix Human Origins platform among four analyzed populations were used. We used the genetic variation of Mbuti as the outgroup. Red asterisk point meant the significant value (Absolute value of Z-scores larger than three or equal to three) observed in the symmetry-f_4 statistics and green circle point denoted the non-significant f_4-statistic values (Absolute value of Z-scores less than three). All ancient East Asians were listed along the Y-axis and f_4 values were labeled along the X-axis. All results were faceted or grouped via Tibetan populations. Significant negative f_4 values indicated that the third population shared more alleles with the second population, and also means Tibetan obtained additional gene flow from the third source population (or related

populations). And the significant positive f_4 value indicated that the third population shared more derived alleles with the first population. The value of f_4-statistics equal to zero was marked as the blue dash line. The bar indicated three standard errors.

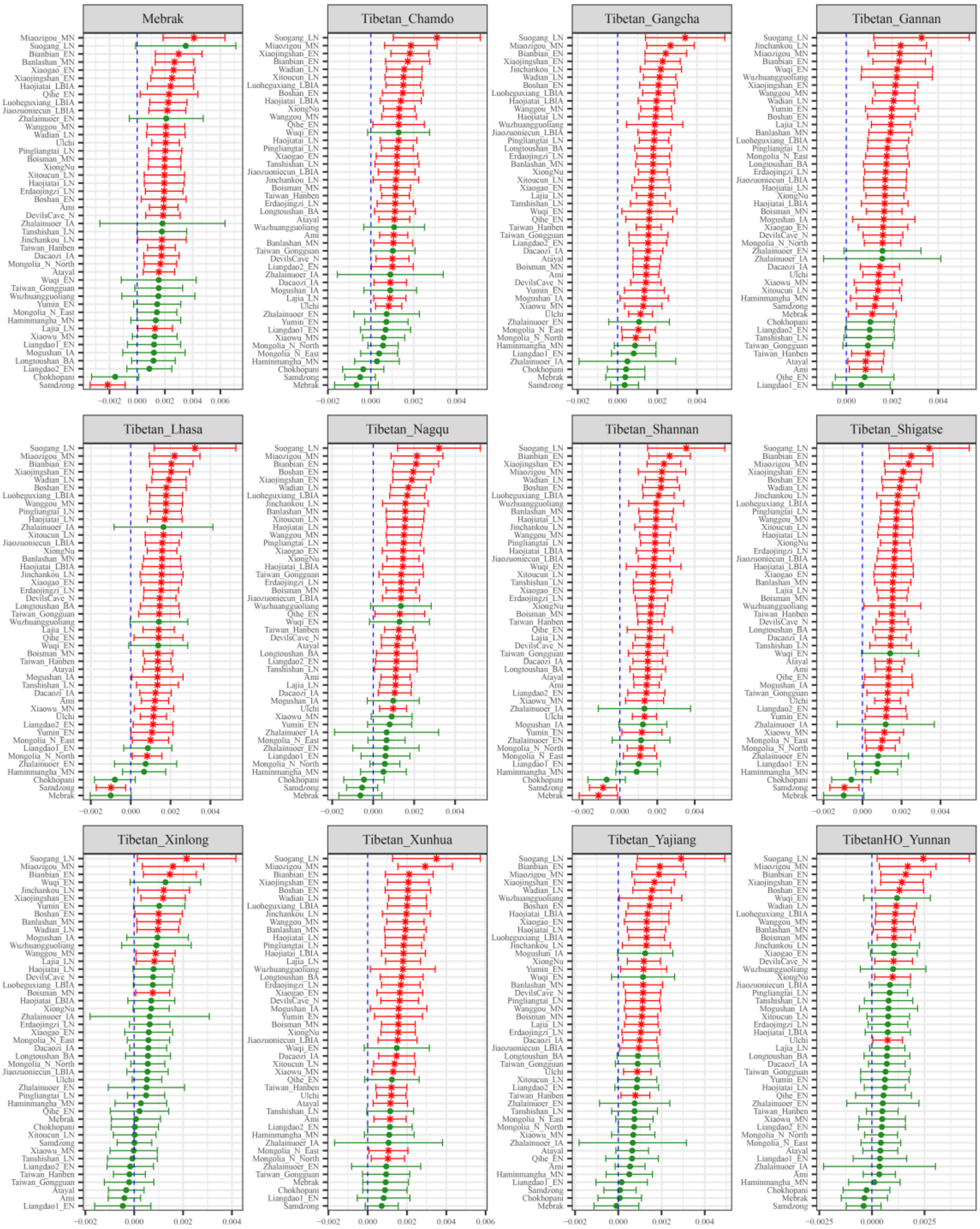

Figure S73. Results of affinity-f_4 statistics for the form f_4*(Shimao_LN, Modern Tibetan; Neolithic to Historic East Asians, Mbuti)* showed the additional shared derived alleles from source populations except for inland Neolithic northern East Asian from Shaanxi or Inner Mongolia or their related ancestral populations.

Here, overlapping SNP loci included in the Affymetrix Human Origins platform among four analyzed populations were used. We used the genetic variation of Mbuti as the outgroup. Red asterisk point meant the significant value (Absolute value of Z-scores larger than three or equal to three) observed in the symmetry-f_4 statistics and green circle point denoted the non-significant f_4-statistic values (Absolute value of Z-scores less than three). All ancient East Asians were listed along the Y-axis and f_4 values were labeled along the X-axis. All results were faceted or grouped via Tibetan populations. Significant negative f_4 values indicated that the third population shared more alleles with the second population, and

also means Tibetan obtained additional gene flow from the third source population (or related populations). And the significant positive f_4 value indicated that the third population shared more derived alleles with the first population. The value of f_4-statistics equal to zero was marked as the blue dash line. The bar indicated three standard errors.

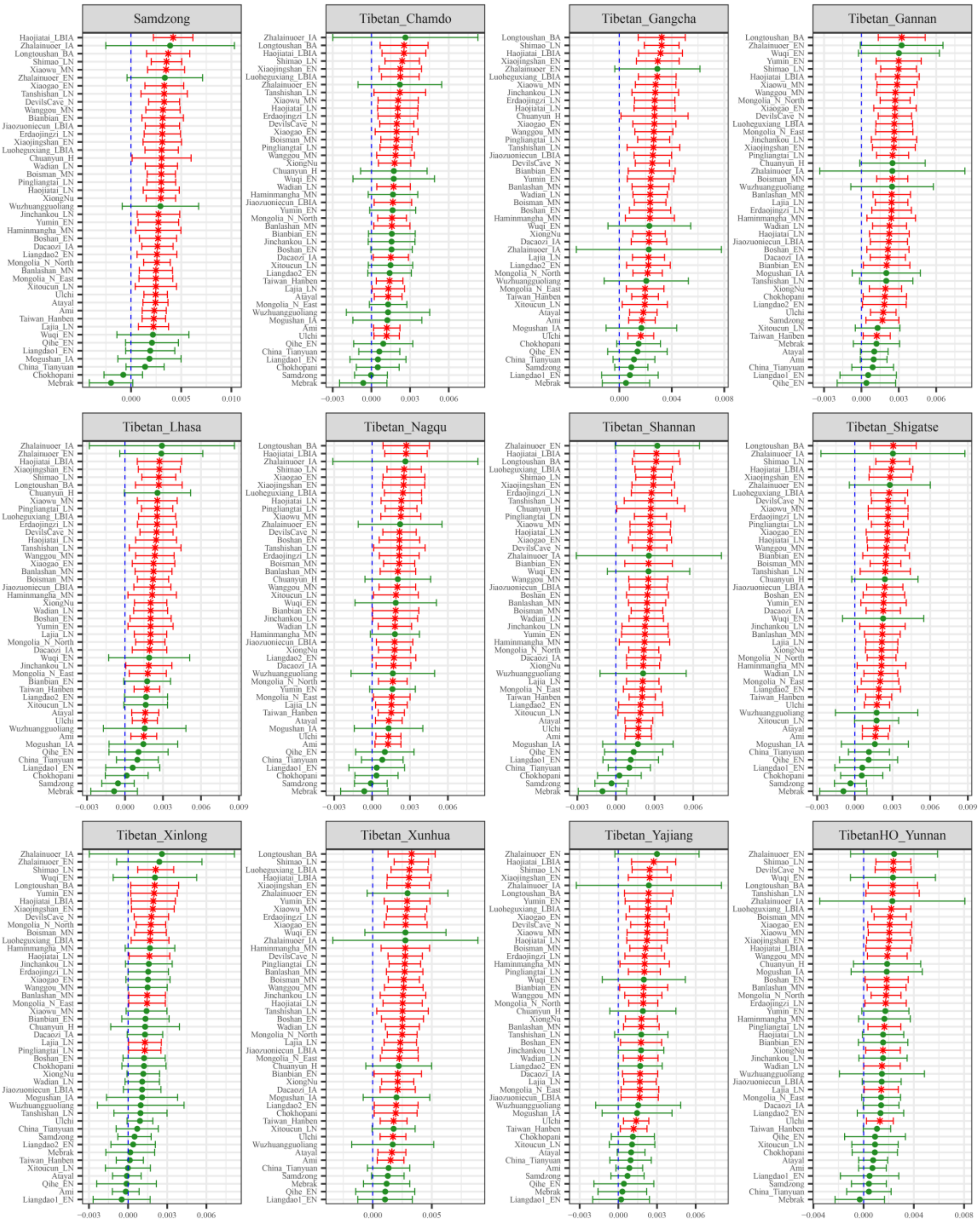

Figure S74. Results of affinity-f_4 statistics for the form f_4*(Miaozigou_MN, Modern Tibetan; Neolithic to Historic East Asians, Mbuti)* showed the additional shared derived alleles from source populations except for inland Neolithic northern East Asian from Shaanxi or Inner Mongolia or their related ancestral populations.

Here, overlapping SNP loci included in the Affymetrix Human Origins platform among four analyzed populations were used. We used the genetic variation of Mbuti as the outgroup. Red asterisk point meant the significant value (Absolute value of Z-scores larger than three or equal to three) observed in the symmetry-f_4 statistics and green circle point denoted the non-significant f_4-statistic values (Absolute value of Z-scores less than three). All ancient East Asians were listed along the Y-axis and f_4 values were labeled along the X-axis. All results were faceted or grouped via Tibetan populations. Significant

negative f_4 values indicated that the third population shared more alleles with the second population, and also means Tibetan obtained additional gene flow from the third source population (or related populations). And the significant positive f_4 value indicated that the third population shared more derived alleles with the first population. The value of f_4-statistics equal to zero was marked as the blue dash line. The bar indicated three standard errors.

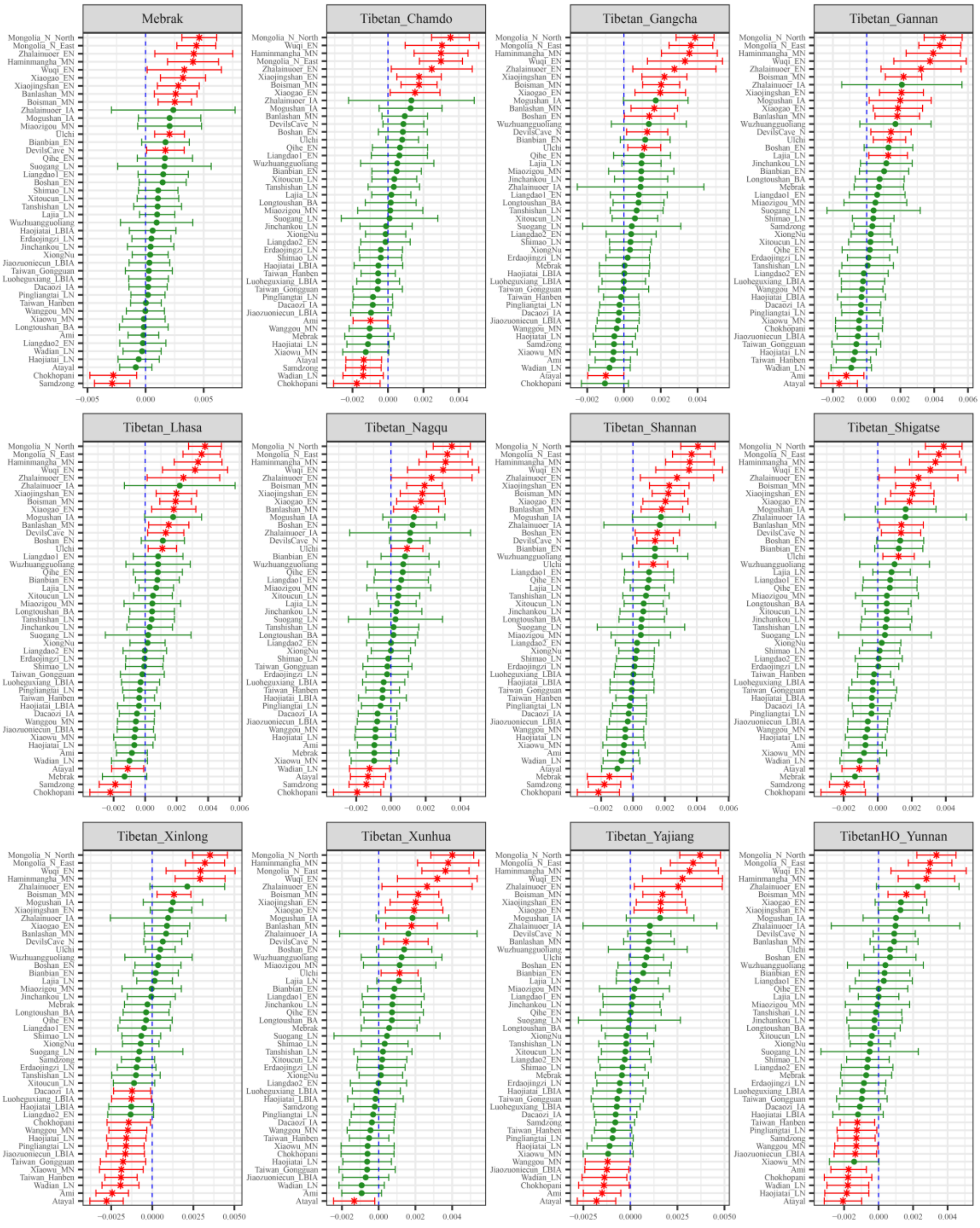

Figure S75. Results of affinity-f_4 statistics for the form f_4*(Yumin_EN, Modern Tibetan; Neolithic to Historic East Asians, Mbuti)* showed the additional shared derived alleles from source populations except for inland Neolithic northern East Asian from Shaanxi or Inner Mongolia or their related ancestral populations.

Here, overlapping SNP loci included in the Affymetrix Human Origins platform among four analyzed populations were used. We used the genetic variation of Mbuti as the outgroup. Red asterisk point meant the significant value (Absolute value of Z-scores larger than three or equal to three) observed in the symmetry-f_4 statistics and green circle point denoted the non-significant f_4-statistic values (Absolute value of Z-scores less than three). All ancient East Asians were listed along the Y-axis and f_4 values were

labeled along the X-axis. All results were faceted or grouped via Tibetan populations. Significant negative f_4 values indicated that the third population shared more alleles with the second population, and also means Tibetan obtained additional gene flow from the third source population (or related populations). And the significant positive f_4 value indicated that the third population shared more derived alleles with the first population. The value of f_4-statistics equal to zero was marked as the blue dash line. The bar indicated three standard errors.

Additional gene flow events when we assumed that Tibetans' direct ancestor is inland Neolithic to Iron Age northern East Asian from upper Yellow River Basin or their related ancestral populations

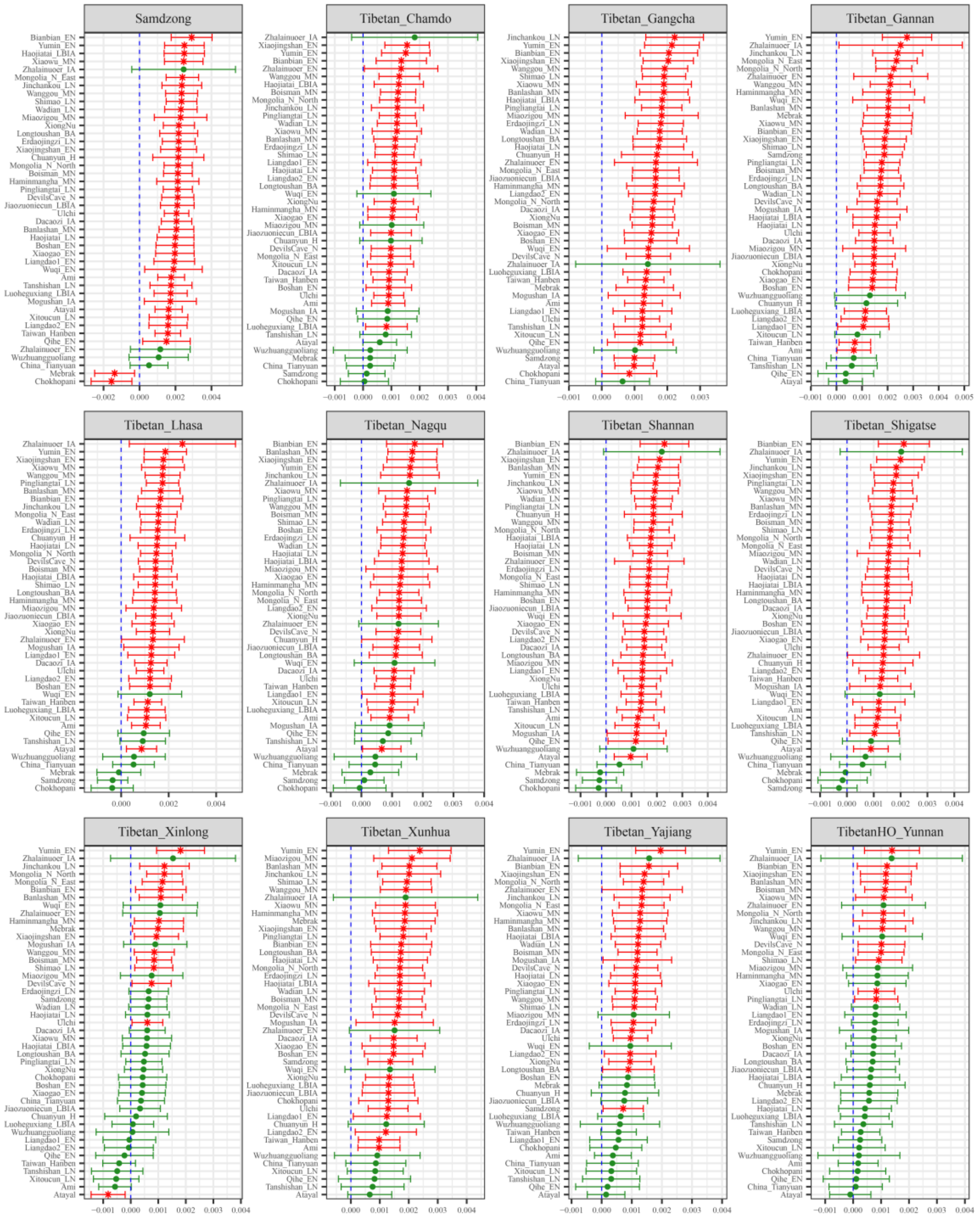

Figure S76. Results of affinity-f_4 statistics for the form f_4*(Lajia_LN, Modern Tibetan; Neolithic to Historic East Asians, Mbuti)* showed the additional shared derived alleles from source populations except for inland Neolithic northern East Asian from Shaanxi or Inner Mongolia or their related ancestral populations.

Here, overlapping SNP loci included in the Affymetrix Human Origins platform among four analyzed populations were used. We used the genetic variation of Mbuti as the outgroup. Red asterisk point meant the significant value (Absolute value of Z-scores larger than three or equal to three) observed in the symmetry-f_4 statistics and green circle point denoted the non-significant f_4-statistic values (Absolute value of Z-scores less than three). All ancient East Asians were listed along the Y-axis and f_4 values were labeled along the X-axis. All results were faceted or grouped via Tibetan populations. Significant negative f_4 values indicated that the third population shared more alleles with the second population, and also means Tibetan obtained additional gene flow from the third source population (or related

populations). And the significant positive f_4 value indicated that the third population shared more derived alleles with the first population. The value of f_4-statistics equal to zero was marked as the blue dash line. The bar indicated three standard errors.

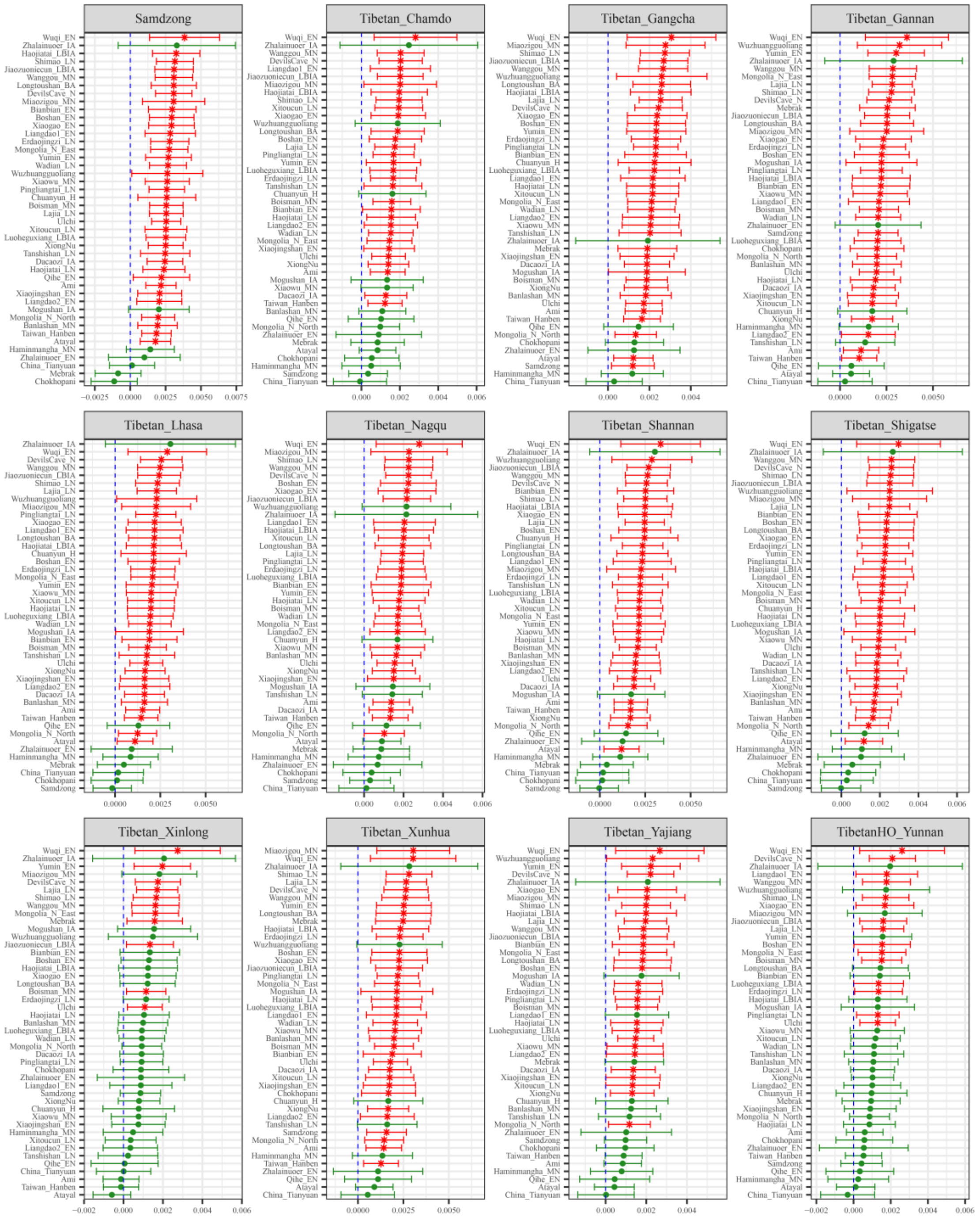

Figure S77. Results of affinity-f_4 statistics for the form *f_4(Jinchankou_LN, Modern Tibetan; Neolithic to Historic East Asians, Mbuti)* showed the additional shared derived alleles from source populations except for inland Neolithic northern East Asian from Shaanxi or Inner Mongolia or their related ancestral populations.

Here, overlapping SNP loci included in the Affymetrix Human Origins platform among four analyzed populations were used. We used the genetic variation of Mbuti as the outgroup. Red asterisk point meant the significant value (Absolute value of Z-scores larger than three or equal to three) observed in the symmetry-f_4 statistics and green circle point denoted the non-significant f_4-statistic values (Absolute value of Z-scores less than three). All ancient East Asians were listed along the Y-axis and f_4 values were labeled along the X-axis. All results were faceted or grouped via Tibetan populations. Significant negative f_4 values indicated that the third population shared more alleles with the second population, and also means Tibetan obtained additional gene flow from the third source population (or related

populations). And the significant positive f_4 value indicated that the third population shared more derived alleles with the first population. The value of f_4-statistics equal to zero was marked as the blue dash line. The bar indicated three standard errors.

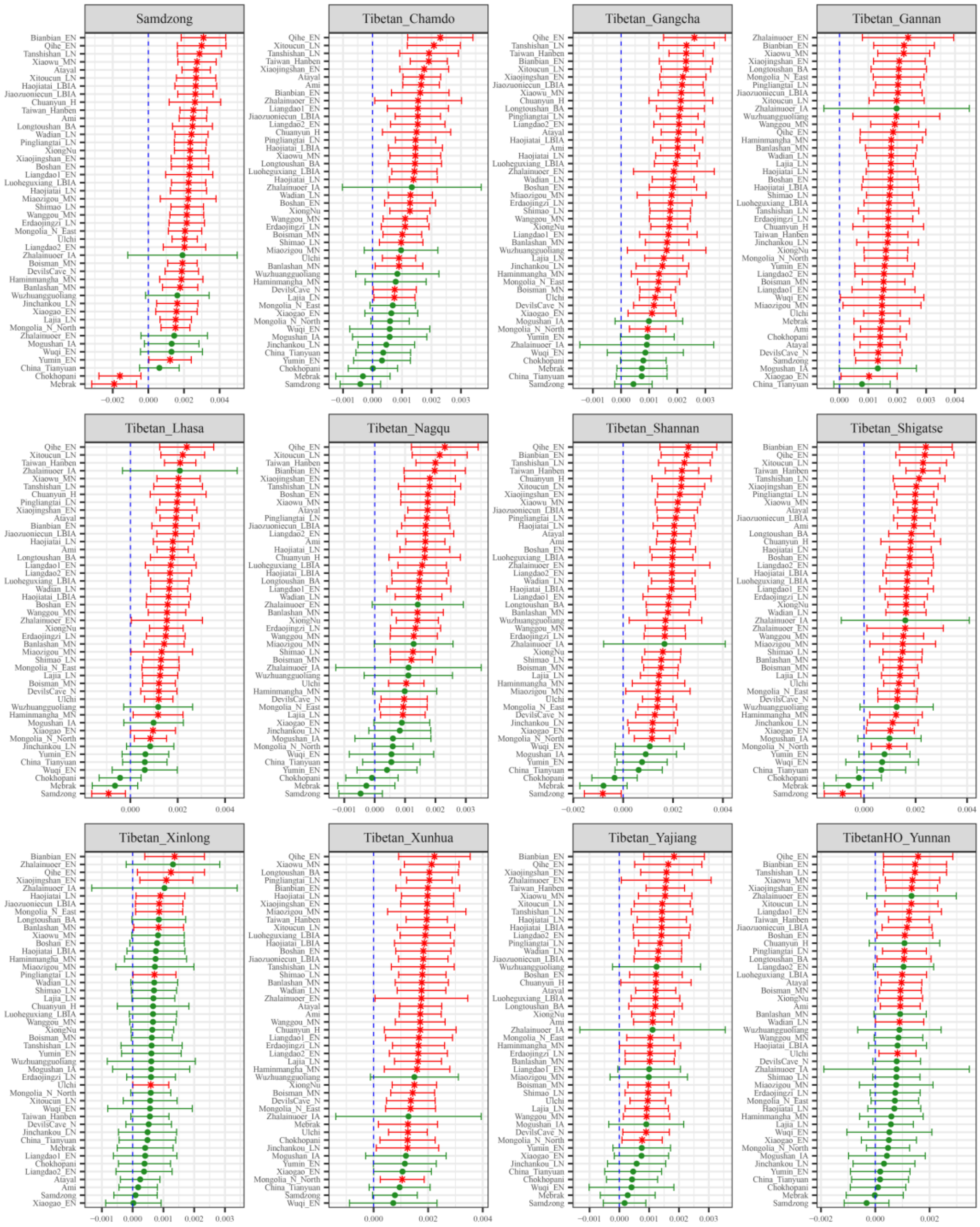

Figure S78. Results of affinity-f_4 statistics for the form f_4*(Dacaozi_IA, Modern Tibetan; Neolithic to Historic East Asians, Mbuti)* showed the additional shared derived alleles from source populations except for inland Neolithic northern East Asian from Shaanxi or Inner Mongolia or their related ancestral populations.

Here, overlapping SNP loci included in the Affymetrix Human Origins platform among four analyzed populations were used. We used the genetic variation of Mbuti as the outgroup. Red asterisk point meant the significant value (Absolute value of Z-scores larger than three or equal to three) observed in the symmetry-f_4 statistics and green circle point denoted the non-significant f_4-statistic values (Absolute value of Z-scores less than three). All ancient East Asians were listed along the Y-axis and f_4 values were labeled along the X-axis. All results were faceted or grouped via Tibetan populations. Significant negative f_4 values indicated that the third population shared more alleles with the second population, and

also means Tibetan obtained additional gene flow from the third source population (or related populations). And the significant positive f_4 value indicated that the third population shared more derived alleles with the first population. The value of f_4-statistics equal to zero was marked as the blue dash line. The bar indicated three standard errors.

Additional gene flow events when we assumed that Tibetans' direct ancestor is Liao River ancients or their related ancestral populations

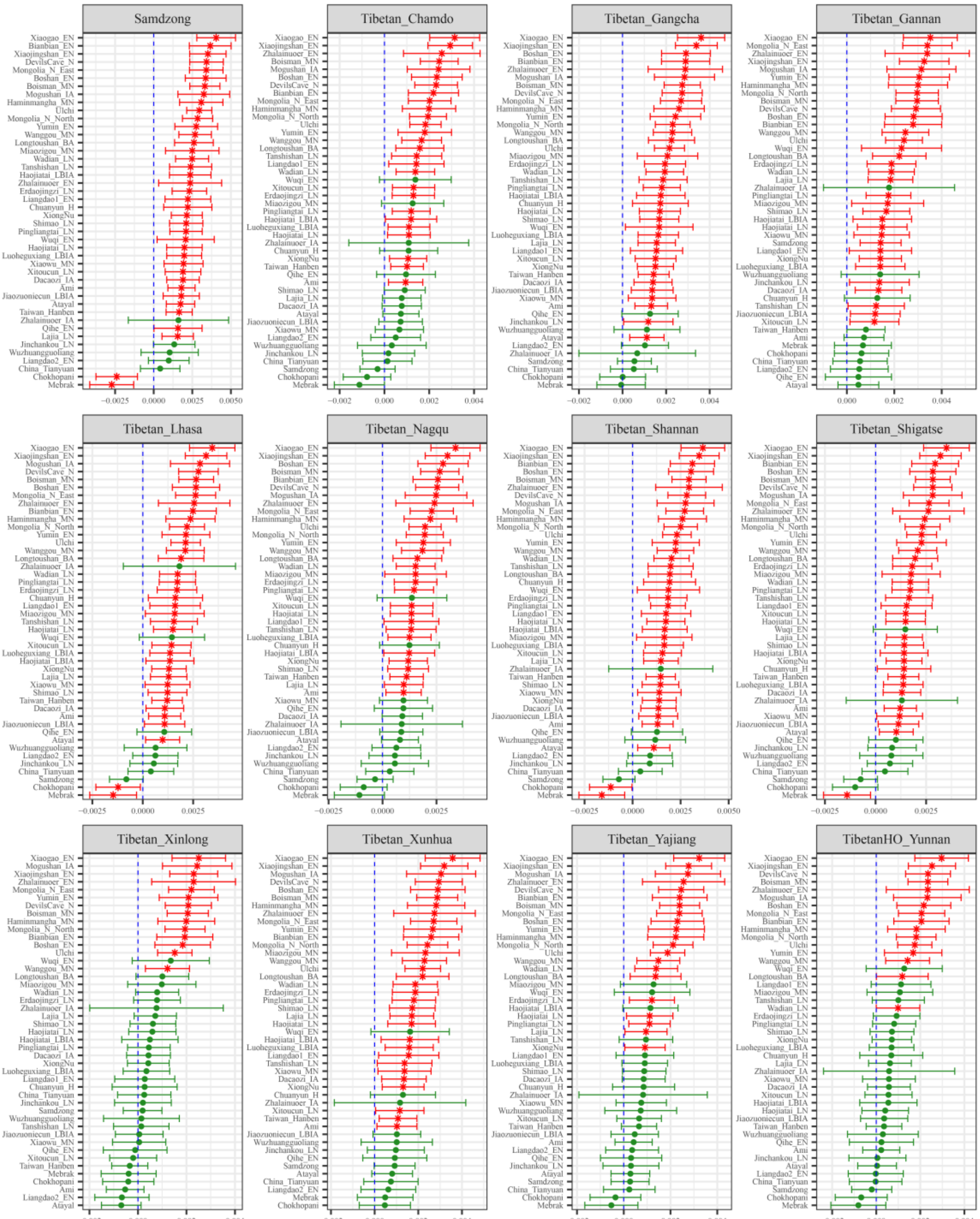

Figure S79. Results of affinity-f_4 statistics for the form f_4*(Banhashan_MN, Modern Tibetan; Neolithic to Historic East Asians, Mbuti)* showed the additional shared derived alleles from source populations except for ancestral populations from Liao River Basin.

Here, overlapping SNP loci included in the Affymetrix Human Origins platform among four analyzed populations were used. We used the genetic variation of Mbuti as the outgroup. Red asterisk point meant the significant value (Absolute value of Z-scores larger than three or equal to three) observed in the symmetry-f_4 statistics and green circle point denoted the non-significant f_4-statistic values (Absolute

value of Z-scores less than three). All ancient East Asians were listed along the Y-axis and f_4 values were labeled along the X-axis. All results were faceted or grouped via Tibetan populations. Significant negative f_4 values indicated that the third population shared more alleles with the second population, and also means Tibetan obtained additional gene flow from the third source population (or related populations). And the significant positive f_4 value indicated that the third population shared more derived alleles with the first population. The value of f_4-statistics equal to zero was marked as the blue dash line. The bar indicated three standard errors.

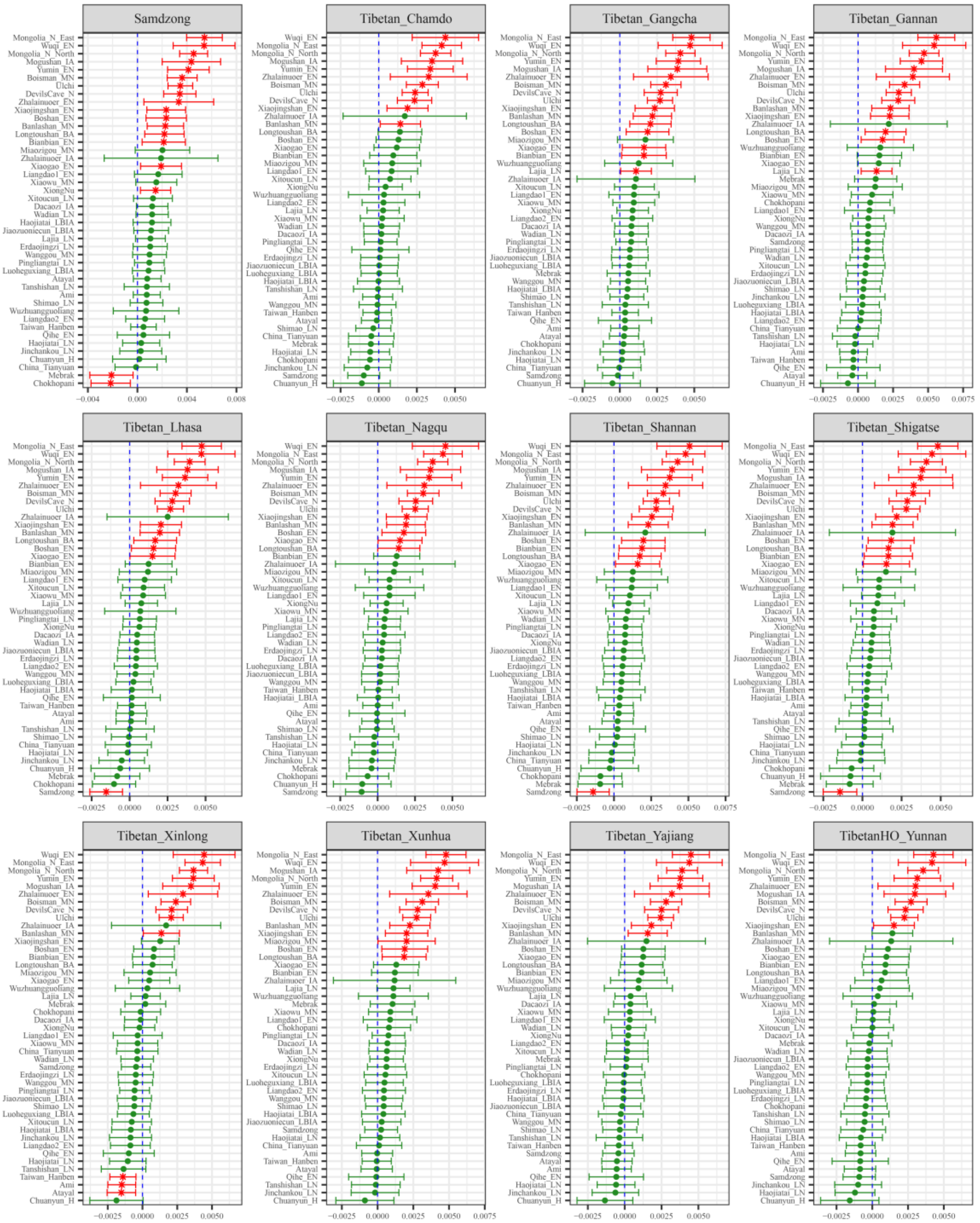

Figure S80. Results of affinity-f_4 statistics for the form f_4*(Haminmangha_MN, Modern Tibetan; Neolithic to Historic East Asians, Mbuti)* showed the additional shared derived alleles from source populations except for ancestral populations from Liao River Basin.

Here, overlapping SNP loci included in the Affymetrix Human Origins platform among four analyzed populations were used. We used the genetic variation of Mbuti as the outgroup. Red asterisk point meant the significant value (Absolute value of Z-scores larger than three or equal to three) observed in the symmetry-f_4 statistics and green circle point denoted the non-significant f_4-statistic values (Absolute

value of Z-scores less than three). All ancient East Asians were listed along the Y-axis and f_4 values were labeled along the X-axis. All results were faceted or grouped via Tibetan populations. Significant negative f_4 values indicated that the third population shared more alleles with the second population, and also means Tibetan obtained additional gene flow from the third source population (or related populations). And the significant positive f_4 value indicated that the third population shared more derived alleles with the first population. The value of f_4-statistics equal to zero was marked as the blue dash line. The bar indicated three standard errors.
