# Supplementary material for: Peopling History of the Tibetan Plateau and Multiple Waves of Admixture of Tibetans Inferred From Both Ancient and Modern Genome-Wide Data
Source: Front Genet. 2021 Sep 3;12:725243. doi: 10.3389/fgene.2021.725243 (PMC8506211; doi:10.3389/fgene.2021.725243)
Supplement: Supplementary file 4 [file Data_Sheet_4.PDF]

Supplementary Figures S81-S107

Peopling history of Tibet Plateau and multiple waves of admixture of Tibetans inferred from both ancient and modern genome-wide data

Guanglin He[1,2,*,#], Mengge Wang[3,*], Xing Zou[3,*], Pengyu Chen[4], Zheng Wang[2], Yan Liu[5], Hongbin Yao[6], Lan-Hai Wei[2], Renkuan Tang[7,#], Chuan-Chao Wang[2,#], Hui-Yuan Yeh[1,#]

[1]School of Humanities, Nanyang Technological University, Nanyang, 639798, Singapore
[2]Department of Anthropology and Ethnology, Institute of Anthropology, National Institute for Data Science in Health and Medicine, and School of Life Sciences, Xiamen University, Xiamen, 361005, China
[3]Institute of Forensic Medicine, West China School of Basic Science and Forensic Medicine, Sichuan University, Chengdu, 610041, China
[4]Center of Forensic Expertise, Affiliated hospital of Zunyi Medical University, Zunyi, Guizhou, 563099, China
[5]School of Basic Medical Sciences, North Sichuan Medical College, Nanchong, Sichuan, 637100, China
[6]Key Laboratory of Evidence Science of Gansu Province, Gansu Institute of Political Science and Law, Lanzhou, 730070, China
[7]Department of Forensic Medicine, College of Basic Medicine, Chongqing Medical University, Chongqing, 400016, China
*These authors contributed equally to this work and should be considered co-first authors.
#Corresponding author
Guanglin He
Affiliation: School of Humanities, Nanyang Technological University, Nanyang, 639798, Singapore; Department of Anthropology and Ethnology, Institute of Anthropology, National Institute for Data Science in Health and Medicine, Xiamen University, Xiamen, China.
E-mail: Guanglinhescu@163.com
Renkuan Tang
Department of Forensic Medicine, College of Basic Medicine, Chongqing Medical University, Chongqing, China
E-mail: renktang2012@163.com
Chuan-Chao Wang
Affiliation: Department of Anthropology and Ethnology, Institute of Anthropology, National Institute for Data Science in Health and Medicine, Xiamen University, Xiamen, China.
E-mail: wang@xmu.edu.cn
Hui-Yuan Yeh
Affiliation: School of Humanities, Nanyang Technological University, Nanyang, 639798, Singapore
E-mail: hyyeh@ntu.edu.sg

Contents of Supplementary Figures S50-S107

Figure S81. Results of affinity-f_4 statistics for the form f_4*(Erdaojingzi_LN, Modern Tibetan; Neolithic to Historic East Asians, Mbuti)* showed the additional shared derived alleles from source populations except for ancestral populations from Liao River Basin. 1

Figure S82. Results of affinity-f_4 statistics for the form f_4*(Longtoushan_BA, Modern Tibetan; Neolithic to Historic East Asians, Mbuti)* showed the additional shared derived alleles from source populations except for ancestral populations from Liao River Basin. 2

Additional gene flow events when we assumed that Tibetans' direct ancestor is ancestral populations from Amur River Basin 4

Figure S83. Results of affinity-f_4 statistics for the form f_4*(Wuqi_EN, Modern Tibetan; Neolithic to Historic East Asians, Mbuti)* showed the additional shared derived alleles from source populations except for ancestral populations from Amur River Basin. 4

Figure S84. Results of affinity-f_4 statistics for the form f_4*(Zhalainuoer_EN, Modern Tibetan; Neolithic to Historic East Asians, Mbuti)* showed the additional shared derived alleles from source populations except for ancestral populations from Amur River Basin. 5

Figure S85. Results of affinity-f_4 statistics for the form f_4*(Boisman_MN, Modern Tibetan; Neolithic to Historic East Asians, Mbuti)* showed the additional shared derived alleles from source populations except for ancestral populations from Amur River Basin. 6

Figure S86. Results of affinity-f_4 statistics for the form f_4*(DevilsCave_N, Modern Tibetan; Neolithic to Historic East Asians, Mbuti)* showed the additional shared derived alleles from source populations except for ancestral populations from Amur River Basin. 7

Figure S87. Results of affinity-f_4 statistics for the form f_4*(Mogushan_IA, Modern Tibetan; Neolithic to Historic East Asians, Mbuti)* showed the additional shared derived alleles from source populations except for ancestral populations from Amur River Basin. 8

Figure S88. Results of affinity-f_4 statistics for the form f_4*(Ulchi, Modern Tibetan; Neolithic to Historic East Asians, Mbuti)* showed the additional shared derived alleles from source populations except for ancestral populations from Amur River Basin. 9

Additional gene flow events when we assumed that Tibetans' direct ancestor is ancestral populations from Nepal 10

Figure S89. Results of affinity-f_4 statistics for the form f_4*(Chokhopani, Modern Tibetan; Neolithic to Historic East Asians, Mbuti)* showed the additional shared derived alleles from source populations except for ancestral populations from Nepal. 10

Figure S90. Results of affinity-f_4 statistics for the form f_4*(Mebrak, Modern Tibetan; Neolithic to Historic East Asians, Mbuti)* showed the additional shared derived alleles from source populations except for ancestral populations from Nepal. 11

Figure S91. Results of affinity-f_4 statistics for the form f_4*(Samdzong, Modern Tibetan; Neolithic to Historic East Asians, Mbuti)* showed the additional shared derived alleles from source populations except for ancestral populations from Nepal. 12

Additional gene flow events when we assumed that Tibetans' direct ancestor is ancestral populations from Mongolia Plateau or Baikal Lake Region 14

Figure S92. Results of affinity-f_4 statistics for the form *f_4(Mongolia_N_East, Modern Tibetan; Neolithic to Historic East Asians, Mbuti)* showed the additional shared derived alleles from source populations except for ancestral populations from Mongolia Plateau or Baikal Lake Region........14
Figure S93. Results of affinity-f_4 statistics for the form *f_4(Mongolia_N_North, Modern Tibetan; Neolithic to Historic East Asians, Mbuti)* showed the additional shared derived alleles from source populations except for ancestral populations from Mongolia Plateau or Baikal Lake Region........15
Figure S94. Results of affinity-f_4 statistics for the form *f_4(Russia_OldBeringSea_Ekven, Modern Tibetan; Neolithic to Historic East Asians, Mbuti)* showed the additional shared derived alleles from source populations except for ancestral populations from Mongolia Plateau or Baikal Lake Region. 16
Figure S95. Results of affinity-f_4 statistics for the form *f_4(Russia_Shamanka_EBA, Modern Tibetan; Neolithic to Historic East Asians, Mbuti)* showed the additional shared derived alleles from source populations except for ancestral populations from Mongolia Plateau or Baikal Lake Region........17
Figure S96. Results of affinity-f_4 statistics for the form *f_4(Russia_Shamanka_Eneolithic, Modern Tibetan; Neolithic to Historic East Asians, Mbuti)* showed the additional shared derived alleles from source populations except for ancestral populations from Mongolia Plateau or Baikal Lake Region. 18
Figure S97. Results of affinity-f_4 statistics for the form *f_4(Russia_UstBelaya_Angara, Modern Tibetan; Neolithic to Historic East Asians, Mbuti)* showed the additional shared derived alleles from source populations except for ancestral populations from Mongolia Plateau or Baikal Lake Region. 19
Figure S98. Results of affinity-f_4 statistics for the form *f_4(Russia_UstIda_EBA, Modern Tibetan; Neolithic to Historic East Asians, Mbuti)* showed the additional shared derived alleles from source populations except for ancestral populations from Mongolia Plateau or Baikal Lake Region........20
Figure S99. Results of affinity-f_4 statistics for the form *f_4(Russia_UstIda_LN, Modern Tibetan; Neolithic to Historic East Asians, Mbuti)* showed the additional shared derived alleles from source populations except for ancestral populations from Mongolia Plateau or Baikal Lake Region........21
Figure S100. Results of affinity-f_4 statistics for the form *f_4(UstBelaya_EBA, Modern Tibetan; Neolithic to Historic East Asians, Mbuti)* showed the additional shared derived alleles from source populations except for ancestral populations from Mongolia Plateau or Baikal Lake Region........22
Figure S101. Results of affinity-f_4 statistics for the form *f_4(XiongNu, Modern Tibetan; Neolithic to Historic East Asians, Mbuti)* showed the additional shared derived alleles from source populations except for ancestral populations from Mongolia Plateau or Baikal Lake Region. 23
Figure S102. Results of affinity-f_4 statistics for the form *f_4(Shirenzigou_IA, Modern Tibetan; Neolithic to Historic East Asians, Mbuti)* showed the additional shared derived alleles from source populations except for ancestral populations from Xinjiang or their related ancestral populations. 24
Figure S103. Results of affinity-f_4 statistics for the form *f_4(Shirenzigou_IA_E, Modern Tibetan; Neolithic to Historic East Asians, Mbuti)* showed the additional shared derived alleles from source populations except for ancestral populations from Xinjiang or their related ancestral populations. 25
Figure S104. Results of affinity-f_4 statistics for the form *f_4(Shirenzigou_IA_W, Modern Tibetan; Neolithic to Historic East Asians, Mbuti)* showed the additional shared derived alleles from source

populations except for ancestral populations from Xinjiang or their related ancestral populations. 26
Figure S105. Admixture graph model of modern highland and lowland Tibetans based on the Human Origin dataset using early Neolithic Boshan people as the source of the second migration into Tibet Plateau. 27
Figure S106. Admixture graph model of modern highland and lowland Tibetans based on the Human Origin dataset using middle Neolithic Xiaowu people as the source of the second migration into Tibet Plateau. 28
Figure S107. Admixture graph model of modern highland and lowland Tibetans based on the Human Origin dataset using late Bronze Age to Iron Age Haojiatai people as the source of the second migration into Tibet Plateau. 29

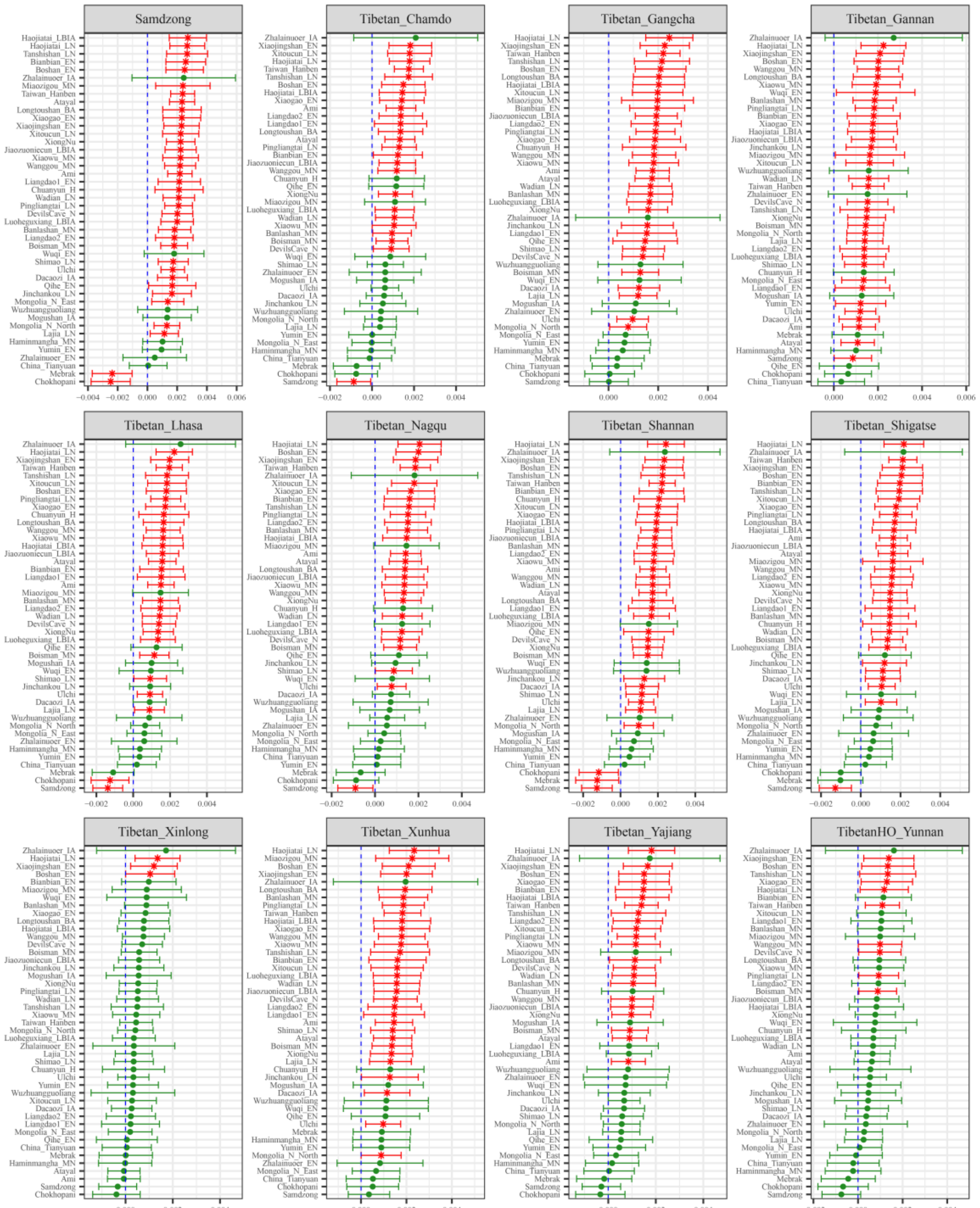

Figure S81. Results of affinity-f_4 statistics for the form f_4*(Erdaojingzi_LN, Modern Tibetan; Neolithic to Historic East Asians, Mbuti)* showed the additional shared derived alleles from source populations except for ancestral populations from Liao River Basin.

Here, overlapping SNP loci included in the Affymetrix Human Origins platform among four analyzed populations were used. We used the genetic variation of Mbuti as the outgroup. Red asterisk point meant the significant value (Absolute value of Z-scores larger than three or equal to three) observed in the symmetry-f_4 statistics and green circle point denoted the non-significant f_4-statistic values (Absolute value of Z-scores less than three). All ancient East Asians were listed along the Y-axis and f_4 values were labeled along the X-axis. All results were faceted or grouped via Tibetan populations. Significant negative f_4 values indicated that the third population shared more alleles with the second population, and also means Tibetan obtained additional gene flow from the third source population (or related populations). And the significant positive f_4 value indicated that the third population shared more derived alleles with the first population. The value of f_4-statistics equal to zero was marked as the blue dash line. The bar indicated three standard errors.

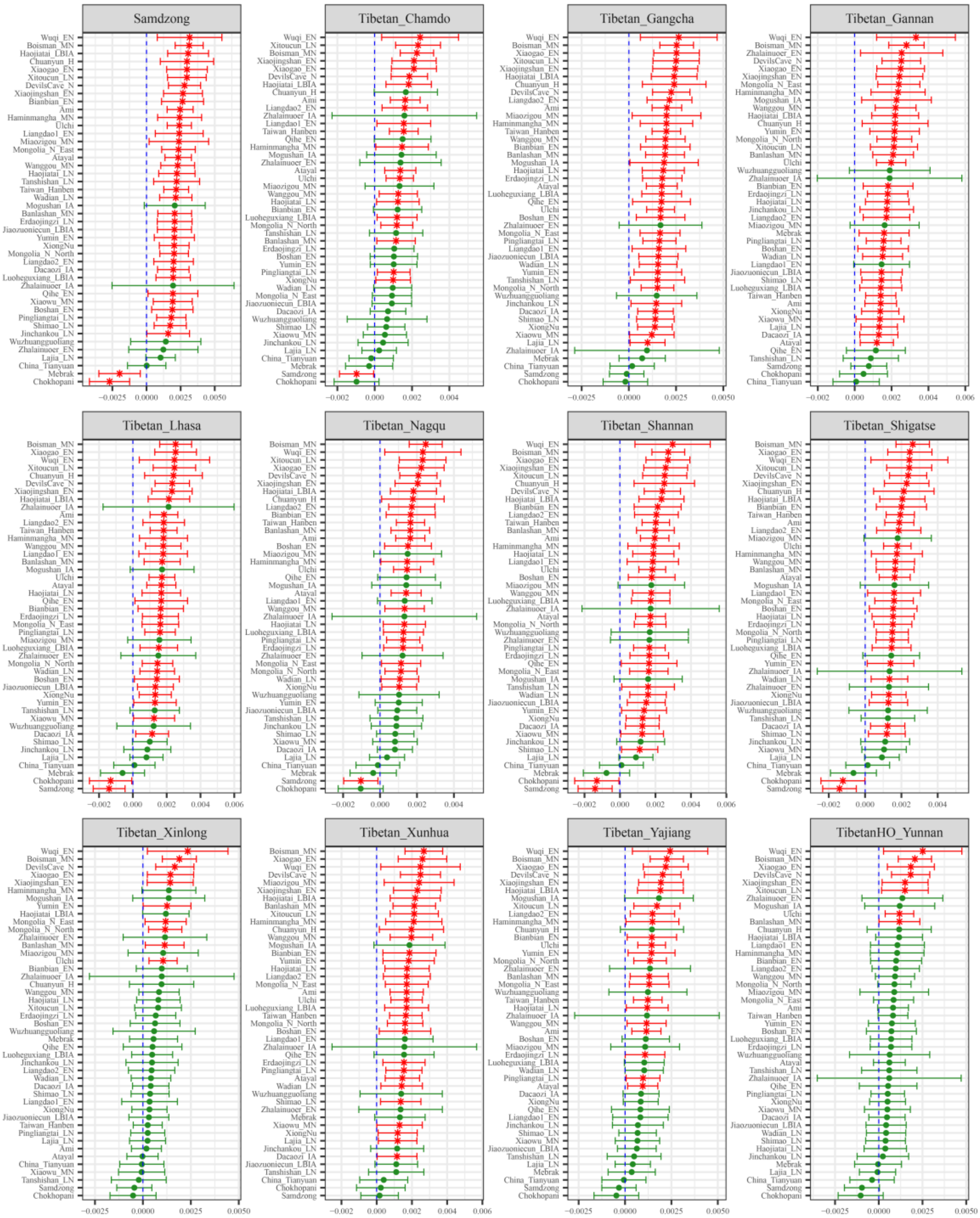

Figure S82. Results of affinity-f_4 statistics for the form f_4(Longtoushan_BA, Modern Tibetan; Neolithic to Historic East Asians, Mbuti) showed the additional shared derived alleles from source populations except for ancestral populations from Liao River Basin.

Here, overlapping SNP loci included in the Affymetrix Human Origins platform among four analyzed populations were used. We used the genetic variation of Mbuti as the outgroup. Red asterisk point meant the significant value (Absolute value of Z-scores larger than three or equal to three) observed in the symmetry-f_4 statistics and green circle point denoted the non-significant f_4-statistic values (Absolute value of Z-scores less than three). All ancient East Asians were listed along the Y-axis and f_4 values were labeled along the X-axis. All results were faceted or grouped via Tibetan populations. Significant negative f_4 values indicated that the third population shared more alleles with the second population, and also means Tibetan obtained additional gene flow from the third source population (or related populations). And the significant positive f_4 value indicated that the third population shared more derived alleles with the first population. The value of f_4-statistics equal to zero was marked as the blue dash line. The bar indicated three standard errors.

Additional gene flow events when we assumed that Tibetans' direct ancestor is ancestral populations from Amur River Basin

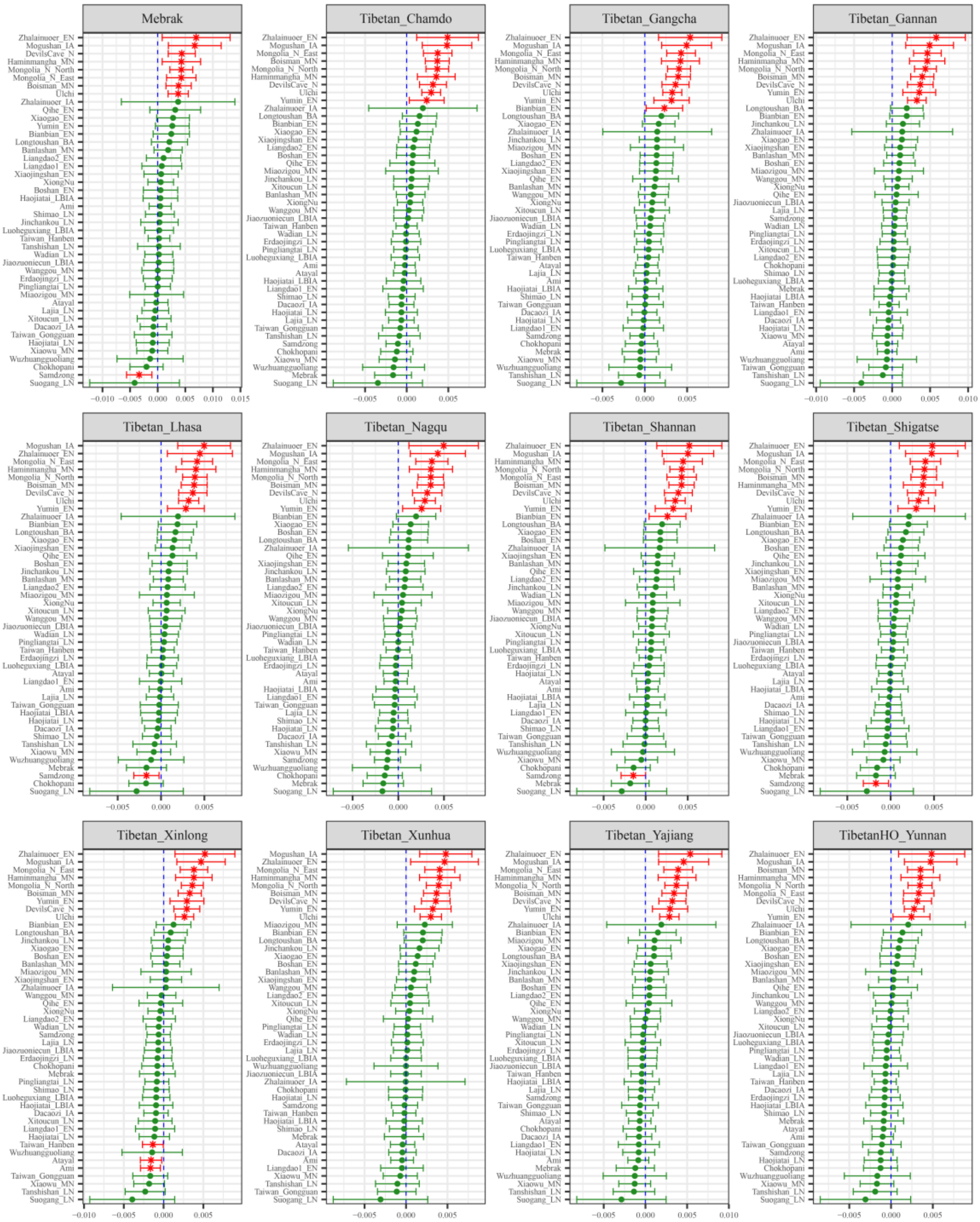

Figure S83. Results of affinity-f_4 statistics for the form f_4*(Wuqi_EN, Modern Tibetan; Neolithic to Historic East Asians, Mbuti)* showed the additional shared derived alleles from source populations except for ancestral populations from Amur River Basin.
Here, overlapping SNP loci included in the Affymetrix Human Origins platform among four analyzed populations were used. We used the genetic variation of Mbuti as the outgroup. Red asterisk point meant the significant value (Absolute value of Z-scores larger than three or equal to three) observed in the symmetry-f_4 statistics and green circle point denoted the non-significant f_4-statistic values (Absolute value of Z-scores less than three). All ancient East Asians were listed along the Y-axis and f_4 values were labeled along the X-axis. All results were faceted or grouped via Tibetan populations. Significant negative f_4 values indicated that the third population shared more alleles with the second population, and also means Tibetan obtained additional gene flow from the third source population (or related populations). And the significant positive f_4 value indicated that the third population shared more derived alleles with the first population. The value of f_4-statistics equal to zero was marked as the blue dash line.

The bar indicated three standard errors.

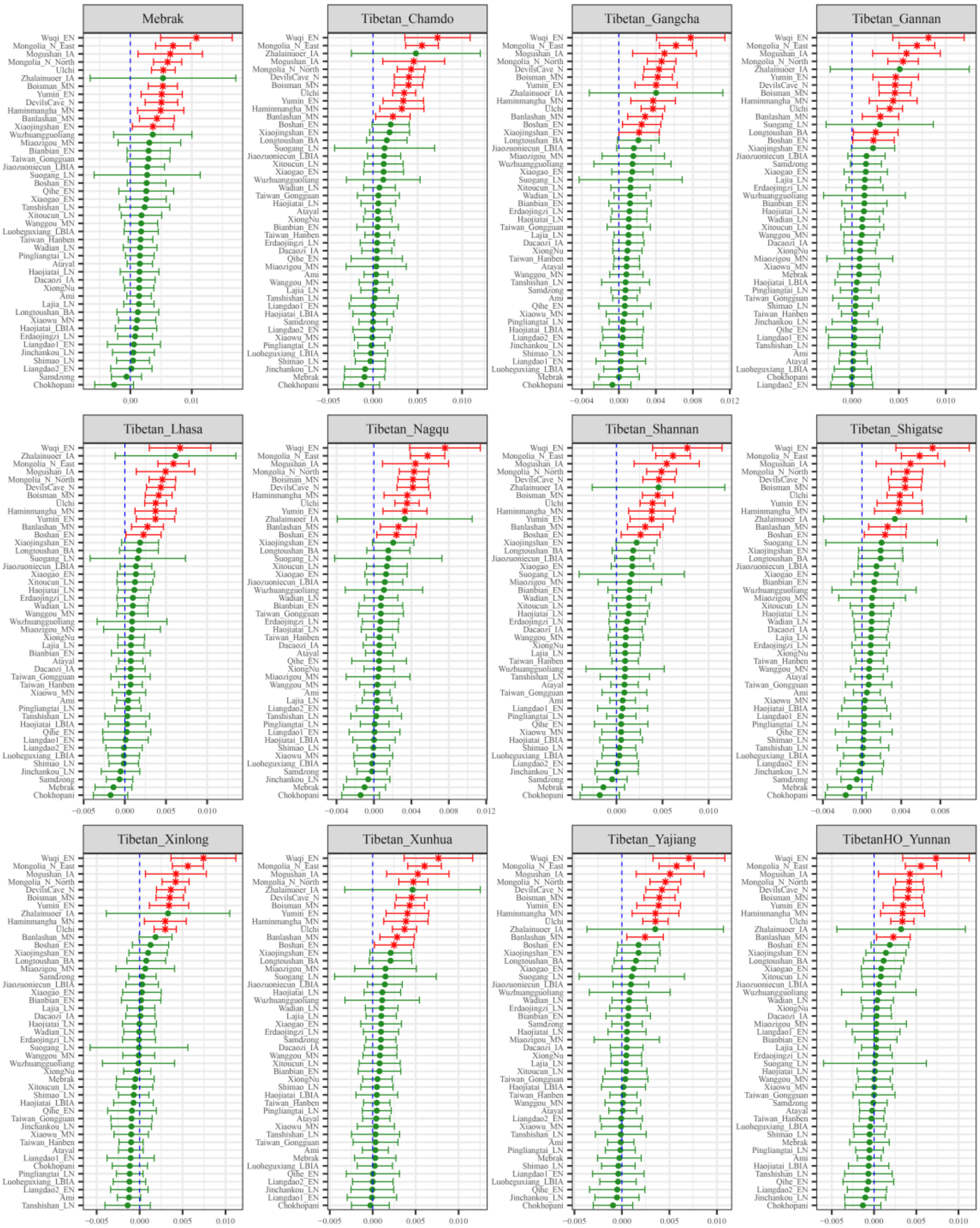

Figure S84. Results of affinity-f_4 statistics for the form f_4(*Zhalainuoer_EN, Modern Tibetan; Neolithic to Historic East Asians, Mbuti*) showed the additional shared derived alleles from source populations except for ancestral populations from Amur River Basin.

Here, overlapping SNP loci included in the Affymetrix Human Origins platform among four analyzed populations were used. We used the genetic variation of Mbuti as the outgroup. Red asterisk point meant the significant value (Absolute value of Z-scores larger than three or equal to three) observed in the symmetry-f_4 statistics and green circle point denoted the non-significant f_4-statistic values (Absolute value of Z-scores less than three). All ancient East Asians were listed along the Y-axis and f_4 values were labeled along the X-axis. All results were faceted or grouped via Tibetan populations. Significant negative f_4 values indicated that the third population shared more alleles with the second population, and also means Tibetan obtained additional gene flow from the third source population (or related populations). And the significant positive f_4 value indicated that the third population shared more derived alleles with the first population. The value of f_4-statistics equal to zero was marked as the blue dash line.

The bar indicated three standard errors.

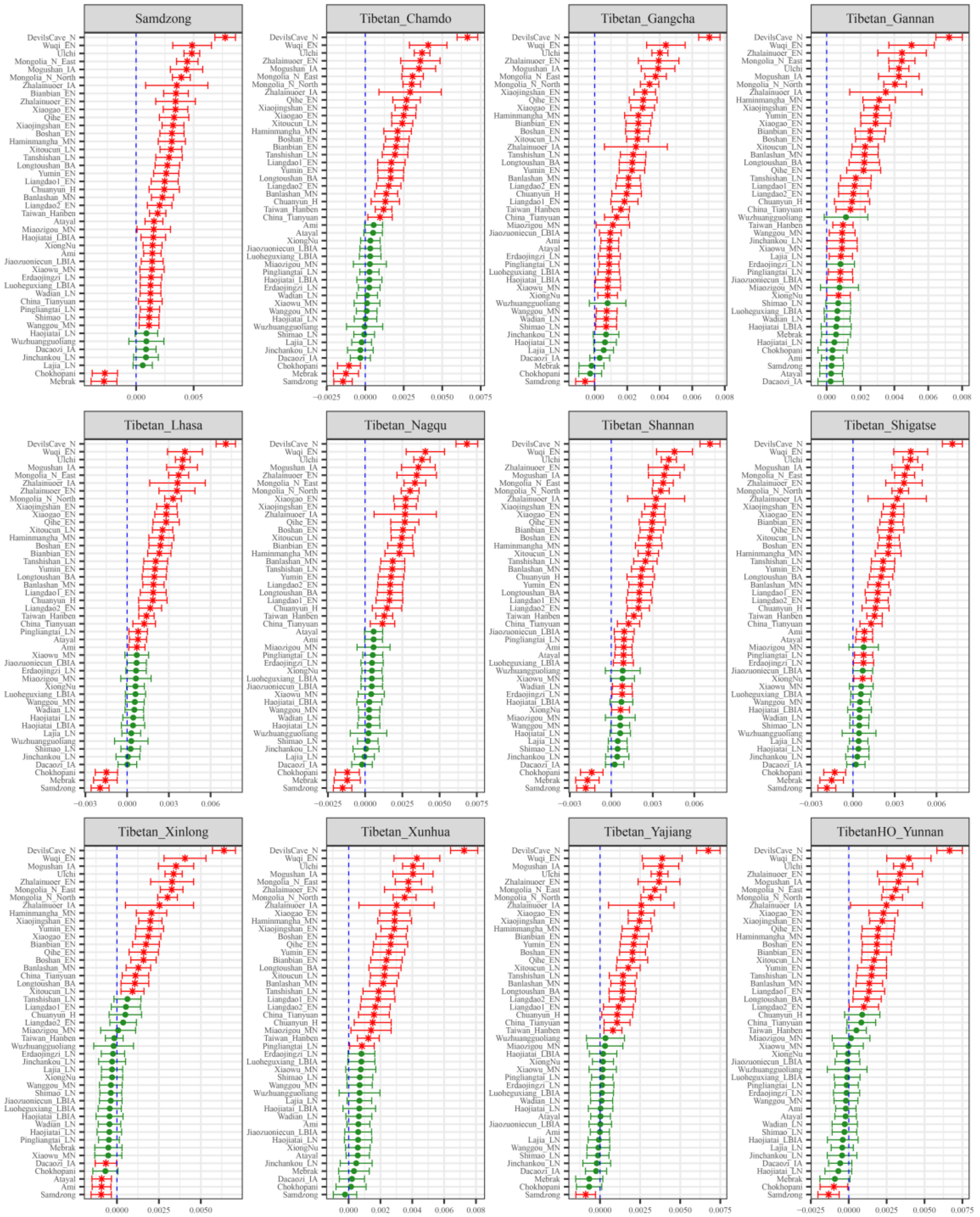

Figure S85. Results of affinity-f_4 statistics for the form f_4*(Boisman_MN, Modern Tibetan; Neolithic to Historic East Asians, Mbuti)* showed the additional shared derived alleles from source populations except for ancestral populations from Amur River Basin.

Here, overlapping SNP loci included in the Affymetrix Human Origins platform among four analyzed populations were used. We used the genetic variation of Mbuti as the outgroup. Red asterisk point meant the significant value (Absolute value of Z-scores larger than three or equal to three) observed in the symmetry-f_4 statistics and green circle point denoted the non-significant f_4-statistic values (Absolute value of Z-scores less than three). All ancient East Asians were listed along the Y-axis and f_4 values were labeled along the X-axis. All results were faceted or grouped via Tibetan populations. Significant negative f_4 values indicated that the third population shared more alleles with the second population, and also means Tibetan obtained additional gene flow from the third source population (or related populations). And the significant positive f_4 value indicated that the third population shared more derived alleles with the first population. The value of f_4-statistics equal to zero was marked as the blue dash line.

The bar indicated three standard errors.

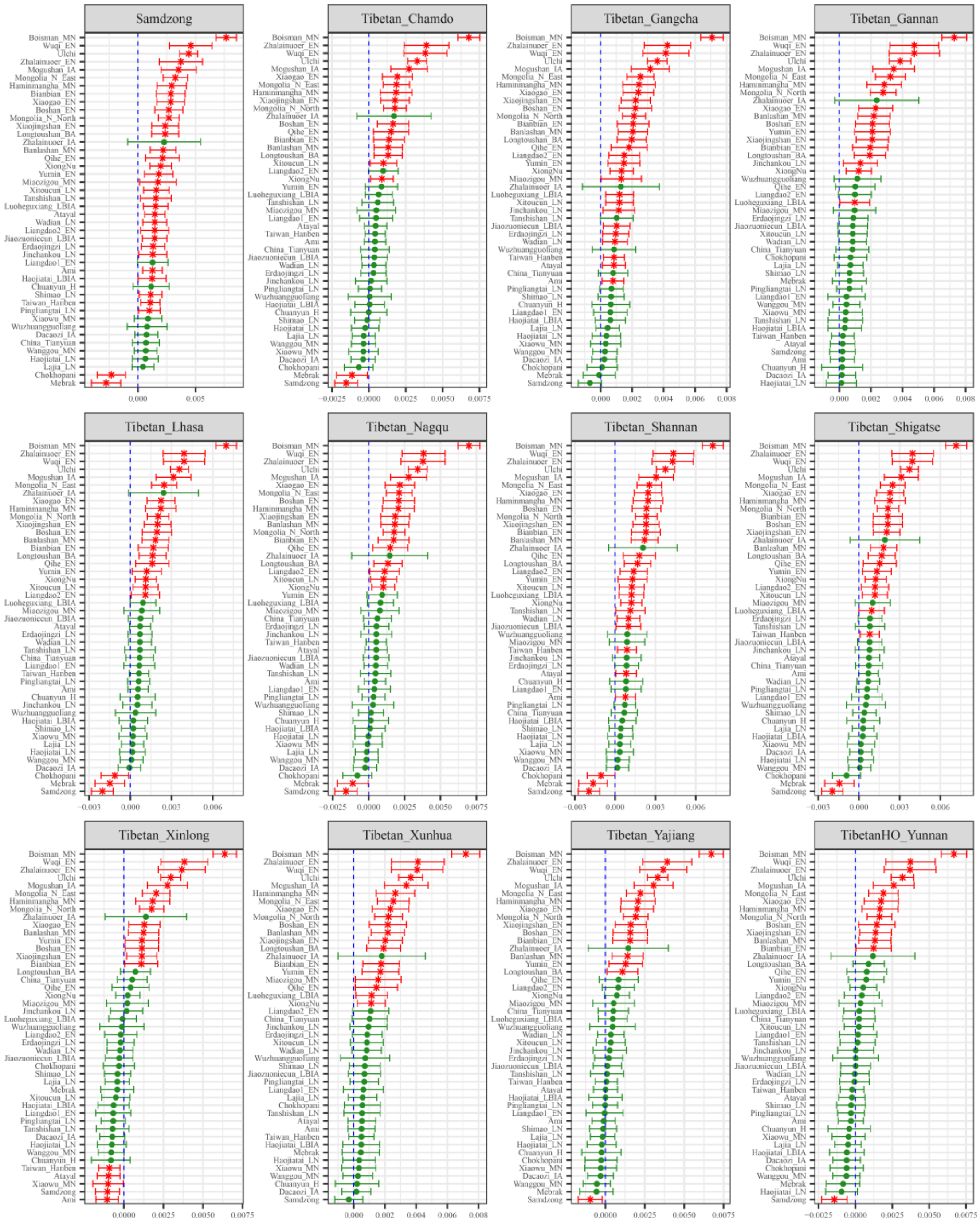

Figure S86. Results of affinity-f_4 statistics for the form f_4*(DevilsCave_N, Modern Tibetan; Neolithic to Historic East Asians, Mbuti)* showed the additional shared derived alleles from source populations except for ancestral populations from Amur River Basin.

Here, overlapping SNP loci included in the Affymetrix Human Origins platform among four analyzed populations were used. We used the genetic variation of Mbuti as the outgroup. Red asterisk point meant the significant value (Absolute value of Z-scores larger than three or equal to three) observed in the symmetry-f_4 statistics and green circle point denoted the non-significant f_4-statistic values (Absolute value of Z-scores less than three). All ancient East Asians were listed along the Y-axis and f_4 values were labeled along the X-axis. All results were faceted or grouped via Tibetan populations. Significant negative f_4 values indicated that the third population shared more alleles with the second population, and also means Tibetan obtained additional gene flow from the third source population (or related populations). And the significant positive f_4 value indicated that the third population shared more derived alleles with the first population. The value of f_4-statistics equal to zero was marked as the blue dash line.

The bar indicated three standard errors.

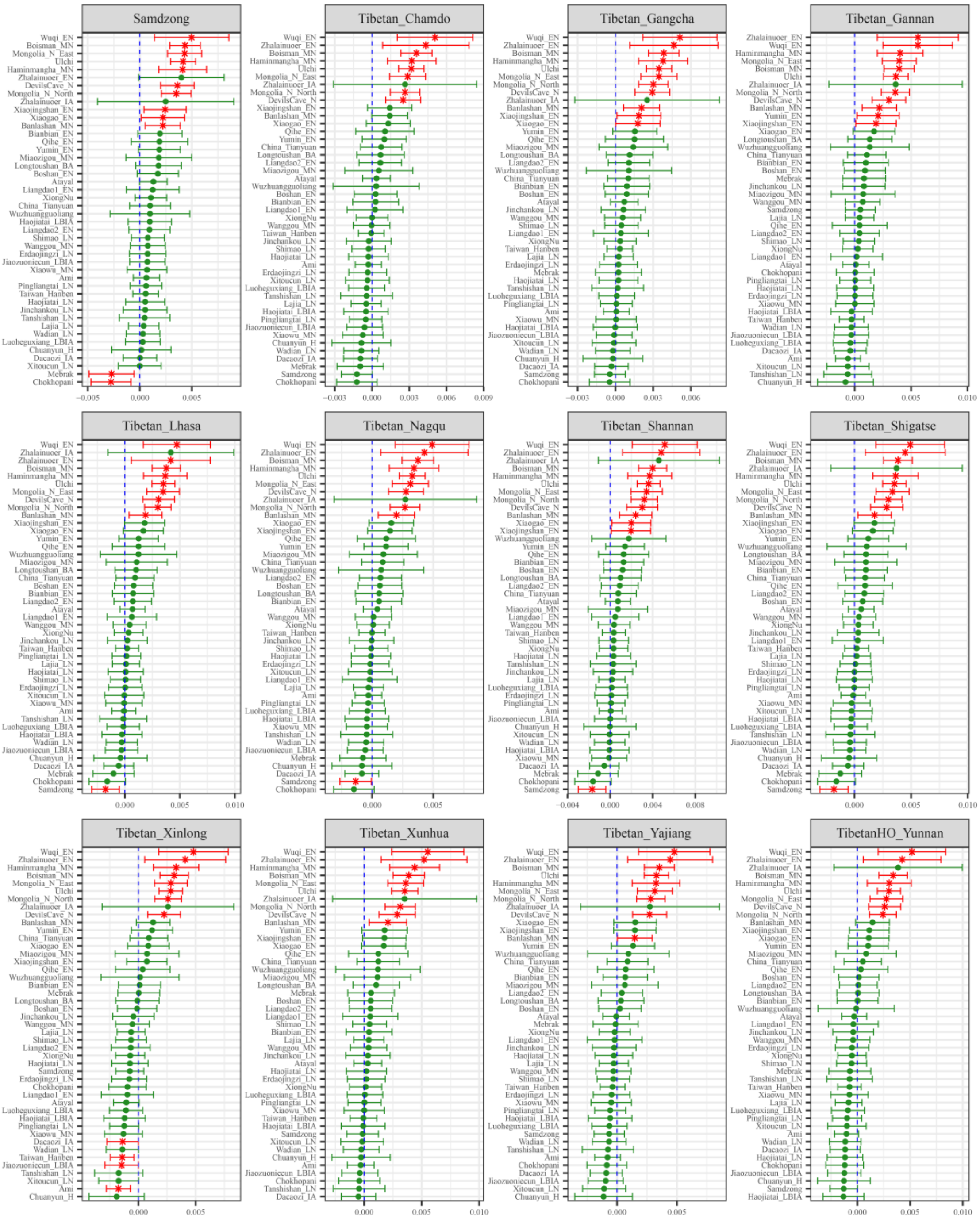

Figure S87. Results of affinity-f_4 statistics for the form f_4*(Mogushan_IA, Modern Tibetan; Neolithic to Historic East Asians, Mbuti)* showed the additional shared derived alleles from source populations except for ancestral populations from Amur River Basin.

Here, overlapping SNP loci included in the Affymetrix Human Origins platform among four analyzed populations were used. We used the genetic variation of Mbuti as the outgroup. Red asterisk point meant the significant value (Absolute value of Z-scores larger than three or equal to three) observed in the symmetry-f_4 statistics and green circle point denoted the non-significant f_4-statistic values (Absolute value of Z-scores less than three). All ancient East Asians were listed along the Y-axis and f_4 values were labeled along the X-axis. All results were faceted or grouped via Tibetan populations. Significant negative f_4 values indicated that the third population shared more alleles with the second population, and also means Tibetan obtained additional gene flow from the third source population (or related populations). And the significant positive f_4 value indicated that the third population shared more derived alleles with the first population. The value of f_4-statistics equal to zero was marked as the blue dash line.

The bar indicated three standard errors.

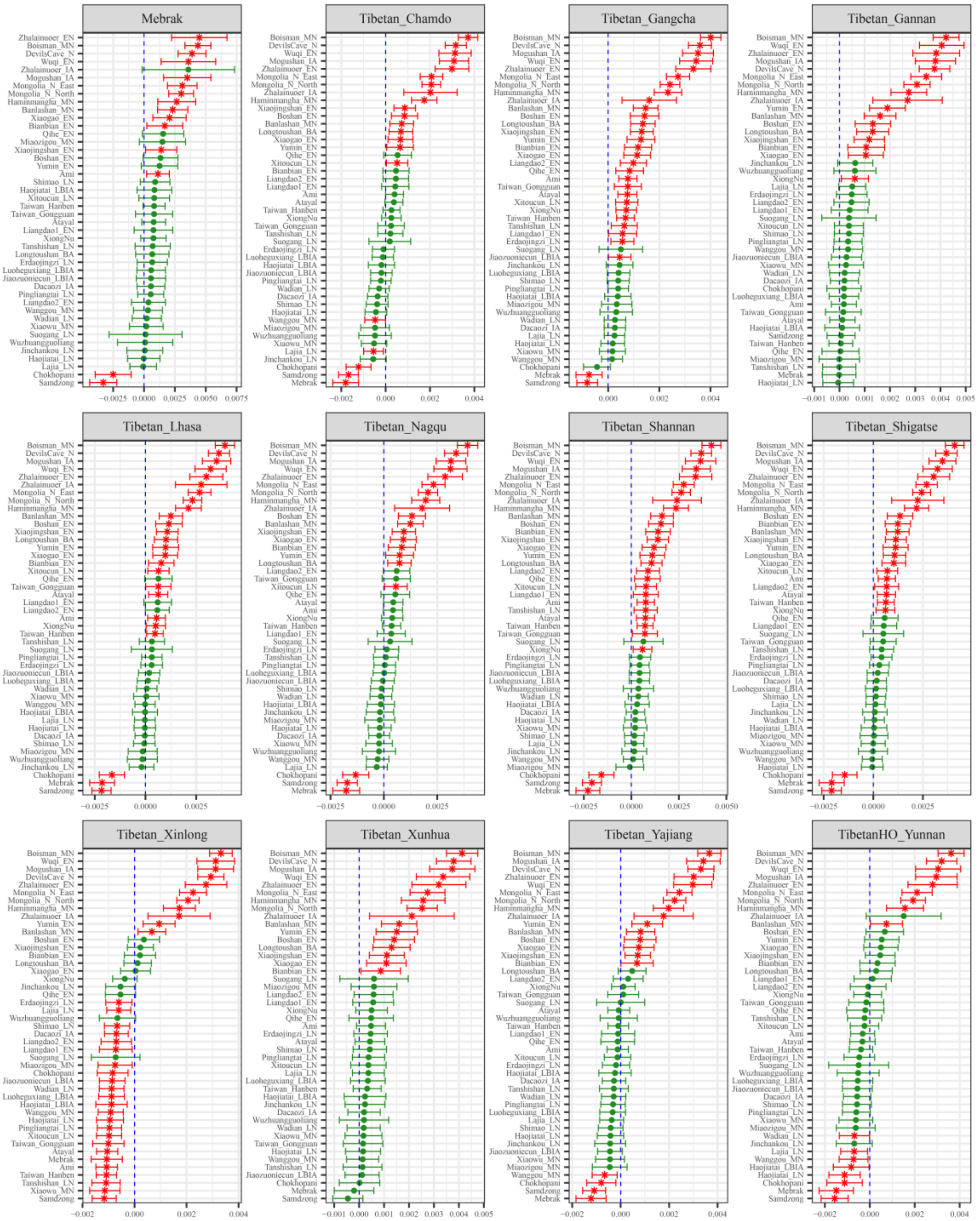

Figure S88. Results of affinity-f_4 statistics for the form f_4*(Ulchi, Modern Tibetan; Neolithic to Historic East Asians, Mbuti)* showed the additional shared derived alleles from source populations except for ancestral populations from Amur River Basin.

Here, overlapping SNP loci included in the Affymetrix Human Origins platform among four analyzed populations were used. We used the genetic variation of Mbuti as the outgroup. Red asterisk point meant the significant value (Absolute value of Z-scores larger than three or equal to three) observed in the symmetry-f_4 statistics and green circle point denoted the non-significant f_4-statistic values (Absolute value of Z-scores less than three). All ancient East Asians were listed along the Y-axis and f_4 values were labeled along the X-axis. All results were faceted or grouped via Tibetan populations. Significant negative f_4 values indicated that the third population shared more alleles with the second population, and also means Tibetan obtained additional gene flow from the third source population (or related populations). And the significant positive f_4 value indicated that the third population shared more derived alleles with the first population. The value of f_4-statistics equal to zero was marked as the blue dash line.

The bar indicated three standard errors.

Additional gene flow events when we assumed that Tibetans' direct ancestor is ancestral populations from Nepal

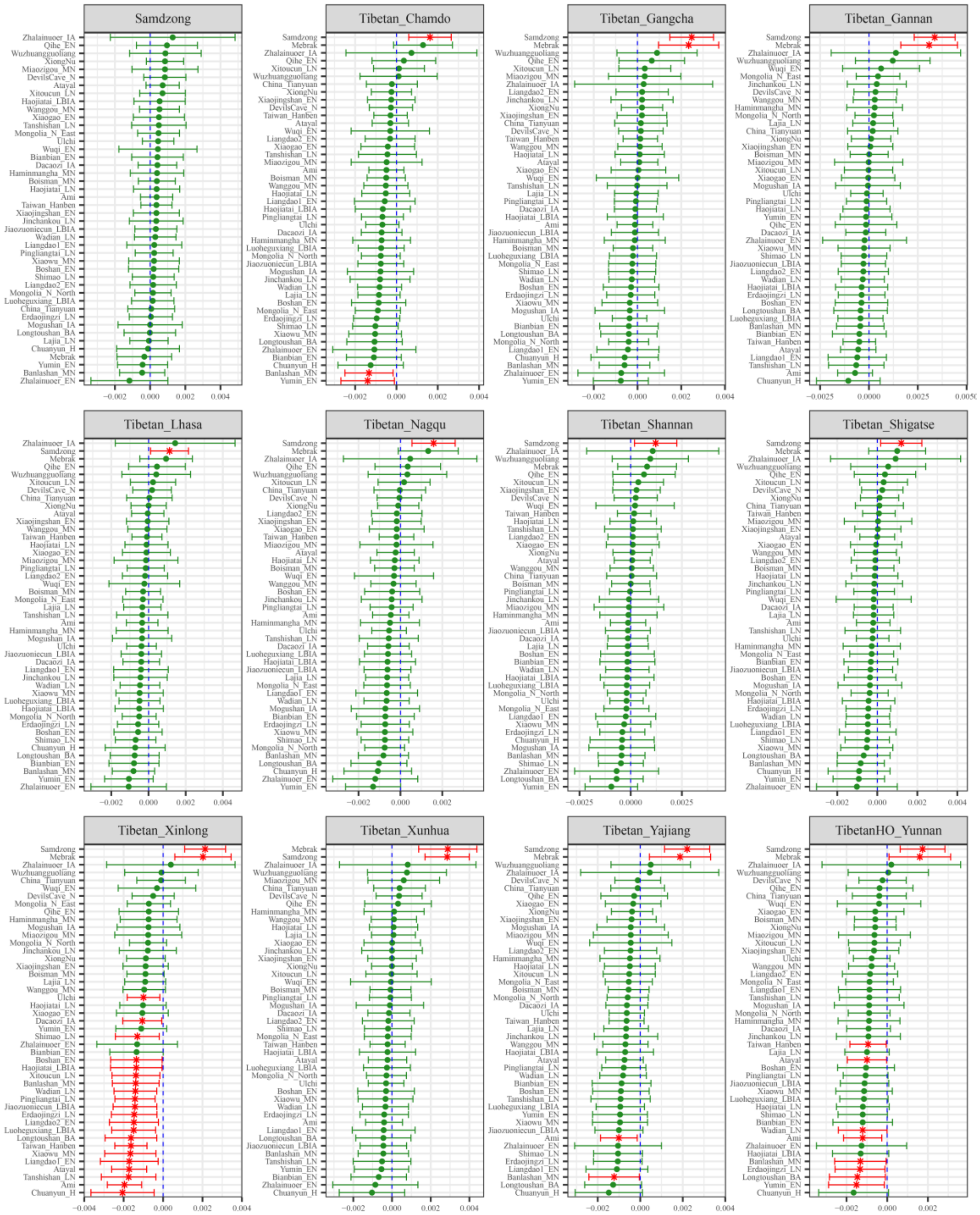

Figure S89. Results of affinity-f_4 statistics for the form f_4*(Chokhopani, Modern Tibetan; Neolithic to Historic East Asians, Mbuti)* showed the additional shared derived alleles from source populations except for ancestral populations from Nepal.

Here, overlapping SNP loci included in the Affymetrix Human Origins platform among four analyzed populations were used. We used the genetic variation of Mbuti as the outgroup. Red asterisk point meant the significant value (Absolute value of Z-scores larger than three or equal to three) observed in the symmetry-f_4 statistics and green circle point denoted the non-significant f_4-statistic values (Absolute value of Z-scores less than three). All ancient East Asians were listed along the Y-axis and f_4 values were labeled along the X-axis. All results were faceted or grouped via Tibetan populations. Significant negative f_4 values indicated that the third population shared more alleles with the second population, and also means Tibetan obtained additional gene flow from the third source population (or related

populations). And the significant positive f_4 value indicated that the third population shared more derived alleles with the first population. The value of f_4-statistics equal to zero was marked as the blue dash line. The bar indicated three standard errors.

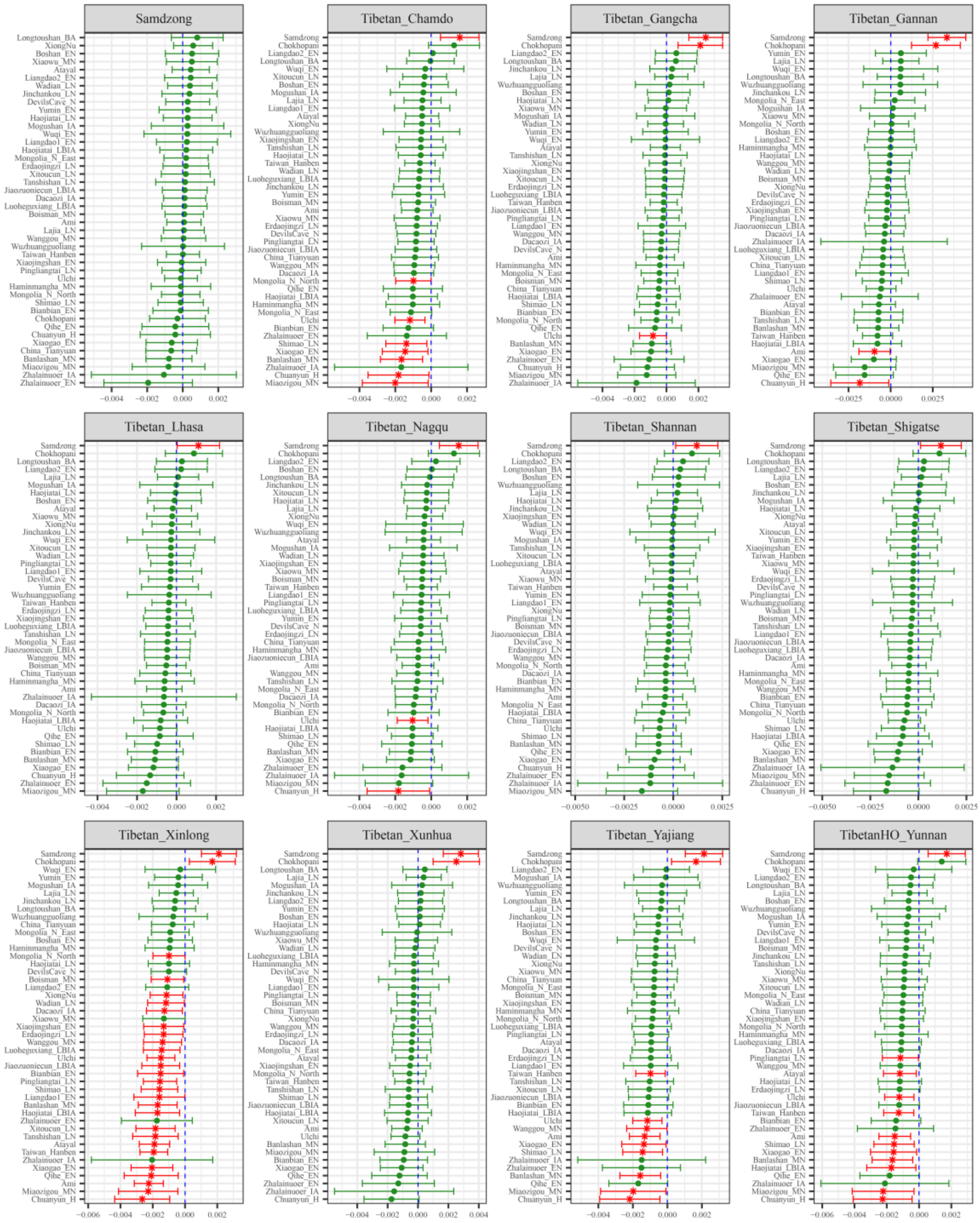

Figure S90. Results of affinity-f_4 statistics for the form f_4(*Mebrak, Modern Tibetan; Neolithic to Historic East Asians, Mbuti*) showed the additional shared derived alleles from source populations except for ancestral populations from Nepal.

Here, overlapping SNP loci included in the Affymetrix Human Origins platform among four analyzed populations were used. We used the genetic variation of Mbuti as the outgroup. Red asterisk point meant the significant value (Absolute value of Z-scores larger than three or equal to three) observed in the symmetry-f_4 statistics and green circle point denoted the non-significant f_4-statistic values (Absolute value of Z-scores less than three). All ancient East Asians were listed along the Y-axis and f_4 values were labeled along the X-axis. All results were faceted or grouped via Tibetan populations. Significant negative f_4 values indicated that the third population shared more alleles with the second population, and also means Tibetan obtained additional gene flow from the third source population (or related

populations). And the significant positive f_4 value indicated that the third population shared more derived alleles with the first population. The value of f_4-statistics equal to zero was marked as the blue dash line. The bar indicated three standard errors.

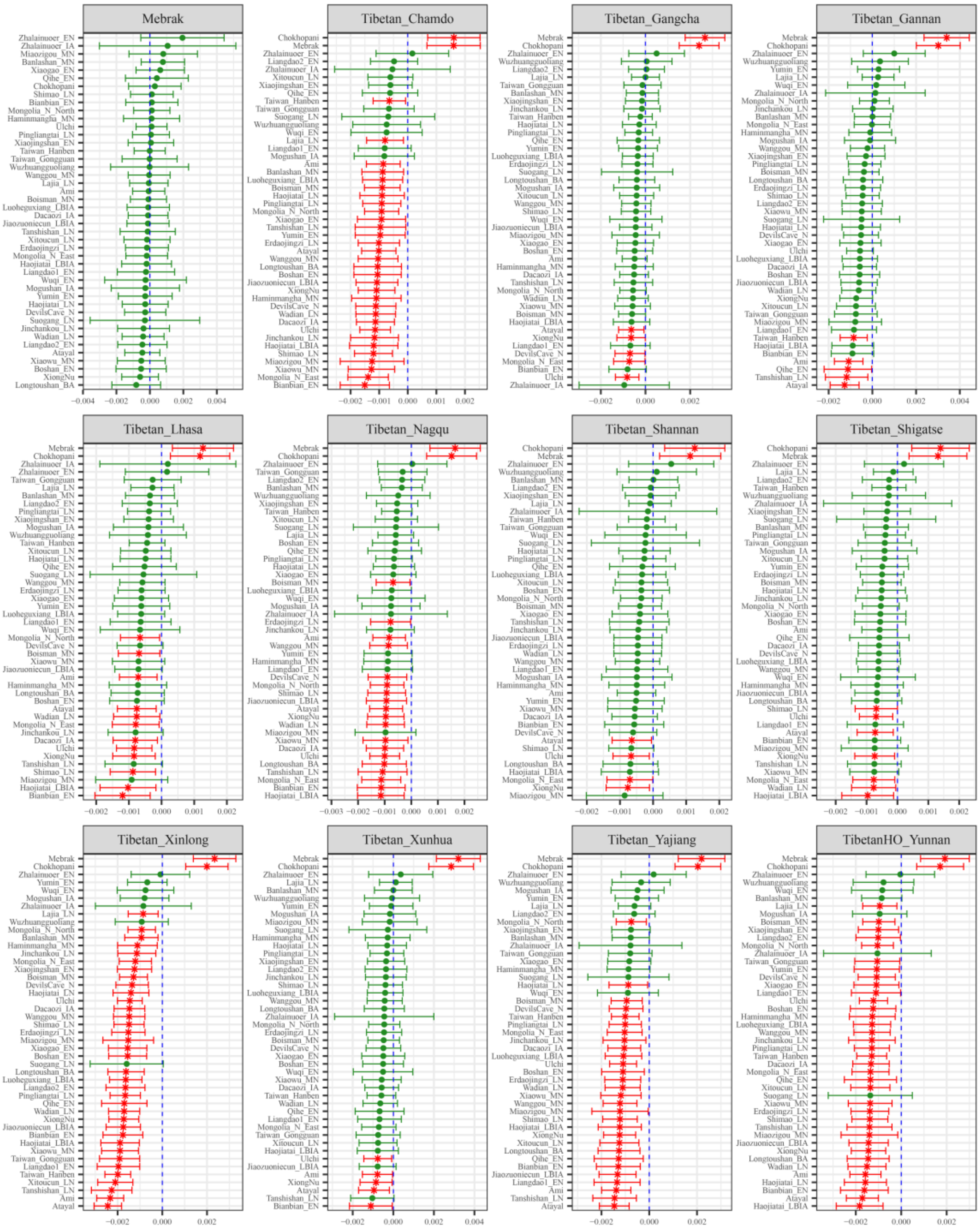

Figure S91. Results of affinity-f_4 statistics for the form f_4*(Samdzong, Modern Tibetan; Neolithic to Historic East Asians, Mbuti)* showed the additional shared derived alleles from source populations except for ancestral populations from Nepal.

Here, overlapping SNP loci included in the Affymetrix Human Origins platform among four analyzed populations were used. We used the genetic variation of Mbuti as the outgroup. Red asterisk point meant the significant value (Absolute value of Z-scores larger than three or equal to three) observed in the symmetry-f_4 statistics and green circle point denoted the non-significant f_4-statistic values (Absolute value of Z-scores less than three). All ancient East Asians were listed along the Y-axis and f_4 values were labeled along the X-axis. All results were faceted or grouped via Tibetan populations. Significant negative f_4 values indicated that the third population shared more alleles with the second population, and also means Tibetan obtained additional gene flow from the third source population (or related

populations). And the significant positive f_4 value indicated that the third population shared more derived alleles with the first population. The value of f_4-statistics equal to zero was marked as the blue dash line. The bar indicated three standard errors.

Additional gene flow events when we assumed that Tibetans' direct ancestor is ancestral populations from Mongolia Plateau or Baikal Lake Region

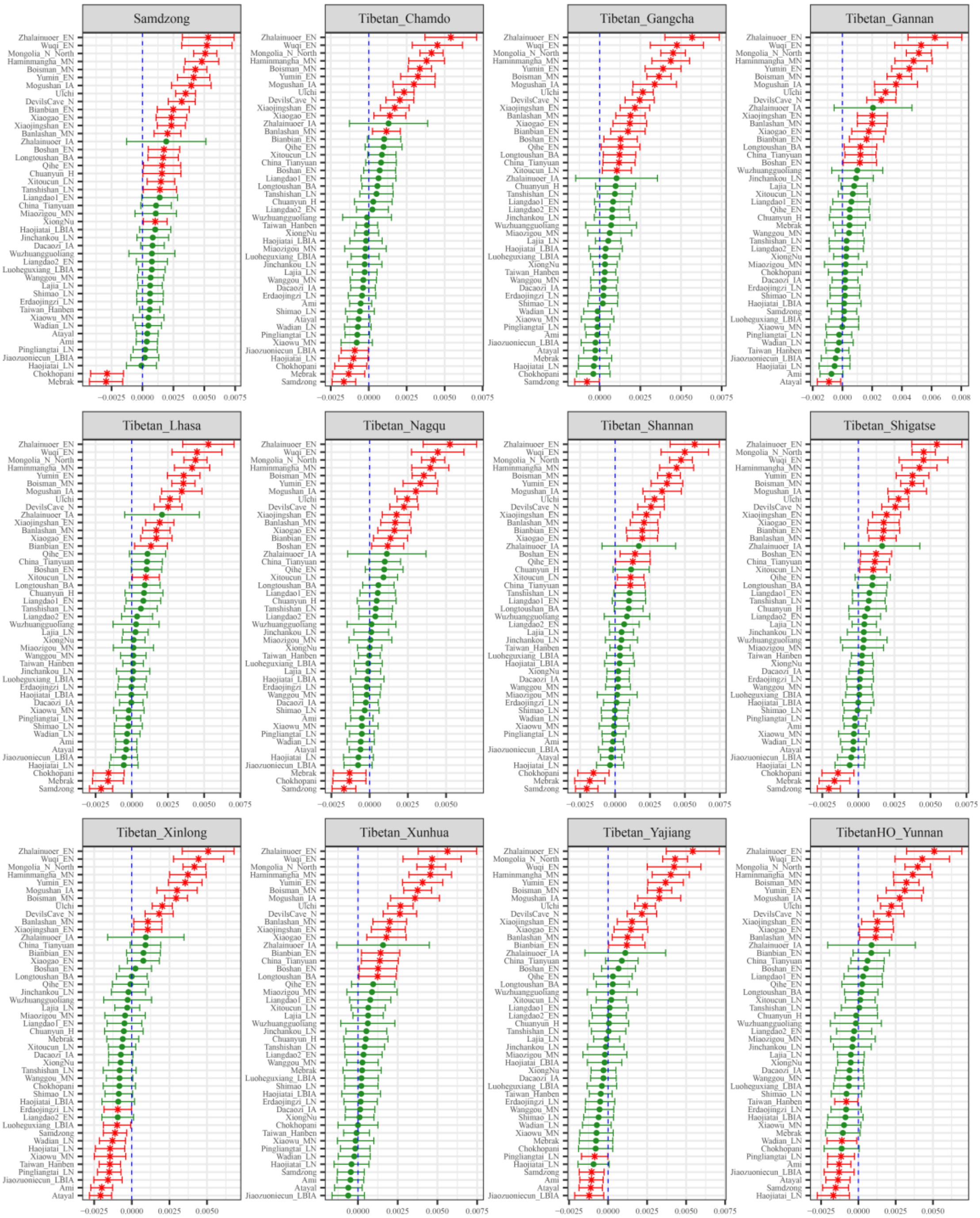

Figure S92. Results of affinity-f_4 statistics for the form *f_4(Mongolia_N_East, Modern Tibetan; Neolithic to Historic East Asians, Mbuti)* showed the additional shared derived alleles from source populations except for ancestral populations from Mongolia Plateau or Baikal Lake Region. Here, overlapping SNP loci included in the Affymetrix Human Origins platform among four analyzed populations were used. We used the genetic variation of Mbuti as the outgroup. Red asterisk point meant the significant value (Absolute value of Z-scores larger than three or equal to three) observed in the symmetry-f_4 statistics and green circle point denoted the non-significant f_4-statistic values (Absolute value of Z-scores less than three). All ancient East Asians were listed along the Y-axis and f_4 values were labeled along the X-axis. All results were faceted or grouped via Tibetan populations. Significant negative f_4 values indicated that the third population shared more alleles with the second population, and also means Tibetan obtained additional gene flow from the third source population (or related populations). And the significant positive f_4 value indicated that the third population shared more derived alleles with the first population. The value of f_4-statistics equal to zero was marked as the blue dash line.

The bar indicated three standard errors.

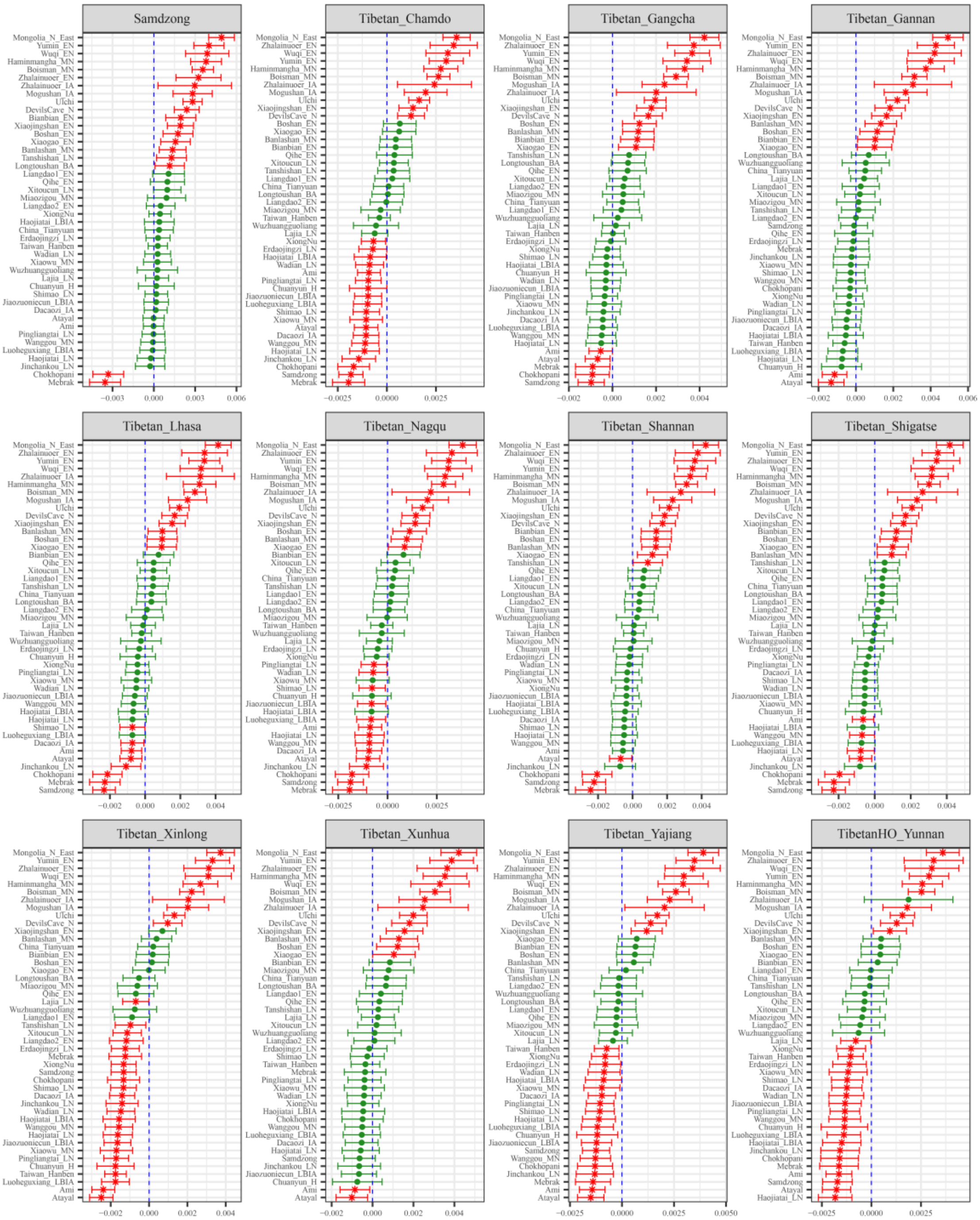

Figure S93. Results of affinity-f_4 statistics for the form f_4*(Mongolia_N_North, Modern Tibetan; Neolithic to Historic East Asians, Mbuti)* showed the additional shared derived alleles from source populations except for ancestral populations from Mongolia Plateau or Baikal Lake Region. Here, overlapping SNP loci included in the Affymetrix Human Origins platform among four analyzed populations were used. We used the genetic variation of Mbuti as the outgroup. Red asterisk point meant the significant value (Absolute value of Z-scores larger than three or equal to three) observed in the symmetry-f_4 statistics and green circle point denoted the non-significant f_4-statistic values (Absolute value of Z-scores less than three). All ancient East Asians were listed along the Y-axis and f_4 values were labeled along the X-axis. All results were faceted or grouped via Tibetan populations. Significant negative f_4 values indicated that the third population shared more alleles with the second population, and also means Tibetan obtained additional gene flow from the third source population (or related populations). And the significant positive f_4 value indicated that the third population shared more derived alleles with the first population. The value of f_4-statistics equal to zero was marked as the blue dash line.

The bar indicated three standard errors.

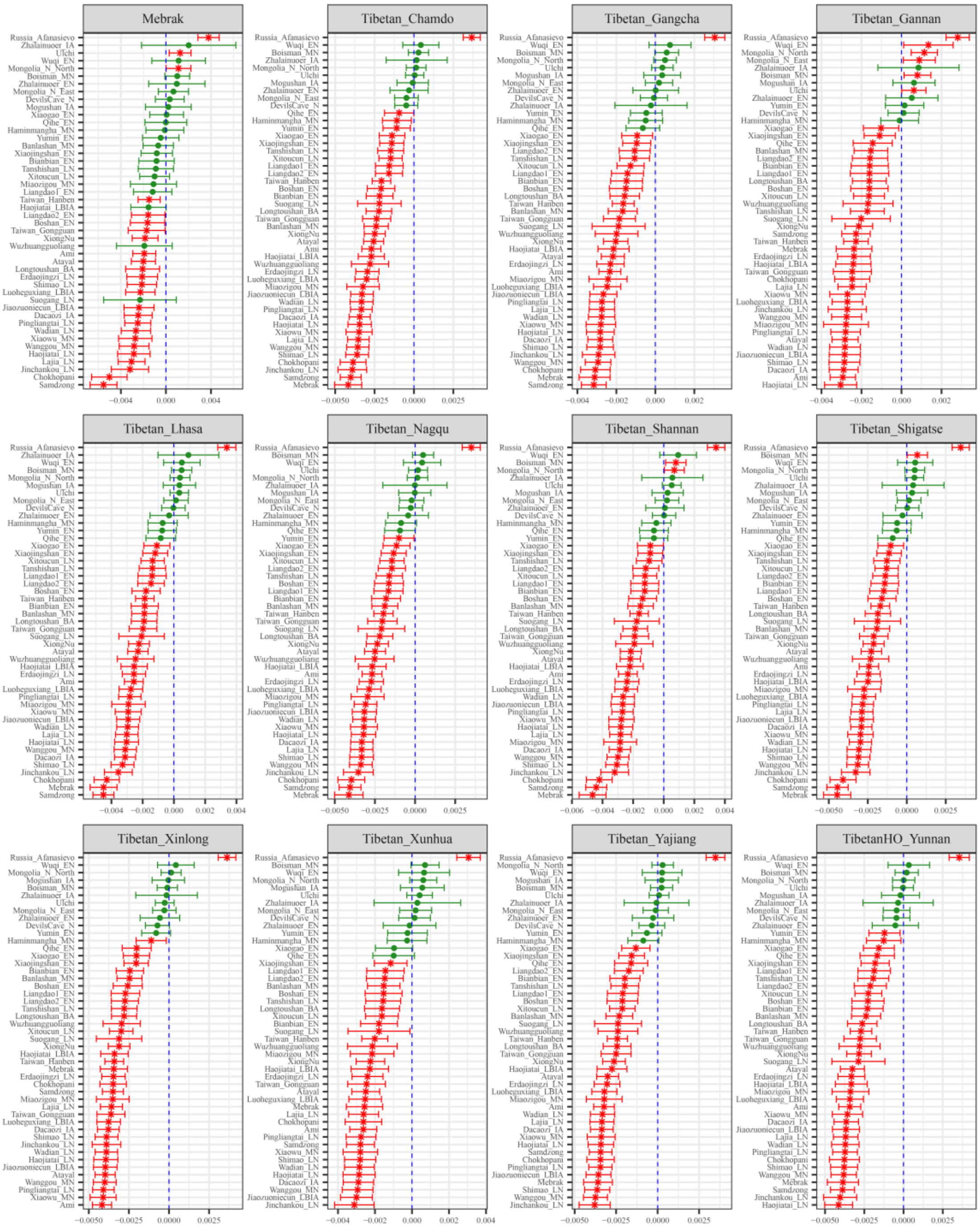

Figure S94. Results of affinity-f_4 statistics for the form f_4*(Russia_OldBeringSea_Ekven, Modern Tibetan; Neolithic to Historic East Asians, Mbuti)* showed the additional shared derived alleles from source populations except for ancestral populations from Mongolia Plateau or Baikal Lake Region. Here, overlapping SNP loci included in the Affymetrix Human Origins platform among four analyzed populations were used. We used the genetic variation of Mbuti as the outgroup. Red asterisk point meant the significant value (Absolute value of Z-scores larger than three or equal to three) observed in the symmetry-f_4 statistics and green circle point denoted the non-significant f_4-statistic values (Absolute value of Z-scores less than three). All ancient East Asians were listed along the Y-axis and f_4 values were labeled along the X-axis. All results were faceted or grouped via Tibetan populations. Significant negative f_4 values indicated that the third population shared more alleles with the second population, and also means Tibetan obtained additional gene flow from the third source population (or related populations). And the significant positive f_4 value indicated that the third population shared more derived alleles with the first population. The value of f_4-statistics equal to zero was marked as the blue dash line.

The bar indicated three standard errors.

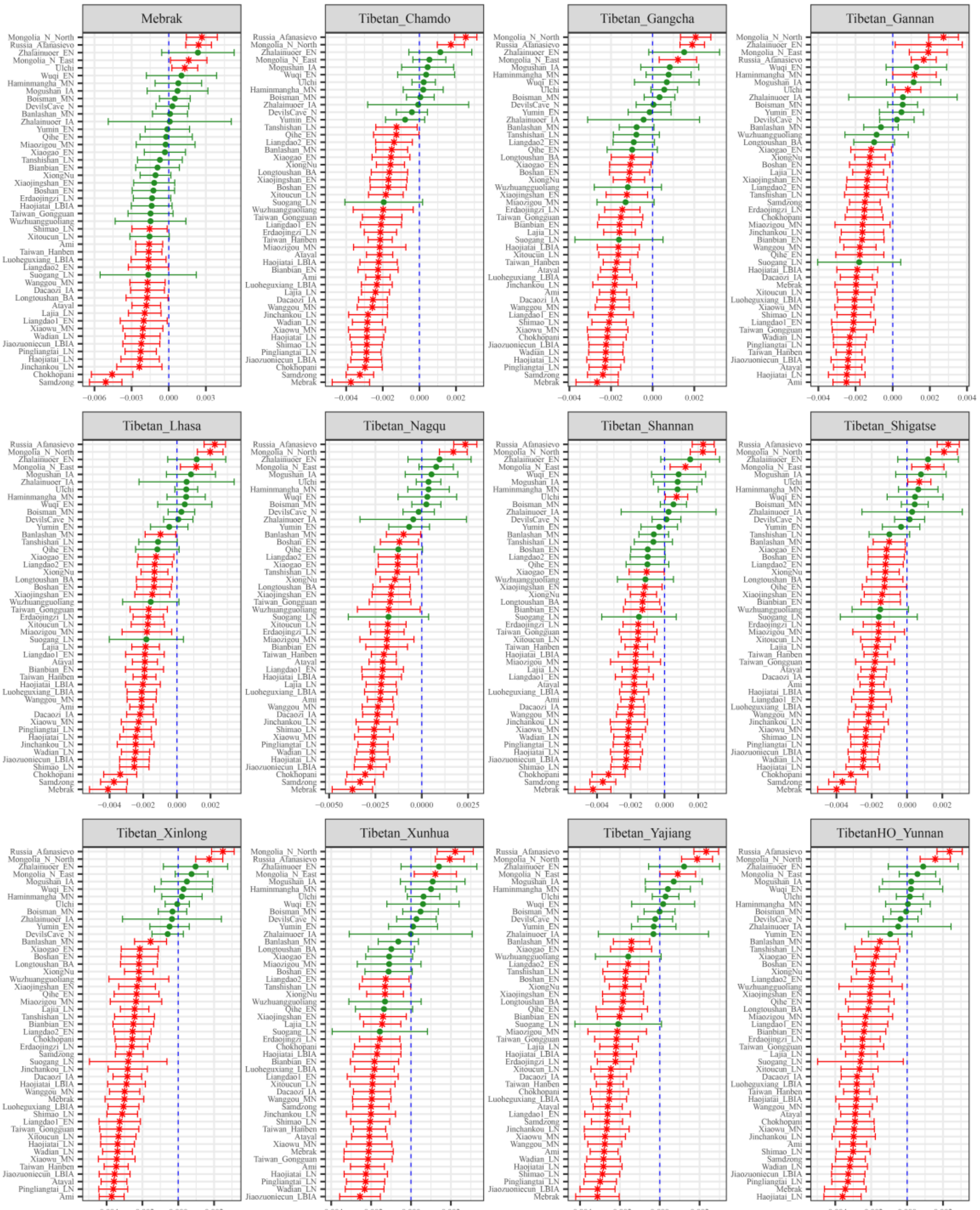

Figure S95. Results of affinity-f_4 statistics for the form f_4*(Russia_Shamanka_EBA, Modern Tibetan; Neolithic to Historic East Asians, Mbuti)* showed the additional shared derived alleles from source populations except for ancestral populations from Mongolia Plateau or Baikal Lake Region. Here, overlapping SNP loci included in the Affymetrix Human Origins platform among four analyzed populations were used. We used the genetic variation of Mbuti as the outgroup. Red asterisk point meant the significant value (Absolute value of Z-scores larger than three or equal to three) observed in the symmetry-f_4 statistics and green circle point denoted the non-significant f_4-statistic values (Absolute value of Z-scores less than three). All ancient East Asians were listed along the Y-axis and f_4 values were labeled along the X-axis. All results were faceted or grouped via Tibetan populations. Significant negative f_4 values indicated that the third population shared more alleles with the second population, and also means Tibetan obtained additional gene flow from the third source population (or related populations). And the significant positive f_4 value indicated that the third population shared more derived alleles with the first population. The value of f_4-statistics equal to zero was marked as the blue dash line. The bar indicated three standard errors.

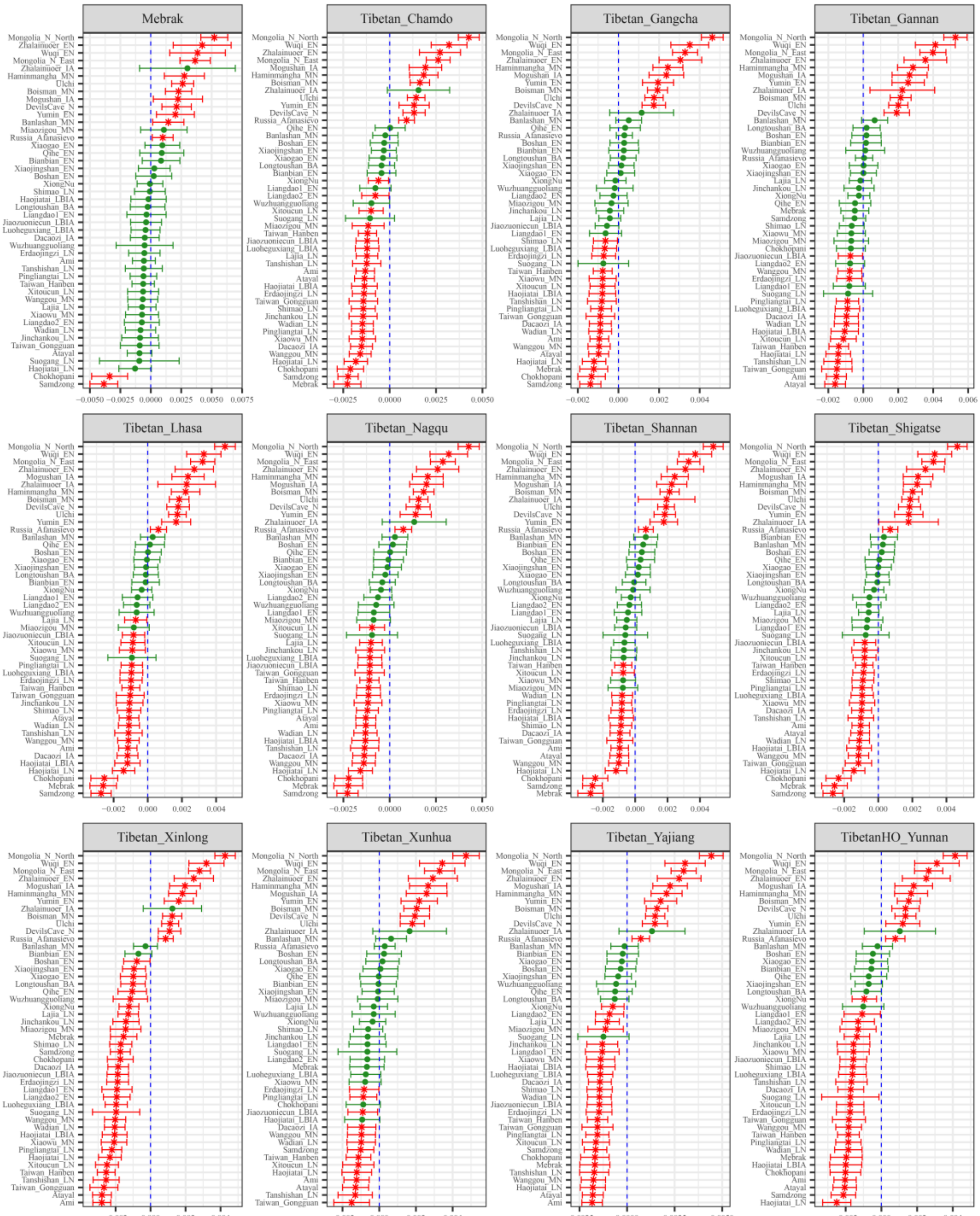

Figure S96. Results of affinity-f_4 statistics for the form f_4*(Russia_Shamanka_Eneolithic, Modern Tibetan; Neolithic to Historic East Asians, Mbuti)* showed the additional shared derived alleles from source populations except for ancestral populations from Mongolia Plateau or Baikal Lake Region. Here, overlapping SNP loci included in the Affymetrix Human Origins platform among four analyzed populations were used. We used the genetic variation of Mbuti as the outgroup. Red asterisk point meant the significant value (Absolute value of Z-scores larger than three or equal to three) observed in the symmetry-f_4 statistics and green circle point denoted the non-significant f_4-statistic values (Absolute value of Z-scores less than three). All ancient East Asians were listed along the Y-axis and f_4 values were labeled along the X-axis. All results were faceted or grouped via Tibetan populations. Significant negative f_4 values indicated that the third population shared more alleles with the second population, and also means Tibetan obtained additional gene flow from the third source population (or related populations). And the significant positive f_4 value indicated that the third population shared more derived alleles with the first population. The value of f_4-statistics equal to zero was marked as the blue dash line. The bar indicated three standard errors.

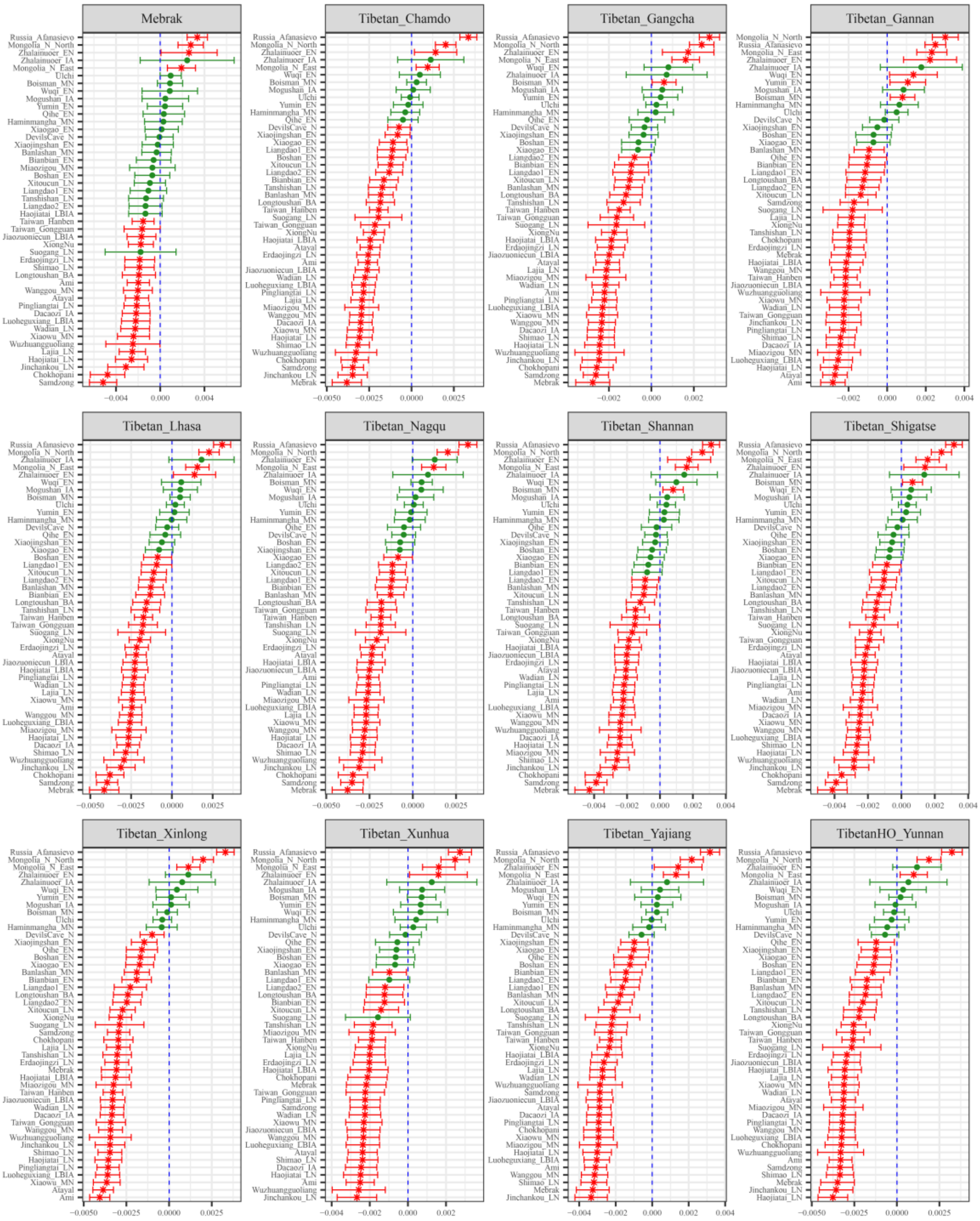

Figure S97. Results of affinity-f_4 statistics for the form *f_4(Russia_UstBelaya_Angara, Modern Tibetan; Neolithic to Historic East Asians, Mbuti)* showed the additional shared derived alleles from source populations except for ancestral populations from Mongolia Plateau or Baikal Lake Region. Here, overlapping SNP loci included in the Affymetrix Human Origins platform among four analyzed populations were used. We used the genetic variation of Mbuti as the outgroup. Red asterisk point meant the significant value (Absolute value of Z-scores larger than three or equal to three) observed in the symmetry-f_4 statistics and green circle point denoted the non-significant f_4-statistic values (Absolute value of Z-scores less than three). All ancient East Asians were listed along the Y-axis and f_4 values were labeled along the X-axis. All results were faceted or grouped via Tibetan populations. Significant negative f_4 values indicated that the third population shared more alleles with the second population, and also means Tibetan obtained additional gene flow from the third source population (or related populations). And the significant positive f_4 value indicated that the third population shared more derived alleles with the first population. The value of f_4-statistics equal to zero was marked as the blue dash line. The bar indicated three standard errors.

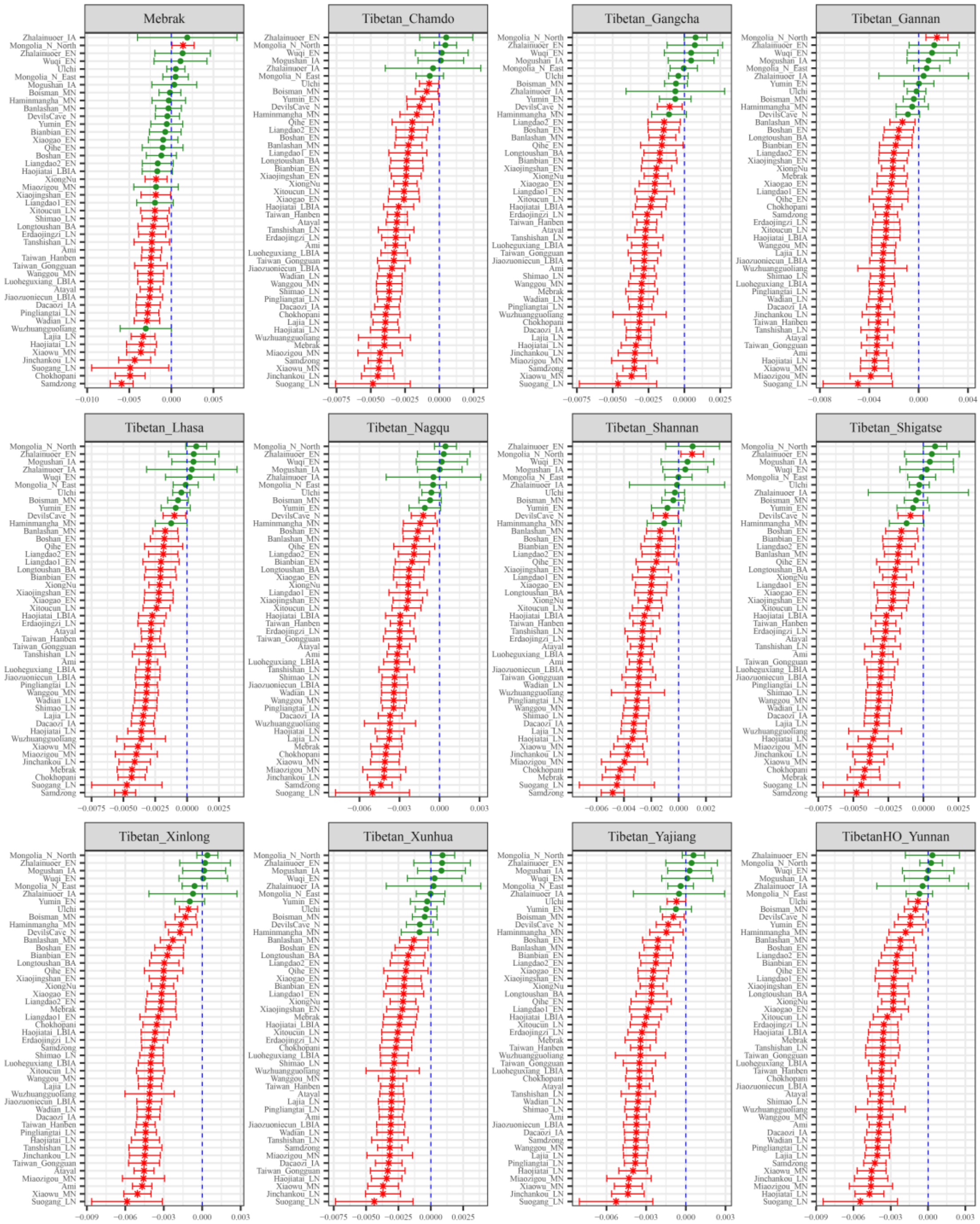

Figure S98. Results of affinity-f_4 statistics for the form f_4*(Russia_UstIda_EBA, Modern Tibetan; Neolithic to Historic East Asians, Mbuti)* showed the additional shared derived alleles from source populations except for ancestral populations from Mongolia Plateau or Baikal Lake Region.
Here, overlapping SNP loci included in the Affymetrix Human Origins platform among four analyzed populations were used. We used the genetic variation of Mbuti as the outgroup. Red asterisk point meant the significant value (Absolute value of Z-scores larger than three or equal to three) observed in the symmetry-f_4 statistics and green circle point denoted the non-significant f_4-statistic values (Absolute value of Z-scores less than three). All ancient East Asians were listed along the Y-axis and f_4 values were labeled along the X-axis. All results were faceted or grouped via Tibetan populations. Significant negative f_4 values indicated that the third population shared more alleles with the second population, and also means Tibetan obtained additional gene flow from the third source population (or related populations). And the significant positive f_4 value indicated that the third population shared more derived alleles with the first population. The value of f_4-statistics equal to zero was marked as the blue dash line.

The bar indicated three standard errors.

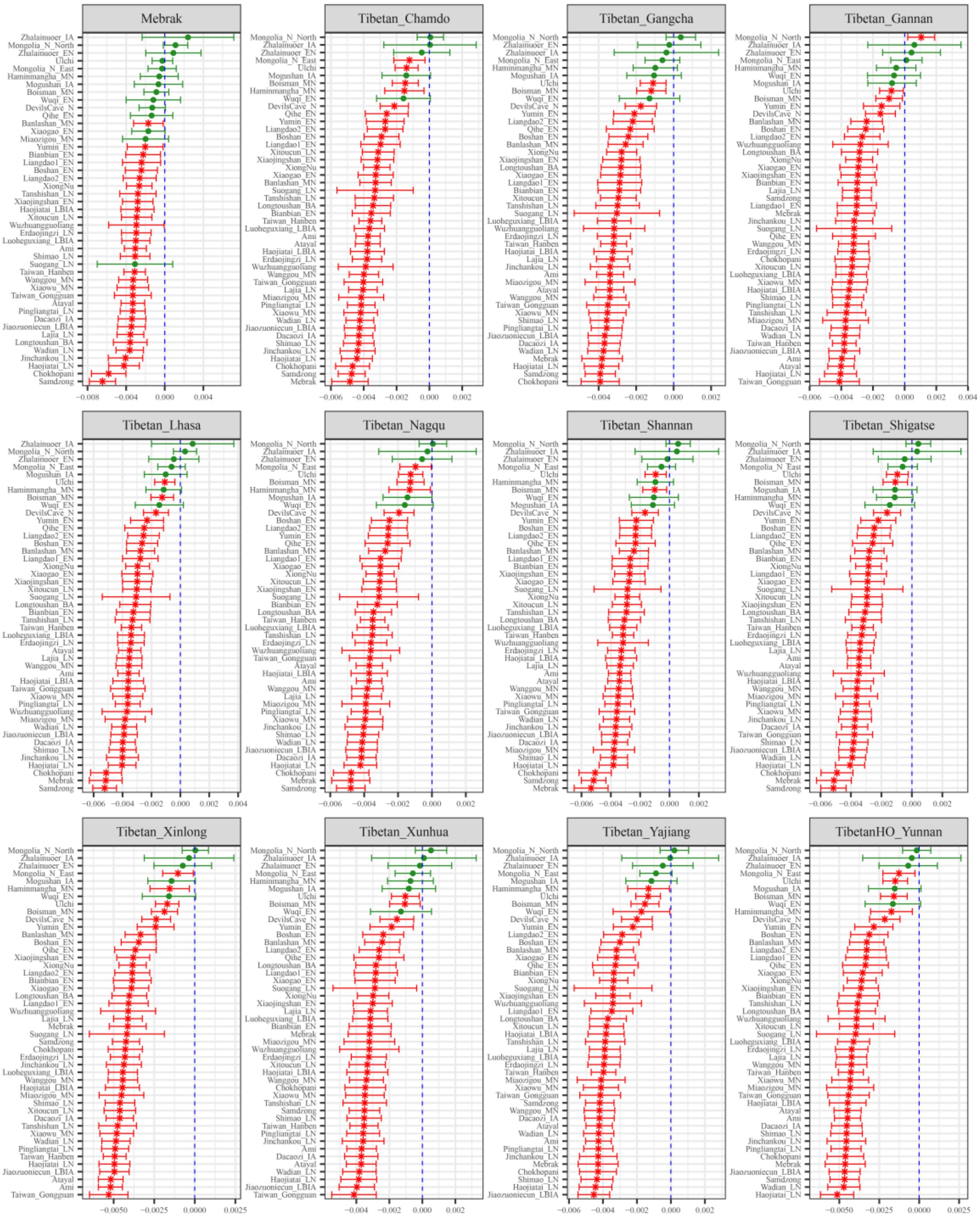

Figure S99. Results of affinity-f_4 statistics for the form f_4*(Russia_UstIda_LN, Modern Tibetan; Neolithic to Historic East Asians, Mbuti)* showed the additional shared derived alleles from source populations except for ancestral populations from Mongolia Plateau or Baikal Lake Region.
Here, overlapping SNP loci included in the Affymetrix Human Origins platform among four analyzed populations were used. We used the genetic variation of Mbuti as the outgroup. Red asterisk point meant the significant value (Absolute value of Z-scores larger than three or equal to three) observed in the symmetry-f_4 statistics and green circle point denoted the non-significant f_4-statistic values (Absolute value of Z-scores less than three). All ancient East Asians were listed along the Y-axis and f_4 values were labeled along the X-axis. All results were faceted or grouped via Tibetan populations. Significant negative f_4 values indicated that the third population shared more alleles with the second population, and also means Tibetan obtained additional gene flow from the third source population (or related populations). And the significant positive f_4 value indicated that the third population shared more derived alleles with the first population. The value of f_4-statistics equal to zero was marked as the blue dash line.

The bar indicated three standard errors.

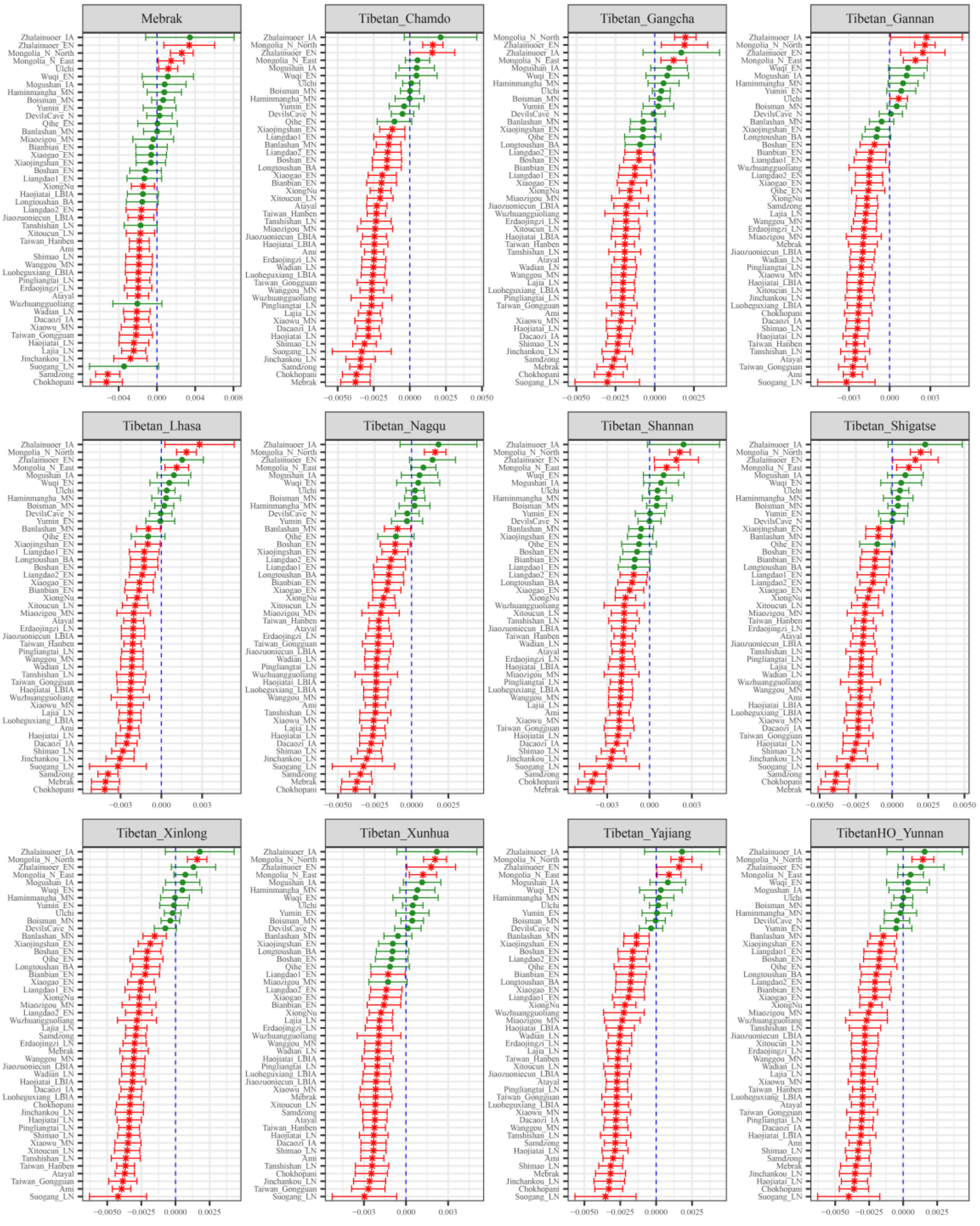

Figure S100. Results of affinity-f_4 statistics for the form f_4*(UstBelaya_EBA, Modern Tibetan; Neolithic to Historic East Asians, Mbuti)* showed the additional shared derived alleles from source populations except for ancestral populations from Mongolia Plateau or Baikal Lake Region. Here, overlapping SNP loci included in the Affymetrix Human Origins platform among four analyzed populations were used. We used the genetic variation of Mbuti as the outgroup. Red asterisk point meant the significant value (Absolute value of Z-scores larger than three or equal to three) observed in the symmetry-f_4 statistics and green circle point denoted the non-significant f_4-statistic values (Absolute value of Z-scores less than three). All ancient East Asians were listed along the Y-axis and f_4 values were labeled along the X-axis. All results were faceted or grouped via Tibetan populations. Significant negative f_4 values indicated that the third population shared more alleles with the second population, and also means Tibetan obtained additional gene flow from the third source population (or related populations). And the significant positive f_4 value indicated that the third population shared more derived alleles with the first population. The value of f_4-statistics equal to zero was marked as the blue dash line.

The bar indicated three standard errors.

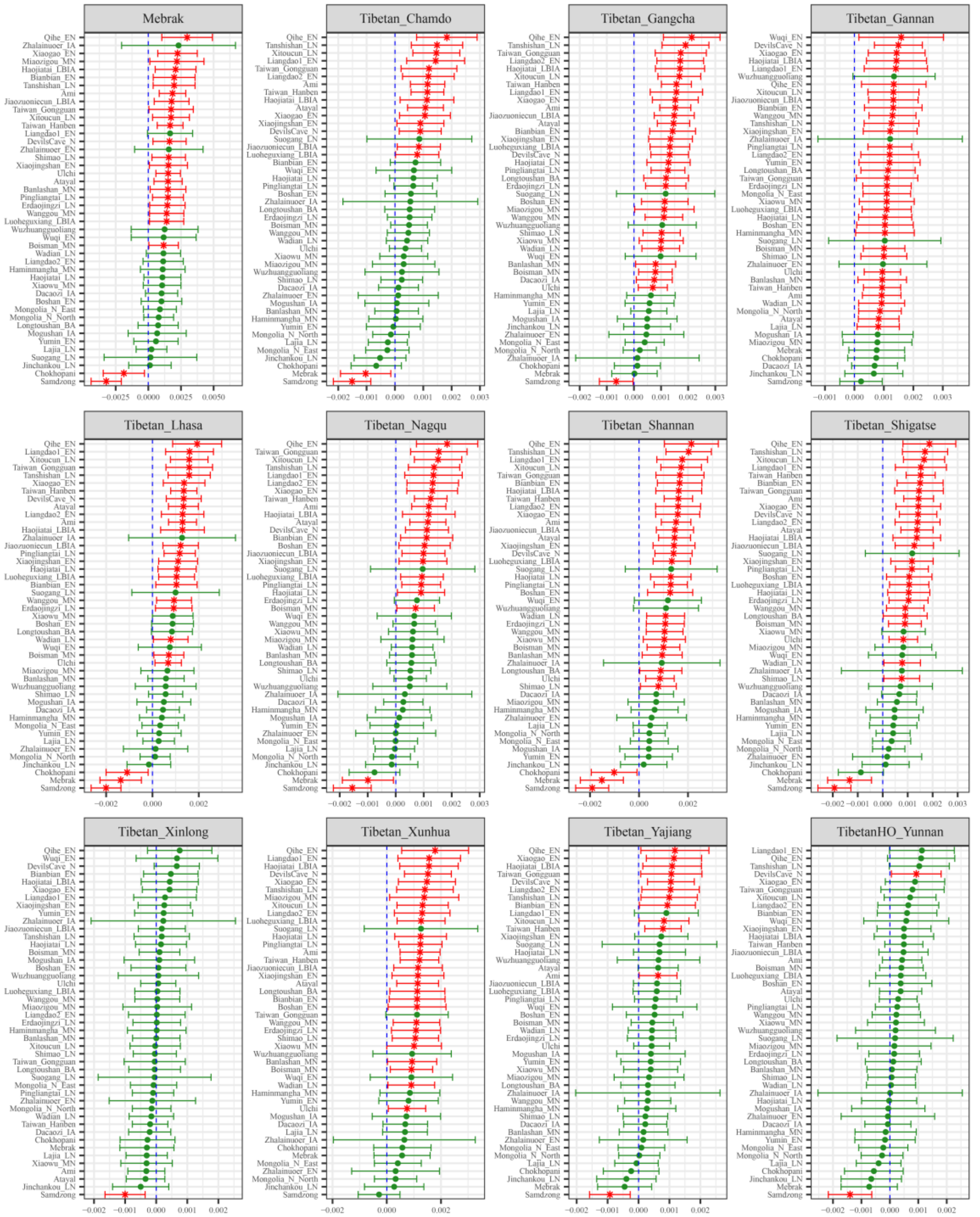

Figure S101. Results of affinity-f_4 statistics for the form f_4*(XiongNu, Modern Tibetan; Neolithic to Historic East Asians, Mbuti)* showed the additional shared derived alleles from source populations except for ancestral populations from Mongolia Plateau or Baikal Lake Region.

Here, overlapping SNP loci included in the Affymetrix Human Origins platform among four analyzed populations were used. We used the genetic variation of Mbuti as the outgroup. Red asterisk point meant the significant value (Absolute value of Z-scores larger than three or equal to three) observed in the symmetry-f_4 statistics and green circle point denoted the non-significant f_4-statistic values (Absolute value of Z-scores less than three). All ancient East Asians were listed along the Y-axis and f_4 values were labeled along the X-axis. All results were faceted or grouped via Tibetan populations. Significant negative f_4 values indicated that the third population shared more alleles with the second population, and also means Tibetan obtained additional gene flow from the third source population (or related populations). And the significant positive f_4 value indicated that the third population shared more derived alleles with the first population. The value of f_4-statistics equal to zero was marked as the blue dash line.

The bar indicated three standard errors.

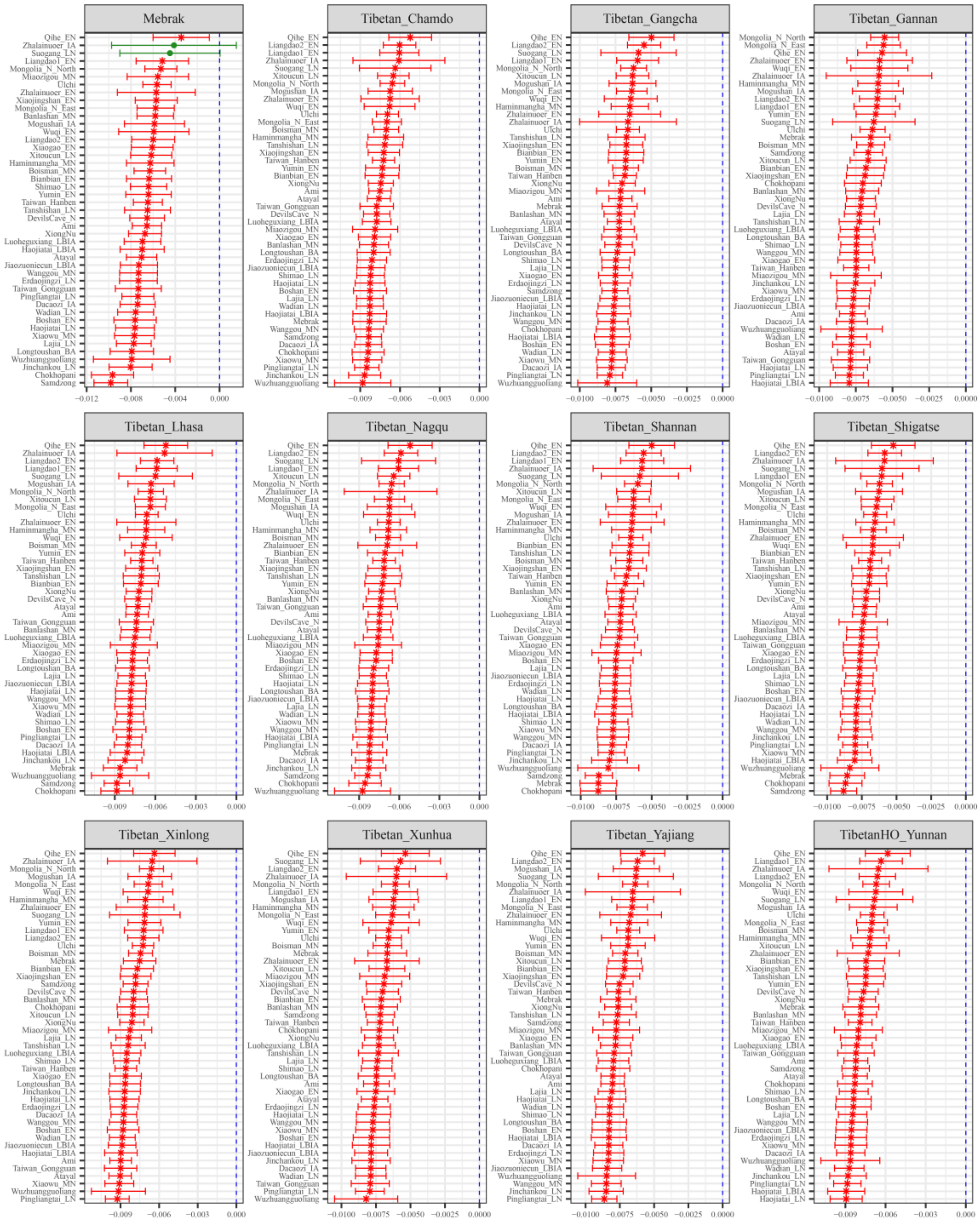

Figure S102. Results of affinity-*f*$_4$ statistics for the form *f*$_4$*(Shirenzigou_IA, Modern Tibetan; Neolithic to Historic East Asians, Mbuti)* showed the additional shared derived alleles from source populations except for ancestral populations from Xinjiang or their related ancestral populations. Here, overlapping SNP loci included in the Affymetrix Human Origins platform among four analyzed populations were used. We used the genetic variation of Mbuti as the outgroup. Red asterisk point meant the significant value (Absolute value of Z-scores larger than three or equal to three) observed in the symmetry-f_4 statistics and green circle point denoted the non-significant f_4-statistic values (Absolute value of Z-scores less than three). All ancient East Asians were listed along the Y-axis and f_4 values were labeled along the X-axis. All results were faceted or grouped via Tibetan populations. Significant negative f_4 values indicated that the third population shared more alleles with the second population, and also means Tibetan obtained additional gene flow from the third source population (or related populations). And the significant positive f_4 value indicated that the third population shared more derived alleles with the first population. The value of f_4-statistics equal to zero was marked as the blue dash line.

The bar indicated three standard errors.

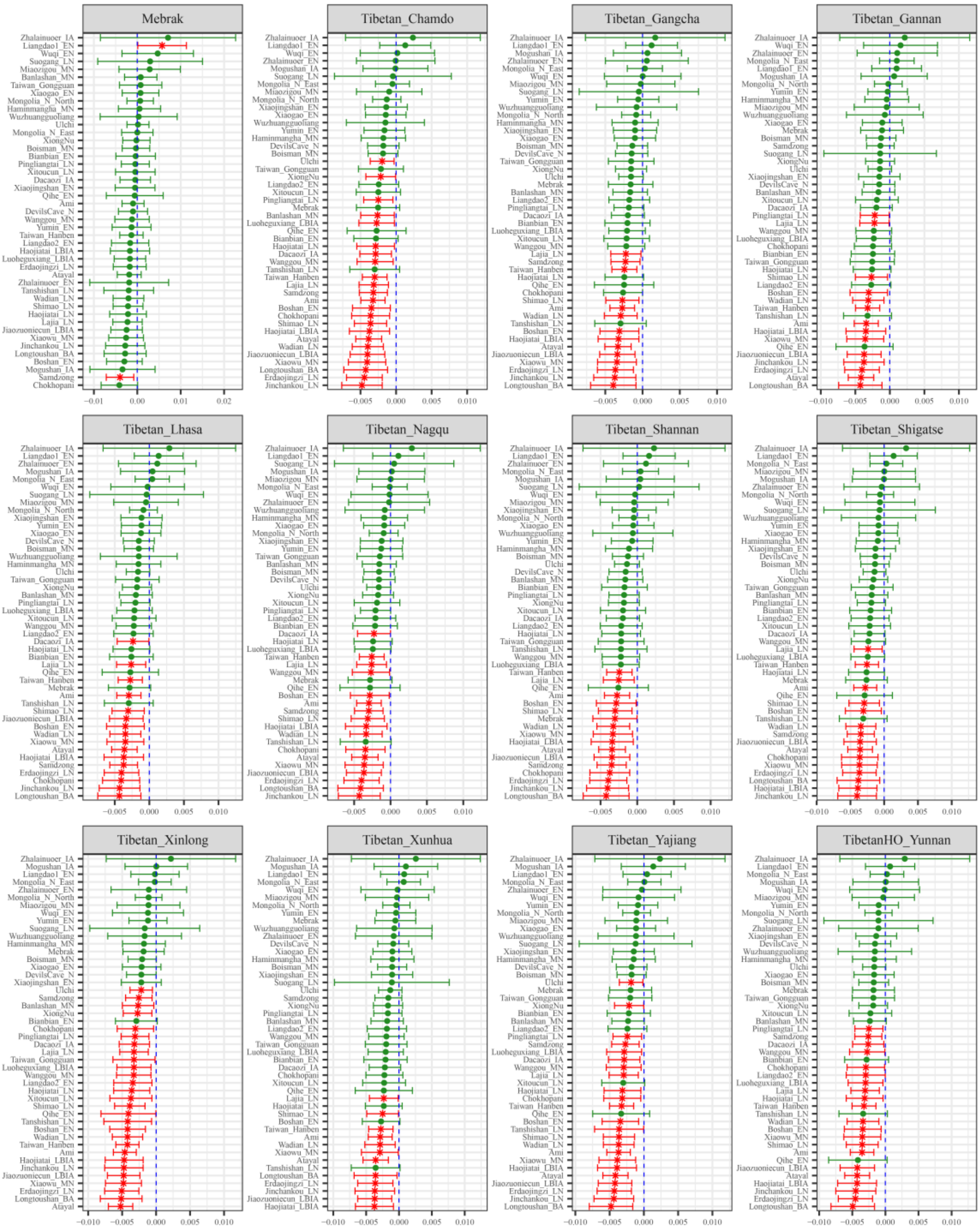

Figure S103. Results of affinity-f_4 statistics for the form f_4*(Shirenzigou_IA_E, Modern Tibetan; Neolithic to Historic East Asians, Mbuti)* showed the additional shared derived alleles from source populations except for ancestral populations from Xinjiang or their related ancestral populations. Here, overlapping SNP loci included in the Affymetrix Human Origins platform among four analyzed populations were used. We used the genetic variation of Mbuti as the outgroup. Red asterisk point meant the significant value (Absolute value of Z-scores larger than three or equal to three) observed in the symmetry-f_4 statistics and green circle point denoted the non-significant f_4-statistic values (Absolute value of Z-scores less than three). All ancient East Asians were listed along the Y-axis and f_4 values were labeled along the X-axis. All results were faceted or grouped via Tibetan populations. Significant negative f_4 values indicated that the third population shared more alleles with the second population, and also means Tibetan obtained additional gene flow from the third source population (or related populations). And the significant positive f_4 value indicated that the third population shared more derived

alleles with the first population. The value of f_4-statistics equal to zero was marked as the blue dash line. The bar indicated three standard errors.

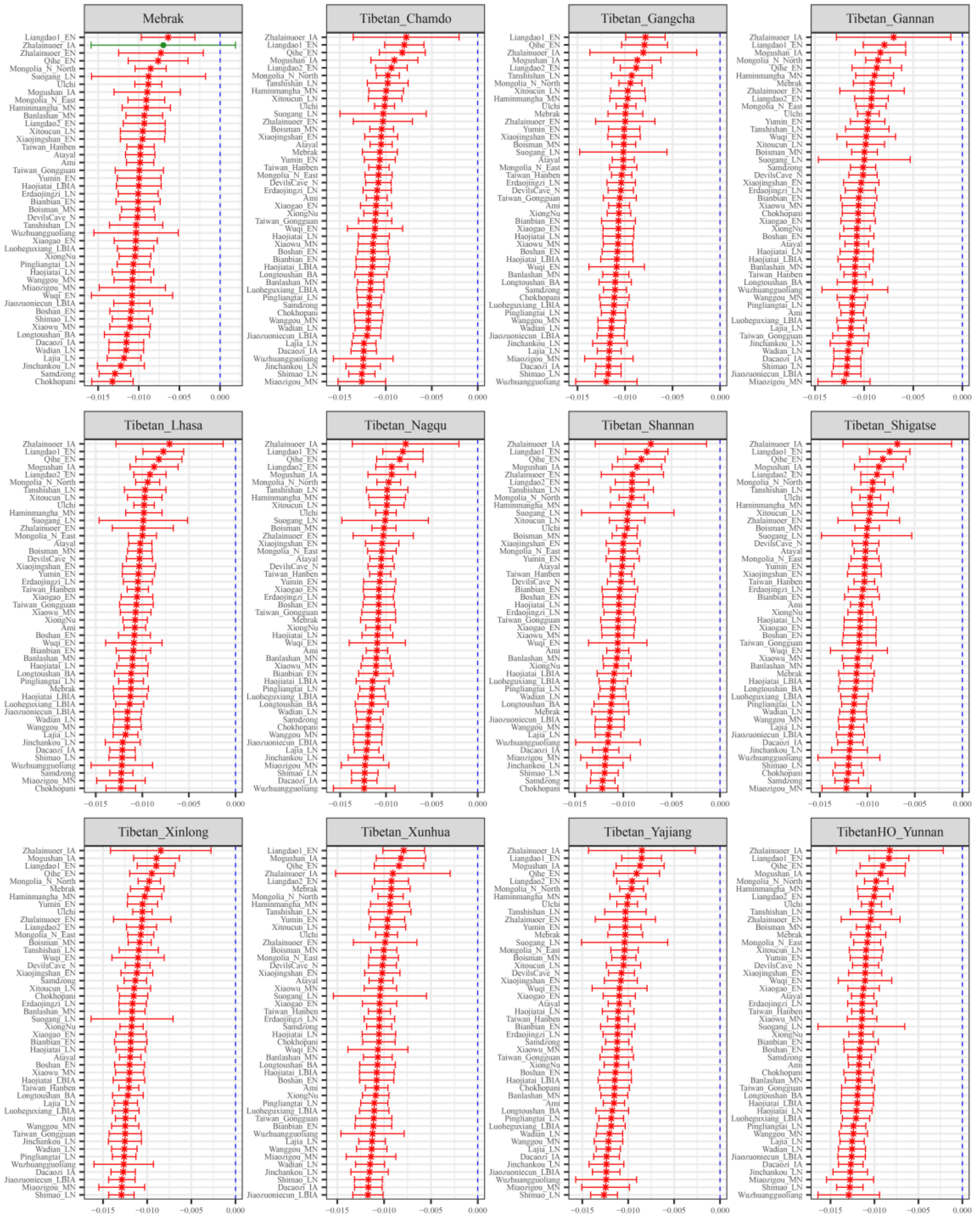

Figure S104. Results of affinity-f_4 statistics for the form *f_4(Shirenzigou_IA_W, Modern Tibetan; Neolithic to Historic East Asians, Mbuti)* showed the additional shared derived alleles from source populations except for ancestral populations from Xinjiang or their related ancestral populations. Here, overlapping SNP loci included in the Affymetrix Human Origins platform among four analyzed populations were used. We used the genetic variation of Mbuti as the outgroup. Red asterisk point meant the significant value (Absolute value of Z-scores larger than three or equal to three) observed in the symmetry-f_4 statistics and green circle point denoted the non-significant f_4-statistic values (Absolute value of Z-scores less than three). All ancient East Asians were listed along the Y-axis and f_4 values were labeled along the X-axis. All results were faceted or grouped via Tibetan populations. Significant negative f_4 values indicated that the third population shared more alleles with the second population, and also means Tibetan obtained additional gene flow from the third source population (or related populations). And the significant positive f_4 value indicated that the third population shared more derived

alleles with the first population. The value of f_4-statistics equal to zero was marked as the blue dash line. The bar indicated three standard errors.

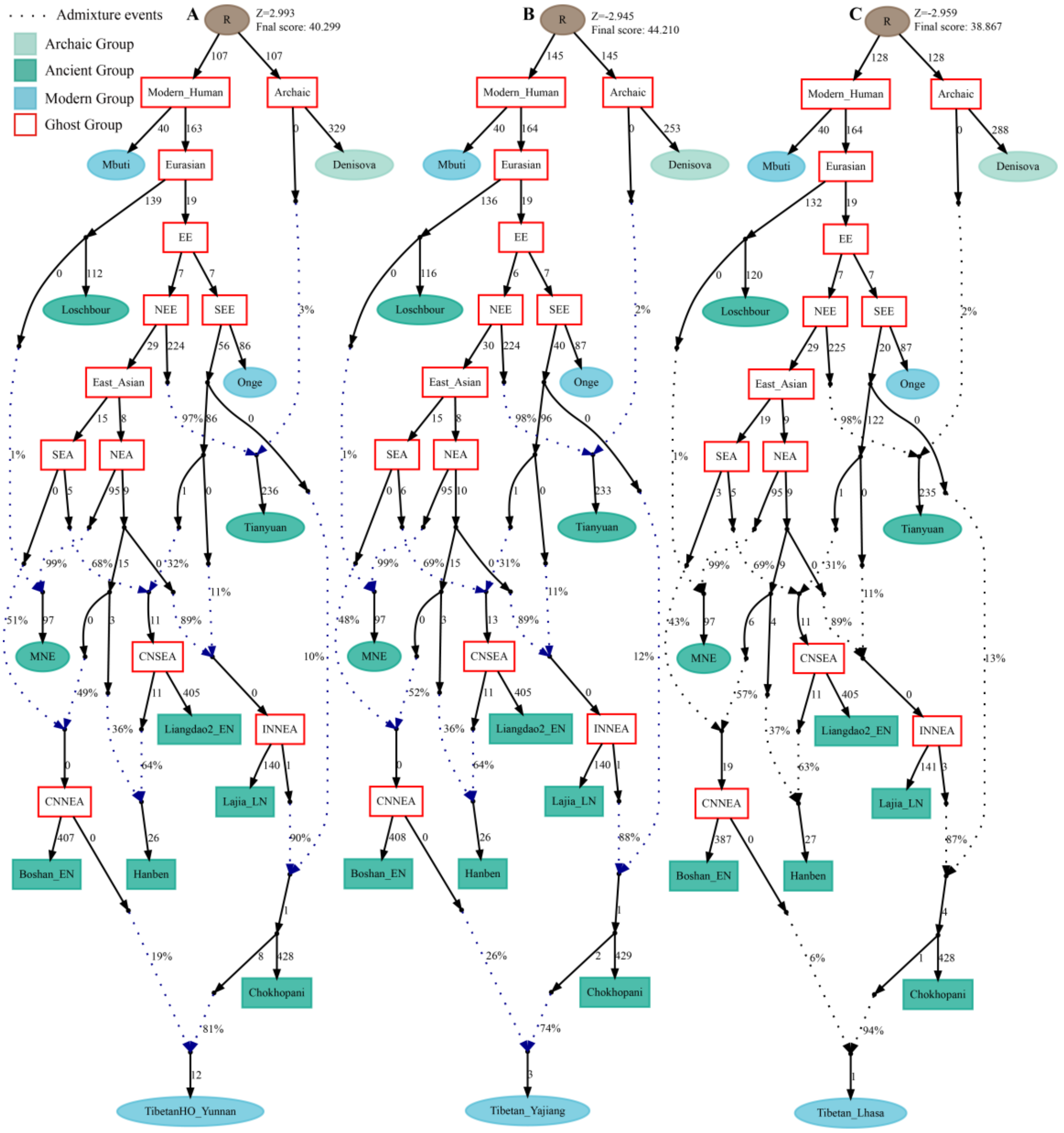

Figure S105. Admixture graph model of modern highland and lowland Tibetans based on the Human Origin dataset using early Neolithic Boshan people as the source of the second migration into Tibet Plateau.

Admixture history of lowland Tibetan from Yunnan (**A**), Tibetan from Yajiang (**B**) and highland Tibetan from Lhasa (**C**). Western Eurasian was represented by Loschbour. Deep southern Eurasian (SEE) and northern Eurasian (NEE) were represented by South Asian Hunter-Gatherer of Onge and 40,000-year-old Tianyuan people. East Asian was subsequently diverged as northern East Asian (NEA) and southern East Asian (SEA). Coastal Neolithic southern East Asian (CNSEA), coastal Neolithic Northern East Asian (CNNEA) and inland Neolithic northern East Asian (INNEA) were represented by Liangdao2_EN, Boshan_EN and Lajia_LN, respectively. All f_4-statistics of included populations are predicted to within 2.959 standard errors of their observed values. Branch lengths are given in units of 1000 times the f_2 drift distance (rounded to the nearest integer). Blue dotted lines denoted admixture events with admixture proportions as shown.

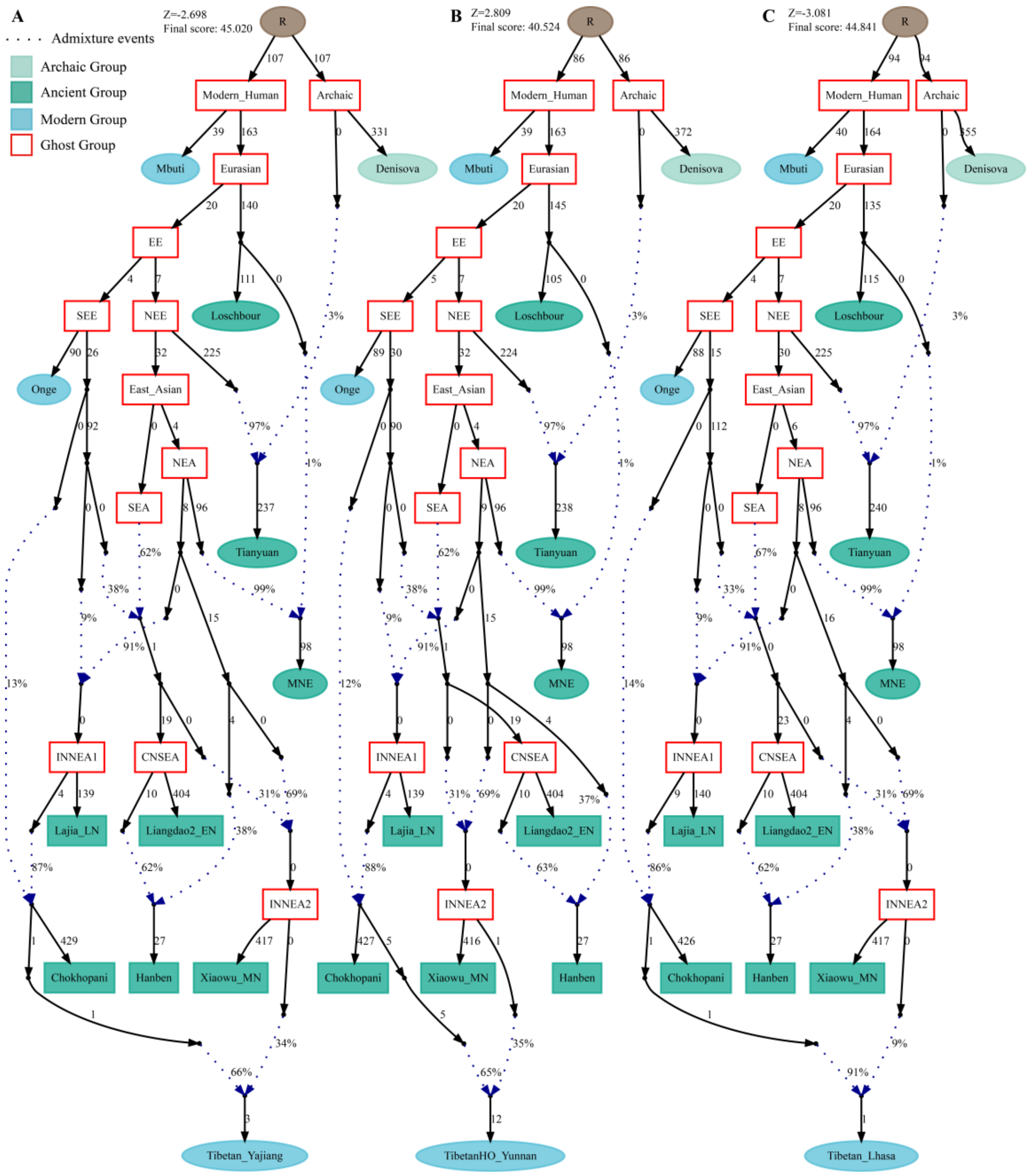

Figure S106. Admixture graph model of modern highland and lowland Tibetans based on the Human Origin dataset using middle Neolithic Xiaowu people as the source of the second migration into Tibet Plateau.

Admixture history of lowland Tibetan from Yunnan (**A**), Tibetan from Yajiang (**B**) and highland Tibetan from Lhasa (**C**). Western Eurasian was represented by Loschbour. Deep southern Eurasian (SEE) and northern Eurasian (NEE) were represented by South Asian Hunter-Gatherer of Onge and 40,000-year-old Tianyuan people. East Asian was subsequently diverged as northern East Asian (NEA) and southern East Asian (SEA). Coastal Neolithic southern East Asian (CNSEA), Inland Neolithic northern East Asian2 (INNEA2) and inland Neolithic northern East Asian1 (INNEA1) were represented by Liangdao2_EN, Xiaowu_MN and Lajia_LN, respectively. All f_4-statistics of included populations are predicted to within 3.018 standard errors of their observed values. Branch lengths are given in units of 1000 times the f_2 drift distance (rounded to the nearest integer). Blue dotted lines denoted admixture events with admixture proportions as shown.

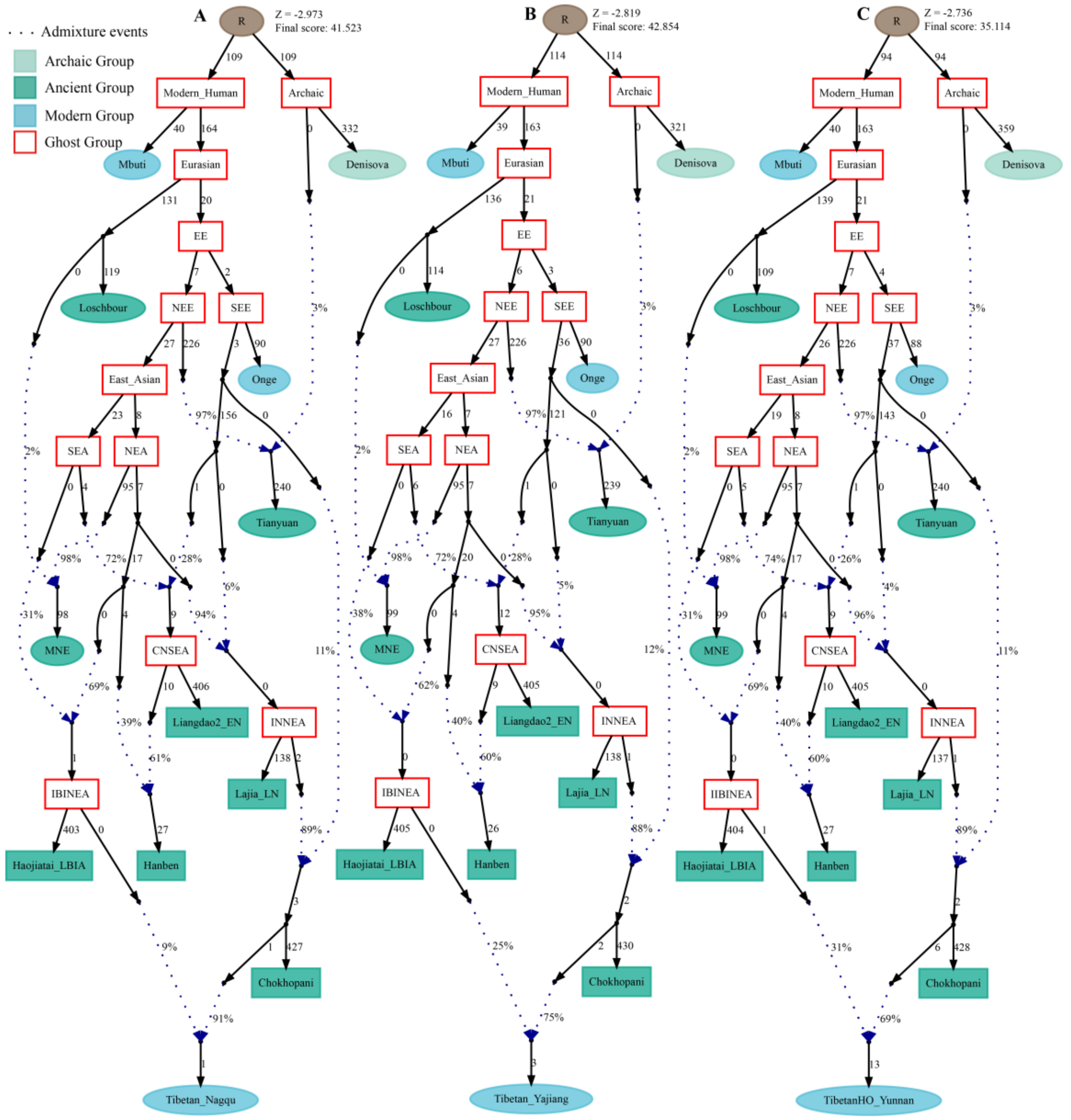

Figure S107. Admixture graph model of modern highland and lowland Tibetans based on the Human Origin dataset using late Bronze Age to Iron Age Haojiatai people as the source of the second migration into Tibet Plateau.

Admixture history of lowland Tibetan from Yunnan (**A**), Tibetan from Yajiang (**B**) and highland Tibetan from Lhasa (**C**). Western Eurasian was represented by Loschbour. Deep southern Eurasian (SEE) and northern Eurasian (NEE) were represented by South Asian Hunter-Gatherer of Onge and 40,000-year-old Tianyuan people. East Asian was subsequently diverged as northern East Asian (NEA) and southern East Asian (SEA). Coastal Neolithic southern East Asian (CNSEA), Inland Neolithic northern East Asian2 (INNEA2) and inland Neolithic northern East Asian1 (INNEA1) were represented by Liangdao2_EN, Haojiatai_LBIA and Lajia_LN, respectively. All f_4-statistics of included populations are predicted to within 2.959 standard errors of their observed values. Branch lengths are given in units of 1000 times the f_2 drift distance (rounded to the nearest integer). Blue dotted lines denoted admixture events with admixture proportions as shown.
